# CAVER 3.0: A Tool for the Analysis of Transport Pathways in Dynamic Protein Structures

**DOI:** 10.1371/journal.pcbi.1002708

**Published:** 2012-10-18

**Authors:** Eva Chovancova, Antonin Pavelka, Petr Benes, Ondrej Strnad, Jan Brezovsky, Barbora Kozlikova, Artur Gora, Vilem Sustr, Martin Klvana, Petr Medek, Lada Biedermannova, Jiri Sochor, Jiri Damborsky

**Affiliations:** 1 Loschmidt Laboratories, Department of Experimental Biology and Research Centre for Toxic Compounds in the Environment, Faculty of Science, Masaryk University, Brno, Czech Republic; 2 Human Computer Interaction Laboratory, Faculty of Informatics, Masaryk University, Brno, Czech Republic; 3 International Centre for Clinical Research, St. Anne's University Hospital Brno, Brno, Czech Republic; UCSD, United States of America

## Abstract

Tunnels and channels facilitate the transport of small molecules, ions and water solvent in a large variety of proteins. Characteristics of individual transport pathways, including their geometry, physico-chemical properties and dynamics are instrumental for understanding of structure-function relationships of these proteins, for the design of new inhibitors and construction of improved biocatalysts. CAVER is a software tool widely used for the identification and characterization of transport pathways in static macromolecular structures. Herein we present a new version of CAVER enabling automatic analysis of tunnels and channels in large ensembles of protein conformations. CAVER 3.0 implements new algorithms for the calculation and clustering of pathways. A trajectory from a molecular dynamics simulation serves as the typical input, while detailed characteristics and summary statistics of the time evolution of individual pathways are provided in the outputs. To illustrate the capabilities of CAVER 3.0, the tool was applied for the analysis of molecular dynamics simulation of the microbial enzyme haloalkane dehalogenase DhaA. CAVER 3.0 safely identified and reliably estimated the importance of all previously published DhaA tunnels, including the tunnels closed in DhaA crystal structures. Obtained results clearly demonstrate that analysis of molecular dynamics simulation is essential for the estimation of pathway characteristics and elucidation of the structural basis of the tunnel gating. CAVER 3.0 paves the way for the study of important biochemical phenomena in the area of molecular transport, molecular recognition and enzymatic catalysis. The software is freely available as a multiplatform command-line application at http://www.caver.cz.

This is a *PLOS Computational Biology* Software Article

## Introduction

Proteins are highly complex systems containing a variety of clefts, grooves, protrusions and empty space in the proteins interior. Besides many tiny cavities, this empty internal spa`ce may form cavities of specific functions, as well as tunnels and channels (or pores), representing potential transport pathways for small molecules, ions and water molecules [1]. Transport pathways play an essential role in the functioning of a large number of proteins. The best known examples include: (i) channels mediating the transport of ions or molecules across biological membranes [2]–[10]; (ii) tunnels facilitating the exchange of ligands between the active site and bulk solvent in enzymes with buried active site cavities [10]–[17]; and (iii) intramolecular tunnels facilitating the transport of reaction intermediates between two distinct active sites in bifunctional enzymes [10], [18]–[22]. The terms tunnel and channel are often used interchangeably in the scientific literature, therefore, we establish following unifying terminology. By channel we mean a pathway leading throughout the protein structure, without any interruption by an internal cavity, with both sides open to the surrounding solvent. By tunnel we mean a pathway connecting a protein surface with an internal cavity or a pathway connecting more than one internal cavity. Accessibility of individual pathways for different substances is largely governed by their size, shape and amino acid composition and can be efficiently modified by protein engineering [4], [15], [23]–[30]. Due to the internal protein dynamics, individual transport pathways and their characteristics may change significantly over time [14], [17], [31], [32]. Therefore, an ensemble of protein conformations, rather than a single static structure, have to be analyzed to get relevant information about the characteristics of individual transport pathways in a given protein [14], [15], [17], [32]–[34].

Recently, several geometry-based tools for rapid identification and analysis of pathways in protein structures have been developed [35]–[44]. The main limitation of these tools is that they were primarily developed for calculation of pathways in static structures. Even though HOLE 2.2 [35], CAVER 1.0 [36], MOLE 1.2 [37] and MolAxis 1.4 [39], [40] can also be used for identification of pathways in molecular dynamics (MD) simulations, their application for systematic studies of dynamic systems is very limited. HOLE 1.0 was the first tool developed for identification of pathways running through macromolecules [35]. Its latest version (HOLE 2.2) supports analysis of structure ensembles. However, HOLE 2.2 cannot be used for calculation of tunnels or multiple pathways, which restricts its application only to the analysis of a single channel penetrating through the protein. Previously, we had developed CAVER 1.0 for analysis of protein tunnels and channels [36]. A tunnel search in CAVER 1.0 is based on a grid approximation causing calculation errors and large demands on processor time and memory [37]. CAVER 1.0 enables calculation of tunnels in an ensemble of structures, but no algorithm for clustering of identified tunnels is implemented. Users have to assign correspondence between tunnels from different snapshots manually, making CAVER 1.0 unsuitable for the analysis of more than tens or hundreds of snapshots.

Some of the limitations of CAVER 1.0 were overcome by MOLE 1.2 [37] and CAVER 2.0 [38]. Instead of grid approximation, these tools employ a Voronoi diagram to describe the skeleton of tunnels within the structure. The remaining shortcoming of both MOLE 1.2 and CAVER 2.0 calculation algorithms is that they construct Voronoi diagram without considering variability in radii of individual protein atoms, thus introducing calculation errors which can be as high as 2 Å for the structures containing hydrogen atoms [39]. For the analysis of MD simulations, MOLE 1.2 employs automatic clustering of tunnels identified throughout the MD simulation based on the sets of tunnel-lining atoms. The introduction of clustering provided significant advance in the analysis of tunnels in the structures from MD simulations. However, the implemented algorithm in MOLE 1.2 clusters each tunnel immediately after its identification, making the results dependent on the order in which individual tunnels are identified. Moreover, the results of MOLE 1.2 are provided separately for each snapshot. MolAxis 1.4 is another Voronoi-diagram based tool for identification of tunnels and channels [39], [40]. Prior to the construction of the Voronoi diagram, MolAxis 1.4 approximates each atom in the analyzed protein by a set of balls (sphere interiors) with the radius of the smallest atom in a given structure, thus avoiding the calculation error observed in the early Voronoi-based tools [39]. Although MolAxis 1.4 provides the opportunity to compute tunnels in multiple structures, it cannot be used for automatic analysis of MD simulations due to the lack of clustering.

Here, we present a new version of CAVER suitable for the effective analysis of tunnels and channels in large ensembles of protein structures. For this purpose, CAVER 3.0 implements new algorithms for both the calculation and clustering of pathways. For the construction of the Voronoi diagram, individual atoms of the analyzed structure are approximated by balls of a fixed size, thus minimizing calculation errors. Similarities between pathways are evaluated based on their geometrical distance. The implemented hierarchical average-link clustering algorithm ensures robust clustering results and enables an easy adjustment of the clustering parameters. We demonstrate that CAVER 3.0 outperforms existing software for geometry-based analysis of pathways in MD simulations. It provides detailed characteristics of individual transport pathways and their time evolution, enables to identify pathways invisible in a static structure and to investigate the structural basis of pathway gating mechanism. This way, CAVER 3.0 opens up new possibilities for the study of important biochemical phenomena, design of biomolecules with appropriate catalytic properties as well as design of inhibitors acting via the blockage of the transport pathways.

## Design and Implementation

CAVER 3.0 is written in the Java programming language and runs on all operating systems with installed Java Runtime Environment 6.0 or higher. The algorithm of CAVER 3.0 consists of three separable steps: (i) identification of pathways in each provided structure, e.g., each snapshot of a MD simulation; (ii) clustering of pathways identified in all snapshots; and (iii) calculation and generation of output data. Due to the separation of the identification and clustering steps, it is possible to run the calculation of pathways in different snapshots in parallel. Moreover, the results of each step can be saved and processed in the subsequent steps with varying parameters, thus accelerating the search for optimal parameters for the studied system.

### 1. Identification of pathways

#### 1.1. Voronoi diagram construction

Representation and processing of Voronoi diagram of balls of equal radii, i.e., ordinary Voronoi diagram of points, is more effective than working with Voronoi diagram of balls with variable radii, i.e., weighted Voronoi diagram, and algorithms for its construction are also better studied [45], [46]. MOLE 1.2 and CAVER 2.0 constructs ordinary Voronoi diagram without considering the differences in radii of individual atoms, i.e., they represent the structure by a set of balls of equal radii, where each ball represents one atom. This can result in the error in the pathway radius estimation as large as *r*_1_−*r*_2_, where *r*_1_ is the radius of the largest atom in the system and *r*_2_ radius of the smallest one. To take differences in atom radii into account and still have molecule represented by balls of equal radii, CAVER 3.0 approximates all atoms in the input structure which are larger than the smallest atom in the structure by a user-specified number of balls with the van der Waals (VDW) radius equal to the VDW radius of the smallest atom, analogously to the solution used in MolAxis 1.4 [39]. By this way, the input structure is represented by a set of balls of equal radii. The representation determines a surface which is never above the VDW surface of the input structure and therefore the pathway radius may be overestimated up to a certain limit, but not underestimated. The upper limits of the overestimation are provided in the output data. If more than four ball centers are co-spherical, more than four Voronoi edges may join in a Voronoi vertex [45]. Handling such degenerate cases complicates design of the algorithm and makes the data structures less efficient. To avoid this, the coordinates of the center of each ball are changed by a pseudorandom value lower than 0.001 Å. In the following text, the resulting set of balls will be referred to as a *representation of input structure* (RIS). Furthermore, the *distance between a point and a ball* will always denote the distance of the point and the closest point on the surface of the ball. Finally, Delaunay triangulation [47] of the RIS centers is used to construct the vertices and edges of the Voronoi diagram [45].

#### 1.2. Cost function

The axes of the pathways are identified as simple paths in a graph composed of Voronoi vertices and edges, i.e., the axis of a pathway is a sequence of Voronoi edges, where each of the two consecutive edges share a vertex. The pathway is then composed of balls with center on the pathway axis and a maximum radius at which the ball does not collide with the RIS. We define the cost of a path so as to reflect the probability that the pathway is actually used as a route for transportation of the substances. In the simplified case of a path of length *L* and constant radius *r*, the cost of the path is
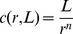
where *n* is a non-negative real number. Therefore, if two paths have equal radii, the shorter has a lower cost. If they have the same length, then the wider has a lower cost, unless *n* is zero. The parameter *n* controls the balance between width and length and can be set to a real number from 0 to 100. If set to 0, only the length of the path is taken into account. On the other hand, if *n* is a high number, a path that is only slightly narrower than the widest path will have a higher cost unless it is many times longer. The default is set to an established value of *n* = 2 to give priority to wide paths with a reasonable length [37], [39]. The cost of a path with continuously changing width can be expressed as
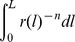



The function *r*(*l*) defines the radius of the largest ball which does not collide with the RIS and is centered at the point on the pathway axis in the distance *l* from the starting vertex measured along the path. The user-defined threshold value *r_max_* is used for *r*(*l*), if the actual radius of the ball is larger than *r_max_*. The minimum value 0.1 Å is used for *r*(*l*) if the actual radius of the ball is smaller than 0.1 Å. The value *L* is the total path length. The cost of the pathway is computed by summing the costs of individual edges forming the pathway axis. The cost of each edge is integrated numerically using trapezoidal rule with a uniform grid. The minimum number of trapezoids is 8 and minimum grid distance 0.1 Å. Prior to the search for the lowest cost paths, all edges which cannot be traversed by a probe of radius of the user-specified value *r_B_* are removed. Because of the approximated VDW molecular surface, some edges appear to be wider than they are in reality. Consequently, some edges are not removed from the Voronoi diagram even though their real width is slightly narrower than *r_B_*. This can happen only if the real edge width is similar to *r_B_*, and may lead to false positive results, i.e., identification of pathways which are in some pathway segment narrower than *r_B_* (Text S1).

Cost of each pathway is transformed into a new measure of pathway importance called *throughput*. A throughput of a pathway is computed as *e^−cost^*, where *e* is Euler's number. Throughput values ranges from 0 to 1—the higher the value, the greater the importance of the pathway. The pathway *A* has a greater throughput than *B* if and only if *A* has a lower cost than *B*.

#### 1.3. Pathway identification

The starting point for the calculation of the pathways is initially placed into the center of gravity of the user-specified residues, atoms or a point defined by *x*, *y* and *z* coordinates. Each of these entities contributes by the same weight. The starting Voronoi vertex is then identified in the vicinity of the initial starting point by the following starting point optimization procedure. The closest Voronoi vertex within the distance *d_max_* from the initial starting point, which is at least *r_min_* far from the RIS, is used as a starting point for the calculation of pathways. If no such vertex can be found, then the vertex with maximum distance to RIS is selected from all vertices located within the *d_max_* distance from the initial starting point. In the case that no vertex exists within the *d_max_* distance, the whole procedure is repeated with the value of 3 Å instead of the user-provided *d_max_* value. If still no vertex is found, the Voronoi vertex closest to the initial starting point is used as the starting Voronoi vertex.

A proper setting of *r_min_* and *d_max_* parameters enables to find an optimal starting point even in cases where the user-specified position of the starting point is too close to RIS, outside (but still close to) the target cavity, or intersects with RIS, thus minimizing the effects of the user-selected starting point location on the calculation results. On the other hand, if a completely wrong initial starting point is selected, the starting point optimization procedure does not help and consequently no or irrelevant pathways are found. It is also important to note that the position of the starting point directly influences overall length of identified pathways and hence also their costs and throughputs. The starting point used for analysis of pathways in a particular macromolecule should be therefore always reported together with the output data to allow reproduction of the calculations.

Pathways are identified as the paths in a graph composed of Voronoi vertices and Voronoi edges. First, two groups of vertices are identified: bulk solvent vertices and surface vertices. A vertex is called a *bulk solvent vertex* if it can be accessed from the structure exterior by a shell probe with a radius *r_S_* (e.g. *r_S_* = 3 Å). Then, a ball called a *bulk solvent ball* is placed into each bulk solvent vertex. The radius of each bulk solvent ball is the largest possible, but satisfying the condition that the ball does not intersect RIS. Next, surface vertices are found by a search in the neighborhood of each bulk solvent ball. Starting in a bulk solvent ball of a center A and radius *r*, a vertex is called a *surface vertex* if it can be accessed by a probe of the radius *r_B_* traveling from A through Voronoi edges so that the probe never intersects RIS and always lies inside a ball of the center A and the radius *r*+*d_s_*, where *d_s_* is by default set to the value of 4 Å. This way, a set of surface vertices and a set of bulk solvent vertices are defined. Furthermore, a *surface boundary vertex* is defined as a surface vertex connected by an edge to a vertex that does not belong to surface vertices. Similarly, a *bulk solvent boundary vertex* is a bulk solvent vertex connected by an edge to a vertex that does not belong to bulk solvent vertices.

The pathway search itself consists of two steps. In the first step, the lowest cost path to every surface boundary vertex is found by Dijkstra's algorithm [48]. In the second step, each path is prolonged by finding a single lowest cost path continuation to a bulk solvent boundary vertex. Then, points are placed on each pathway axis in regular intervals and a ball is placed into each point. The radius of the ball is the maximum possible, but such that the ball does not collide with RIS. Finally, balls from the end of the path are removed one by one until the ball with the radius smaller or equal to *r_S_* is reached.

#### 1.4. Removal of redundant pathways

Several pathways with nearly identical axes may be identified within one snapshot. To remove such redundant pathways, the following iterative procedure is employed. The lowest cost pathway *P* is selected and all pathways within a user specified distance from *P* are discarded. The procedure is repeated with the next remaining lowest cost pathway, until all pathways are either selected or discarded.

### 2. Clustering of pathways

#### 2.1. Calculation of pathway distances

Previously, we proposed a method for estimation of a distance (i.e. dissimilarity) of two pathways, which evaluated the distance between each ball from the first pathway and its respective closest ball from the second pathway [49]. To accelerate the computation of all pairwise pathway similarities, we have now designed a more efficient algorithm. The basic idea is to represent each pathway by a sequence of *N* points and then compute only distances between pairs of points with the corresponding order, thus performing only *N* distance computations for each pair of pathways. First, a point *S* is constructed as the center of gravity of the starting Voronoi vertices from all snapshots. Then, a set of points *P* is constructed iteratively by processing the pathways ordered by their cost in ascending order. For the pathway being processed, *an extreme point X* is identified as the pathway axis point of the greatest distance from *S*. The distance |*SX*| will be called *a straight length* of a pathway. Then, the point *Y* from *P* is identified such that the angle *XSY* is minimal. If *P* is still empty or the angle size is greater than the user specified value, the extreme point *X* is added to *P* and its weight is set to one. Otherwise, *X* is discarded and the point *Y* is moved along the *SY* half-line so that its new distance to *S* is |*SX*|+*w_Y_*·|*SY*|, where *w_Y_* is the weight of the point *Y*. The weight of the point *Y* is then increased by one. In the end, each point in the set *P* captures averaged straight lengths of pathways whose end points are close to each other.

Next, all the pathways are processed once more. First, a set of points *Q* is identified within *P* such that for the extreme point *X* of the actually processed pathway and any point *Y* from *Q*, the size of the angle *XSY* is not greater than a parameter called smoothness. Then, the distance *d_end_* is computed as
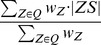
Thus an average of straight lengths of the pathways in the vicinity of the actually processed pathway was estimated.

Next, the geometry of each pathway axis is converted into *N* points by the following procedure. For each pathway, the distance *d_end_* is divided into a sequence of *N*+2 intervals *I_start_*, *I_1_*, *I_2_*, …, *I_N_* and *I_end_*. The size of the intervals *I_start_* and *I_end_* is *z_start_* and *z_end_*, where values *z_end_* and *z_start_* may be specified by the user and the intervals themselves determine the pathway beginning and end sections that are not used for the evaluation of pathway similarities. For each *i* from 1 to *N*, the center of gravity *G_i_* of all points whose distance from *S* falls within the interval *I_i_* is computed. Thus, each pathway is characterized by a sequence of points *G_1_*, *G_2_*, *_…_*, *G_N_*. The distance of two pathways *A* and *B* is defined as
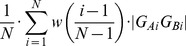
where |*G_Ai_G_Bi_*| denotes the Euclidean distance of two corresponding points from pathways *A* and *B* and *w* is a linear function *w*(*x*) = *ax*+*b*. The coefficients *a* and *b* are derived from the user defined weighting parameter *q* so that the ratio between the weights of the end point *w*(1) and starting point *w*(0) equals *q* and *w*(0.5) equals 1. The weighting of the point distances allows the user to emphasize either the importance of the pathway end section by setting *q*>1, or of the pathway beginning section by setting *q*<1. In practice, the weighting is performed by identification of such points *K_i_* lying on the halfline *SG_i_* that
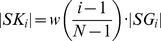
and then the distance of the pathways *A* and *B* is calculated as
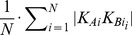



Pathway distances are calculated on the fly during the above described algorithm for the removal of redundant pathways, as well as for all pairs of non-redundant pathways identified throughout the MD simulation, which is used to establish the correspondence between pathways in different snapshots. The matrix of pairwise distances of all non-redundant pathways is stored on the hard disk for further processing. The presented algorithm for the calculation of pathway distances has several advantageous properties: (i) the algorithm is fast and scalable as only a small and adjustable number of distances need be evaluated for each pair of pathways; (ii) each pathway is characterized by the same number (3*N*) of coordinates, which allows fast clustering of pathways; and (iii) pairwise distances of pathways can be calculated using only selected pathway sections and/or the importance of the pathway beginning or end sections can be emphasized according to the needs of individual users.

#### 2.2. Clustering of pathways

The clustering of pathways is based on the pre-calculated distance matrix. Average-link hierarchical clustering [50] is used to construct a tree hierarchy of pathway axes based on their pairwise distances. The size of the clusters may be then optimized rapidly by cutting the tree at a varying level of detail. This accurate clustering becomes demanding with respect to computer time and disk capacity for datasets containing more than 50,000 pathways. Therefore, a fast approximate algorithm based on supervised machine learning was also implemented, enabling the classification of even larger datasets with hundreds of thousands of pathways. In the first step, a set of pathways of manageable size is randomly sampled from the pathway dataset and clustered using the average-link hierarchical clustering. Each pathway is then assigned to one of the *N*+1 classes. *N* classes correspond to the *N* clusters with highest priority and the last class contains pathways belonging to all remaining clusters. This dataset is then used for training a *k*-nearest neighbor classifier as implemented in the program Weka 3.6 [51]. Finally, the classifier is used to assign each of the remaining pathways from the original dataset either to one of the *N* clusters or to the last class containing pathways from clusters with lower priorities.

### 3. Calculation and generation of output data

The identified pathway clusters are ranked by their property called *priority*, which is calculated as a sum of throughputs of all pathways in a given cluster, divided by the total number of snapshots that were analyzed. Thus, both the number of pathways in the cluster and their throughputs contribute to the pathway ranking. If the cluster contains more pathways that were identified in the same snapshot, only the pathway with the highest throughput is considered.

The pathways are represented by a sequence of balls centered at the points placed in regular intervals along the pathway axis. Each ball has the maximum possible radius such that the ball does not intersect RIS. The pathway representation shows the maximum size of the spherical probe which can travel through the individual pathway segments. It is important to note that the pathway representation does not provide information about the total volume of cavity or void around the pathway.

An atom and its corresponding residue are considered as pathway-lining, if the distance of the atom's surface to the surface of some pathway ball is smaller than a user defined threshold value. A Java implementation of KD tree [52] downloaded from http://home.wlu.edu/~levys/software/kd is used for efficient spatial searching. The smallest ball of the pathway, i.e. the narrowest part, will hereafter be called the *pathway bottleneck*. Bottleneck residues are identified as residues with at least one atom within the user specified distance from the pathway bottleneck. For visualization purposes, the pathways are stored in PDB file format. The PDB files are accompanied by Python and Tcl scripts, simplifying visualization of the results in PyMOL [53] and VMD [54].

Pathway profiles and characteristics are provided in the comma-separated values (.csv) file format. Radii of each pathway are measured in regular intervals along the pathway axis, thus providing information on the pathway profile. Besides that, the pathway radius is also estimated in regular intervals of the distance from the point *S* (center of mass of the starting vertices). These pathway profiles, calculated as the dependence of the pathway radius on the distance measured along a straight line, are more straightforwardly aligned to one another than the profiles calculated along the pathway axis. The aligned pathway profiles are visualized as *heat map* images, where the color of the point with coordinates *x* and *y* expresses the radius of a given pathway in the *x*-th snapshot and the *y*-th distance interval. Additionally, the time evolution of pathway bottlenecks radii can be visualized as heat map where the color of the point with coordinates *x* and *y* expresses the bottleneck radius of the y-th pathway in the *x*-th snapshot.

## Results

### Description of the software tool

CAVER 3.0 was primarily developed for the analysis of protein structures, but it can be used for analysis of any molecular structure containing tunnel-like features. The user has to provide a structure or a set of aligned structures with consistently numbered atoms and residues in PDB format as an input and specify the starting point of the calculation. For the clustering of the identified pathways, the user can choose either an accurate hierarchical average-link clustering algorithm or the combination of this clustering with supervised machine learning classification, which enables fast processing of very large datasets containing hundreds of thousands of pathways. A number of additional settings are available, enabling users to adjust the calculation and clustering parameters based on their needs. All results are presented in a comprehensive way. Output data from the calculation includes: (i) characteristics of individual pathways summarized over all analyzed snapshots—frequency and priority of a given collective pathway, mean and maximum radius of the narrowest region (bottleneck), mean length, curvature and throughput of a collective pathway; (ii) characteristics of individual pathways in individual snapshots, enabling to track the changes of the collective pathways over time—bottleneck radius, length, curvature, throughput, cost; (iii) data for plotting of the pathway profiles—radius over distance from the start; (iv) data for plotting of bottleneck radius and throughput histograms; (v) list of atoms and residues lining individual collective pathways; (vi) list of the residues making up the bottleneck; (vii) heat maps showing time evolution of the bottleneck radii and pathway profiles; and (viii) scripts for visualization of the results in PyMOL and VMD. The individual pathways making up the collective pathways can either be visualized in a single frame, which is useful for the evaluation of the clustering results and for a general overview of the collective pathway variability, or as a MD trajectory tracking the changes of the collective pathways over time.

### Comparison with other tools

#### Identification of pathways in static crystal structures

CAVER 3.0 was tested on a variety of biomolecular structures (Protocol S1), which had previously been used for the validation of MOLE 1.2 [37] and MolAxis 1.4 [40]. Comparison of the results obtained by CAVER 3.0, MOLE 1.2 and MolAxis 1.4 with the identical set of protein structures is shown in Supporting Table S1. The pathways identified by CAVER 3.0 are generally shorter than the pathways identified by MOLE 1.2 and MolAxis 1.4 due to the more accurate definition of protein surface at which the pathways end. MOLE 1.2 tends to identify narrower pathways than the two other tools. The differences in the pathways identified by MOLE- are caused by the construction of the Voronoi diagrams without considering the variability in atom radii. MOLE 1.2 also employs a distinct search strategy—CAVER 3.0 and MolAxis 1.4 search for all pathways simultaneously, while MOLE 1.2 performs a consecutive search and penalizes repeated searches of the same parts of the Voronoi diagram.

#### Identification of pathways in molecular dynamics trajectories

**Figure 1:**
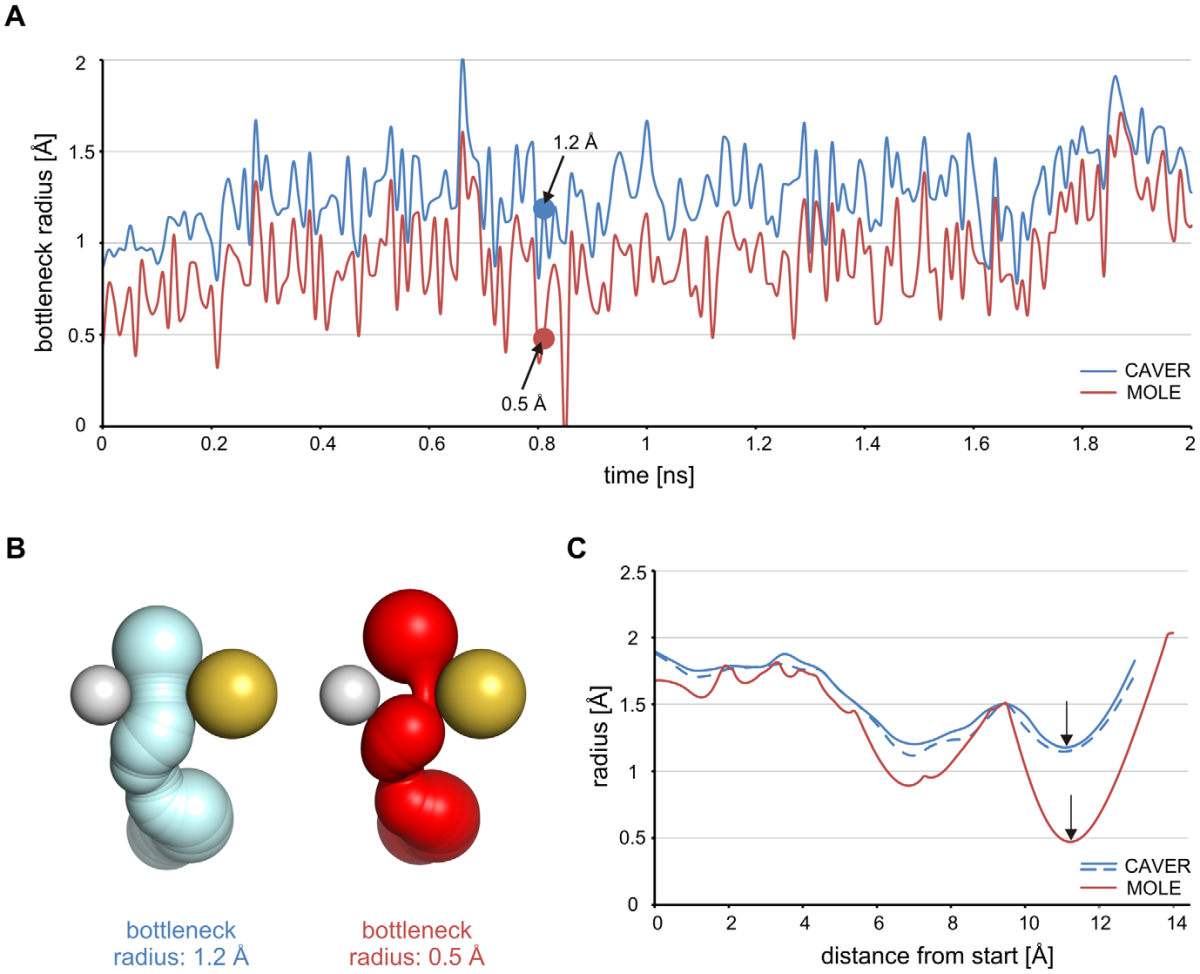
Comparison of the p1 tunnel of DhaA calculated by CAVER 3.0 and MOLE 1.2. (A) Time evolution of the bottleneck radius calculated by CAVER 3.0 (blue) and MOLE 1.2 (red). Only a part of the 10 ns MD simulation is shown for clarity. The sample snapshot (black arrows) was taken at 0.81 ns. (B) Geometry of the p1 tunnel in the sample snapshot calculated by CAVER 3.0 (blue) and MOLE 1.2 (red). Hydrogen atom of the bottleneck residue Ala145 (white ball) is shown together with the sulfur atom of the bottleneck residue Cys176 (yellow ball) and with sphere representation of the tunnels. The underestimation of the bottleneck radius by MOLE 1.2 is visible as an empty space between the tunnel and the hydrogen atom of Ala145. (C) Profile of the p1 tunnel in the sample snapshot calculated by CAVER 3.0 (blue) and MOLE 1.2 (red). The dashed line indicates the profile representing the maximum possible difference between the CAVER-calculated (solid line) and the correct profile of the p1 tunnel.

CAVER 3.0 and MOLE 1.2 were used for the analysis of 1,000 snapshots from a 10 ns MD simulation of haloalkane dehalogenase DhaA using various settings of clustering parameters (Protocol S2 and S3). MolAxis 1.4 does not employ an algorithm for pathway clustering and therefore is not suitable for automatic analysis of tunnels in MD simulations. The average-link algorithm implemented in CAVER 3.0 provided robust results with better separated clusters than the clustering algorithm implemented in MOLE 1.2 (Figure S1). Another advantage of the hierarchical algorithm implemented in CAVER 3.0 is the possibility to modify the results from clustering by adjusting the threshold value without a need to repeat the clustering procedure. This significantly speeds-up the search for the optimal clustering parameters. CAVER 3.0 additionally provides other options to control the clustering results—based on the user-defined parameters, the pairwise distance of the pathways may be calculated using only selected pathway sections, with constant weights along the entire pathway length or with a higher weight on either the beginning or end sections of the pathway. Settings that provided comparable clusters for the p1 tunnel (previously also named the upper or main tunnel [36]) were selected and the time evolution of the p1 tunnel calculated by both tools was compared (Figure S1C and S1F). This comparison revealed that the average bottleneck radius of the p1 tunnel calculated by CAVER 3.0 (1.4 Å, mean error<0.04 Å) differs from that calculated by MOLE 1.2 (1.1 Å; Figure 1A). This discrepancy is due to the calculation error of MOLE 1.2 (Figure 1B), which is proportional to the difference between the radii of individual atoms making up the pathway and considerably varies in individual snapshots. For example, in a sample snapshot, MOLE 1.2 identified a p1 tunnel with a bottleneck radius of 0.5 Å, while the bottleneck calculated by CAVER 3.0 was 1.2 Å with the maximum approximation error of 0.04 Å (Figure 1C).

### Case study

#### Description of model system

Haloalkane dehalogenase DhaA isolated from various strains of *Rhodococcus* spp. [55]–[58] catalyzes hydrolytic cleavage of the carbon-halogen bond in a variety of halogenated compounds. The active site of DhaA is buried inside the protein core and is connected with the outside solvent by access tunnels [59]. The access tunnels of DhaA were studied in detail using classical and random acceleration molecular dynamics (RAMD) simulations [15], [60]. This study suggested five pathways for the release of products and/or the exchange of water solvent, named p1, p2a, p2b, p2c and p3 [15]. Key residues of the access tunnels identified by RAMD were randomized by directed evolution, resulting in 25 unique mutants with higher activities towards toxic recalcitrant compound 1,2,3-trichloropropane, with the best mutant showing 32-fold improvement over the wild type enzyme [28]. The wealth of available knowledge on access tunnels of DhaA makes this enzyme a good model system to validate the ability of CAVER 3.0 to identify and characterize tunnels in trajectories from MD simulations.

#### Identification of tunnels

**Figure 2:**
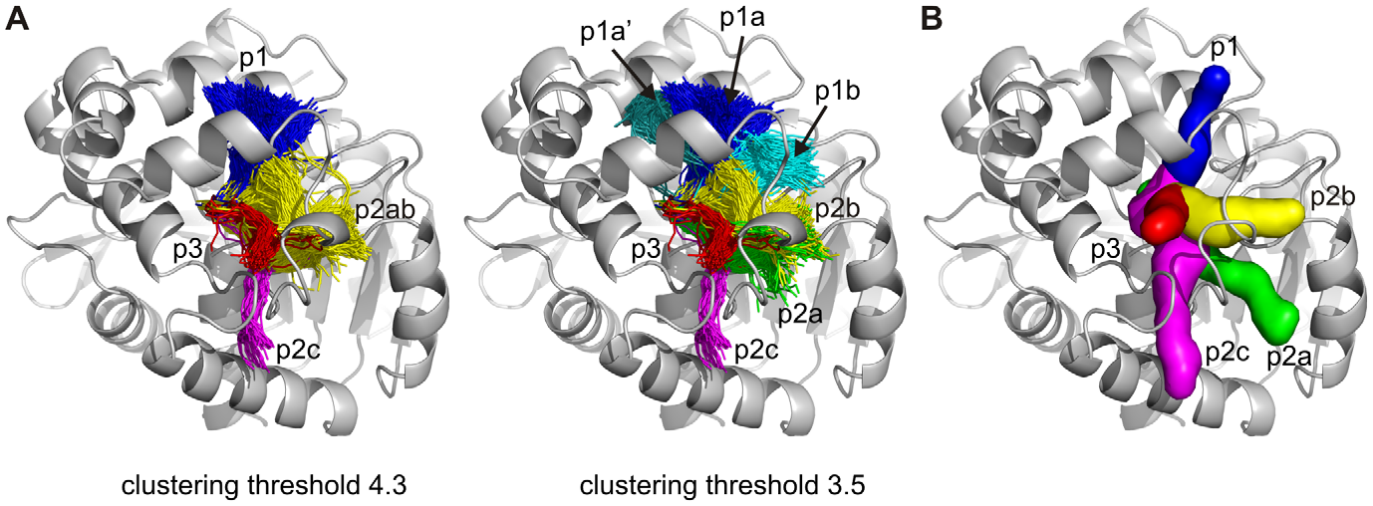
Comparison of the DhaA tunnels identified by CAVER 3.0 with the previously proposed pathways. (A) The top ranked collective pathways identified throughout the molecular dynamics simulation of DhaA by CAVER 3.0 are all depicted in one frame as pathway centerlines. The p2a and p2b tunnels were initially identified as one collective pathway—p2ab—using the clustering threshold of 4.3. Decreasing the clustering threshold to 3.5 led to the separation of the p2a and p2b tunnels as well as the splitting of the p1 collective pathway into three clusters—p1a, p1a′ and p1b. A random subsample of identified tunnels is shown for clarity. (B) Representative DhaA pathways (surface representation) for the release of products and/or exchange of water solvent as identified previously by RAMD and classical MD simulations [15].

CAVER 3.0 was used for the analysis of 10,000 snapshots from a 10 ns classical MD simulation of DhaA in explicit water solvent (Protocol S2 and S3). In each snapshot, all possible pathways with the bottleneck radius equal or larger than 0.9 Å were identified, leading to a set of nearly 30,000 pathways. The pathways were clustered by the average-link algorithm based on the pairwise distances of the pathways. All five previously described DhaA tunnels [15] were identified among the top four ranked pathway clusters (Figure 2, Table S2). The p2a and p2b tunnels possess a common opening and thus were initially identified as a single pathway (p2ab). Therefore, the clustering threshold was decreased in order to evaluate the relative importance of the p2a and p2b tunnels to each other (Figure 2). We note that the localization of the p2a and p2b tunnels differs slightly from the study of Klvana *et al.* [15], where these tunnels were observed to run roughly parallel to each other. In our analysis, we found out that these tunnels may cross each other and consequently four different pathways may in theory be identified by geometrical analysis. The lower clustering threshold also led to the splitting of the p1 tunnel into three branches—the dominant p1a tunnel, corresponding to the p1 tunnel observed in previous analyses [15] and the less frequent and narrower p1a′ and p1b tunnels (Figure 2, Table 1).

**Table 1:** Characteristics of the top ranked tunnels of DhaA identified by CAVER 3.0 in molecular dynamics trajectory using the probe radius of 0.9 Å and the clustering threshold of 3.5.

Tunnel	p1a	p1a′	p1b	p2a	p2b	p2c	p3
**Cluster ranking**	1	5	4	3	2	6	7
**Snapshots** a	9958	409	743	1428	5011	120	131
**Snapshots with an open tunnel** b	5292	0	5	3	82	0	0
**Mean bottleneck radius [Å]** c	1.4	1.0	1.0	1.0	1.0	1.0	0.9
**Max. bottleneck radius [Å]**	2.3	1.3	1.8	1.5	1.8	1.2	1.2
**Mean throughput** c	0.621	0.432	0.479	0.403	0.475	0.343	0.304

a number of snapshots in which at least one pathway with bottleneck radius ≥0.9 Å was identified;

b number of snapshots in which at least one pathway with bottleneck radius ≥1.4 Å was identified;

c characteristics averaged over identified pathways (i.e. pathways with bottleneck radius ≥0.9 Å), real values will be lower, especially for p1a′, p1b, p2a, p2c and p3 tunnels, which were identified only in a small portion of snapshots.

**Figure 3:**
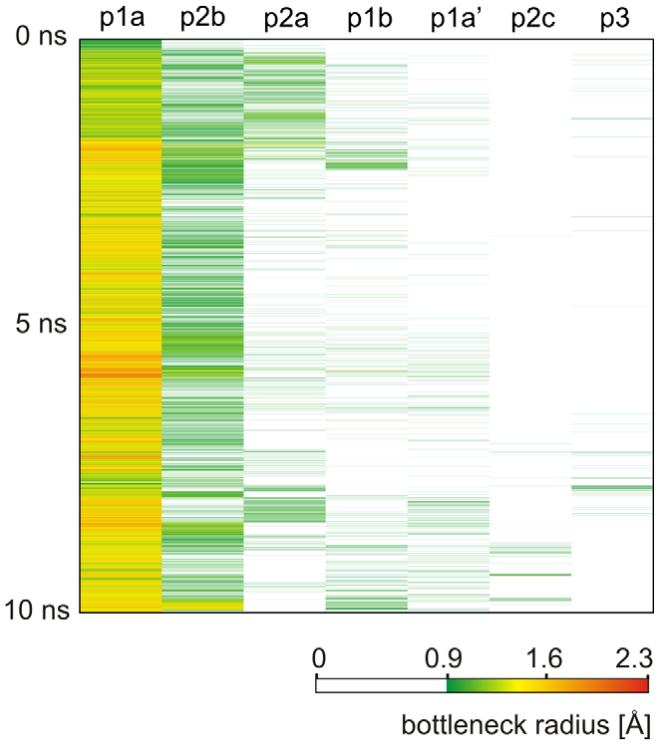
Time evolution of the bottleneck radii of DhaA tunnels identified by CAVER 3.0. The color map ranges from very narrow (green) to wide (red) bottlenecks. White color indicates that no pathway with bottleneck radius ≥0.9 Å was identified for the given pathway cluster in the given snapshot.

We found a good agreement between the results of CAVER 3.0 and the previous MD and RAMD study of DhaA product release pathways [15] (Text S2): (i) all five previously proposed DhaA pathways were reliably identified by CAVER 3.0, with estimated relative importance p1≫p2b≫p2a>p2c∼p3; (ii) the p1 tunnel was found to be the dominant transport pathway—it was the most frequently identified collective pathway, had by far the highest maximum and mean radii of bottlenecks and was frequently open for water molecules (Table 1, Figure 3); (iii) based on all studied characteristics, the p2b and p2a tunnels were found to be the second and the third most important, respectively; (iv) the p2c and p3 pathways were only rarely identified, however, compared to other possible tunnels ranked at lower places, the p2c and p3 pathways were still significantly more frequent and showed a considerable widening of the bottlenecks in some snapshots (up to 1.2 Å, Table S2).

It is important to note that RAMD and other methods specialized in the simulation of ligand migration through protein structure provide information specific to a particular ligand [31]. The specificity of obtained results represents an advantage in many instances, but it may become a drawback if the user aims for a general overview of the possible transport pathways in the studied protein. In contrast, CAVER 3.0 facilitates the analysis of pathways in classical MD simulations and thus provides a general picture of the variability of potential transport pathways.

#### Analysis of tunnel dynamics

**Figure 4:**
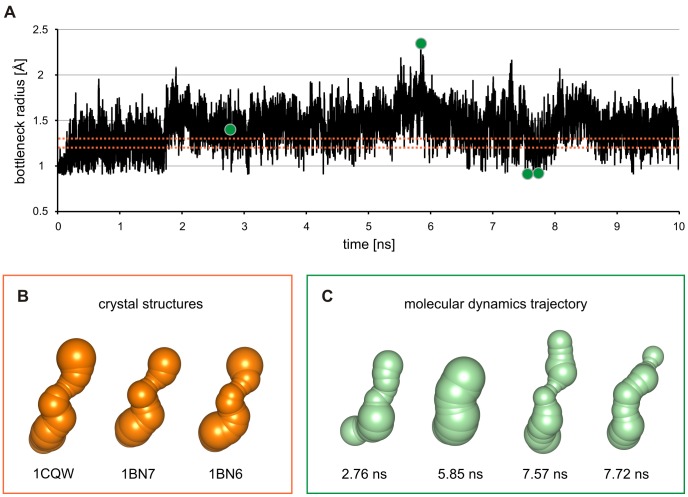
Time evolution of the DhaA p1 tunnel. (A) Evolution of the p1 tunnel bottleneck radius over time. The dotted orange lines indicate bottleneck radii of the p1 tunnel in DhaA crystal structures with added hydrogen atoms: PDB-IDs 1BN6 and 1BN7 (bottleneck radius 1.2 Å) and 1CQW (1.3 Å). The four green circles indicate bottleneck radii of the p1 tunnel from the 2.76 ns (bottleneck radius 1.4 Å), 5.85 ns (2.3 Å), 7.57 ns (0.9 Å) and the 7.72 ns (0.9 Å) snapshots from the molecular dynamics simulation of DhaA. (B) The p1 tunnel identified in DhaA crystal structures with added hydrogen atoms (PDB-IDs 1CQW, 1BN6, 1BN7). (C) The p1 tunnel identified in the 2.76 ns, 5.85 ns, 7.57 ns and 7.72 ns snapshots of the MD trajectory of DhaA.

The capability of CAVER 3.0 to analyze dynamical systems is essential for the identification of biochemically relevant tunnels. All previously known DhaA tunnels were reliably identified by the analysis of the 10 ns MD simulation snapshots, while only two of them—p1 and p2ab—were found in the crystal structures of DhaA (PDB-IDs 1CQW, 1BN7 and 1BN6) under the same calculation settings (Protocol S4). By decreasing the probe radius from 0.9 Å to 0.8 Å, more than ten potential tunnels were identified in each crystal structure, including the p1 variants (p1a, p1a′ and p1b), p2ab and p3 tunnels (Table S3). The p2c tunnel was not observed. While the p1 tunnel was clearly wider and had higher throughput than all other tunnels in all three crystal structures, the remaining tunnels (with the exception of p2a and p2b in the structure PDB-ID 1BN6) had very similar characteristics to one another (Table S3), demonstrating that it is difficult to identify the biochemically relevant tunnels by analyzing the static crystal structures only. Such analysis may easily overlook relevant tunnels that are temporarily closed, and/or identify tunnels whose relevancy is disputable. In contrast, tunnel characteristics obtained from the analysis of MD simulation, such as the frequency of tunnel identification, the frequency of tunnel opening or the bottleneck radius fluctuation, may be used as indicators of the tunnel's functional relevancy (Table S2). This can be demonstrated on the exemplary analysis of the time evolution of the p1 tunnel bottleneck. In all three available crystal structures of DhaA with added hydrogen atoms, the value of the bottleneck radius 1.2 Å (PDB-IDs 1BN6, 1BN7) and 1.3 Å (PDB-ID 1CQW) indicates that the p1 tunnel may not be relevant, considering the usual probe radius of 1.4 Å. In the MD simulation, however, the p1 tunnel was open in a large number of snapshots (bottleneck radius wider than 1.4 Å), suggesting the importance of the tunnel for enzyme function (Figure 3 and Figure 4). The maximum radius of the p1 tunnel bottleneck in the MD simulation was 2.3 Å (Figure 4 and Table S4).

#### Analysis of bottleneck dynamics

The residues forming the tunnel bottleneck represent promising hotspots for the modification of tunnel properties, since their substitutions have potentially the most pronounced impact on the tunnel geometry. Similarly to other tunnel characteristics, the analysis of the bottlenecks is more informative when the dynamics of the protein is considered. The list of the bottleneck residues obtained by the analysis of the MD trajectory using CAVER 3.0 suggested the existence of two distinct bottlenecks along the DhaA p1 tunnel, while only one of them can be identified in the crystal structures (Table S4). The analysis of the bottlenecks in the MD trajectory also revealed the structural details of the tunnel gating.

**Figure 5:**
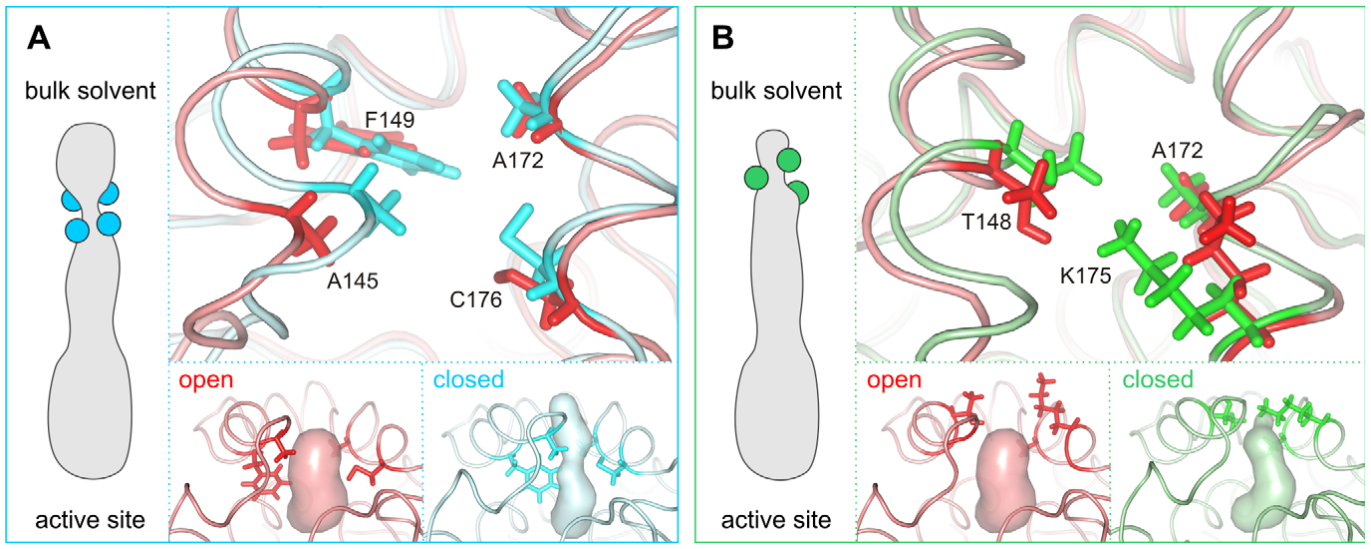
Bottleneck dynamics and structural basis of gating in the p1 tunnel of DhaA. (A) The bottleneck 1 represents the most frequent bottleneck of the p1 tunnel and is mostly formed by Ala145, Phe149, Ala172 and Cys176. Comparison of snapshots with an open (red) and closed (blue) p1 tunnels suggested that the gating is mediated by: (i) movement of the N-terminal part of the cap domain carrying Ala145 and Phe149; (ii) movement of the α5-helix with Cys176 and Ala172; and (iii) conformational change of the bottleneck residues Phe149 and Cys176. The bottleneck radius in the selected snapshots with an open and closed tunnel was 2.3 Å and 0.9 Å, respectively. (B) The bottleneck 2 of the p1 tunnel is mostly formed by Thr148, Ala172 and Lys175. Comparison of snapshots with an open (red) and closed (green) p1 tunnel suggested that gating is mediated by: (i) movement of the N-terminal part of the cap domain carrying Thr148; (ii) movement of the α5-helix carrying Ala172; and (iii) the conformational change of the bottleneck residues Thr148 and Lys175. The bottleneck radius in the selected snapshot with an open and closed tunnel was 2.3 Å and 0.9 Å, respectively.

The most frequent bottleneck in the DhaA p1 tunnel is formed mainly by Phe149 (71% of p1 tunnels), Cys176 (59%), Ala172 (50%) and Ala145 (38%) residues, mostly in combinations Ala145-Phe149-Cys176 and Phe149-Ala172-Cys176 (26% and 24% of all p1 pathways, respectively). Comparison of structures with an open and closed p1 tunnels suggested that the gating in this bottleneck is mediated by the movement of the N-terminal part of the cap domain (Glu139-Phe149) carrying the bottleneck residues Ala145 and Phe149, movement of the C-terminal part of the α5-helix with the bottleneck residue Cys176, and by the conformational change of the bottleneck residues Phe149 and Cys176 (Figure 5A). The proposed structural basis of gating in the bottleneck is in agreement with the results from RAMD analysis [15].

The second bottleneck is positioned at the tunnel entrance from the bulk solvent and is formed predominantly by Lys175 (26% of all p1 pathways), Thr148 (23%) and Ala172 (50%; also included in the first bottleneck), most often in the combination Thr148-Ala172-Lys175 (16% of all p1 pathways). The gating in the second bottleneck seems to be mediated mainly by the conformational change of Lys175 (Figure 5B). This residue has previously been observed to participate in the chloride ion release during MD simulations [15]. The gating is also facilitated by the movement of the N-terminal part of the cap domain (Glu139-Phe149) with the bottleneck residue Thr148, by the conformational change of Thr148 and by the movement of the α5-helix with the bottleneck residue Ala172. Unlike the first bottleneck, the second one cannot be observed in the crystal structures, and can only be identified by the analysis of MD trajectories.

The importance of the bottleneck residues identified by CAVER 3.0 for the catalytic properties of DhaA had already been demonstrated experimentally (Table S5, Table 2). Two independent directed evolution experiments provided DhaA variants C176Y+Y273F and C176F+G3D with 3.5-times [61] and 4-times [62] higher activity towards the important environmental pollutant 1,2,3-trichloropropane (TCP). Both these variants carried mutation in the position Cys176, which forms the bottleneck of the p1 tunnel. In another study [28], the DhaA variant C176Y+Y273F was used as a template for focused directed evolution, subjecting Ile135 (bottleneck of p2a, p2c and p3 tunnels), Val245 (bottleneck of p2b and p1b tunnels) and Leu246 (bottleneck of p2a and p2b tunnels) to simultaneous saturation mutagenesis. The best variant I135F+C176Y+V245F+L246I+Y273F showed 32-times higher activity towards TCP than the wild type enzyme, which is due to decreased accessibility of buried active site for water molecules [28]. A focused directed evolution of the DhaA variant H272F was conducted to improve the binding of a fluorescent probe into the enzyme active site [63]. The best variant K175M+C176G+Y273L, with four orders of magnitude improved binding rate, carried two substitutions in the p1 tunnel bottlenecks (K175M and C176G) and one in the active site cavity (Y273L). Altogether, these four examples demonstrate that analysis of access tunnels using CAVER 3.0 can be useful for the selection of suitable hot-spots for engineering of enzyme catalytic properties. The study of dynamical systems enables the identification of all important bottlenecks and provides invaluable information about their relative relevance.

**Table 2:** DhaA variants with modified catalytic properties, carrying substitutions in the bottleneck residues (in bold) identified by CAVER 3.0.

Mutations	Effect	Reference
**C176Y**+Y273F	3.5-times higher activity with TCP	[61]
**C176F**+G3D	4.0-times higher activity with TCP	[62]
**I135F+C176Y+V245F+L246I**+Y273F	32-times higher activity with TCP	[28]
**K175M+C176G**+Y273L	10,000-times higher binding rate of fluorescent probe	[63]

## Conclusions

CAVER 3.0 is a new tool for geometry-based analysis of pathways in both static and dynamic protein structures. For the analysis of dynamical systems, the software implements several new algorithms for accurate calculation and clustering of pathways. CAVER 3.0 requires a protein structure or a set of aligned structures and the specification of the calculation starting point as the only obligatory inputs. A number of additional settings are available, enabling users to customize the calculation based on their needs. In the output, CAVER 3.0 provides all necessary data for the analysis of the time evolution of individual pathways.

To demonstrate the capabilities of CAVER 3.0, we used it for the analysis of tunnels in a molecular dynamics simulation of the microbial enzyme haloalkane dehalogenase DhaA. We were able to identify and reliably estimate the importance of all previously published DhaA tunnels, including tunnels closed in DhaA crystal structures as well as to correctly predict the bottleneck residues important for the catalytic function of this enzyme. Detailed investigation of the dynamics of the p1 tunnel revealed the structural basis of tunnel gating. The obtained results demonstrate that the analysis of static structures can lead to overlooking of relevant auxiliary tunnels or prediction of tunnels whose relevancy is disputable.

In summary, CAVER 3.0 enables an effective analysis of pathways in dynamical protein structures, which provides important insights into the structure-function relationships of proteins and facilitates the design of improved biocatalysts and new inhibitors.

## Availability

CAVER 3.0 is a multiplatform command-line Java application. The software is licensed under the GNU General Public License v3.0 and is freely available at http://www.caver.cz or as supplementary material accompanying this paper (Software S1).

## Supporting Information

Figure S1 Clusters of pathways calculated in the molecular dynamics simulation of haloalkane dehalogenase DhaA by CAVER 3.0 (A–C) and MOLE 1.2 (D–F). Pathways identified throughout the simulation are shown in one frame as pathway centerlines; if more pathways from the same snapshot are grouped to the same cluster, only the pathway with the lowest cost is shown. The variants of the p1 (main) tunnel are in different shades of blue, variants of the p2 tunnel in yellow, green and magenta and the p3 tunnel is in red. (A–C) CAVER 3.0 results for different settings of the clustering threshold. (A) Clustering was performed with constant weights along the entire pathway and low clustering threshold of 3.5. The p1 pathways with dispersed openings as well as the p2a and p2b pathways which have a common opening are separated into different clusters. (B) Increasing the clustering threshold led to the joining of the p2a and p2b pathway clusters. (C) Further increase of the clustering threshold led to the grouping of all the p1 pathways into a single cluster. Note that some of the previously visible p1 pathways are not visible after the change of threshold since in individual snapshots, only the pathway with the lowest cost is shown for each cluster. (D–F) MOLE 1.2 results for different settings of the clustering parameters. (D) The parameters were set to distinguish the known variants of the p2 tunnel; the p2a and p2b pathway clusters are not well defined as they largely overlap along the entire tunnel length. The p1 tunnel was divided into multiple clusters. (E) Recalculation with a lower value of the bound parameter led to the grouping of a portion of the p1 pathways into one cluster, while other p1 pathways remained separated. The p2a and p2b clusters are not well defined—part of the p2b cluster overlaps with the p2a cluster and part with the p1b cluster. (F) The bound parameter was optimized to join all the p1 pathways into a single cluster. This led to also the p2a and p2b pathways being clustered together; part of the p2ab cluster overlaps with the p2c cluster. Note that many of the previously visible p1 pathways are not visible, since in individual snapshots, only the pathway with the lowest cost is retained for each cluster.
(TIF)

Protocol S1 Comparison of CAVER 3.0, MOLE 1.2 and MolAxis 1.4.
(PDF)

Protocol S2 Molecular dynamics simulation of haloalkane dehalogenase DhaA.
(PDF)

Protocol S3 Analysis of molecular dynamics simulation of DhaA.
(PDF)

Protocol S4 Analysis of crystal structures of DhaA.
(PDF)

Software S1 CAVER 3.0 package containing CAVER 3.0 executable, source code, license, documentation and examples. The latest release of CAVER 3.0 can be downloaded from http://www.caver.cz.
(ZIP)

Table S1 Comparison of pathways calculated by CAVER 3.0, MOLE 1.2 and MolAxis 1.4.
(PDF)

Table S2 Characteristics of the pathways identified in 10,000 snapshots of the 10 ns molecular dynamics trajectory of DhaA using the probe radius of 0.9 Å and the clustering threshold of 4.3.
(PDF)

Table S3 Characteristics of the pathways identified in DhaA crystal structures using the probe radius of 0.8 Å.
(PDF)

Table S4 Comparison of characteristics of the DhaA p1 tunnel obtained by the analysis of the molecular dynamics trajectory and crystal structures.
(PDF)

Table S5 Bottleneck residues of the top ranked tunnels of DhaA identified by CAVER 3.0 in molecular dynamics trajectory using the probe radius of 0.9 Å and the clustering threshold of 3.5.
(PDF)

Text S1 Evaluation of potential false positive results.
(PDF)

Text S2 Comparison of tunnels identified by CAVER 3.0 with known DhaA tunnels.
(PDF)

## References

[1] Damborsky J, Petrek M, Banas P, Otyepka M (2007) Identification of tunnels in proteins, nucleic acids, inorganic materials and molecular ensembles. Biotechnol J 2: 62–67. 17183511 10.1002/biot.200600208

[2] Agre P, Brown D, Nielsen S (1995) Aquaporin water channels: unanswered questions and unresolved controversies. Curr Opin Cell Biol 7: 472–483. 7495566 10.1016/0955-0674(95)80003-4 PMC7135066

[3] Gold VAM, Duong F, Collinson I (2007) Structure and function of the bacterial Sec translocon. Mol Membr Biol 24: 387–394. 17710643 10.1080/09687680701416570

[4] Gouaux E, Mackinnon R (2005) Principles of selective ion transport in channels and pumps. Science 310: 1461–1465. 16322449 10.1126/science.1113666

[5] Jiang Y, Lee A, Chen J, Cadene M, Chait BT, et al (2002) Crystal structure and mechanism of a calcium-gated potassium channel. Nature 417: 515–522. 12037559 10.1038/417515a

[6] MacKinnon R (2003) Potassium channels. FEBS Lett 555: 62–65. 14630320 10.1016/s0014-5793(03)01104-9

[7] Khademi S, O'Connell J 3rd, Remis J, Robles-Colmenares Y, Miercke LJW, et al (2004) Mechanism of ammonia transport by Amt/MEP/Rh: structure of AmtB at 1.35 A. Science 305: 1587–1594. 15361618 10.1126/science.1101952

[8] Doyle DA, Morais Cabral J, Pfuetzner RA, Kuo A, Gulbis JM, et al (1998) The structure of the potassium channel: molecular basis of K+ conduction and selectivity. Science 280: 69–77. 9525859 10.1126/science.280.5360.69

[9] Miyazawa A, Fujiyoshi Y, Unwin N (2003) Structure and gating mechanism of the acetylcholine receptor pore. Nature 423: 949–955. 12827192 10.1038/nature01748

[10] Zhou HX, McCammon JA (2010) The gates of ion channels and enzymes. Trends Biochem Sci 35: 179–185. 19926290 10.1016/j.tibs.2009.10.007 PMC2867094

[11] Barney BM, Yurth MG, Dos Santos PC, Dean DR, Seefeldt LC (2009) A substrate channel in the nitrogenase MoFe protein. J Biol Inorg Chem 14: 1015–1022. 19458968 10.1007/s00775-009-0544-2 PMC2819195

[12] Cojocaru V, Winn PJ, Wade RC (2007) The ins and outs of cytochrome P450s. Biochim Biophys Acta 1770: 390–401. 16920266 10.1016/j.bbagen.2006.07.005

[13] Coulombe R, Yue KQ, Ghisla S, Vrielink A (2001) Oxygen access to the active site of cholesterol oxidase through a narrow channel is gated by an Arg-Glu pair. J Biol Chem 276: 30435–30441. 11397813 10.1074/jbc.M104103200

[14] Ho FM (2008) Uncovering channels in photosystem II by computer modelling: current progress, future prospects, and lessons from analogous systems. Photosyn Res 98: 503–522. 18798008 10.1007/s11120-008-9358-2

[15] Klvana M, Pavlova M, Koudelakova T, Chaloupkova R, Dvorak P, et al (2009) Pathways and mechanisms for product release in the engineered haloalkane dehalogenases explored using classical and random acceleration molecular dynamics simulations. J Mol Biol 392: 1339–1356. 19577578 10.1016/j.jmb.2009.06.076

[16] Moriguchi T, Ida K, Hikima T, Ueno G, Yamamoto M, et al (2010) Channeling and conformational changes in the heterotetrameric sarcosine oxidase from *Corynebacterium* sp. U-96. J Biochem 148: 491–505. 20675294 10.1093/jb/mvq083

[17] Shen T, Tai K, Henchman RH, McCammon JA (2002) Molecular dynamics of acetylcholinesterase. Acc Chem Res 35: 332–340. 12069617 10.1021/ar010025i

[18] Huang X, Holden HM, Raushel FM (2001) Channeling of substrates and intermediates in enzyme-catalyzed reactions. Annu Rev Biochem 70: 149–180. 11395405 10.1146/annurev.biochem.70.1.149

[19] Hyde CC, Ahmed SA, Padlan EA, Miles EW, Davies DR (1988) Three-dimensional structure of the tryptophan synthase alpha 2 beta 2 multienzyme complex from *Salmonella typhimurium* . J Biol Chem 263: 17857–17871. 3053720

[20] Krahn JM, Kim JH, Burns MR, Parry RJ, Zalkin H, et al (1997) Coupled formation of an amidotransferase interdomain ammonia channel and a phosphoribosyltransferase active site. Biochemistry 36: 11061–11068. 9333323 10.1021/bi9714114

[21] Raushel FM, Thoden JB, Holden HM (2003) Enzymes with molecular tunnels. Acc Chem Res 36: 539–548. 12859215 10.1021/ar020047k

[22] Teplyakov A, Obmolova G, Badet B, Badet-Denisot MA (2001) Channeling of ammonia in glucosamine-6-phosphate synthase. J Mol Biol 313: 1093–1102. 11700065 10.1006/jmbi.2001.5094

[23] Zamocky M, Herzog C, Nykyri LM, Koller F (1995) Site-directed mutagenesis of the lower parts of the major substrate channel of yeast catalase A leads to highly increased peroxidatic activity. FEBS Lett 367: 241–245. 7607315 10.1016/0014-5793(95)00568-t

[24] Fishman A, Tao Y, Bentley WE, Wood TK (2004) Protein engineering of toluene 4-monooxygenase of *Pseudomonas mendocina* KR1 for synthesizing 4-nitrocatechol from nitrobenzene. Biotechnol Bioeng 87: 779–790. 15329936 10.1002/bit.20185

[25] Huang X, Raushel FM (2000) An engineered blockage within the ammonia tunnel of carbamoyl phosphate synthetase prevents the use of glutamine as a substrate but not ammonia. Biochemistry 39: 3240–3247. 10727215 10.1021/bi9926173

[26] Hub JS, de Groot BL (2008) Mechanism of selectivity in aquaporins and aquaglyceroporins. Proc Natl Acad Sci USA 105: 1198–1203. 18202181 10.1073/pnas.0707662104 PMC2234115

[27] Chaloupkova R, Sykorova J, Prokop Z, Jesenska A, Monincova M, et al (2003) Modification of activity and specificity of haloalkane dehalogenase from *Sphingomonas paucimobilis* UT26 by engineering of its entrance tunnel. J Biol Chem 278: 52622–52628. 14525993 10.1074/jbc.M306762200

[28] Pavlova M, Klvana M, Prokop Z, Chaloupkova R, Banas P, et al (2009) Redesigning dehalogenase access tunnels as a strategy for degrading an anthropogenic substrate. Nat Chem Biol 5: 727–733. 19701186 10.1038/nchembio.205

[29] Schmitt J, Brocca S, Schmid RD, Pleiss J (2002) Blocking the tunnel: engineering of *Candida rugosa* lipase mutants with short chain length specificity. Protein Eng 15: 595–601. 12200542 10.1093/protein/15.7.595

[30] Wen Z, Baudry J, Berenbaum MR, Schuler MA (2005) Ile115Leu mutation in the SRS1 region of an insect cytochrome P450 (CYP6B1) compromises substrate turnover via changes in a predicted product release channel. Protein Eng Des Sel 18: 191–199. 15837716 10.1093/protein/gzi023

[31] Arroyo-Mañez P, Bikiel DE, Boechi L, Capece L, Di Lella S, et al (2011) Protein dynamics and ligand migration interplay as studied by computer simulation. Biochim Biophys Acta 1814: 1054–1064. 20797453 10.1016/j.bbapap.2010.08.005

[32] Karplus M, McCammon JA (2002) Molecular dynamics simulations of biomolecules. Nat Struct Biol 9: 646–652. 12198485 10.1038/nsb0902-646

[33] Li W, Shen J, Liu G, Tang Y, Hoshino T (2011) Exploring coumarin egress channels in human cytochrome P450 2A6 by random acceleration and steered molecular dynamics simulations. Proteins 79: 271–281. 21058395 10.1002/prot.22880

[34] Otyepka M, Skopalik J, Anzenbacherová E, Anzenbacher P (2007) What common structural features and variations of mammalian P450s are known to date? Biochim Biophys Acta 1770: 376–389. 17069978 10.1016/j.bbagen.2006.09.013

[35] Smart OS, Neduvelil JG, Wang X, Wallace BA, Sansom MS (1996) HOLE: a program for the analysis of the pore dimensions of ion channel structural models. J Mol Graph 14: 354–360. 9195488 10.1016/s0263-7855(97)00009-x

[36] Petrek M, Otyepka M, Banas P, Kosinova P, Koca J, et al (2006) CAVER: a new tool to explore routes from protein clefts, pockets and cavities. BMC Bioinformatics 7: 316. 16792811 10.1186/1471-2105-7-316 PMC1539030

[37] Petrek M, Kosinova P, Koca J, Otyepka M (2007) MOLE: a Voronoi diagram-based explorer of molecular channels, pores, and tunnels. Structure 15: 1357–1363. 17997961 10.1016/j.str.2007.10.007

[38] Medek P, Benes P, Sochor J (2008) Multicriteria tunnel computation. In: CGIM '08 Proceedings of the Tenth IASTED International Conference on Computer Graphics and Imaging; 13–15 February 2008; Innsbruck, Austria. CGIM 2008. Available: http://www.actapress.com/Content_of_Proceeding.aspx?proceedingID=472. Accessed 15 August 2011.

[39] Yaffe E, Fishelovitch D, Wolfson HJ, Halperin D, Nussinov R (2008) MolAxis: efficient and accurate identification of channels in macromolecules. Proteins 73: 72–86. 18393395 10.1002/prot.22052 PMC2693897

[40] Yaffe E, Fishelovitch D, Wolfson HJ, Halperin D, Nussinov R (2008) MolAxis: a server for identification of channels in macromolecules. Nucleic Acids Res 36: W210–215. 18448468 10.1093/nar/gkn223 PMC2447770

[41] Coleman RG, Sharp KA (2009) Finding and characterizing tunnels in macromolecules with application to ion channels and pores. Biophys J 96: 632–645. 18849407 10.1529/biophysj.108.135970 PMC2716472

[42] Ho BK, Gruswitz F (2008) HOLLOW: generating accurate representations of channel and interior surfaces in molecular structures. BMC Struct Biol 8: 49. 19014592 10.1186/1472-6807-8-49 PMC2603037

[43] Pellegrini-Calace M, Maiwald T, Thornton JM (2009) PoreWalker: a novel tool for the identification and characterization of channels in transmembrane proteins from their three-dimensional structure. PLoS Comput Biol 5: e1000440. 19609355 10.1371/journal.pcbi.1000440 PMC2704872

[44] Voss NR, Gerstein M (2010) 3V: cavity, channel and cleft volume calculator and extractor. Nucleic Acids Res 38: W555–562. 20478824 10.1093/nar/gkq395 PMC2896178

[45] Aurenhammer F (1991) Voronoi diagrams: A survey of a fundamental geometric data structure. ACM Comput Surv 23: 345–405.

[46] Kim DS, Cho Y, Kim D (2005) Euclidean Voronoi diagram of 3D balls and its computation via tracing edges. Comput Aided Des 37: 1412–1424.

[47] Barber CB, Dobkin DP, Huhdanpaa H (1996) The Quickhull algorithm for convex hulls. ACM T Math Software 22: 469–483.

[48] Dijkstra EW (1959) A note on two problems in connexion with graphs. Numer Math 1: 269–271.

[49] Benes P, Medek P, Sochor J (2009) Computation of channels in protein dynamics. In: Proceedings of the IADIS International Conference Applied Computing; 19–21 November 2009; Rome, Italy. IADIS 2009. Available: http://www.iadis.net/dl/final_uploads/200917L031.pdf. Accessed 15 August 2011.

[50] Loewenstein Y, Portugaly E, Fromer M, Linial M (2008) Efficient algorithms for accurate hierarchical clustering of huge datasets: tackling the entire protein space. Bioinformatics 24: i41–49. 18586742 10.1093/bioinformatics/btn174 PMC2718652

[51] Bouckaert RR, Frank E, Hall MA, Holmes G, Pfahringer B, et al (2010) WEKA–Experiences with a Java Open-Source Project. J Mach Learn Res 11: 2533–2541.

[52] Bentley JL (1975) Multidimensional binary search trees used for associative searching. Commun ACM 18: 509–517.

[53] Schrödinger LLC (2010) The PyMOL Molecular Graphics System, version 1.4r1. Available: http://www.pymol.org. http://www.pymol.org/Accessed 7 July 2011.

[54] Humphrey W, Dalke A, Schulten K (1996) VMD: visual molecular dynamics. J Mol Graph 14: 33–38. 8744570 10.1016/0263-7855(96)00018-5

[55] Yokota T, Omori T, Kodama T (1987) Purification and properties of haloalkane dehalogenase from *Corynebacterium* sp. strain m15-3. J Bacteriol 169: 4049–4054. 3624201 10.1128/jb.169.9.4049-4054.1987 PMC213707

[56] Sallis PJ, Armfield SJ, Bull AT, Hardman DJ (1990) Isolation and characterization of a haloalkane halidohydrolase from *Rhodococcus erythropolis* Y2. J Gen Microbiol 136: 115–120. 2351952 10.1099/00221287-136-1-115

[57] Kulakova AN, Larkin MJ, Kulakov LA (1997) The plasmid-located haloalkane dehalogenase gene from *Rhodococcus rhodochrous* NCIMB 13064. Microbiology 143: 109–115. 9025284 10.1099/00221287-143-1-109

[58] Janssen DB, Gerritse J, Brackman J, Kalk C, Jager D, et al (1988) Purification and characterization of a bacterial dehalogenase with activity toward halogenated alkanes, alcohols and ethers. Eur J Biochem 171: 67–72. 3338472 10.1111/j.1432-1033.1988.tb13759.x

[59] Otyepka M, Damborsky J (2002) Functionally relevant motions of haloalkane dehalogenases occur in the specificity-modulating cap domains. Protein Sci 11: 1206–1217. 11967377 10.1110/ps3830102 PMC2373552

[60] Lüdemann SK, Lounnas V, Wade RC (2000) How do substrates enter and products exit the buried active site of cytochrome P450cam? 1. Random expulsion molecular dynamics investigation of ligand access channels and mechanisms. J Mol Biol 303: 797–811. 11061976 10.1006/jmbi.2000.4154

[61] Bosma T, Damborsky J, Stucki G, Janssen DB (2002) Biodegradation of 1,2,3-trichloropropane through directed evolution and heterologous expression of a haloalkane dehalogenase gene. Appl Environ Microbiol 68: 3582–3587. 12089046 10.1128/AEM.68.7.3582-3587.2002 PMC126774

[62] Gray KA, Richardson TH, Kretz K, Short JM, Bartnek F, et al (2001) Rapid evolution of reversible denaturation and elevated melting temperature in a microbial haloalkane dehalogenase. Adv Synth Catal 343: 607–617.

[63] Los GV, Encell LP, McDougall MG, Hartzell DD, Karassina N, et al (2008) HaloTag: a novel protein labeling technology for cell imaging and protein analysis. ACS Chem Biol 3: 373–382. 18533659 10.1021/cb800025k

