# Supplementary figures and images for: CAVER 3.0: A Tool for the Analysis of Transport Pathways in Dynamic Protein Structures
Source: PLoS Comput Biol. 2012 Oct 18;8(10):e1002708. doi: 10.1371/journal.pcbi.1002708 (PMC3475669; doi:10.1371/journal.pcbi.1002708)

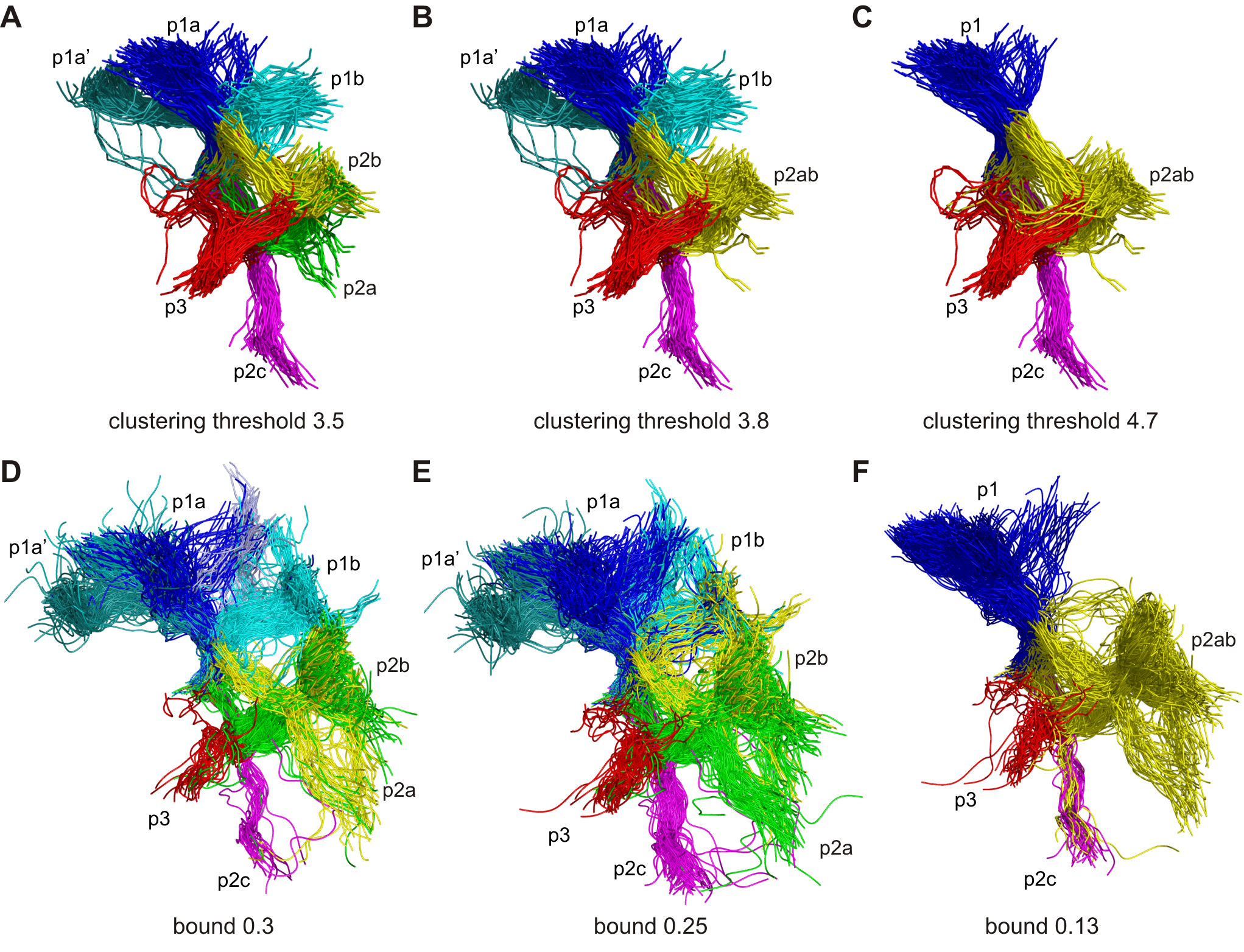

Supplement: Figure S1 — Clusters of pathways calculated in the molecular dynamics simulation of haloalkane dehalogenase DhaA by CAVER 3.0 (A–C) and MOLE 1.2 (D–F). Pathways identified throughout the simulation are shown in one frame as pathway centerlines; if more pathways from the same snapshot are grouped to the same cluster, only the pathway with the lowest cost is shown. The variants of the p1 (main) tunnel are in different shades of blue, variants of the p2 tunnel in yellow, green and magenta and the p3 tunnel is in red. (A–C) CAVER 3.0 results for different settings of the clustering threshold. (A) Clustering was performed with constant weights along the entire pathway and low clustering threshold of 3.5. The p1 pathways with dispersed openings as well as the p2a and p2b pathways which have a common opening are separated into different clusters. (B) Increasing the clustering threshold led to the joining of the p2a and p2b pathway clusters. (C) Further increase of the clustering threshold led to the grouping of all the p1 pathways into a single cluster. Note that some of the previously visible p1 pathways are not visible after the change of threshold since in individual snapshots, only the pathway with the lowest cost is shown for each cluster. (D–F) MOLE 1.2 results for different settings of the clustering parameters. (D) The parameters were set to distinguish the known variants of the p2 tunnel; the p2a and p2b pathway clusters are not well defined as they largely overlap along the entire tunnel length. The p1 tunnel was divided into multiple clusters. (E) Recalculation with a lower value of the bound parameter led to the grouping of a portion of the p1 pathways into one cluster, while other p1 pathways remained separated. The p2a and p2b clusters are not well defined—part of the p2b cluster overlaps with the p2a cluster and part with the p1b cluster. (F) The bound parameter was optimized to join all the p1 pathways into a single cluster. This led to also the p2a and p2b pathways be [file pcbi.1002708.s001.tif]

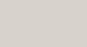

Supplement: Software S1 — CAVER 3.0 package containing CAVER 3.0 executable, source code, license, documentation and examples. The latest release of CAVER 3.0 can be downloaded from http://www.caver.cz. (ZIP) [file pcbi.1002708.s006.zip › caver_3.0/caver/bin/bottleneck_unknown.png]

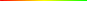

Supplement: Software S1 — CAVER 3.0 package containing CAVER 3.0 executable, source code, license, documentation and examples. The latest release of CAVER 3.0 can be downloaded from http://www.caver.cz. (ZIP) [file pcbi.1002708.s006.zip › caver_3.0/caver/bin/pallette.png]
