# Supplementary material for: CAVER 3.0: A Tool for the Analysis of Transport Pathways in Dynamic Protein Structures
Source: PLoS Comput Biol. 2012 Oct 18;8(10):e1002708. doi: 10.1371/journal.pcbi.1002708 (PMC3475669; doi:10.1371/journal.pcbi.1002708)
Supplement: Protocol S1 — Comparison of CAVER 3.0, MOLE 1.2 and MolAxis 1.4. (PDF) [file pcbi.1002708.s002.pdf]

Protocol S1: Comparison of CAVER 3.0, MOLE 1.2 and MolAxis 1.4

The crystal structures were prepared for the comparison by removing all ligands with the exception of HEM. Atoms with alternate conformations were retained in the conformation with higher occupancy. Analyses by CAVER 3.0 were performed using the default settings except for the frame clustering threshold of 2 and the probe radius of 0.6 Å. The starting points, shell radius, the number of approximating balls and the application of the average surface were adjusted for each protein in the test set as follows:

PDB-ID	Starting point (x, y, z coordinates)			Shell radius [Å]	No. of approximating spheres	Average surface
2OAR	62.150	160.98	-0.050	15	12	no
2BG9	61.170	63.440	23.860	15	4	no
1BL8	82.530	26.552	46.581	5	12	no
1AKD	17.012	24.139	7.790	3	12	yes
2ACE	4.397	68.330	63.294	5	12	yes
1MXT	17.083	-0.533	12.495	3	12	yes
1MQF	56.786	17.650	16.101	5	12	yes
1THG	51.745	4.591	7.890	3	12	yes
2JBV	84.060	35.959	74.901	5	12	yes

MOLE 1.2 [1] was set to calculate 50 tunnels for each protein in the test set. The calculation was performed without clustering in order to retrieve all tunnels, including those of high similarity. The starting points were set as follows:

PDB	Starting point (x, y, z coordinates)		
2OAR	62.150	160.980	-0.050
2BG9	61.170	63.440	23.860
1BL8	82.530	26.552	46..581
1AKD	17.012	24.139	7.790
2ACE	4.397	68.330	63.294
1MXT	17.083	-0.533	12.495
1MQF	56.786	17.650	16.101
1THG	51.745	4.591	7.890
2JBV	84.060	35.959	74.901

Analyses by MolAxis 1.4 [2,3] were performed using approximation resolution of 0.1 Å. For chamber proteins, the calculations were started from a point within 3 Å of the user-specified starting point. For transmembrane proteins (2BG9, 1BL8 and 2OAR), the search for the channel was confined to a "via sphere" of 3 Å radius centered at the user-specified point along the channel vector. The radius of the outer sphere and the display cutoff were adjusted in individual cases.

PDB	Starting point (x, y, z coordinates)			Outer sphere radius [Å]	Display radius cutoff [Å]	Channel vector
2OAR	27.654[a]	129.514[a]	1.14[a]	50	11	1, 1, 1
2BG9	61.170[a]	63.440[a]	23.860[a]	140	12	0, 0, 1
1BL8	82.530[a]	26.552[a]	46.581[a]	70	5	0, 0, 1
1AKD	17.012	24.139	7.790	30	3.6	-
2ACE	4.397	68.330	63.294	30	3.5	-
1MXT	17.083	-0.533	12.495	30	4	-
1MQF	56.786	17.650	16.101	30	4	-
1THG	51.745	4.591	7.890	30	4	-
2JBV	84.060	35.959	74.901	30	3	-

[a]center of the "via sphere"

References

1. Petrek M, Kosinova P, Koca J, Otyepka M (2007) MOLE: a Voronoi diagram-based explorer of molecular channels, pores, and tunnels. Structure 15: 1357–1363.
2. Yaffe E, Fishelovitch D, Wolfson HJ, Halperin D, Nussinov R (2008) MolAxis: efficient and accurate identification of channels in macromolecules. Proteins 73: 72–86.
3. Yaffe E, Fishelovitch D, Wolfson HJ, Halperin D, Nussinov R (2008) MolAxis: a server for identification of channels in macromolecules. Nucleic Acids Res 36: W210–215.
