# Supplementary material for: CAVER 3.0: A Tool for the Analysis of Transport Pathways in Dynamic Protein Structures
Source: PLoS Comput Biol. 2012 Oct 18;8(10):e1002708. doi: 10.1371/journal.pcbi.1002708 (PMC3475669; doi:10.1371/journal.pcbi.1002708)
Supplement: Protocol S2 — Molecular dynamics simulation of haloalkane dehalogenase DhaA. (PDF) [file pcbi.1002708.s003.pdf]

Protocol S2: Molecular dynamics simulation of haloalkane dehalogenase DhaA

Preparation of protein system

The crystal structure of DhaA (PDB-ID 1CQW) [1] was obtained from the RCSB PDB database [2]. The substitutions V172A, I209L and G292A were modeled into the crystal structure of DhaA to ensure correspondence with the wild type enzyme used in a detailed study of DhaA transport pathways [3]. The substitutions were modeled in PyMOL 1.4 [4] and hydrogen atoms were added by WHAT IF 6.0 [5]. Prepared systems were neutralized by the addition of 18 Na^+ ions and the box of TIP3P water molecules [6] was added to a distance of 10 Å from any solute atom using the Tleap module of AMBER 8 [7].

Molecular dynamics

All calculations were carried out in the Sander module of AMBER 8 using the ff99SB force field [8]. Investigated systems were energetically minimized by 300 steps of the steepest descent (SD) method, with the heavy atoms of the protein fixed. The minimization was followed by 20 ps of molecular dynamics of counter ions and all water molecules. The whole system was minimized in four rounds of 300 SD steps with decreasing restraint on the protein backbone (500, 125, 25 and 0 $kcal.mol^{-1}.Å^{-2}$). A molecular dynamics simulation with 2 fs time step was applied to the whole system using constant pressure periodic boundary conditions. During the initial 200 ps of simulation, the system was slowly heated from 0 to 300 K and the constant temperature was maintained by the weak coupling algorithm [9] during all 10 ns of the total simulation time. The particle mesh Ewald method was employed for treatment of the electrostatic interactions [10] and SHAKE algorithm was employed to fix all bonds containing hydrogen atoms [11]. A 10 Å cut-off was applied to non-bonded interactions. The coordinates were saved in 0.5 ps intervals. Obtained trajectories were analyzed using Ptraj module of AMBER and the visualization was performed with PyMOL 1.4 and VMD 1.8.6 [12] programs.

References

1. Newman J, Peat TS, Richard R, Kan L, Swanson PE, et al. (1999) Haloalkane dehalogenases: structure of a *Rhodococcus* enzyme. Biochemistry 38: 16105–16114.
2. Rose PW, Beran B, Bi C, Bluhm WF, Dimitropoulos D, et al. (2011) The RCSB Protein Data Bank: redesigned web site and web services. Nucleic Acids Res 39: D392–401.
3. Klvana M, Pavlova M, Koudelakova T, Chaloupkova R, Dvorak P, et al. (2009) Pathways and mechanisms for product release in the engineered haloalkane dehalogenases explored using classical and random acceleration molecular dynamics simulations. J Mol Biol 392: 1339–1356.
4. Schrödinger LLC (2010) The PyMOL Molecular Graphics System, Version 1.4r1.
5. Vriend G (1990) WHAT IF: a molecular modeling and drug design program. J Mol Graph 8: 52–56.
6. Jorgensen WL, Chandrasekhar J, Madura JD, Impey RW, Klein ML (1983) Comparison of simple potential of functions for simulating liquid water. J Chem Phys 79: 926–935.
7. Pearlman D (1995) AMBER, a package of computer programs for applying molecular mechanics, normal mode analysis, molecular dynamics and free energy calculations to simulate the structural and energetic properties of molecules. Comput Phys Commun 91: 1–41.
8. Hornak V, Abel R, Okur A, Strockbine B, Roitberg A, et al. (2006) Comparison of multiple Amber force fields and development of improved protein backbone parameters. Proteins 65: 712–725.
9. Berendsen HJC, Postma JPM, Gunsteren WFV, Dinola A, Haak JR (1984) Molecular dynamics with coupling to an external bath. J Chem Phys 81: 3684–3690.
10. Darden T, York D, Pedersen L (1993) Particle mesh Ewald: An *N*.log(*N*) method for Ewald sums in large systems. J Chem Phys 98: 10089–10092.
11. Ryckaert J, Ciccotti G, Berendsen H (1977) Numerical integration of the cartesian equations of motion of

a system with constraints: molecular dynamics of n-alkanes. J Comput Phys 23: 327–341.
12. Humphrey W, Dalke A, Schulten K (1996) VMD: visual molecular dynamics. J Mol Graph 14: 33–38.
