# Supplementary material for: CAVER 3.0: A Tool for the Analysis of Transport Pathways in Dynamic Protein Structures
Source: PLoS Comput Biol. 2012 Oct 18;8(10):e1002708. doi: 10.1371/journal.pcbi.1002708 (PMC3475669; doi:10.1371/journal.pcbi.1002708)
Supplement: Protocol S3 — Analysis of molecular dynamics simulation of DhaA. (PDF) [file pcbi.1002708.s004.pdf]

Protocol S3: Analysis of molecular dynamics simulation of DhaA

Comparison of CAVER 3.0 and MOLE 1.2

The snapshots sampled every 10 ps from 10 ns molecular dynamics simulation of DhaA were used as input structures for CAVER 3.0 and MOLE 1.2 [1]. Both calculations were performed using the same set of atomic radii and the same initial starting point. CAVER 3.0 calculations were conducted using the shell probe of 3 Å and the depth of 4 Å to define the protein surface. Each atom in the structure was approximated by 12 spheres. The tunnel search was performed using the probe radius of 0.75 Å. Redundant tunnels in each frame were removed using the default settings. The non-redundant set of identified tunnels was clustered using the hierarchical average-link clustering algorithm based on the pairwise distances of tunnels computed with the weighting coefficient of 1.0. Results were adjusted without re-clustering by varying the clustering threshold parameter. The clustering threshold of 4.7 was used for the comparison with MOLE 1.2 (Figure S1C). MOLE 1.2 was set to calculate 10 tunnels in each snapshot. For each clustering setting, a separate calculation was performed using the sampling parameter of 1.0 and the bound of 0.10, 0.11, 0.13, 0.14, 0.15, 0.20, 0.25, 0.30, 0.35 and 0.40. For comparison with CAVER 3.0, the bound of 0.13 was used (Figure S1F).

Case study

The snapshots sampled every 1 ps from 10 ns molecular dynamics simulation of DhaA were analyzed by CAVER 3.0. The initial starting point was placed at the center of gravity of ND2 atom of Asn41, OD1 atom of Asp106 and CB atom of Pro206 (residue numbering according to DhaA sequence; UniProt accession number P59336). The tunnels were identified using the probe radius of 0.9 Å; otherwise the settings as described above were used. The tunnels were clustered according to the pairwise distances of tunnels calculated using the weighting coefficient of 1.0 and two different clustering thresholds: (i) the clustering threshold of 3.5 and (ii) the clustering threshold of 4.3. The results were analyzed in PyMOL 1.4 [2].

References

1. Petrek M, Kosinova P, Koca J, Otyepka M (2007) MOLE: a Voronoi diagram-based explorer of molecular channels, pores, and tunnels. Structure 15: 1357–1363.
2. Schrödinger LLC (2010) The PyMOL Molecular Graphics System, Version 1.4r1.
