# Supplementary material for: CAVER 3.0: A Tool for the Analysis of Transport Pathways in Dynamic Protein Structures
Source: PLoS Comput Biol. 2012 Oct 18;8(10):e1002708. doi: 10.1371/journal.pcbi.1002708 (PMC3475669; doi:10.1371/journal.pcbi.1002708)
Supplement: Protocol S4 — Analysis of crystal structures of DhaA. (PDF) [file pcbi.1002708.s005.pdf]

Protocol S4: Analysis of crystal structures of DhaA

The crystal structures of DhaA (PDB-IDs 1CQW, 1BN7 and 1BN6) [1] were obtained from the RCSB PDB database [2]. Substitutions V172A, I209L and G292A were modeled into the crystal structures of DhaA to ensure correspondence with the wild type enzyme used in the detailed study of DhaA transport pathways [3] and the molecular dynamics simulations described in Protocol S2 and Protocol S3. Mutations were modeled using PyMOL 1.4 [4]. This software was also used for removing alternate conformations of residues, water molecules and ligands and adding hydrogen atoms to all three structures. Prepared structures both with and without hydrogen atoms were analyzed by CAVER 3.0 using the probe radius of 0.9 Å and 0.8 Å and the same settings as for the analysis of molecular dynamics simulations (Protocol S3). The non-redundant set of identified tunnels was clustered based on the pairwise distances of tunnels calculated using the weighting coefficient of 1.0 and the clustering threshold of 4.3.

References

1. Newman J, Peat TS, Richard R, Kan L, Swanson PE, et al. (1999) Haloalkane dehalogenases: structure of a *Rhodococcus* enzyme. Biochemistry 38: 16105–16114.
2. Rose PW, Beran B, Bi C, Bluhm WF, Dimitropoulos D, et al. (2011) The RCSB Protein Data Bank: redesigned web site and web services. Nucleic Acids Res 39: D392–401.
3. Klvana M, Pavlova M, Koudelakova T, Chaloupkova R, Dvorak P, et al. (2009) Pathways and mechanisms for product release in the engineered haloalkane dehalogenases explored using classical and random acceleration molecular dynamics simulations. J Mol Biol 392: 1339–1356.
4. Schrödinger LLC (2010) The PyMOL Molecular Graphics System, Version 1.4r1.
