# Supplementary material for: CAVER 3.0: A Tool for the Analysis of Transport Pathways in Dynamic Protein Structures
Source: PLoS Comput Biol. 2012 Oct 18;8(10):e1002708. doi: 10.1371/journal.pcbi.1002708 (PMC3475669; doi:10.1371/journal.pcbi.1002708)
Supplement: Table S1 — Comparison of pathways calculated by CAVER 3.0, MOLE 1.2 and MolAxis 1.4. (PDF) [file pcbi.1002708.s007.pdf]

Table S1 Comparison of pathways calculated by CAVER 3.0, MOLE 1.2 and MolAxis 1.4

Mechanosensitive channel MscL (2OAR)

CAVER 3.0			MOLE 1.2			MolAxis 1.4		
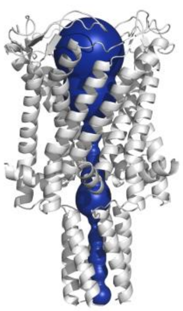			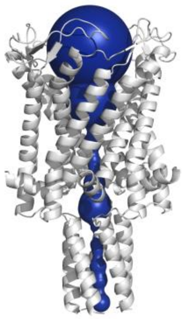			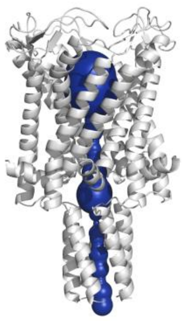		
Pathway	Bottleneck radius [Å]	Rank	Pathway	Bottleneck radius [Å]	Rank	Pathway	Bottleneck radius [Å]	Rank
Blue	1.0	18	Blue	0.1	32	Blue	0.9	1

Nicotinic acetylcholine receptor (2BG9)

CAVER 3.0			MOLE 1.2			MolAxis 1.4		
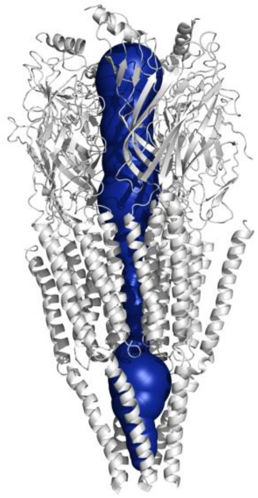			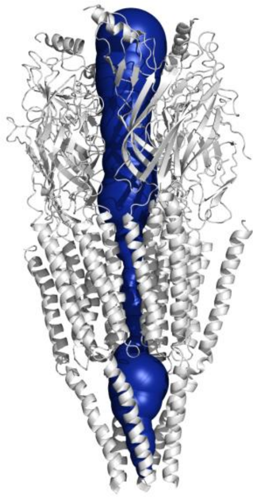			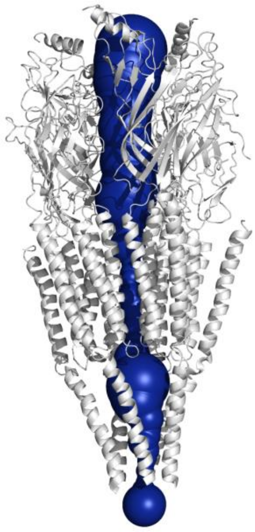		
Pathway	Bottleneck radius [Å]	Rank	Pathway	Bottleneck radius [Å]	Rank	Pathway	Bottleneck radius [Å]	Rank
Blue	2.7	28	Blue	2.4	18	Blue	2.2	1

Potassium channel KcsA (1BL8)

CAVER 3.0			MOLE 1.2			MolAxis 1.4		
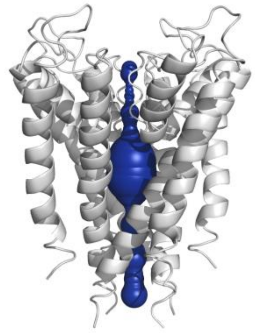			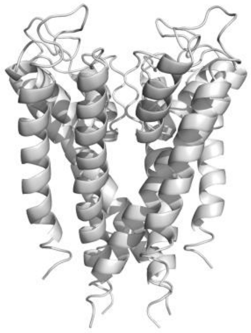			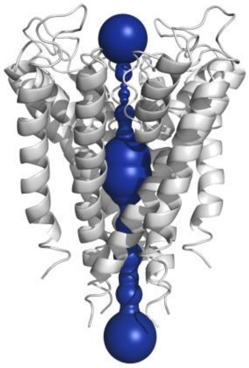		
Pathway	Bottleneck radius [Å]	Rank	Pathway	Bottleneck radius [Å]	Rank	Pathway	Bottleneck radius [Å]	Rank
Blue	0.7	21	Blue	- [a]	- [a]	Blue	0.7	1

Cytochrome P450 CAM (1AKD)

CAVER 3.0			MOLE 1.2			MolAxis 1.4		
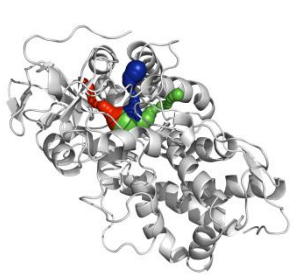			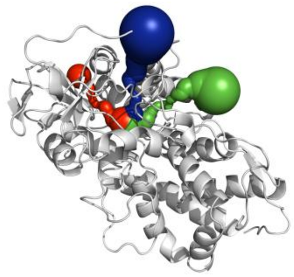			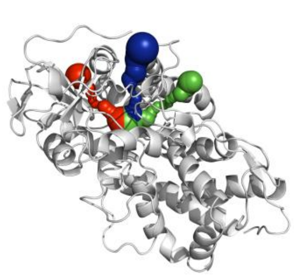		
Pathway	Bottleneck radius [Å]	Rank	Pathway	Bottleneck radius [Å]	Rank	Pathway	Bottleneck radius [Å]	Rank
Blue	0.7	1	Blue	0.6	1	Blue	0.7	2
Green	0.8	2	Green	0.6	2	Green	0.7	8
Red	0.8	3	Red	0.8	4	Red	0.9	1

Acetylcholinesterase (2ACE)

CAVER 3.0			MOLE 1.2			MolAxis 1.4		
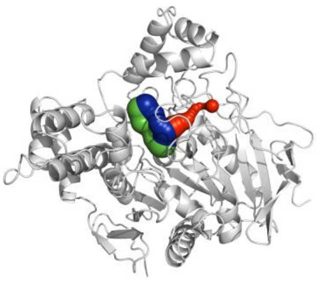			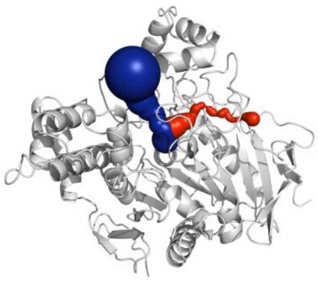			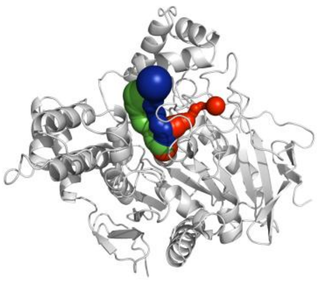		
Pathway	Bottleneck radius [Å]	Rank	Pathway	Bottleneck radius [Å]	Rank	Pathway	Bottleneck radius [Å]	Rank
Blue	2.0	1	Blue	1.9	1	Blue	1.5	1
Green	1.7	2	Green	- [a]	- [a]	Green	1.7	2
Red	0.6	3	Red	0.2	73	Red	0.7	5

Cholesterol oxidase (1MXT)

CAVER 3.0			MOLE 1.2			MolAxis 1.4		
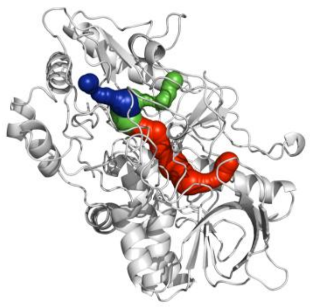			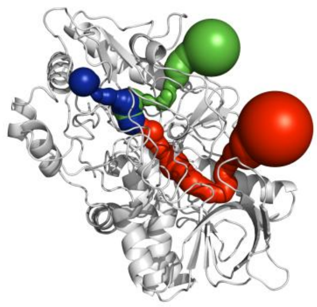			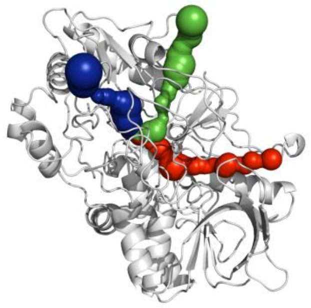		
Pathway	Bottleneck radius [Å]	Rank	Pathway	Bottleneck radius [Å]	Rank	Pathway	Bottleneck radius [Å]	Rank
Blue	1.1	1	Blue	0.9	1	Blue	1.2	1
Green	1.0	2	Green	0.7	3	Green	1.0	2
Red	1.6	3	Red	1.7	2	Red	1.3	3

Catalase (1MQF)								
**CAVER 3.0**			**MOLE 1.2**			**MolAxis 1.4**		
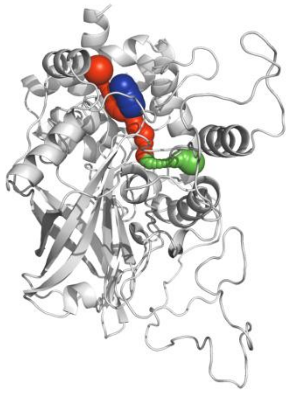			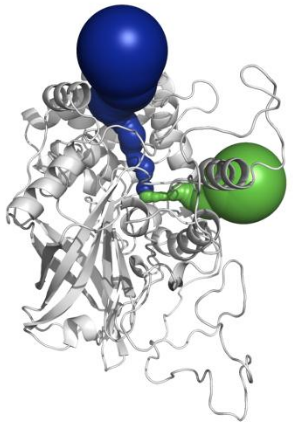			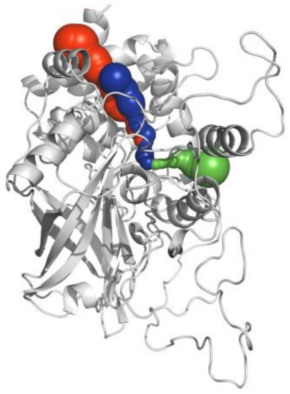		
Pathway	Bottleneck radius [Å]	Rank	Pathway	Bottleneck radius [Å]	Rank	Pathway	Bottleneck radius [Å]	Rank
Blue	1.3	1	Blue	1.0	1	Blue	1.3	1
Green	0.9	2	Green	0.7	3	Green	0.9	2
Red	1.2	3	Red	- [a]	- [a]	Red	1.2	4

Lipase (1THG)								
**CAVER 3.0**			**MOLE 1.2**			**MolAxis 1.4**		
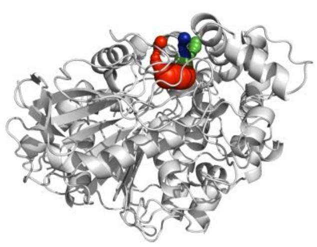			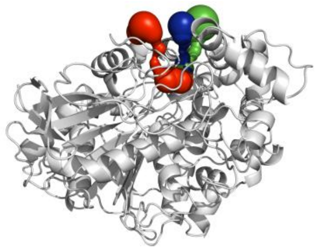			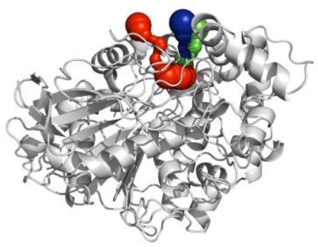		
Pathway	Bottleneck radius [Å]	Rank	Pathway	Bottleneck radius [Å]	Rank	Pathway	Bottleneck radius [Å]	Rank
Blue	0.9	1	Blue	0.8	1	Blue	0.9	2
Green	1.0	2	Green	0.8	2	Green	1.0	1
Red	0.8	3	Red	0.6	5	Red	0.8	4

Choline oxidase (2JBV)								
**CAVER 3.0**			**MOLE 1.2**			**MolAxis 1.4**		
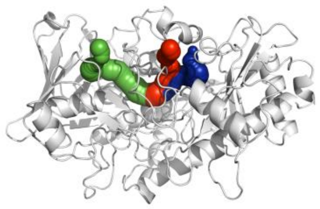			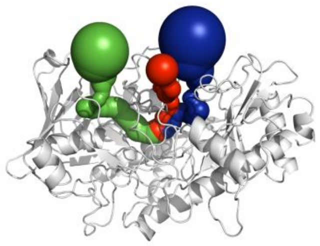			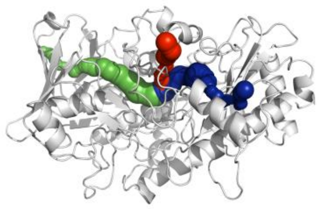		
Pathway	Bottleneck radius [Å]	Rank	Pathway	Radius [Å]	Rank	Pathway	Bottleneck radius [Å]	Rank
Blue	1.1	1	Blue	0.7	1	Blue	0.9	5
Green	1.2	2	Green	1.2	2	Green	0.9	4
Red	0.6	3	Red	0.8	4	Red	0.6	3

[a]equivalent pathway was not found even among 300 calculated tunnels
