# Supplementary material for: CAVER 3.0: A Tool for the Analysis of Transport Pathways in Dynamic Protein Structures
Source: PLoS Comput Biol. 2012 Oct 18;8(10):e1002708. doi: 10.1371/journal.pcbi.1002708 (PMC3475669; doi:10.1371/journal.pcbi.1002708)
Supplement: Table S2 — Characteristics of the pathways identified in 10,000 snapshots of the 10 ns molecular dynamics trajectory of DhaA using the probe radius of 0.9 Å and the clustering threshold of 4.3. (PDF) [file pcbi.1002708.s008.pdf]

Table S2 Characteristics of the pathways identified in 10,000 snapshots of the 10 ns molecular dynamics trajectory of DhaA using the probe radius of 0.9 Å and the clustering threshold of 4.3

Rank	Pathway cluster	No. of snapshots[a]	No. of snapshots with an open pathway[b]	Average bottleneck radius[c] [Å]	Maximum bottleneck radius[Å]	Average throughput[c]
1	p1	9960	5291	1.4	2.3	0.621
2	p2ab	6330	85	1.0	1.8	0.460
3	p2c	120	0	1.0	1.2	0.343
4	p3	131	0	0.9	1.2	0.304
5	-	23	0	0.9	1.0	0.342
6	-	28	0	0.9	1.0	0.237
7	-	25	0	0.9	1.0	0.259
8	-	7	0	0.9	1.0	0.222
9	-	3	0	0.9	1.0	0.169
10	-	1	0	0.9	0.9	0.127
11	-	1	0	0.9	0.9	0.125
12	-	2	0	0.9	0.9	0.056
13	-	1	0	0.9	0.9	0.090
14	-	1	0	0.9	0.9	0.081
15	-	1	0	0.9	0.9	0.042

[a]number of snapshots in which a given pathway was identified (bottleneck radius ≥ 0.9 Å); [b]number of snapshots in which a given pathway was identified and was open (bottleneck radius ≥ 1.4 Å); [c]mean over snapshots in which the given pathway cluster has at least one pathway.
