# Supplementary material for: CAVER 3.0: A Tool for the Analysis of Transport Pathways in Dynamic Protein Structures
Source: PLoS Comput Biol. 2012 Oct 18;8(10):e1002708. doi: 10.1371/journal.pcbi.1002708 (PMC3475669; doi:10.1371/journal.pcbi.1002708)
Supplement: Table S3 — Characteristics of the pathways identified in DhaA crystal structures using the probe radius of 0.8 Å. (PDF) [file pcbi.1002708.s009.pdf]

Table S3 Characteristics of the pathways identified in DhaA crystal structures using the probe radius of 0.8 Å

	PDB-ID 1BN6[a]			PDB-ID 1BN7[a]			PDB-ID 1CQW[a]		
**Rank**	**Pathway**	**Bottleneck radius [Å]**	**Average Throughput**	**Pathway**	**Bottleneck radius [Å]**	**Average Throughput**	**Pathway**	**Bottleneck radius [Å]**	**Average Throughput**
1	p1	1.5	0.713	p1a	1.2	0.722	p1a	1.6	0.709
2	p2a	1.4	0.607	p2ab	0.9	0.500	p2ab	1.0	0.492
3	p2b	1.1	0.602	p1b	0.8	0.422	p1a'	0.9	0.422
4	-	0.9	0.301	-	0.8	0.325	-	0.9	0.272
5	-	0.8	0.221	-	0.9	0.265	p3	0.8	0.241
6	-	0.8	0.125	p3	0.9	0.236	-	0.8	0.099
7	-	0.9	0.100	-	0.8	0.216	-	0.9	0.083
8	-	0.9	0.090	-	0.8	0.097	-	0.8	0.032
9	-	0.8	0.042	-	0.8	0.091	-	0.8	0.028
10	-	0.8	0.038	-	0.8	0.085	-	0.8	0.018
11	-	0.8	0.030	-	0.8	0.051	-	0.8	0.005
12	-	0.8	0.026	-	0.8	0.036	n.a.[b]	-	-
13	-	0.8	0.004	-	0.8	0.034	n.a.[b]	-	-
14	-	0.8	0.0004	n.a.[b]	-	-	n.a.[b]	-	-

[a]DhaA crystal structures with introduced substitutions V172A, I209L and G292A (see Protocol S4), without hydrogen atoms; [b]pathway not identified (n.a. - not applicable).
