# Supplementary material for: CAVER 3.0: A Tool for the Analysis of Transport Pathways in Dynamic Protein Structures
Source: PLoS Comput Biol. 2012 Oct 18;8(10):e1002708. doi: 10.1371/journal.pcbi.1002708 (PMC3475669; doi:10.1371/journal.pcbi.1002708)
Supplement: Table S4 — Comparison of characteristics of the DhaA p1 tunnel obtained by the analysis of the molecular dynamics trajectory and crystal structures. (PDF) [file pcbi.1002708.s010.pdf]

Table S4 Comparison of characteristics of the DhaA p1 tunnel obtained by the analysis of the molecular dynamics trajectory and crystal structures

	**Molecular dynamics trajectory**	**PDB-ID 1CQW[a]**	**PDB-ID 1BN7[a]**	**PDB-ID 1BN6[a]**
**Mean bottleneck radius[Å]**	1.4	1.3	1.2	1.2
**Maximum bottleneck radius [Å]**	2.3	1.3	1.2	1.2
**Bottleneck residues[b]**	F149 (71 %), C176 (59 %), A172 (50 %), A145 (38 %), K175 (26 %), T148 (23 %)	A145, F149, C176	A145, F149, C176	A145, F149, C176

[a]crystal structures with introduced substitutions V172A, I209L and G292A and added hydrogen atoms (see Protocol S4); [b]three residues closest to the tunnel bottleneck; for molecular dynamics, only the most frequent bottleneck residues are listed, together with the percentage of snapshots in which they formed the bottleneck of the p1 tunnel.
