# Supplementary material for: CAVER 3.0: A Tool for the Analysis of Transport Pathways in Dynamic Protein Structures
Source: PLoS Comput Biol. 2012 Oct 18;8(10):e1002708. doi: 10.1371/journal.pcbi.1002708 (PMC3475669; doi:10.1371/journal.pcbi.1002708)
Supplement: Table S5 — Bottleneck residues of the top ranked tunnels of DhaA identified by CAVER 3.0 in molecular dynamics trajectory using the probe radius of 0.9 Å and the clustering threshold of 3.5. (PDF) [file pcbi.1002708.s011.pdf]

Table S5 Bottleneck residues of the top ranked tunnels of DhaA identified by CAVER 3.0 in molecular dynamics trajectory using the probe radius of 0.9 Å and the clustering threshold of 3.5.

Pathway	p1a	p1a´	p1b	p2a	p2b	p2c	p3
**Bottleneck residues**[a]	F149 (71 %), C176 (59 %), A172 (50 %), A145 (38 %), K175 (27 %), T148 (23 %)	A172 (91 %), F152 (90%), F168 (82 %), A167 (20 %)	V245 (98%), F144 (85%), P142 (79 %), A145 (30 %)	L246 (93 %), L209 (57 %), W141 (54 %), I135 (36 %), I132 (34 %)	W141 (82 %), V245 (78 %), L246 (69 %), P142 (59 %)	P210 (73 %), I135 (47 %), R133 (41 %), P134 (37 %),	I211 (72 %), I135 (56 %), W138 (56 %), W141 (41 %), T137 (20 %)

[a]three residues closest to the pathway bottleneck were considered in each snapshot; only the most frequent bottleneck residues are listed together with the percentage of snapshots in which they formed the bottleneck.
