# Supplementary material for: CAVER 3.0: A Tool for the Analysis of Transport Pathways in Dynamic Protein Structures
Source: PLoS Comput Biol. 2012 Oct 18;8(10):e1002708. doi: 10.1371/journal.pcbi.1002708 (PMC3475669; doi:10.1371/journal.pcbi.1002708)
Supplement: Text S1 — Evaluation of potential false positive results. (PDF) [file pcbi.1002708.s012.pdf]

Text S1: Evaluation of potential false positive results

Due to approximated VDW molecular surface, it may happen that some pathways are identified even though the real radius of some pathway segment is slightly smaller than the user-specified probe radius r_B. Such pathways will be referred to as false positives. This can happen only if the real pathway radius is similar to r_B. Therefore, the number of false positives in the analyzed dataset will depend on the number of pathways with the bottleneck radius, i.e., the radius of the narrowest pathway segment, similar to r_B.

The number of potential false positives within the 16,634 pathways identified in 10,000 snapshots of the 10 ns molecular dynamics trajectory of haloalkane dehalogenase DhaA using the probe radius of 0.9 Å and the clustering threshold of 4.3 was calculated as follows. The pathway was assigned as potential false positive if for at least one of the pathway segments $r_{det} - e_{max} < 0.9$, where r_{det} is the radius of a given pathway segment as determined by CAVER 3.0 and e_{max} is the maximal possible error in determination of r_{det} of this pathway segment.

Altogether, 1,981 pathways (~ 12 %) were assigned as potential false positives. All potential false positives were represented by the pathways with bottleneck radii very similar to r_B — 100% of potential false positives had the bottleneck radius ≤ 1.02 Å, 85 % had the bottleneck radius within the range of 0.90 Å–0.95 Å. It is important to note that the calculation of potential false positives involved a maximum possible overestimation error, i.e., obtained numbers represent the upper bound of possible false positives.
