# Supplementary material for: CAVER 3.0: A Tool for the Analysis of Transport Pathways in Dynamic Protein Structures
Source: PLoS Comput Biol. 2012 Oct 18;8(10):e1002708. doi: 10.1371/journal.pcbi.1002708 (PMC3475669; doi:10.1371/journal.pcbi.1002708)
Supplement: Text S2 — Comparison of tunnels identified by CAVER 3.0 with known DhaA tunnels. (PDF) [file pcbi.1002708.s013.pdf]

Text S2: Comparison of tunnels identified by CAVER 3.0 with known DhaA tunnels

All five DhaA access tunnels previously proposed by classical molecular dynamics (MD) and Random Acceleration Molecular Dynamics (RAMD) simulations [1] were reliably identified by CAVER 3.0 (Table S2). The p1 tunnel was the most frequently observed pathway in the analyzed MD simulation, had by far the highest maximum and mean bottleneck radii and was frequently open for water molecules (bottleneck radius >= 1.4 A). The importance of the p1 tunnel suggested by CAVER 3.0 is in correspondence with previous MD and RAMD simulations [1], where the p1 tunnel was found to be the dominant transport pathway for both product release and the exchange of water molecules between the active site cavity and bulk solvent. The p1 tunnel is also clearly visible in DhaA crystal structures.

The second best ranked tunnel identified by CAVER 3.0 corresponds to the p2a and p2b pathways of Klvana *et al.* [1]. The p2ab pathway was identified in 63 % of snapshots (Table S2). In approximately 75 % of cases, the p2b pathway was geometrically preferred over the p2a pathway. The p2b tunnel was found to be open for water molecules during the simulation, although only in a few snapshots (9 %). It also had the second highest maximum bottleneck radius. These findings are in good correspondence with the previous MD analyses, where the p2b tunnel was preferred for the exchange of water molecules over the p2a, p2c, and p3 tunnels. The previous study also showed that the accessibility of the p2b tunnel for water is low in the absence of a chloride ion in the active site [1], possibly explaining a relatively low frequency of open states of the p2b tunnel in our simulation. The p2a tunnel, previously referred to as the "slot" [2] was open for water molecules in only 3 snapshots. In previous MD and RAMD simulations, the p2a tunnel was rarely used by both water molecules and 2,3-dichloropropane-1-ol [1]. We note that the definition of p2b and p2a tunnels used in our study may slightly differ from Klvana *et al.* [1], where these pathways were found to run roughly parallel to each other. In our analysis, we observed that these two tunnels may cross each other, and consequently four different pathways may be identified by the geometrical analysis.

The other two remaining tunnels, p2c and p3 (ranked third and fourth), were identified in only a small portion of snapshots (12 % and 13 %) and did not open for waters during the simulation (Table S2). However, compared to the other possible tunnels ranked at lower places (none of which was identified in more than 3 % of snapshots), the p2c and p3 still demonstrated considerable widening of the bottleneck (up to 1.2 Å) in some snapshots and it is likely that these two tunnels would open if the simulation was longer or ions were present in the active site. In previous MD and RAMD simulations, the p2c tunnel was used for the exchange of water molecules only in the presence of chloride ion in the active site and p3 tunnel was not used by waters at all, but was once used for the release of 2,3-dichloropropane-1-ol [1].

References

1. Klvana M, Pavlova M, Koudelakova T, Chaloupkova R, Dvorak P, et al. (2009) Pathways and mechanisms for product release in the engineered haloalkane dehalogenases explored using classical and random acceleration molecular dynamics simulations. J Mol Biol 392: 1339–1356.
2. Otyepka M, Damborský J (2002) Functionally relevant motions of haloalkane dehalogenases occur in the specificity-modulating cap domains. Protein Sci 11: 1206–1217.
