# Supplementary material for: CAVER 3.0: A Tool for the Analysis of Transport Pathways in Dynamic Protein Structures
Source: PLoS Comput Biol. 2012 Oct 18;8(10):e1002708. doi: 10.1371/journal.pcbi.1002708 (PMC3475669; doi:10.1371/journal.pcbi.1002708)
Supplement: Software S1 — CAVER 3.0 package containing CAVER 3.0 executable, source code, license, documentation and examples. The latest release of CAVER 3.0 can be downloaded from http://www.caver.cz. (ZIP) [file pcbi.1002708.s006.zip › caver_3.0/source_code/caver/lib/junit/junit-3.8.2-api.zip!/index-all.html]

Index (JUnit API)


|  |  |  |  |
| --- | --- | --- | --- |
| **Overview** \| Package \| Class \| **Tree** \| **Deprecated** \| **Index** \| **Help** | | |  |
| PREV   NEXT | **FRAMES**    **NO FRAMES**     **All Classes** |  |  |


A B C E F G I J P R S T W 

---


## **A**

**ActiveTestSuite** - class junit.extensions.ActiveTestSuite.: A TestSuite for active Tests. **ActiveTestSuite()** - Constructor for class junit.extensions.ActiveTestSuite: **ActiveTestSuite(Class)** - Constructor for class junit.extensions.ActiveTestSuite: **ActiveTestSuite(String)** - Constructor for class junit.extensions.ActiveTestSuite: **ActiveTestSuite(Class, String)** - Constructor for class junit.extensions.ActiveTestSuite: **Assert** - class junit.framework.Assert.: A set of assert methods. **Assert()** - Constructor for class junit.framework.Assert: Protect constructor since it is a static only class **AssertionFailedError** - error junit.framework.AssertionFailedError.: Thrown when an assertion failed. **AssertionFailedError()** - Constructor for class junit.framework.AssertionFailedError: **AssertionFailedError(String)** - Constructor for class junit.framework.AssertionFailedError: **addError(Test, Throwable)** - Method in interface junit.framework.TestListener: An error occurred. **addError(Test, Throwable)** - Method in class junit.framework.TestResult: Adds an error to the list of errors. **addFailure(Test, AssertionFailedError)** - Method in interface junit.framework.TestListener: A failure occurred. **addFailure(Test, AssertionFailedError)** - Method in class junit.framework.TestResult: Adds a failure to the list of failures. **addListener(TestListener)** - Method in class junit.framework.TestResult: Registers a TestListener **addTest(Test)** - Method in class junit.framework.TestSuite: Adds a test to the suite. **addTestSuite(Class)** - Method in class junit.framework.TestSuite: Adds the tests from the given class to the suite **assertEquals(String, Object, Object)** - Static method in class junit.framework.Assert: Asserts that two objects are equal. **assertEquals(Object, Object)** - Static method in class junit.framework.Assert: Asserts that two objects are equal. **assertEquals(String, String, String)** - Static method in class junit.framework.Assert: Asserts that two Strings are equal. **assertEquals(String, String)** - Static method in class junit.framework.Assert: Asserts that two Strings are equal. **assertEquals(String, double, double, double)** - Static method in class junit.framework.Assert: Asserts that two doubles are equal concerning a delta. **assertEquals(double, double, double)** - Static method in class junit.framework.Assert: Asserts that two doubles are equal concerning a delta. **assertEquals(String, float, float, float)** - Static method in class junit.framework.Assert: Asserts that two floats are equal concerning a delta. **assertEquals(float, float, float)** - Static method in class junit.framework.Assert: Asserts that two floats are equal concerning a delta. **assertEquals(String, long, long)** - Static method in class junit.framework.Assert: Asserts that two longs are equal. **assertEquals(long, long)** - Static method in class junit.framework.Assert: Asserts that two longs are equal. **assertEquals(String, boolean, boolean)** - Static method in class junit.framework.Assert: Asserts that two booleans are equal. **assertEquals(boolean, boolean)** - Static method in class junit.framework.Assert: Asserts that two booleans are equal. **assertEquals(String, byte, byte)** - Static method in class junit.framework.Assert: Asserts that two bytes are equal. **assertEquals(byte, byte)** - Static method in class junit.framework.Assert: Asserts that two bytes are equal. **assertEquals(String, char, char)** - Static method in class junit.framework.Assert: Asserts that two chars are equal. **assertEquals(char, char)** - Static method in class junit.framework.Assert: Asserts that two chars are equal. **assertEquals(String, short, short)** - Static method in class junit.framework.Assert: Asserts that two shorts are equal. **assertEquals(short, short)** - Static method in class junit.framework.Assert: Asserts that two shorts are equal. **assertEquals(String, int, int)** - Static method in class junit.framework.Assert: Asserts that two ints are equal. **assertEquals(int, int)** - Static method in class junit.framework.Assert: Asserts that two ints are equal. **assertFalse(String, boolean)** - Static method in class junit.framework.Assert: Asserts that a condition is false. **assertFalse(boolean)** - Static method in class junit.framework.Assert: Asserts that a condition is false. **assertNotNull(Object)** - Static method in class junit.framework.Assert: Asserts that an object isn't null. **assertNotNull(String, Object)** - Static method in class junit.framework.Assert: Asserts that an object isn't null. **assertNotSame(String, Object, Object)** - Static method in class junit.framework.Assert: Asserts that two objects do not refer to the same object. **assertNotSame(Object, Object)** - Static method in class junit.framework.Assert: Asserts that two objects do not refer to the same object. **assertNull(Object)** - Static method in class junit.framework.Assert: Asserts that an object is null. **assertNull(String, Object)** - Static method in class junit.framework.Assert: Asserts that an object is null. **assertSame(String, Object, Object)** - Static method in class junit.framework.Assert: Asserts that two objects refer to the same object. **assertSame(Object, Object)** - Static method in class junit.framework.Assert: Asserts that two objects refer to the same object. **assertTrue(String, boolean)** - Static method in class junit.framework.Assert: Asserts that a condition is true. **assertTrue(boolean)** - Static method in class junit.framework.Assert: Asserts that a condition is true.

---


## **B**

**basicRun(TestResult)** - Method in class junit.extensions.TestDecorator: The basic run behaviour.

---


## **C**

**ComparisonCompactor** - class junit.framework.ComparisonCompactor.: **ComparisonCompactor(int, String, String)** - Constructor for class junit.framework.ComparisonCompactor: **ComparisonFailure** - error junit.framework.ComparisonFailure.: Thrown when an assert equals for Strings failed. **ComparisonFailure(String, String, String)** - Constructor for class junit.framework.ComparisonFailure: Constructs a comparison failure. **compact(String)** - Method in class junit.framework.ComparisonCompactor: **countTestCases()** - Method in class junit.extensions.RepeatedTest: **countTestCases()** - Method in class junit.extensions.TestDecorator: **countTestCases()** - Method in interface junit.framework.Test: Counts the number of test cases that will be run by this test. **countTestCases()** - Method in class junit.framework.TestCase: Counts the number of test cases executed by run(TestResult result). **countTestCases()** - Method in class junit.framework.TestSuite: Counts the number of test cases that will be run by this test. **createResult()** - Method in class junit.framework.TestCase: Creates a default TestResult object **createTest(Class, String)** - Static method in class junit.framework.TestSuite: ...as the moon sets over the early morning Merlin, Oregon mountains, our intrepid adventurers type...

---


## **E**

**ExceptionTestCase** - class junit.extensions.ExceptionTestCase.: A TestCase that expects an Exception of class fExpected to be thrown. **ExceptionTestCase(String, Class)** - Constructor for class junit.extensions.ExceptionTestCase: **endTest(Test)** - Method in interface junit.framework.TestListener: A test ended. **endTest(Test)** - Method in class junit.framework.TestResult: Informs the result that a test was completed. **errorCount()** - Method in class junit.framework.TestResult: Gets the number of detected errors. **errors()** - Method in class junit.framework.TestResult: Returns an Enumeration for the errors **exceptionMessage()** - Method in class junit.framework.TestFailure

---


## **F**

**fErrors** - Variable in class junit.framework.TestResult: **fFailedTest** - Variable in class junit.framework.TestFailure: **fFailures** - Variable in class junit.framework.TestResult: **fListeners** - Variable in class junit.framework.TestResult: **fRunTests** - Variable in class junit.framework.TestResult: **fTest** - Variable in class junit.extensions.TestDecorator: **fThrownException** - Variable in class junit.framework.TestFailure: **fail(String)** - Static method in class junit.framework.Assert: Fails a test with the given message. **fail()** - Static method in class junit.framework.Assert: Fails a test with no message. **failNotEquals(String, Object, Object)** - Static method in class junit.framework.Assert: **failNotSame(String, Object, Object)** - Static method in class junit.framework.Assert: **failSame(String)** - Static method in class junit.framework.Assert: **failedTest()** - Method in class junit.framework.TestFailure: Gets the failed test. **failureCount()** - Method in class junit.framework.TestResult: Gets the number of detected failures. **failures()** - Method in class junit.framework.TestResult: Returns an Enumeration for the failures

---


## **G**

**getActual()** - Method in class junit.framework.ComparisonFailure: Gets the actual string value **getExpected()** - Method in class junit.framework.ComparisonFailure: Gets the expected string value **getMessage()** - Method in class junit.framework.ComparisonFailure: Returns "..." in place of common prefix and "..." in place of common suffix between expected and actual. **getName()** - Method in class junit.framework.TestCase: Gets the name of a TestCase **getName()** - Method in class junit.framework.TestSuite: Returns the name of the suite. **getTest()** - Method in class junit.extensions.TestDecorator: **getTestConstructor(Class)** - Static method in class junit.framework.TestSuite: Gets a constructor which takes a single String as its argument or a no arg constructor.

---


## **I**

**isFailure()** - Method in class junit.framework.TestFailure

---


## **J**

**junit.extensions** - package junit.extensions: **junit.framework** - package junit.framework

---


## **P**

**Protectable** - interface junit.framework.Protectable.: A *Protectable* can be run and can throw a Throwable. **protect()** - Method in interface junit.framework.Protectable: Run the the following method protected.

---


## **R**

**RepeatedTest** - class junit.extensions.RepeatedTest.: A Decorator that runs a test repeatedly. **RepeatedTest(Test, int)** - Constructor for class junit.extensions.RepeatedTest: **removeListener(TestListener)** - Method in class junit.framework.TestResult: Unregisters a TestListener **run(TestResult)** - Method in class junit.extensions.ActiveTestSuite: **run(TestResult)** - Method in class junit.extensions.RepeatedTest: **run(TestResult)** - Method in class junit.extensions.TestDecorator: **run(TestResult)** - Method in class junit.extensions.TestSetup: **run(TestResult)** - Method in interface junit.framework.Test: Runs a test and collects its result in a TestResult instance. **run()** - Method in class junit.framework.TestCase: A convenience method to run this test, collecting the results with a default TestResult object. **run(TestResult)** - Method in class junit.framework.TestCase: Runs the test case and collects the results in TestResult. **run(TestCase)** - Method in class junit.framework.TestResult: Runs a TestCase. **run(TestResult)** - Method in class junit.framework.TestSuite: Runs the tests and collects their result in a TestResult. **runBare()** - Method in class junit.framework.TestCase: Runs the bare test sequence. **runCount()** - Method in class junit.framework.TestResult: Gets the number of run tests. **runFinished()** - Method in class junit.extensions.ActiveTestSuite: **runProtected(Test, Protectable)** - Method in class junit.framework.TestResult: Runs a TestCase. **runTest(Test, TestResult)** - Method in class junit.extensions.ActiveTestSuite: **runTest()** - Method in class junit.extensions.ExceptionTestCase: Execute the test method expecting that an Exception of class fExpected or one of its subclasses will be thrown **runTest()** - Method in class junit.framework.TestCase: Override to run the test and assert its state. **runTest(Test, TestResult)** - Method in class junit.framework.TestSuite

---


## **S**

**setName(String)** - Method in class junit.framework.TestCase: Sets the name of a TestCase **setName(String)** - Method in class junit.framework.TestSuite: Sets the name of the suite. **setUp()** - Method in class junit.extensions.TestSetup: Sets up the fixture. **setUp()** - Method in class junit.framework.TestCase: Sets up the fixture, for example, open a network connection. **shouldStop()** - Method in class junit.framework.TestResult: Checks whether the test run should stop **startTest(Test)** - Method in interface junit.framework.TestListener: A test started. **startTest(Test)** - Method in class junit.framework.TestResult: Informs the result that a test will be started. **stop()** - Method in class junit.framework.TestResult: Marks that the test run should stop.

---


## **T**

**Test** - interface junit.framework.Test.: A *Test* can be run and collect its results. **TestCase** - class junit.framework.TestCase.: A test case defines the fixture to run multiple tests. **TestCase()** - Constructor for class junit.framework.TestCase: No-arg constructor to enable serialization. **TestCase(String)** - Constructor for class junit.framework.TestCase: Constructs a test case with the given name. **TestDecorator** - class junit.extensions.TestDecorator.: A Decorator for Tests. **TestDecorator(Test)** - Constructor for class junit.extensions.TestDecorator: **TestFailure** - class junit.framework.TestFailure.: A `TestFailure` collects a failed test together with the caught exception. **TestFailure(Test, Throwable)** - Constructor for class junit.framework.TestFailure: Constructs a TestFailure with the given test and exception. **TestListener** - interface junit.framework.TestListener.: A Listener for test progress **TestResult** - class junit.framework.TestResult.: A `TestResult` collects the results of executing a test case. **TestResult()** - Constructor for class junit.framework.TestResult: **TestSetup** - class junit.extensions.TestSetup.: A Decorator to set up and tear down additional fixture state. **TestSetup(Test)** - Constructor for class junit.extensions.TestSetup: **TestSuite** - class junit.framework.TestSuite.: A `TestSuite` is a `Composite` of Tests. **TestSuite()** - Constructor for class junit.framework.TestSuite: Constructs an empty TestSuite. **TestSuite(Class)** - Constructor for class junit.framework.TestSuite: Constructs a TestSuite from the given class. **TestSuite(Class, String)** - Constructor for class junit.framework.TestSuite: Constructs a TestSuite from the given class with the given name. **TestSuite(String)** - Constructor for class junit.framework.TestSuite: Constructs an empty TestSuite. **TestSuite(Class[])** - Constructor for class junit.framework.TestSuite: Constructs a TestSuite from the given array of classes. **TestSuite(Class[], String)** - Constructor for class junit.framework.TestSuite: Constructs a TestSuite from the given array of classes with the given name. **tearDown()** - Method in class junit.extensions.TestSetup: Tears down the fixture. **tearDown()** - Method in class junit.framework.TestCase: Tears down the fixture, for example, close a network connection. **testAt(int)** - Method in class junit.framework.TestSuite: Returns the test at the given index **testCount()** - Method in class junit.framework.TestSuite: Returns the number of tests in this suite **tests()** - Method in class junit.framework.TestSuite: Returns the tests as an enumeration **thrownException()** - Method in class junit.framework.TestFailure: Gets the thrown exception. **toString()** - Method in class junit.extensions.RepeatedTest: **toString()** - Method in class junit.extensions.TestDecorator: **toString()** - Method in class junit.framework.TestCase: Returns a string representation of the test case **toString()** - Method in class junit.framework.TestFailure: Returns a short description of the failure. **toString()** - Method in class junit.framework.TestSuite: **trace()** - Method in class junit.framework.TestFailure

---


## **W**

**warning(String)** - Static method in class junit.framework.TestSuite: Returns a test which will fail and log a warning message. **wasSuccessful()** - Method in class junit.framework.TestResult: Returns whether the entire test was successful or not.

---

A B C E F G I J P R S T W


|  |  |  |  |
| --- | --- | --- | --- |
| **Overview** \| Package \| Class \| **Tree** \| **Deprecated** \| **Index** \| **Help** | | |  |
| PREV   NEXT | **FRAMES**    **NO FRAMES**     **All Classes** |  |  |


---
