# Supplementary material for: CAVER 3.0: A Tool for the Analysis of Transport Pathways in Dynamic Protein Structures
Source: PLoS Comput Biol. 2012 Oct 18;8(10):e1002708. doi: 10.1371/journal.pcbi.1002708 (PMC3475669; doi:10.1371/journal.pcbi.1002708)
Supplement: Software S1 — CAVER 3.0 package containing CAVER 3.0 executable, source code, license, documentation and examples. The latest release of CAVER 3.0 can be downloaded from http://www.caver.cz. (ZIP) [file pcbi.1002708.s006.zip › caver_3.0/source_code/caver/lib/junit/junit-3.8.2-api.zip!/junit/extensions/ActiveTestSuite.html]

ActiveTestSuite (JUnit API)


|  |  |  |  |
| --- | --- | --- | --- |
| **Overview** \| **Package** \| **Class** \| **Tree** \| **Deprecated** \| **Index** \| **Help** | | |  |
| PREV CLASS   **NEXT CLASS** | **FRAMES**    **NO FRAMES**     **All Classes** |  |  |
| SUMMARY: NESTED \| FIELD \| CONSTR \| METHOD | DETAIL: FIELD \| CONSTR \| METHOD |  |  |


---


## junit.extensions Class ActiveTestSuite

```
java.lang.Object
  junit.framework.TestSuite
      junit.extensions.ActiveTestSuite
```

**All Implemented Interfaces:**: Test

---

public class **ActiveTestSuite** extends TestSuite

A TestSuite for active Tests. It runs each
test in a separate thread and waits until all
threads have terminated.
-- Aarhus Radisson Scandinavian Center 11th floor

---

**Constructor Summary**

`ActiveTestSuite()`

`ActiveTestSuite(java.lang.Class theClass)`

`ActiveTestSuite(java.lang.Class theClass,
java.lang.String name)`

`ActiveTestSuite(java.lang.String name)`


|  |  |
| --- | --- |
| **Method Summary** | |
| `void` | `run(TestResult result)`             Runs the tests and collects their result in a TestResult. |
| `void` | `runFinished()` |
| `void` | `runTest(Test test, TestResult result)` |

**Methods inherited from class junit.framework.TestSuite**

`addTest, addTestSuite, countTestCases, createTest, getName, getTestConstructor, setName, testAt, testCount, tests, toString, warning`

**Methods inherited from class java.lang.Object**

`clone, equals, finalize, getClass, hashCode, notify, notifyAll, wait, wait, wait`

**Constructor Detail**

### ActiveTestSuite

```
public ActiveTestSuite()
```

---


### ActiveTestSuite

```
public ActiveTestSuite(java.lang.Class theClass)
```

---


### ActiveTestSuite

```
public ActiveTestSuite(java.lang.String name)
```

---


### ActiveTestSuite

```
public ActiveTestSuite(java.lang.Class theClass,
                       java.lang.String name)
```


**Method Detail**

### run

```
public void run(TestResult result)
```

:   **Description copied from class: `TestSuite`**
:   Runs the tests and collects their result in a TestResult.

    :   **Specified by:**: `run` in interface `Test` **Overrides:**: `run` in class `TestSuite`

---


### runTest

```
public void runTest(Test test,
                    TestResult result)
```

:   **Overrides:**: `runTest` in class `TestSuite`

---


### runFinished

```
public void runFinished()
```


---


|  |  |  |  |
| --- | --- | --- | --- |
| **Overview** \| **Package** \| **Class** \| **Tree** \| **Deprecated** \| **Index** \| **Help** | | |  |
| PREV CLASS   **NEXT CLASS** | **FRAMES**    **NO FRAMES**     **All Classes** |  |  |
| SUMMARY: NESTED \| FIELD \| CONSTR \| METHOD | DETAIL: FIELD \| CONSTR \| METHOD |  |  |


---
