# Supplementary material for: CAVER 3.0: A Tool for the Analysis of Transport Pathways in Dynamic Protein Structures
Source: PLoS Comput Biol. 2012 Oct 18;8(10):e1002708. doi: 10.1371/journal.pcbi.1002708 (PMC3475669; doi:10.1371/journal.pcbi.1002708)
Supplement: Software S1 — CAVER 3.0 package containing CAVER 3.0 executable, source code, license, documentation and examples. The latest release of CAVER 3.0 can be downloaded from http://www.caver.cz. (ZIP) [file pcbi.1002708.s006.zip › caver_3.0/source_code/caver/lib/junit/junit-3.8.2-api.zip!/junit/extensions/ExceptionTestCase.html]

ExceptionTestCase (JUnit API)


|  |  |  |  |
| --- | --- | --- | --- |
| **Overview** \| **Package** \| **Class** \| **Tree** \| **Deprecated** \| **Index** \| **Help** | | |  |
| **PREV CLASS**   **NEXT CLASS** | **FRAMES**    **NO FRAMES**     **All Classes** |  |  |
| SUMMARY: NESTED \| FIELD \| CONSTR \| METHOD | DETAIL: FIELD \| CONSTR \| METHOD |  |  |


---


## junit.extensions Class ExceptionTestCase

```
java.lang.Object
  junit.framework.Assert
      junit.framework.TestCase
          junit.extensions.ExceptionTestCase
```

**All Implemented Interfaces:**: Test

---

public class **ExceptionTestCase** extends TestCase

A TestCase that expects an Exception of class fExpected to be thrown.
The other way to check that an expected exception is thrown is:

```
 try {
   shouldThrow();
 }
 catch (SpecialException e) {
   return;
 }
 fail("Expected SpecialException");
```

To use ExceptionTestCase, create a TestCase like:

```
 new ExceptionTestCase("testShouldThrow", SpecialException.class);
```

---

**Constructor Summary**

`ExceptionTestCase(java.lang.String name,
java.lang.Class exception)`


|  |  |
| --- | --- |
| **Method Summary** | |
| `protected  void` | `runTest()`             Execute the test method expecting that an Exception of class fExpected or one of its subclasses will be thrown |

**Methods inherited from class junit.framework.TestCase**

`countTestCases, createResult, getName, run, run, runBare, setName, setUp, tearDown, toString`

**Methods inherited from class junit.framework.Assert**

`assertEquals, assertEquals, assertEquals, assertEquals, assertEquals, assertEquals, assertEquals, assertEquals, assertEquals, assertEquals, assertEquals, assertEquals, assertEquals, assertEquals, assertEquals, assertEquals, assertEquals, assertEquals, assertEquals, assertEquals, assertFalse, assertFalse, assertNotNull, assertNotNull, assertNotSame, assertNotSame, assertNull, assertNull, assertSame, assertSame, assertTrue, assertTrue, fail, fail, failNotEquals, failNotSame, failSame`

**Methods inherited from class java.lang.Object**

`clone, equals, finalize, getClass, hashCode, notify, notifyAll, wait, wait, wait`

**Constructor Detail**

### ExceptionTestCase

```
public ExceptionTestCase(java.lang.String name,
                         java.lang.Class exception)
```


**Method Detail**

### runTest

```
protected void runTest()
                throws java.lang.Throwable
```

:   Execute the test method expecting that an Exception of
    class fExpected or one of its subclasses will be thrown

    :   **Overrides:**: `runTest` in class `TestCase`
    :   **Throws:**: `java.lang.Throwable` - if any exception is thrown


---


|  |  |  |  |
| --- | --- | --- | --- |
| **Overview** \| **Package** \| **Class** \| **Tree** \| **Deprecated** \| **Index** \| **Help** | | |  |
| **PREV CLASS**   **NEXT CLASS** | **FRAMES**    **NO FRAMES**     **All Classes** |  |  |
| SUMMARY: NESTED \| FIELD \| CONSTR \| METHOD | DETAIL: FIELD \| CONSTR \| METHOD |  |  |


---
