# Supplementary material for: CAVER 3.0: A Tool for the Analysis of Transport Pathways in Dynamic Protein Structures
Source: PLoS Comput Biol. 2012 Oct 18;8(10):e1002708. doi: 10.1371/journal.pcbi.1002708 (PMC3475669; doi:10.1371/journal.pcbi.1002708)
Supplement: Software S1 — CAVER 3.0 package containing CAVER 3.0 executable, source code, license, documentation and examples. The latest release of CAVER 3.0 can be downloaded from http://www.caver.cz. (ZIP) [file pcbi.1002708.s006.zip › caver_3.0/source_code/caver/lib/junit/junit-3.8.2-api.zip!/junit/extensions/package-summary.html]

junit.extensions (JUnit API)


|  |  |  |  |
| --- | --- | --- | --- |
| **Overview** \| **Package** \| Class \| **Tree** \| **Deprecated** \| **Index** \| **Help** | | |  |
| PREV PACKAGE   **NEXT PACKAGE** | **FRAMES**    **NO FRAMES**     **All Classes** |  |  |


---

## Package junit.extensions

|  |  |
| --- | --- |
| **Class Summary** | |
| **ActiveTestSuite** | A TestSuite for active Tests. |
| **ExceptionTestCase** | A TestCase that expects an Exception of class fExpected to be thrown. |
| **RepeatedTest** | A Decorator that runs a test repeatedly. |
| **TestDecorator** | A Decorator for Tests. |
| **TestSetup** | A Decorator to set up and tear down additional fixture state. |

---


|  |  |  |  |
| --- | --- | --- | --- |
| **Overview** \| **Package** \| Class \| **Tree** \| **Deprecated** \| **Index** \| **Help** | | |  |
| PREV PACKAGE   **NEXT PACKAGE** | **FRAMES**    **NO FRAMES**     **All Classes** |  |  |


---
