# Supplementary material for: CAVER 3.0: A Tool for the Analysis of Transport Pathways in Dynamic Protein Structures
Source: PLoS Comput Biol. 2012 Oct 18;8(10):e1002708. doi: 10.1371/journal.pcbi.1002708 (PMC3475669; doi:10.1371/journal.pcbi.1002708)
Supplement: Software S1 — CAVER 3.0 package containing CAVER 3.0 executable, source code, license, documentation and examples. The latest release of CAVER 3.0 can be downloaded from http://www.caver.cz. (ZIP) [file pcbi.1002708.s006.zip › caver_3.0/source_code/caver/lib/junit/junit-3.8.2-api.zip!/junit/extensions/RepeatedTest.html]

RepeatedTest (JUnit API)


|  |  |  |  |
| --- | --- | --- | --- |
| **Overview** \| **Package** \| **Class** \| **Tree** \| **Deprecated** \| **Index** \| **Help** | | |  |
| **PREV CLASS**   **NEXT CLASS** | **FRAMES**    **NO FRAMES**     **All Classes** |  |  |
| SUMMARY: NESTED \| FIELD \| CONSTR \| METHOD | DETAIL: FIELD \| CONSTR \| METHOD |  |  |


---


## junit.extensions Class RepeatedTest

```
java.lang.Object
  junit.framework.Assert
      junit.extensions.TestDecorator
          junit.extensions.RepeatedTest
```

**All Implemented Interfaces:**: Test

---

public class **RepeatedTest** extends TestDecorator

A Decorator that runs a test repeatedly.

---

**Field Summary**

**Fields inherited from class junit.extensions.TestDecorator**

`fTest`


**Constructor Summary**

`RepeatedTest(Test test,
int repeat)`


|  |  |
| --- | --- |
| **Method Summary** | |
| `int` | `countTestCases()`             Counts the number of test cases that will be run by this test. |
| `void` | `run(TestResult result)`             Runs a test and collects its result in a TestResult instance. |
| `java.lang.String` | `toString()` |

**Methods inherited from class junit.extensions.TestDecorator**

`basicRun, getTest`

**Methods inherited from class junit.framework.Assert**

`assertEquals, assertEquals, assertEquals, assertEquals, assertEquals, assertEquals, assertEquals, assertEquals, assertEquals, assertEquals, assertEquals, assertEquals, assertEquals, assertEquals, assertEquals, assertEquals, assertEquals, assertEquals, assertEquals, assertEquals, assertFalse, assertFalse, assertNotNull, assertNotNull, assertNotSame, assertNotSame, assertNull, assertNull, assertSame, assertSame, assertTrue, assertTrue, fail, fail, failNotEquals, failNotSame, failSame`

**Methods inherited from class java.lang.Object**

`clone, equals, finalize, getClass, hashCode, notify, notifyAll, wait, wait, wait`

**Constructor Detail**

### RepeatedTest

```
public RepeatedTest(Test test,
                    int repeat)
```


**Method Detail**

### countTestCases

```
public int countTestCases()
```

:   **Description copied from interface: `Test`**
:   Counts the number of test cases that will be run by this test.

    :   **Specified by:**: `countTestCases` in interface `Test` **Overrides:**: `countTestCases` in class `TestDecorator`

---


### run

```
public void run(TestResult result)
```

:   **Description copied from interface: `Test`**
:   Runs a test and collects its result in a TestResult instance.

    :   **Specified by:**: `run` in interface `Test` **Overrides:**: `run` in class `TestDecorator`

---


### toString

```
public java.lang.String toString()
```

:   **Overrides:**: `toString` in class `TestDecorator`


---


|  |  |  |  |
| --- | --- | --- | --- |
| **Overview** \| **Package** \| **Class** \| **Tree** \| **Deprecated** \| **Index** \| **Help** | | |  |
| **PREV CLASS**   **NEXT CLASS** | **FRAMES**    **NO FRAMES**     **All Classes** |  |  |
| SUMMARY: NESTED \| FIELD \| CONSTR \| METHOD | DETAIL: FIELD \| CONSTR \| METHOD |  |  |


---
