# Supplementary material for: CAVER 3.0: A Tool for the Analysis of Transport Pathways in Dynamic Protein Structures
Source: PLoS Comput Biol. 2012 Oct 18;8(10):e1002708. doi: 10.1371/journal.pcbi.1002708 (PMC3475669; doi:10.1371/journal.pcbi.1002708)
Supplement: Software S1 — CAVER 3.0 package containing CAVER 3.0 executable, source code, license, documentation and examples. The latest release of CAVER 3.0 can be downloaded from http://www.caver.cz. (ZIP) [file pcbi.1002708.s006.zip › caver_3.0/source_code/caver/lib/junit/junit-3.8.2-api.zip!/junit/extensions/TestDecorator.html]

TestDecorator (JUnit API)


|  |  |  |  |
| --- | --- | --- | --- |
| **Overview** \| **Package** \| **Class** \| **Tree** \| **Deprecated** \| **Index** \| **Help** | | |  |
| **PREV CLASS**   **NEXT CLASS** | **FRAMES**    **NO FRAMES**     **All Classes** |  |  |
| SUMMARY: NESTED \| FIELD \| CONSTR \| METHOD | DETAIL: FIELD \| CONSTR \| METHOD |  |  |


---


## junit.extensions Class TestDecorator

```
java.lang.Object
  junit.framework.Assert
      junit.extensions.TestDecorator
```

**All Implemented Interfaces:**: Test

**Direct Known Subclasses:**: RepeatedTest, TestSetup

---

public class **TestDecorator** extends Assert implements Test

A Decorator for Tests. Use TestDecorator as the base class
for defining new test decorators. Test decorator subclasses
can be introduced to add behaviour before or after a test
is run.

---

|  |  |
| --- | --- |
| **Field Summary** | |
| `protected  Test` | `fTest` |


**Constructor Summary**

`TestDecorator(Test test)`


|  |  |
| --- | --- |
| **Method Summary** | |
| `void` | `basicRun(TestResult result)`             The basic run behaviour. |
| `int` | `countTestCases()`             Counts the number of test cases that will be run by this test. |
| `Test` | `getTest()` |
| `void` | `run(TestResult result)`             Runs a test and collects its result in a TestResult instance. |
| `java.lang.String` | `toString()` |

**Methods inherited from class junit.framework.Assert**

`assertEquals, assertEquals, assertEquals, assertEquals, assertEquals, assertEquals, assertEquals, assertEquals, assertEquals, assertEquals, assertEquals, assertEquals, assertEquals, assertEquals, assertEquals, assertEquals, assertEquals, assertEquals, assertEquals, assertEquals, assertFalse, assertFalse, assertNotNull, assertNotNull, assertNotSame, assertNotSame, assertNull, assertNull, assertSame, assertSame, assertTrue, assertTrue, fail, fail, failNotEquals, failNotSame, failSame`

**Methods inherited from class java.lang.Object**

`clone, equals, finalize, getClass, hashCode, notify, notifyAll, wait, wait, wait`

**Field Detail**

### fTest

```
protected Test fTest
```


**Constructor Detail**

### TestDecorator

```
public TestDecorator(Test test)
```


**Method Detail**

### basicRun

```
public void basicRun(TestResult result)
```

:   The basic run behaviour.

---


### countTestCases

```
public int countTestCases()
```

:   **Description copied from interface: `Test`**
:   Counts the number of test cases that will be run by this test.

    :   **Specified by:**: `countTestCases` in interface `Test`

---


### run

```
public void run(TestResult result)
```

:   **Description copied from interface: `Test`**
:   Runs a test and collects its result in a TestResult instance.

    :   **Specified by:**: `run` in interface `Test`

---


### toString

```
public java.lang.String toString()
```

---


### getTest

```
public Test getTest()
```


---


|  |  |  |  |
| --- | --- | --- | --- |
| **Overview** \| **Package** \| **Class** \| **Tree** \| **Deprecated** \| **Index** \| **Help** | | |  |
| **PREV CLASS**   **NEXT CLASS** | **FRAMES**    **NO FRAMES**     **All Classes** |  |  |
| SUMMARY: NESTED \| FIELD \| CONSTR \| METHOD | DETAIL: FIELD \| CONSTR \| METHOD |  |  |


---
