# Supplementary material for: CAVER 3.0: A Tool for the Analysis of Transport Pathways in Dynamic Protein Structures
Source: PLoS Comput Biol. 2012 Oct 18;8(10):e1002708. doi: 10.1371/journal.pcbi.1002708 (PMC3475669; doi:10.1371/journal.pcbi.1002708)
Supplement: Software S1 — CAVER 3.0 package containing CAVER 3.0 executable, source code, license, documentation and examples. The latest release of CAVER 3.0 can be downloaded from http://www.caver.cz. (ZIP) [file pcbi.1002708.s006.zip › caver_3.0/source_code/caver/lib/junit/junit-3.8.2-api.zip!/junit/extensions/TestSetup.html]

TestSetup (JUnit API)


|  |  |  |  |
| --- | --- | --- | --- |
| **Overview** \| **Package** \| **Class** \| **Tree** \| **Deprecated** \| **Index** \| **Help** | | |  |
| **PREV CLASS**   NEXT CLASS | **FRAMES**    **NO FRAMES**     **All Classes** |  |  |
| SUMMARY: NESTED \| FIELD \| CONSTR \| METHOD | DETAIL: FIELD \| CONSTR \| METHOD |  |  |


---


## junit.extensions Class TestSetup

```
java.lang.Object
  junit.framework.Assert
      junit.extensions.TestDecorator
          junit.extensions.TestSetup
```

**All Implemented Interfaces:**: Test

---

public class **TestSetup** extends TestDecorator

A Decorator to set up and tear down additional fixture state.
Subclass TestSetup and insert it into your tests when you want
to set up additional state once before the tests are run.

---

**Field Summary**

**Fields inherited from class junit.extensions.TestDecorator**

`fTest`


**Constructor Summary**

`TestSetup(Test test)`


|  |  |
| --- | --- |
| **Method Summary** | |
| `void` | `run(TestResult result)`             Runs a test and collects its result in a TestResult instance. |
| `protected  void` | `setUp()`             Sets up the fixture. |
| `protected  void` | `tearDown()`             Tears down the fixture. |

**Methods inherited from class junit.extensions.TestDecorator**

`basicRun, countTestCases, getTest, toString`

**Methods inherited from class junit.framework.Assert**

`assertEquals, assertEquals, assertEquals, assertEquals, assertEquals, assertEquals, assertEquals, assertEquals, assertEquals, assertEquals, assertEquals, assertEquals, assertEquals, assertEquals, assertEquals, assertEquals, assertEquals, assertEquals, assertEquals, assertEquals, assertFalse, assertFalse, assertNotNull, assertNotNull, assertNotSame, assertNotSame, assertNull, assertNull, assertSame, assertSame, assertTrue, assertTrue, fail, fail, failNotEquals, failNotSame, failSame`

**Methods inherited from class java.lang.Object**

`clone, equals, finalize, getClass, hashCode, notify, notifyAll, wait, wait, wait`

**Constructor Detail**

### TestSetup

```
public TestSetup(Test test)
```


**Method Detail**

### run

```
public void run(TestResult result)
```

:   **Description copied from interface: `Test`**
:   Runs a test and collects its result in a TestResult instance.

    :   **Specified by:**: `run` in interface `Test` **Overrides:**: `run` in class `TestDecorator`

---


### setUp

```
protected void setUp()
              throws java.lang.Exception
```

:   Sets up the fixture. Override to set up additional fixture
    state.

    :   **Throws:**: `java.lang.Exception`

---


### tearDown

```
protected void tearDown()
                 throws java.lang.Exception
```

:   Tears down the fixture. Override to tear down the additional
    fixture state.

    :   **Throws:**: `java.lang.Exception`


---


|  |  |  |  |
| --- | --- | --- | --- |
| **Overview** \| **Package** \| **Class** \| **Tree** \| **Deprecated** \| **Index** \| **Help** | | |  |
| **PREV CLASS**   NEXT CLASS | **FRAMES**    **NO FRAMES**     **All Classes** |  |  |
| SUMMARY: NESTED \| FIELD \| CONSTR \| METHOD | DETAIL: FIELD \| CONSTR \| METHOD |  |  |


---
