# Supplementary material for: CAVER 3.0: A Tool for the Analysis of Transport Pathways in Dynamic Protein Structures
Source: PLoS Comput Biol. 2012 Oct 18;8(10):e1002708. doi: 10.1371/journal.pcbi.1002708 (PMC3475669; doi:10.1371/journal.pcbi.1002708)
Supplement: Software S1 — CAVER 3.0 package containing CAVER 3.0 executable, source code, license, documentation and examples. The latest release of CAVER 3.0 can be downloaded from http://www.caver.cz. (ZIP) [file pcbi.1002708.s006.zip › caver_3.0/source_code/caver/lib/junit/junit-3.8.2-api.zip!/junit/framework/Assert.html]

Assert (JUnit API)


|  |  |  |  |
| --- | --- | --- | --- |
| **Overview** \| **Package** \| **Class** \| **Tree** \| **Deprecated** \| **Index** \| **Help** | | |  |
| PREV CLASS   **NEXT CLASS** | **FRAMES**    **NO FRAMES**     **All Classes** |  |  |
| SUMMARY: NESTED \| FIELD \| CONSTR \| METHOD | DETAIL: FIELD \| CONSTR \| METHOD |  |  |


---


## junit.framework Class Assert

```
java.lang.Object
  junit.framework.Assert
```

**Direct Known Subclasses:**: TestCase, TestDecorator

---

public class **Assert** extends java.lang.Object

A set of assert methods. Messages are only displayed when an assert fails.

---

|  |  |
| --- | --- |
| **Constructor Summary** | |
| `protected` | `Assert()`             Protect constructor since it is a static only class |


|  |  |
| --- | --- |
| **Method Summary** | |
| `static void` | `assertEquals(boolean expected, boolean actual)`             Asserts that two booleans are equal. |
| `static void` | `assertEquals(byte expected, byte actual)`             Asserts that two bytes are equal. |
| `static void` | `assertEquals(char expected, char actual)`             Asserts that two chars are equal. |
| `static void` | `assertEquals(double expected, double actual, double delta)`             Asserts that two doubles are equal concerning a delta. |
| `static void` | `assertEquals(float expected, float actual, float delta)`             Asserts that two floats are equal concerning a delta. |
| `static void` | `assertEquals(int expected, int actual)`             Asserts that two ints are equal. |
| `static void` | `assertEquals(long expected, long actual)`             Asserts that two longs are equal. |
| `static void` | `assertEquals(java.lang.Object expected, java.lang.Object actual)`             Asserts that two objects are equal. |
| `static void` | `assertEquals(short expected, short actual)`             Asserts that two shorts are equal. |
| `static void` | `assertEquals(java.lang.String message, boolean expected, boolean actual)`             Asserts that two booleans are equal. |
| `static void` | `assertEquals(java.lang.String message, byte expected, byte actual)`             Asserts that two bytes are equal. |
| `static void` | `assertEquals(java.lang.String message, char expected, char actual)`             Asserts that two chars are equal. |
| `static void` | `assertEquals(java.lang.String message, double expected, double actual, double delta)`             Asserts that two doubles are equal concerning a delta. |
| `static void` | `assertEquals(java.lang.String message, float expected, float actual, float delta)`             Asserts that two floats are equal concerning a delta. |
| `static void` | `assertEquals(java.lang.String message, int expected, int actual)`             Asserts that two ints are equal. |
| `static void` | `assertEquals(java.lang.String message, long expected, long actual)`             Asserts that two longs are equal. |
| `static void` | `assertEquals(java.lang.String message, java.lang.Object expected, java.lang.Object actual)`             Asserts that two objects are equal. |
| `static void` | `assertEquals(java.lang.String message, short expected, short actual)`             Asserts that two shorts are equal. |
| `static void` | `assertEquals(java.lang.String expected, java.lang.String actual)`             Asserts that two Strings are equal. |
| `static void` | `assertEquals(java.lang.String message, java.lang.String expected, java.lang.String actual)`             Asserts that two Strings are equal. |
| `static void` | `assertFalse(boolean condition)`             Asserts that a condition is false. |
| `static void` | `assertFalse(java.lang.String message, boolean condition)`             Asserts that a condition is false. |
| `static void` | `assertNotNull(java.lang.Object object)`             Asserts that an object isn't null. |
| `static void` | `assertNotNull(java.lang.String message, java.lang.Object object)`             Asserts that an object isn't null. |
| `static void` | `assertNotSame(java.lang.Object expected, java.lang.Object actual)`             Asserts that two objects do not refer to the same object. |
| `static void` | `assertNotSame(java.lang.String message, java.lang.Object expected, java.lang.Object actual)`             Asserts that two objects do not refer to the same object. |
| `static void` | `assertNull(java.lang.Object object)`             Asserts that an object is null. |
| `static void` | `assertNull(java.lang.String message, java.lang.Object object)`             Asserts that an object is null. |
| `static void` | `assertSame(java.lang.Object expected, java.lang.Object actual)`             Asserts that two objects refer to the same object. |
| `static void` | `assertSame(java.lang.String message, java.lang.Object expected, java.lang.Object actual)`             Asserts that two objects refer to the same object. |
| `static void` | `assertTrue(boolean condition)`             Asserts that a condition is true. |
| `static void` | `assertTrue(java.lang.String message, boolean condition)`             Asserts that a condition is true. |
| `static void` | `fail()`             Fails a test with no message. |
| `static void` | `fail(java.lang.String message)`             Fails a test with the given message. |
| `static void` | `failNotEquals(java.lang.String message, java.lang.Object expected, java.lang.Object actual)` |
| `static void` | `failNotSame(java.lang.String message, java.lang.Object expected, java.lang.Object actual)` |
| `static void` | `failSame(java.lang.String message)` |

**Methods inherited from class java.lang.Object**

`clone, equals, finalize, getClass, hashCode, notify, notifyAll, toString, wait, wait, wait`

**Constructor Detail**

### Assert

```
protected Assert()
```

:   Protect constructor since it is a static only class


**Method Detail**

### assertTrue

```
public static void assertTrue(java.lang.String message,
                              boolean condition)
```

:   Asserts that a condition is true. If it isn't it throws
    an AssertionFailedError with the given message.

---


### assertTrue

```
public static void assertTrue(boolean condition)
```

:   Asserts that a condition is true. If it isn't it throws
    an AssertionFailedError.

---


### assertFalse

```
public static void assertFalse(java.lang.String message,
                               boolean condition)
```

:   Asserts that a condition is false. If it isn't it throws
    an AssertionFailedError with the given message.

---


### assertFalse

```
public static void assertFalse(boolean condition)
```

:   Asserts that a condition is false. If it isn't it throws
    an AssertionFailedError.

---


### fail

```
public static void fail(java.lang.String message)
```

:   Fails a test with the given message.

---


### fail

```
public static void fail()
```

:   Fails a test with no message.

---


### assertEquals

```
public static void assertEquals(java.lang.String message,
                                java.lang.Object expected,
                                java.lang.Object actual)
```

:   Asserts that two objects are equal. If they are not
    an AssertionFailedError is thrown with the given message.

---


### assertEquals

```
public static void assertEquals(java.lang.Object expected,
                                java.lang.Object actual)
```

:   Asserts that two objects are equal. If they are not
    an AssertionFailedError is thrown.

---


### assertEquals

```
public static void assertEquals(java.lang.String message,
                                java.lang.String expected,
                                java.lang.String actual)
```

:   Asserts that two Strings are equal.

---


### assertEquals

```
public static void assertEquals(java.lang.String expected,
                                java.lang.String actual)
```

:   Asserts that two Strings are equal.

---


### assertEquals

```
public static void assertEquals(java.lang.String message,
                                double expected,
                                double actual,
                                double delta)
```

:   Asserts that two doubles are equal concerning a delta. If they are not
    an AssertionFailedError is thrown with the given message. If the expected
    value is infinity then the delta value is ignored.

---


### assertEquals

```
public static void assertEquals(double expected,
                                double actual,
                                double delta)
```

:   Asserts that two doubles are equal concerning a delta. If the expected
    value is infinity then the delta value is ignored.

---


### assertEquals

```
public static void assertEquals(java.lang.String message,
                                float expected,
                                float actual,
                                float delta)
```

:   Asserts that two floats are equal concerning a delta. If they are not
    an AssertionFailedError is thrown with the given message. If the expected
    value is infinity then the delta value is ignored.

---


### assertEquals

```
public static void assertEquals(float expected,
                                float actual,
                                float delta)
```

:   Asserts that two floats are equal concerning a delta. If the expected
    value is infinity then the delta value is ignored.

---


### assertEquals

```
public static void assertEquals(java.lang.String message,
                                long expected,
                                long actual)
```

:   Asserts that two longs are equal. If they are not
    an AssertionFailedError is thrown with the given message.

---


### assertEquals

```
public static void assertEquals(long expected,
                                long actual)
```

:   Asserts that two longs are equal.

---


### assertEquals

```
public static void assertEquals(java.lang.String message,
                                boolean expected,
                                boolean actual)
```

:   Asserts that two booleans are equal. If they are not
    an AssertionFailedError is thrown with the given message.

---


### assertEquals

```
public static void assertEquals(boolean expected,
                                boolean actual)
```

:   Asserts that two booleans are equal.

---


### assertEquals

```
public static void assertEquals(java.lang.String message,
                                byte expected,
                                byte actual)
```

:   Asserts that two bytes are equal. If they are not
    an AssertionFailedError is thrown with the given message.

---


### assertEquals

```
public static void assertEquals(byte expected,
                                byte actual)
```

:   Asserts that two bytes are equal.

---


### assertEquals

```
public static void assertEquals(java.lang.String message,
                                char expected,
                                char actual)
```

:   Asserts that two chars are equal. If they are not
    an AssertionFailedError is thrown with the given message.

---


### assertEquals

```
public static void assertEquals(char expected,
                                char actual)
```

:   Asserts that two chars are equal.

---


### assertEquals

```
public static void assertEquals(java.lang.String message,
                                short expected,
                                short actual)
```

:   Asserts that two shorts are equal. If they are not
    an AssertionFailedError is thrown with the given message.

---


### assertEquals

```
public static void assertEquals(short expected,
                                short actual)
```

:   Asserts that two shorts are equal.

---


### assertEquals

```
public static void assertEquals(java.lang.String message,
                                int expected,
                                int actual)
```

:   Asserts that two ints are equal. If they are not
    an AssertionFailedError is thrown with the given message.

---


### assertEquals

```
public static void assertEquals(int expected,
                                int actual)
```

:   Asserts that two ints are equal.

---


### assertNotNull

```
public static void assertNotNull(java.lang.Object object)
```

:   Asserts that an object isn't null.

---


### assertNotNull

```
public static void assertNotNull(java.lang.String message,
                                 java.lang.Object object)
```

:   Asserts that an object isn't null. If it is
    an AssertionFailedError is thrown with the given message.

---


### assertNull

```
public static void assertNull(java.lang.Object object)
```

:   Asserts that an object is null.

---


### assertNull

```
public static void assertNull(java.lang.String message,
                              java.lang.Object object)
```

:   Asserts that an object is null. If it is not
    an AssertionFailedError is thrown with the given message.

---


### assertSame

```
public static void assertSame(java.lang.String message,
                              java.lang.Object expected,
                              java.lang.Object actual)
```

:   Asserts that two objects refer to the same object. If they are not
    an AssertionFailedError is thrown with the given message.

---


### assertSame

```
public static void assertSame(java.lang.Object expected,
                              java.lang.Object actual)
```

:   Asserts that two objects refer to the same object. If they are not
    the same an AssertionFailedError is thrown.

---


### assertNotSame

```
public static void assertNotSame(java.lang.String message,
                                 java.lang.Object expected,
                                 java.lang.Object actual)
```

:   Asserts that two objects do not refer to the same object. If they do
    refer to the same object an AssertionFailedError is thrown with the
    given message.

---


### assertNotSame

```
public static void assertNotSame(java.lang.Object expected,
                                 java.lang.Object actual)
```

:   Asserts that two objects do not refer to the same object. If they do
    refer to the same object an AssertionFailedError is thrown.

---


### failSame

```
public static void failSame(java.lang.String message)
```

---


### failNotSame

```
public static void failNotSame(java.lang.String message,
                               java.lang.Object expected,
                               java.lang.Object actual)
```

---


### failNotEquals

```
public static void failNotEquals(java.lang.String message,
                                 java.lang.Object expected,
                                 java.lang.Object actual)
```


---


|  |  |  |  |
| --- | --- | --- | --- |
| **Overview** \| **Package** \| **Class** \| **Tree** \| **Deprecated** \| **Index** \| **Help** | | |  |
| PREV CLASS   **NEXT CLASS** | **FRAMES**    **NO FRAMES**     **All Classes** |  |  |
| SUMMARY: NESTED \| FIELD \| CONSTR \| METHOD | DETAIL: FIELD \| CONSTR \| METHOD |  |  |


---
