# Supplementary material for: CAVER 3.0: A Tool for the Analysis of Transport Pathways in Dynamic Protein Structures
Source: PLoS Comput Biol. 2012 Oct 18;8(10):e1002708. doi: 10.1371/journal.pcbi.1002708 (PMC3475669; doi:10.1371/journal.pcbi.1002708)
Supplement: Software S1 — CAVER 3.0 package containing CAVER 3.0 executable, source code, license, documentation and examples. The latest release of CAVER 3.0 can be downloaded from http://www.caver.cz. (ZIP) [file pcbi.1002708.s006.zip › caver_3.0/source_code/caver/lib/junit/junit-3.8.2-api.zip!/junit/framework/AssertionFailedError.html]

AssertionFailedError (JUnit API)


|  |  |  |  |
| --- | --- | --- | --- |
| **Overview** \| **Package** \| **Class** \| **Tree** \| **Deprecated** \| **Index** \| **Help** | | |  |
| PREV CLASS   **NEXT CLASS** | **FRAMES**    **NO FRAMES**     **All Classes** |  |  |
| SUMMARY: NESTED \| FIELD \| CONSTR \| METHOD | DETAIL: FIELD \| CONSTR \| METHOD |  |  |


---


## junit.framework Class AssertionFailedError

```
java.lang.Object
  java.lang.Throwable
      java.lang.Error
          junit.framework.AssertionFailedError
```

**All Implemented Interfaces:**: java.io.Serializable

**Direct Known Subclasses:**: ComparisonFailure

---

public class **AssertionFailedError** extends java.lang.Error

Thrown when an assertion failed.

**See Also:**: Serialized Form

---

**Constructor Summary**

`AssertionFailedError()`

`AssertionFailedError(java.lang.String message)`


**Methods inherited from class java.lang.Throwable**

`fillInStackTrace, getCause, getLocalizedMessage, getMessage, getStackTrace, initCause, printStackTrace, printStackTrace, printStackTrace, setStackTrace, toString`

**Methods inherited from class java.lang.Object**

`clone, equals, finalize, getClass, hashCode, notify, notifyAll, wait, wait, wait`

**Constructor Detail**

### AssertionFailedError

```
public AssertionFailedError()
```

---


### AssertionFailedError

```
public AssertionFailedError(java.lang.String message)
```


---


|  |  |  |  |
| --- | --- | --- | --- |
| **Overview** \| **Package** \| **Class** \| **Tree** \| **Deprecated** \| **Index** \| **Help** | | |  |
| PREV CLASS   **NEXT CLASS** | **FRAMES**    **NO FRAMES**     **All Classes** |  |  |
| SUMMARY: NESTED \| FIELD \| CONSTR \| METHOD | DETAIL: FIELD \| CONSTR \| METHOD |  |  |


---
