# Supplementary material for: CAVER 3.0: A Tool for the Analysis of Transport Pathways in Dynamic Protein Structures
Source: PLoS Comput Biol. 2012 Oct 18;8(10):e1002708. doi: 10.1371/journal.pcbi.1002708 (PMC3475669; doi:10.1371/journal.pcbi.1002708)
Supplement: Software S1 — CAVER 3.0 package containing CAVER 3.0 executable, source code, license, documentation and examples. The latest release of CAVER 3.0 can be downloaded from http://www.caver.cz. (ZIP) [file pcbi.1002708.s006.zip › caver_3.0/source_code/caver/lib/junit/junit-3.8.2-api.zip!/junit/framework/ComparisonFailure.html]

ComparisonFailure (JUnit API)


|  |  |  |  |
| --- | --- | --- | --- |
| **Overview** \| **Package** \| **Class** \| **Tree** \| **Deprecated** \| **Index** \| **Help** | | |  |
| **PREV CLASS**   NEXT CLASS | **FRAMES**    **NO FRAMES**     **All Classes** |  |  |
| SUMMARY: NESTED \| FIELD \| CONSTR \| METHOD | DETAIL: FIELD \| CONSTR \| METHOD |  |  |


---


## junit.framework Class ComparisonFailure

```
java.lang.Object
  java.lang.Throwable
      java.lang.Error
          junit.framework.AssertionFailedError
              junit.framework.ComparisonFailure
```

**All Implemented Interfaces:**: java.io.Serializable

---

public class **ComparisonFailure** extends AssertionFailedError

Thrown when an assert equals for Strings failed.
Inspired by a patch from Alex Chaffee mailto:alex@purpletech.com

**See Also:**: Serialized Form

---

**Constructor Summary**

`ComparisonFailure(java.lang.String message,
java.lang.String expected,
java.lang.String actual)`
  
          Constructs a comparison failure.


|  |  |
| --- | --- |
| **Method Summary** | |
| `java.lang.String` | `getActual()`             Gets the actual string value |
| `java.lang.String` | `getExpected()`             Gets the expected string value |
| `java.lang.String` | `getMessage()`             Returns "..." in place of common prefix and "..." in place of common suffix between expected and actual. |

**Methods inherited from class java.lang.Throwable**

`fillInStackTrace, getCause, getLocalizedMessage, getStackTrace, initCause, printStackTrace, printStackTrace, printStackTrace, setStackTrace, toString`

**Methods inherited from class java.lang.Object**

`clone, equals, finalize, getClass, hashCode, notify, notifyAll, wait, wait, wait`

**Constructor Detail**

### ComparisonFailure

```
public ComparisonFailure(java.lang.String message,
                         java.lang.String expected,
                         java.lang.String actual)
```

:   Constructs a comparison failure.

    **Parameters:**: `message` - the identifying message or null: `expected` - the expected string value: `actual` - the actual string value


**Method Detail**

### getMessage

```
public java.lang.String getMessage()
```

:   Returns "..." in place of common prefix and "..." in
    place of common suffix between expected and actual.

    :   **See Also:**: `Throwable.getMessage()`

---


### getActual

```
public java.lang.String getActual()
```

:   Gets the actual string value

    :   **Returns:**: the actual string value

---


### getExpected

```
public java.lang.String getExpected()
```

:   Gets the expected string value

    :   **Returns:**: the expected string value


---


|  |  |  |  |
| --- | --- | --- | --- |
| **Overview** \| **Package** \| **Class** \| **Tree** \| **Deprecated** \| **Index** \| **Help** | | |  |
| **PREV CLASS**   NEXT CLASS | **FRAMES**    **NO FRAMES**     **All Classes** |  |  |
| SUMMARY: NESTED \| FIELD \| CONSTR \| METHOD | DETAIL: FIELD \| CONSTR \| METHOD |  |  |


---
