# Supplementary material for: CAVER 3.0: A Tool for the Analysis of Transport Pathways in Dynamic Protein Structures
Source: PLoS Comput Biol. 2012 Oct 18;8(10):e1002708. doi: 10.1371/journal.pcbi.1002708 (PMC3475669; doi:10.1371/journal.pcbi.1002708)
Supplement: Software S1 — CAVER 3.0 package containing CAVER 3.0 executable, source code, license, documentation and examples. The latest release of CAVER 3.0 can be downloaded from http://www.caver.cz. (ZIP) [file pcbi.1002708.s006.zip › caver_3.0/source_code/caver/lib/junit/junit-3.8.2-api.zip!/junit/framework/package-frame.html]

junit.framework (JUnit API)


junit.framework

Interfaces 
  
*Protectable*
  
*Test*
  
*TestListener*

Classes 
  
Assert
  
ComparisonCompactor
  
TestCase
  
TestFailure
  
TestResult
  
TestSuite

Errors 
  
AssertionFailedError
  
ComparisonFailure
