# Supplementary material for: CAVER 3.0: A Tool for the Analysis of Transport Pathways in Dynamic Protein Structures
Source: PLoS Comput Biol. 2012 Oct 18;8(10):e1002708. doi: 10.1371/journal.pcbi.1002708 (PMC3475669; doi:10.1371/journal.pcbi.1002708)
Supplement: Software S1 — CAVER 3.0 package containing CAVER 3.0 executable, source code, license, documentation and examples. The latest release of CAVER 3.0 can be downloaded from http://www.caver.cz. (ZIP) [file pcbi.1002708.s006.zip › caver_3.0/source_code/caver/lib/junit/junit-3.8.2-api.zip!/junit/framework/package-summary.html]

junit.framework (JUnit API)


|  |  |  |  |
| --- | --- | --- | --- |
| **Overview** \| **Package** \| Class \| **Tree** \| **Deprecated** \| **Index** \| **Help** | | |  |
| **PREV PACKAGE**   NEXT PACKAGE | **FRAMES**    **NO FRAMES**     **All Classes** |  |  |


---

## Package junit.framework

|  |  |
| --- | --- |
| **Interface Summary** | |
| **Protectable** | A *Protectable* can be run and can throw a Throwable. |
| **Test** | A *Test* can be run and collect its results. |
| **TestListener** | A Listener for test progress |

|  |  |
| --- | --- |
| **Class Summary** | |
| **Assert** | A set of assert methods. |
| **ComparisonCompactor** |  |
| **TestCase** | A test case defines the fixture to run multiple tests. |
| **TestFailure** | A `TestFailure` collects a failed test together with the caught exception. |
| **TestResult** | A `TestResult` collects the results of executing a test case. |
| **TestSuite** | A `TestSuite` is a `Composite` of Tests. |

|  |  |
| --- | --- |
| **Error Summary** | |
| **AssertionFailedError** | Thrown when an assertion failed. |
| **ComparisonFailure** | Thrown when an assert equals for Strings failed. |

---


|  |  |  |  |
| --- | --- | --- | --- |
| **Overview** \| **Package** \| Class \| **Tree** \| **Deprecated** \| **Index** \| **Help** | | |  |
| **PREV PACKAGE**   NEXT PACKAGE | **FRAMES**    **NO FRAMES**     **All Classes** |  |  |


---
