# Supplementary material for: CAVER 3.0: A Tool for the Analysis of Transport Pathways in Dynamic Protein Structures
Source: PLoS Comput Biol. 2012 Oct 18;8(10):e1002708. doi: 10.1371/journal.pcbi.1002708 (PMC3475669; doi:10.1371/journal.pcbi.1002708)
Supplement: Software S1 — CAVER 3.0 package containing CAVER 3.0 executable, source code, license, documentation and examples. The latest release of CAVER 3.0 can be downloaded from http://www.caver.cz. (ZIP) [file pcbi.1002708.s006.zip › caver_3.0/source_code/caver/lib/junit/junit-3.8.2-api.zip!/junit/framework/Test.html]

Test (JUnit API)


|  |  |  |  |
| --- | --- | --- | --- |
| **Overview** \| **Package** \| **Class** \| **Tree** \| **Deprecated** \| **Index** \| **Help** | | |  |
| **PREV CLASS**   **NEXT CLASS** | **FRAMES**    **NO FRAMES**     **All Classes** |  |  |
| SUMMARY: NESTED \| FIELD \| CONSTR \| METHOD | DETAIL: FIELD \| CONSTR \| METHOD |  |  |


---


## junit.framework Interface Test

**All Known Implementing Classes:**: TestCase, TestDecorator, TestSuite

---

public interface **Test**

A *Test* can be run and collect its results.

**See Also:**: `TestResult`

---

|  |  |
| --- | --- |
| **Method Summary** | |
| `int` | `countTestCases()`             Counts the number of test cases that will be run by this test. |
| `void` | `run(TestResult result)`             Runs a test and collects its result in a TestResult instance. |

**Method Detail**

### countTestCases

```
public int countTestCases()
```

:   Counts the number of test cases that will be run by this test.

---


### run

```
public void run(TestResult result)
```

:   Runs a test and collects its result in a TestResult instance.


---


|  |  |  |  |
| --- | --- | --- | --- |
| **Overview** \| **Package** \| **Class** \| **Tree** \| **Deprecated** \| **Index** \| **Help** | | |  |
| **PREV CLASS**   **NEXT CLASS** | **FRAMES**    **NO FRAMES**     **All Classes** |  |  |
| SUMMARY: NESTED \| FIELD \| CONSTR \| METHOD | DETAIL: FIELD \| CONSTR \| METHOD |  |  |


---
