# Supplementary material for: CAVER 3.0: A Tool for the Analysis of Transport Pathways in Dynamic Protein Structures
Source: PLoS Comput Biol. 2012 Oct 18;8(10):e1002708. doi: 10.1371/journal.pcbi.1002708 (PMC3475669; doi:10.1371/journal.pcbi.1002708)
Supplement: Software S1 — CAVER 3.0 package containing CAVER 3.0 executable, source code, license, documentation and examples. The latest release of CAVER 3.0 can be downloaded from http://www.caver.cz. (ZIP) [file pcbi.1002708.s006.zip › caver_3.0/source_code/caver/lib/junit/junit-3.8.2-api.zip!/junit/framework/TestCase.html]

TestCase (JUnit API)


|  |  |  |  |
| --- | --- | --- | --- |
| **Overview** \| **Package** \| **Class** \| **Tree** \| **Deprecated** \| **Index** \| **Help** | | |  |
| **PREV CLASS**   **NEXT CLASS** | **FRAMES**    **NO FRAMES**     **All Classes** |  |  |
| SUMMARY: NESTED \| FIELD \| CONSTR \| METHOD | DETAIL: FIELD \| CONSTR \| METHOD |  |  |


---


## junit.framework Class TestCase

```
java.lang.Object
  junit.framework.Assert
      junit.framework.TestCase
```

**All Implemented Interfaces:**: Test

**Direct Known Subclasses:**: ExceptionTestCase

---

public abstract class **TestCase** extends Assert implements Test

A test case defines the fixture to run multiple tests. To define a test case  
1) implement a subclass of TestCase  
2) define instance variables that store the state of the fixture  
3) initialize the fixture state by overriding `setUp`  
4) clean-up after a test by overriding `tearDown`.  
Each test runs in its own fixture so there
can be no side effects among test runs.
Here is an example:

```
 public class MathTest extends TestCase {
     protected double fValue1;
     protected double fValue2;

    protected void setUp() {
         fValue1= 2.0;
         fValue2= 3.0;
     }
 }
```

For each test implement a method which interacts
with the fixture. Verify the expected results with assertions specified
by calling `assertTrue` with a boolean.

```
    public void testAdd() {
        double result= fValue1 + fValue2;
        assertTrue(result == 5.0);
    }
```

Once the methods are defined you can run them. The framework supports
both a static type safe and more dynamic way to run a test.
In the static way you override the runTest method and define the method to
be invoked. A convenient way to do so is with an anonymous inner class.

```
 TestCase test= new MathTest("add") {
        public void runTest() {
            testAdd();
        }
 };
 test.run();
```

The dynamic way uses reflection to implement `runTest`. It dynamically finds
and invokes a method.
In this case the name of the test case has to correspond to the test method
to be run.

```
 TestCase test= new MathTest("testAdd");
 test.run();
```

The tests to be run can be collected into a TestSuite. JUnit provides
different *test runners* which can run a test suite and collect the results.
A test runner either expects a static method `suite` as the entry
point to get a test to run or it will extract the suite automatically.

```
 public static Test suite() {
      suite.addTest(new MathTest("testAdd"));
      suite.addTest(new MathTest("testDivideByZero"));
      return suite;
  }
```

**See Also:**: `TestResult`, `TestSuite`

---

**Constructor Summary**

`TestCase()`
  
          No-arg constructor to enable serialization.

`TestCase(java.lang.String name)`
  
          Constructs a test case with the given name.


|  |  |
| --- | --- |
| **Method Summary** | |
| `int` | `countTestCases()`             Counts the number of test cases executed by run(TestResult result). |
| `protected  TestResult` | `createResult()`             Creates a default TestResult object |
| `java.lang.String` | `getName()`             Gets the name of a TestCase |
| `TestResult` | `run()`             A convenience method to run this test, collecting the results with a default TestResult object. |
| `void` | `run(TestResult result)`             Runs the test case and collects the results in TestResult. |
| `void` | `runBare()`             Runs the bare test sequence. |
| `protected  void` | `runTest()`             Override to run the test and assert its state. |
| `void` | `setName(java.lang.String name)`             Sets the name of a TestCase |
| `protected  void` | `setUp()`             Sets up the fixture, for example, open a network connection. |
| `protected  void` | `tearDown()`             Tears down the fixture, for example, close a network connection. |
| `java.lang.String` | `toString()`             Returns a string representation of the test case |

**Methods inherited from class junit.framework.Assert**

`assertEquals, assertEquals, assertEquals, assertEquals, assertEquals, assertEquals, assertEquals, assertEquals, assertEquals, assertEquals, assertEquals, assertEquals, assertEquals, assertEquals, assertEquals, assertEquals, assertEquals, assertEquals, assertEquals, assertEquals, assertFalse, assertFalse, assertNotNull, assertNotNull, assertNotSame, assertNotSame, assertNull, assertNull, assertSame, assertSame, assertTrue, assertTrue, fail, fail, failNotEquals, failNotSame, failSame`

**Methods inherited from class java.lang.Object**

`clone, equals, finalize, getClass, hashCode, notify, notifyAll, wait, wait, wait`

**Constructor Detail**

### TestCase

```
public TestCase()
```

:   No-arg constructor to enable serialization. This method
    is not intended to be used by mere mortals without calling setName().

---


### TestCase

```
public TestCase(java.lang.String name)
```

:   Constructs a test case with the given name.


**Method Detail**

### countTestCases

```
public int countTestCases()
```

:   Counts the number of test cases executed by run(TestResult result).

    :   **Specified by:**: `countTestCases` in interface `Test`

---


### createResult

```
protected TestResult createResult()
```

:   Creates a default TestResult object

    :   **See Also:**: `TestResult`

---


### run

```
public TestResult run()
```

:   A convenience method to run this test, collecting the results with a
    default TestResult object.

    :   **See Also:**: `TestResult`

---


### run

```
public void run(TestResult result)
```

:   Runs the test case and collects the results in TestResult.

    :   **Specified by:**: `run` in interface `Test`

---


### runBare

```
public void runBare()
             throws java.lang.Throwable
```

:   Runs the bare test sequence.

    :   **Throws:**: `java.lang.Throwable` - if any exception is thrown

---


### runTest

```
protected void runTest()
                throws java.lang.Throwable
```

:   Override to run the test and assert its state.

    :   **Throws:**: `java.lang.Throwable` - if any exception is thrown

---


### setUp

```
protected void setUp()
              throws java.lang.Exception
```

:   Sets up the fixture, for example, open a network connection.
    This method is called before a test is executed.

    :   **Throws:**: `java.lang.Exception`

---


### tearDown

```
protected void tearDown()
                 throws java.lang.Exception
```

:   Tears down the fixture, for example, close a network connection.
    This method is called after a test is executed.

    :   **Throws:**: `java.lang.Exception`

---


### toString

```
public java.lang.String toString()
```

:   Returns a string representation of the test case

---


### getName

```
public java.lang.String getName()
```

:   Gets the name of a TestCase

    :   **Returns:**: returns a String

---


### setName

```
public void setName(java.lang.String name)
```

:   Sets the name of a TestCase

    :   **Parameters:**: `name` - The name to set


---


|  |  |  |  |
| --- | --- | --- | --- |
| **Overview** \| **Package** \| **Class** \| **Tree** \| **Deprecated** \| **Index** \| **Help** | | |  |
| **PREV CLASS**   **NEXT CLASS** | **FRAMES**    **NO FRAMES**     **All Classes** |  |  |
| SUMMARY: NESTED \| FIELD \| CONSTR \| METHOD | DETAIL: FIELD \| CONSTR \| METHOD |  |  |


---
