# Supplementary material for: CAVER 3.0: A Tool for the Analysis of Transport Pathways in Dynamic Protein Structures
Source: PLoS Comput Biol. 2012 Oct 18;8(10):e1002708. doi: 10.1371/journal.pcbi.1002708 (PMC3475669; doi:10.1371/journal.pcbi.1002708)
Supplement: Software S1 — CAVER 3.0 package containing CAVER 3.0 executable, source code, license, documentation and examples. The latest release of CAVER 3.0 can be downloaded from http://www.caver.cz. (ZIP) [file pcbi.1002708.s006.zip › caver_3.0/source_code/caver/lib/junit/junit-3.8.2-api.zip!/junit/framework/TestFailure.html]

TestFailure (JUnit API)


|  |  |  |  |
| --- | --- | --- | --- |
| **Overview** \| **Package** \| **Class** \| **Tree** \| **Deprecated** \| **Index** \| **Help** | | |  |
| **PREV CLASS**   **NEXT CLASS** | **FRAMES**    **NO FRAMES**     **All Classes** |  |  |
| SUMMARY: NESTED \| FIELD \| CONSTR \| METHOD | DETAIL: FIELD \| CONSTR \| METHOD |  |  |


---


## junit.framework Class TestFailure

```
java.lang.Object
  junit.framework.TestFailure
```

---

public class **TestFailure** extends java.lang.Object

A `TestFailure` collects a failed test together with
the caught exception.

**See Also:**: `TestResult`

---

|  |  |
| --- | --- |
| **Field Summary** | |
| `protected  Test` | `fFailedTest` |
| `protected  java.lang.Throwable` | `fThrownException` |


**Constructor Summary**

`TestFailure(Test failedTest,
java.lang.Throwable thrownException)`
  
          Constructs a TestFailure with the given test and exception.


|  |  |
| --- | --- |
| **Method Summary** | |
| `java.lang.String` | `exceptionMessage()` |
| `Test` | `failedTest()`             Gets the failed test. |
| `boolean` | `isFailure()` |
| `java.lang.Throwable` | `thrownException()`             Gets the thrown exception. |
| `java.lang.String` | `toString()`             Returns a short description of the failure. |
| `java.lang.String` | `trace()` |

**Methods inherited from class java.lang.Object**

`clone, equals, finalize, getClass, hashCode, notify, notifyAll, wait, wait, wait`

**Field Detail**

### fFailedTest

```
protected Test fFailedTest
```

---


### fThrownException

```
protected java.lang.Throwable fThrownException
```


**Constructor Detail**

### TestFailure

```
public TestFailure(Test failedTest,
                   java.lang.Throwable thrownException)
```

:   Constructs a TestFailure with the given test and exception.


**Method Detail**

### failedTest

```
public Test failedTest()
```

:   Gets the failed test.

---


### thrownException

```
public java.lang.Throwable thrownException()
```

:   Gets the thrown exception.

---


### toString

```
public java.lang.String toString()
```

:   Returns a short description of the failure.

---


### trace

```
public java.lang.String trace()
```

---


### exceptionMessage

```
public java.lang.String exceptionMessage()
```

---


### isFailure

```
public boolean isFailure()
```


---


|  |  |  |  |
| --- | --- | --- | --- |
| **Overview** \| **Package** \| **Class** \| **Tree** \| **Deprecated** \| **Index** \| **Help** | | |  |
| **PREV CLASS**   **NEXT CLASS** | **FRAMES**    **NO FRAMES**     **All Classes** |  |  |
| SUMMARY: NESTED \| FIELD \| CONSTR \| METHOD | DETAIL: FIELD \| CONSTR \| METHOD |  |  |


---
