# Supplementary material for: CAVER 3.0: A Tool for the Analysis of Transport Pathways in Dynamic Protein Structures
Source: PLoS Comput Biol. 2012 Oct 18;8(10):e1002708. doi: 10.1371/journal.pcbi.1002708 (PMC3475669; doi:10.1371/journal.pcbi.1002708)
Supplement: Software S1 — CAVER 3.0 package containing CAVER 3.0 executable, source code, license, documentation and examples. The latest release of CAVER 3.0 can be downloaded from http://www.caver.cz. (ZIP) [file pcbi.1002708.s006.zip › caver_3.0/source_code/caver/lib/junit/junit-3.8.2-api.zip!/junit/framework/TestListener.html]

TestListener (JUnit API)


|  |  |  |  |
| --- | --- | --- | --- |
| **Overview** \| **Package** \| **Class** \| **Tree** \| **Deprecated** \| **Index** \| **Help** | | |  |
| **PREV CLASS**   NEXT CLASS | **FRAMES**    **NO FRAMES**     **All Classes** |  |  |
| SUMMARY: NESTED \| FIELD \| CONSTR \| METHOD | DETAIL: FIELD \| CONSTR \| METHOD |  |  |


---


## junit.framework Interface TestListener

---

public interface **TestListener**

A Listener for test progress

---

|  |  |
| --- | --- |
| **Method Summary** | |
| `void` | `addError(Test test, java.lang.Throwable t)`             An error occurred. |
| `void` | `addFailure(Test test, AssertionFailedError t)`             A failure occurred. |
| `void` | `endTest(Test test)`             A test ended. |
| `void` | `startTest(Test test)`             A test started. |

**Method Detail**

### addError

```
public void addError(Test test,
                     java.lang.Throwable t)
```

:   An error occurred.

---


### addFailure

```
public void addFailure(Test test,
                       AssertionFailedError t)
```

:   A failure occurred.

---


### endTest

```
public void endTest(Test test)
```

:   A test ended.

---


### startTest

```
public void startTest(Test test)
```

:   A test started.


---


|  |  |  |  |
| --- | --- | --- | --- |
| **Overview** \| **Package** \| **Class** \| **Tree** \| **Deprecated** \| **Index** \| **Help** | | |  |
| **PREV CLASS**   NEXT CLASS | **FRAMES**    **NO FRAMES**     **All Classes** |  |  |
| SUMMARY: NESTED \| FIELD \| CONSTR \| METHOD | DETAIL: FIELD \| CONSTR \| METHOD |  |  |


---
