# Supplementary material for: CAVER 3.0: A Tool for the Analysis of Transport Pathways in Dynamic Protein Structures
Source: PLoS Comput Biol. 2012 Oct 18;8(10):e1002708. doi: 10.1371/journal.pcbi.1002708 (PMC3475669; doi:10.1371/journal.pcbi.1002708)
Supplement: Software S1 — CAVER 3.0 package containing CAVER 3.0 executable, source code, license, documentation and examples. The latest release of CAVER 3.0 can be downloaded from http://www.caver.cz. (ZIP) [file pcbi.1002708.s006.zip › caver_3.0/source_code/caver/lib/junit/junit-3.8.2-api.zip!/junit/framework/TestResult.html]

TestResult (JUnit API)


|  |  |  |  |
| --- | --- | --- | --- |
| **Overview** \| **Package** \| **Class** \| **Tree** \| **Deprecated** \| **Index** \| **Help** | | |  |
| **PREV CLASS**   **NEXT CLASS** | **FRAMES**    **NO FRAMES**     **All Classes** |  |  |
| SUMMARY: NESTED \| FIELD \| CONSTR \| METHOD | DETAIL: FIELD \| CONSTR \| METHOD |  |  |


---


## junit.framework Class TestResult

```
java.lang.Object
  junit.framework.TestResult
```

---

public class **TestResult** extends java.lang.Object

A `TestResult` collects the results of executing
a test case. It is an instance of the Collecting Parameter pattern.
The test framework distinguishes between *failures* and *errors*.
A failure is anticipated and checked for with assertions. Errors are
unanticipated problems like an `ArrayIndexOutOfBoundsException`.

**See Also:**: `Test`

---

|  |  |
| --- | --- |
| **Field Summary** | |
| `protected  java.util.Vector` | `fErrors` |
| `protected  java.util.Vector` | `fFailures` |
| `protected  java.util.Vector` | `fListeners` |
| `protected  int` | `fRunTests` |


**Constructor Summary**

`TestResult()`


|  |  |
| --- | --- |
| **Method Summary** | |
| `void` | `addError(Test test, java.lang.Throwable t)`             Adds an error to the list of errors. |
| `void` | `addFailure(Test test, AssertionFailedError t)`             Adds a failure to the list of failures. |
| `void` | `addListener(TestListener listener)`             Registers a TestListener |
| `void` | `endTest(Test test)`             Informs the result that a test was completed. |
| `int` | `errorCount()`             Gets the number of detected errors. |
| `java.util.Enumeration` | `errors()`             Returns an Enumeration for the errors |
| `int` | `failureCount()`             Gets the number of detected failures. |
| `java.util.Enumeration` | `failures()`             Returns an Enumeration for the failures |
| `void` | `removeListener(TestListener listener)`             Unregisters a TestListener |
| `protected  void` | `run(TestCase test)`             Runs a TestCase. |
| `int` | `runCount()`             Gets the number of run tests. |
| `void` | `runProtected(Test test, Protectable p)`             Runs a TestCase. |
| `boolean` | `shouldStop()`             Checks whether the test run should stop |
| `void` | `startTest(Test test)`             Informs the result that a test will be started. |
| `void` | `stop()`             Marks that the test run should stop. |
| `boolean` | `wasSuccessful()`             Returns whether the entire test was successful or not. |

**Methods inherited from class java.lang.Object**

`clone, equals, finalize, getClass, hashCode, notify, notifyAll, toString, wait, wait, wait`

**Field Detail**

### fFailures

```
protected java.util.Vector fFailures
```

---


### fErrors

```
protected java.util.Vector fErrors
```

---


### fListeners

```
protected java.util.Vector fListeners
```

---


### fRunTests

```
protected int fRunTests
```


**Constructor Detail**

### TestResult

```
public TestResult()
```


**Method Detail**

### addError

```
public void addError(Test test,
                     java.lang.Throwable t)
```

:   Adds an error to the list of errors. The passed in exception
    caused the error.

---


### addFailure

```
public void addFailure(Test test,
                       AssertionFailedError t)
```

:   Adds a failure to the list of failures. The passed in exception
    caused the failure.

---


### addListener

```
public void addListener(TestListener listener)
```

:   Registers a TestListener

---


### removeListener

```
public void removeListener(TestListener listener)
```

:   Unregisters a TestListener

---


### endTest

```
public void endTest(Test test)
```

:   Informs the result that a test was completed.

---


### errorCount

```
public int errorCount()
```

:   Gets the number of detected errors.

---


### errors

```
public java.util.Enumeration errors()
```

:   Returns an Enumeration for the errors

---


### failureCount

```
public int failureCount()
```

:   Gets the number of detected failures.

---


### failures

```
public java.util.Enumeration failures()
```

:   Returns an Enumeration for the failures

---


### run

```
protected void run(TestCase test)
```

:   Runs a TestCase.

---


### runCount

```
public int runCount()
```

:   Gets the number of run tests.

---


### runProtected

```
public void runProtected(Test test,
                         Protectable p)
```

:   Runs a TestCase.

---


### shouldStop

```
public boolean shouldStop()
```

:   Checks whether the test run should stop

---


### startTest

```
public void startTest(Test test)
```

:   Informs the result that a test will be started.

---


### stop

```
public void stop()
```

:   Marks that the test run should stop.

---


### wasSuccessful

```
public boolean wasSuccessful()
```

:   Returns whether the entire test was successful or not.


---


|  |  |  |  |
| --- | --- | --- | --- |
| **Overview** \| **Package** \| **Class** \| **Tree** \| **Deprecated** \| **Index** \| **Help** | | |  |
| **PREV CLASS**   **NEXT CLASS** | **FRAMES**    **NO FRAMES**     **All Classes** |  |  |
| SUMMARY: NESTED \| FIELD \| CONSTR \| METHOD | DETAIL: FIELD \| CONSTR \| METHOD |  |  |


---
