# Supplementary material for: CAVER 3.0: A Tool for the Analysis of Transport Pathways in Dynamic Protein Structures
Source: PLoS Comput Biol. 2012 Oct 18;8(10):e1002708. doi: 10.1371/journal.pcbi.1002708 (PMC3475669; doi:10.1371/journal.pcbi.1002708)
Supplement: Software S1 — CAVER 3.0 package containing CAVER 3.0 executable, source code, license, documentation and examples. The latest release of CAVER 3.0 can be downloaded from http://www.caver.cz. (ZIP) [file pcbi.1002708.s006.zip › caver_3.0/source_code/caver/lib/junit/junit-3.8.2-api.zip!/junit/framework/TestSuite.html]

TestSuite (JUnit API)


|  |  |  |  |
| --- | --- | --- | --- |
| **Overview** \| **Package** \| **Class** \| **Tree** \| **Deprecated** \| **Index** \| **Help** | | |  |
| **PREV CLASS**   NEXT CLASS | **FRAMES**    **NO FRAMES**     **All Classes** |  |  |
| SUMMARY: NESTED \| FIELD \| CONSTR \| METHOD | DETAIL: FIELD \| CONSTR \| METHOD |  |  |


---


## junit.framework Class TestSuite

```
java.lang.Object
  junit.framework.TestSuite
```

**All Implemented Interfaces:**: Test

**Direct Known Subclasses:**: ActiveTestSuite

---

public class **TestSuite** extends java.lang.Object implements Test

A `TestSuite` is a `Composite` of Tests.
It runs a collection of test cases. Here is an example using
the dynamic test definition.

```
 TestSuite suite= new TestSuite();
 suite.addTest(new MathTest("testAdd"));
 suite.addTest(new MathTest("testDivideByZero"));
```

Alternatively, a TestSuite can extract the tests to be run automatically.
To do so you pass the class of your TestCase class to the
TestSuite constructor.

```
 TestSuite suite= new TestSuite(MathTest.class);
```

This constructor creates a suite with all the methods
starting with "test" that take no arguments.

A final option is to do the same for a large array of test classes.

```
 Class[] testClasses = { MathTest.class, AnotherTest.class }
 TestSuite suite= new TestSuite(testClasses);
```

**See Also:**: `Test`

---

**Constructor Summary**

`TestSuite()`
  
          Constructs an empty TestSuite.

`TestSuite(java.lang.Class theClass)`
  
          Constructs a TestSuite from the given class.

`TestSuite(java.lang.Class[] classes)`
  
          Constructs a TestSuite from the given array of classes.

`TestSuite(java.lang.Class[] classes,
java.lang.String name)`
  
          Constructs a TestSuite from the given array of classes with the given name.

`TestSuite(java.lang.Class theClass,
java.lang.String name)`
  
          Constructs a TestSuite from the given class with the given name.

`TestSuite(java.lang.String name)`
  
          Constructs an empty TestSuite.


|  |  |
| --- | --- |
| **Method Summary** | |
| `void` | `addTest(Test test)`             Adds a test to the suite. |
| `void` | `addTestSuite(java.lang.Class testClass)`             Adds the tests from the given class to the suite |
| `int` | `countTestCases()`             Counts the number of test cases that will be run by this test. |
| `static Test` | `createTest(java.lang.Class theClass, java.lang.String name)`             ...as the moon sets over the early morning Merlin, Oregon mountains, our intrepid adventurers type... |
| `java.lang.String` | `getName()`             Returns the name of the suite. |
| `static java.lang.reflect.Constructor` | `getTestConstructor(java.lang.Class theClass)`             Gets a constructor which takes a single String as its argument or a no arg constructor. |
| `void` | `run(TestResult result)`             Runs the tests and collects their result in a TestResult. |
| `void` | `runTest(Test test, TestResult result)` |
| `void` | `setName(java.lang.String name)`             Sets the name of the suite. |
| `Test` | `testAt(int index)`             Returns the test at the given index |
| `int` | `testCount()`             Returns the number of tests in this suite |
| `java.util.Enumeration` | `tests()`             Returns the tests as an enumeration |
| `java.lang.String` | `toString()` |
| `static Test` | `warning(java.lang.String message)`             Returns a test which will fail and log a warning message. |

**Methods inherited from class java.lang.Object**

`clone, equals, finalize, getClass, hashCode, notify, notifyAll, wait, wait, wait`

**Constructor Detail**

### TestSuite

```
public TestSuite()
```

:   Constructs an empty TestSuite.

---


### TestSuite

```
public TestSuite(java.lang.Class theClass)
```

:   Constructs a TestSuite from the given class. Adds all the methods
    starting with "test" as test cases to the suite.
    Parts of this method was written at 2337 meters in the Hueffihuette,
    Kanton Uri

---


### TestSuite

```
public TestSuite(java.lang.Class theClass,
                 java.lang.String name)
```

:   Constructs a TestSuite from the given class with the given name.

    **See Also:**: `TestSuite(Class)`

---


### TestSuite

```
public TestSuite(java.lang.String name)
```

:   Constructs an empty TestSuite.

---


### TestSuite

```
public TestSuite(java.lang.Class[] classes)
```

:   Constructs a TestSuite from the given array of classes.

    **Parameters:**: `classes` -

---


### TestSuite

```
public TestSuite(java.lang.Class[] classes,
                 java.lang.String name)
```

:   Constructs a TestSuite from the given array of classes with the given name.

    **See Also:**: `TestSuite(Class[])`


**Method Detail**

### createTest

```
public static Test createTest(java.lang.Class theClass,
                              java.lang.String name)
```

:   ...as the moon sets over the early morning Merlin, Oregon
    mountains, our intrepid adventurers type...

---


### getTestConstructor

```
public static java.lang.reflect.Constructor getTestConstructor(java.lang.Class theClass)
                                                        throws java.lang.NoSuchMethodException
```

:   Gets a constructor which takes a single String as
    its argument or a no arg constructor.

    :   **Throws:**: `java.lang.NoSuchMethodException`

---


### warning

```
public static Test warning(java.lang.String message)
```

:   Returns a test which will fail and log a warning message.

---


### addTest

```
public void addTest(Test test)
```

:   Adds a test to the suite.

---


### addTestSuite

```
public void addTestSuite(java.lang.Class testClass)
```

:   Adds the tests from the given class to the suite

---


### countTestCases

```
public int countTestCases()
```

:   Counts the number of test cases that will be run by this test.

    :   **Specified by:**: `countTestCases` in interface `Test`

---


### getName

```
public java.lang.String getName()
```

:   Returns the name of the suite. Not all
    test suites have a name and this method
    can return null.

---


### run

```
public void run(TestResult result)
```

:   Runs the tests and collects their result in a TestResult.

    :   **Specified by:**: `run` in interface `Test`

---


### runTest

```
public void runTest(Test test,
                    TestResult result)
```

---


### setName

```
public void setName(java.lang.String name)
```

:   Sets the name of the suite.

    :   **Parameters:**: `name` - The name to set

---


### testAt

```
public Test testAt(int index)
```

:   Returns the test at the given index

---


### testCount

```
public int testCount()
```

:   Returns the number of tests in this suite

---


### tests

```
public java.util.Enumeration tests()
```

:   Returns the tests as an enumeration

---


### toString

```
public java.lang.String toString()
```


---


|  |  |  |  |
| --- | --- | --- | --- |
| **Overview** \| **Package** \| **Class** \| **Tree** \| **Deprecated** \| **Index** \| **Help** | | |  |
| **PREV CLASS**   NEXT CLASS | **FRAMES**    **NO FRAMES**     **All Classes** |  |  |
| SUMMARY: NESTED \| FIELD \| CONSTR \| METHOD | DETAIL: FIELD \| CONSTR \| METHOD |  |  |


---
