# Supplementary material for: CAVER 3.0: A Tool for the Analysis of Transport Pathways in Dynamic Protein Structures
Source: PLoS Comput Biol. 2012 Oct 18;8(10):e1002708. doi: 10.1371/journal.pcbi.1002708 (PMC3475669; doi:10.1371/journal.pcbi.1002708)
Supplement: Software S1 — CAVER 3.0 package containing CAVER 3.0 executable, source code, license, documentation and examples. The latest release of CAVER 3.0 can be downloaded from http://www.caver.cz. (ZIP) [file pcbi.1002708.s006.zip › caver_3.0/source_code/caver/lib/junit/junit-3.8.2-api.zip!/overview-tree.html]

Class Hierarchy (JUnit API)


|  |  |  |  |
| --- | --- | --- | --- |
| **Overview** \| Package \| Class \| **Tree** \| **Deprecated** \| **Index** \| **Help** | | |  |
| PREV   NEXT | **FRAMES**    **NO FRAMES**     **All Classes** |  |  |


---


## Hierarchy For All Packages

**Package Hierarchies:**: junit.extensions, junit.framework

---

## Class Hierarchy

- class java.lang.Object
  - class junit.framework.**Assert**
    - class junit.framework.**TestCase** (implements junit.framework.Test)
      - class junit.extensions.**ExceptionTestCase**
      - class junit.extensions.**TestDecorator** (implements junit.framework.Test)
        - class junit.extensions.**RepeatedTest**- class junit.extensions.**TestSetup**
    - class junit.framework.**ComparisonCompactor**- class junit.framework.**TestFailure**- class junit.framework.**TestResult**- class junit.framework.**TestSuite** (implements junit.framework.Test)
            - class junit.extensions.**ActiveTestSuite**
            - class java.lang.Throwable (implements java.io.Serializable)
              - class java.lang.Error
                - class junit.framework.**AssertionFailedError**
                  - class junit.framework.**ComparisonFailure**

## Interface Hierarchy

- interface junit.framework.**Protectable**- interface junit.framework.**Test**- interface junit.framework.**TestListener**

---


|  |  |  |  |
| --- | --- | --- | --- |
| **Overview** \| Package \| Class \| **Tree** \| **Deprecated** \| **Index** \| **Help** | | |  |
| PREV   NEXT | **FRAMES**    **NO FRAMES**     **All Classes** |  |  |


---
