# Supplementary material for: CAVER 3.0: A Tool for the Analysis of Transport Pathways in Dynamic Protein Structures
Source: PLoS Comput Biol. 2012 Oct 18;8(10):e1002708. doi: 10.1371/journal.pcbi.1002708 (PMC3475669; doi:10.1371/journal.pcbi.1002708)
Supplement: Software S1 — CAVER 3.0 package containing CAVER 3.0 executable, source code, license, documentation and examples. The latest release of CAVER 3.0 can be downloaded from http://www.caver.cz. (ZIP) [file pcbi.1002708.s006.zip › caver_3.0/source_code/caver/lib/junit/junit-3.8.2-api.zip!/serialized-form.html]

Serialized Form (JUnit API)


|  |  |  |  |
| --- | --- | --- | --- |
| **Overview** \| Package \| Class \| **Tree** \| **Deprecated** \| **Index** \| **Help** | | |  |
| PREV   NEXT | **FRAMES**    **NO FRAMES**     **All Classes** |  |  |


---


# Serialized Form


---

**Package** **junit.framework**

**Class junit.framework.AssertionFailedError extends java.lang.Error implements Serializable**

**serialVersionUID:** 1l

**Class junit.framework.ComparisonFailure extends AssertionFailedError implements Serializable**

**serialVersionUID:** 1l

**Serialized Fields**

### fExpected

```
java.lang.String fExpected
```

---

### fActual

```
java.lang.String fActual
```

---


|  |  |  |  |
| --- | --- | --- | --- |
| **Overview** \| Package \| Class \| **Tree** \| **Deprecated** \| **Index** \| **Help** | | |  |
| PREV   NEXT | **FRAMES**    **NO FRAMES**     **All Classes** |  |  |


---
