# Supplementary material for: CAVER 3.0: A Tool for the Analysis of Transport Pathways in Dynamic Protein Structures
Source: PLoS Comput Biol. 2012 Oct 18;8(10):e1002708. doi: 10.1371/journal.pcbi.1002708 (PMC3475669; doi:10.1371/journal.pcbi.1002708)
Supplement: Software S1 — CAVER 3.0 package containing CAVER 3.0 executable, source code, license, documentation and examples. The latest release of CAVER 3.0 can be downloaded from http://www.caver.cz. (ZIP) [file pcbi.1002708.s006.zip › caver_3.0/examples/guided_example/caver_guided_example.pdf]

CAVER 3.0 - GUIDED EXAMPLE

Analysis of tunnels in snapshots from MD simulation

This example will show you how to identify and analyze tunnels in snapshots from the MD simulation. For the demo purposes, you will analyze 50 snapshots from the 10 ns MD simulation of haloalkane dehalogenase DhaA. The input structures can be found in the examples/guided_example/md_snapshots directory. If you want to go through the whole analysis, go to the **examples/guided_example/inputs** directory. If you just want to explore the outputs of individual steps, go to the **examples/guided_example/results** directory and analyze the pre-calculated results. Important: If you want to exactly reproduce the results of previous analyses, you have to use the exactly same input files, with the same file names and content (the same number and order of atoms), as well as the same setting of the *seed* parameter.

1. Calculation settings

Go to the examples/guided_example/inputs directory. Open the config.txt file and check the settings of parameters. If you plan to visualize the results in VMD, you should set properly the *path_to_vmd* parameter (Advanced settings).

2. Running CAVER 3.0

Important: To ensure smooth calculation, please check that you have enough operating memory to allocate specified heap space and still maintain memory requirements of your system.

Launch the caver.bat file or type the following command in the command prompt (make sure that you are running the command from the examples/guided_example/inputs directory):
java -Xmx1200m -cp ../../../caver/lib -jar ../../../caver/caver.jar -home ../../../caver -pdb ../md_snapshots -conf ./config.txt -out ./out

Important: If you are getting the "*java is not recognized as an internal or external command, operable program or batch file*" error, try to specify the full path to java executable in the running command (or caver.bat file), e.g., "C:\Program Files\Java\jre1.6.0_06\bin\java" -Xmx1200m ... Alternatively, you may add java to the Path in your Environment Variables.
Important: If you are getting the "*Error occurred during initialization of VM. Could not reserve enough space for object heap. Could not create the Java virtual machine.*" error, you should decrease Java heap size or use 64bit Java.

In the command prompt, you will see the settings used for the identification of tunnels and the calculation progress:

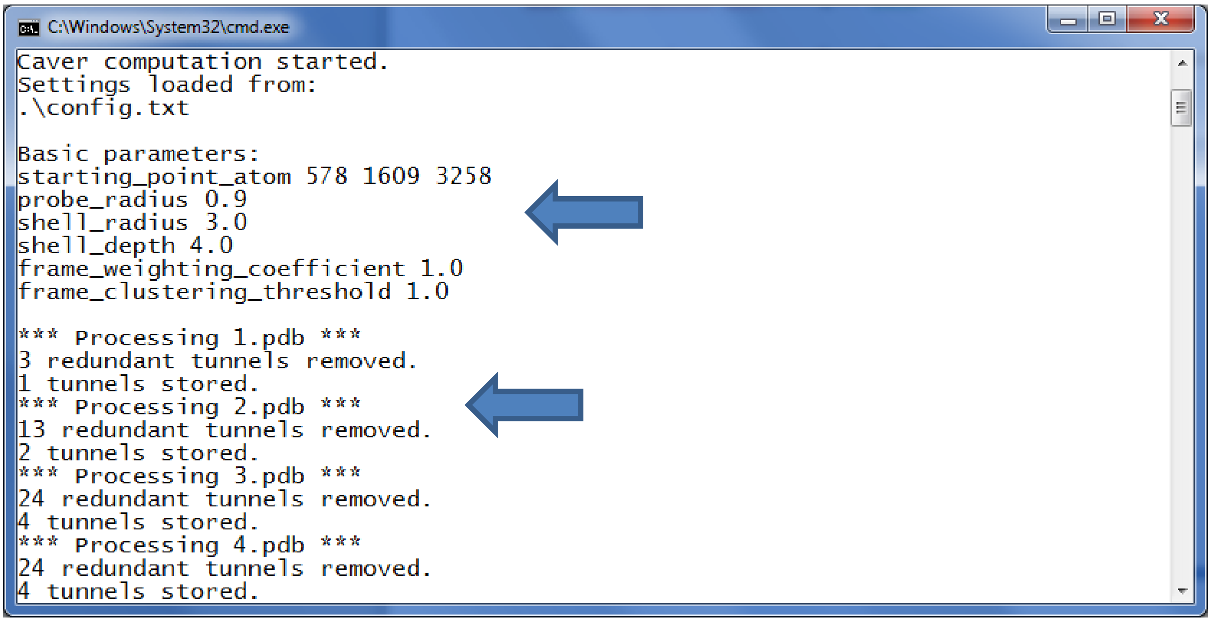

After the tunnels are identified in all analyzed snapshots, they are loaded for clustering:

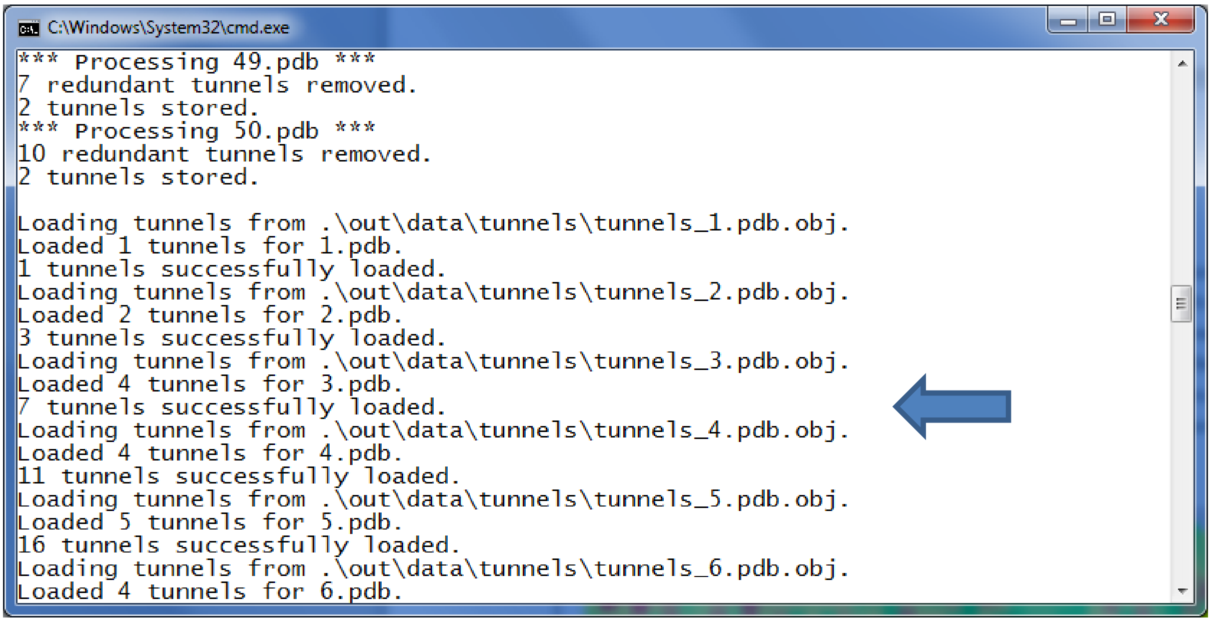

The clustering step involves the calculation of average surface of tunnels, computation of pairwise tunnel distances and average-link hierarchical clustering:

```
C:\Windows\System32\cmd.exe
Loaded 6 tunnels for 45.pdb.
136 tunnels successfully loaded.
Loading tunnels from .\out\data\tunnels\tunnels_46.pdb.obj.
Loaded 1 tunnels for 46.pdb.
137 tunnels successfully loaded.
Loading tunnels from .\out\data\tunnels\tunnels_47.pdb.obj.
Loaded 1 tunnels for 47.pdb.
138 tunnels successfully loaded.
Loading tunnels from .\out\data\tunnels\tunnels_48.pdb.obj.
Loaded 2 tunnels for 48.pdb.
140 tunnels successfully loaded.
Loading tunnels from .\out\data\tunnels\tunnels_49.pdb.obj.
Loaded 2 tunnels for 49.pdb.
142 tunnels successfully loaded.
Loading tunnels from .\out\data\tunnels\tunnels_50.pdb.obj.
Loaded 2 tunnels for 50.pdb.
144 tunnels successfully loaded.

Computing average surface for 144 tunnels.
Average surface represented by 27 points.
Computing distance matrix using weighting_coefficient 1.0.
Performing average link hierarchical clustering to create .\out\data\tree.txt.
144 tunnels clustered into 11 clusters using clustering_threshold 2.0.
```

Once clustering is finished, the output files specified in the config.txt (the summary information, tunnel characteristics and tunnel profiles) are generated. The results are stored in the out directory:

```
C:\Windows\System32\cmd.exe
140 tunnels successfully loaded.
Loading tunnels from .\out\data\tunnels\tunnels_49.pdb.obj.
Loaded 2 tunnels for 49.pdb.
142 tunnels successfully loaded.
Loading tunnels from .\out\data\tunnels\tunnels_50.pdb.obj.
Loaded 2 tunnels for 50.pdb.
144 tunnels successfully loaded.

Computing average surface for 144 tunnels.
Average surface represented by 27 points.
Computing distance matrix using weighting_coefficient 1.0.
Performing average link hierarchical clustering to create .\out\data\tree.txt.
144 tunnels clustered into 11 clusters using clustering_threshold 2.0.

Statistical analysis of results started.
Saving summary information.
Saving tunnel characteristics.
Saving tunnel profiles.
Statistical analysis finished.
Visualizations started.
Cluster subsampling for visualization: random.
Visualizations finished.

Finished successfully.
```

Tip: Try to increase the maximum heap size if you encounter "out of memory" error during the calculation of tunnels. If you want to use heap size larger than 1200m, you will probably need to use 64bit java. Alternatively, you may try to solve the "out of memory" error by setting *number_of_approximating_balls* parameter to a lower value.

3. Analysis of clustering results

First, you should evaluate clustering results. Provided scripts enable you to open the results all at once in PyMOL or VMD. For PyMOL, go to the out/pymol directory. If you have "py" extension associated with PyMOL, you can launch the results by clicking the view_timeless.py file. Alternatively, you can open the results via the PyMOL menu (File > Run > *Path_to_pymol_dir*/view_timeless.py) or PyMOL command line ("run *Path_to_pymol_dir*/view_timeless.py"):

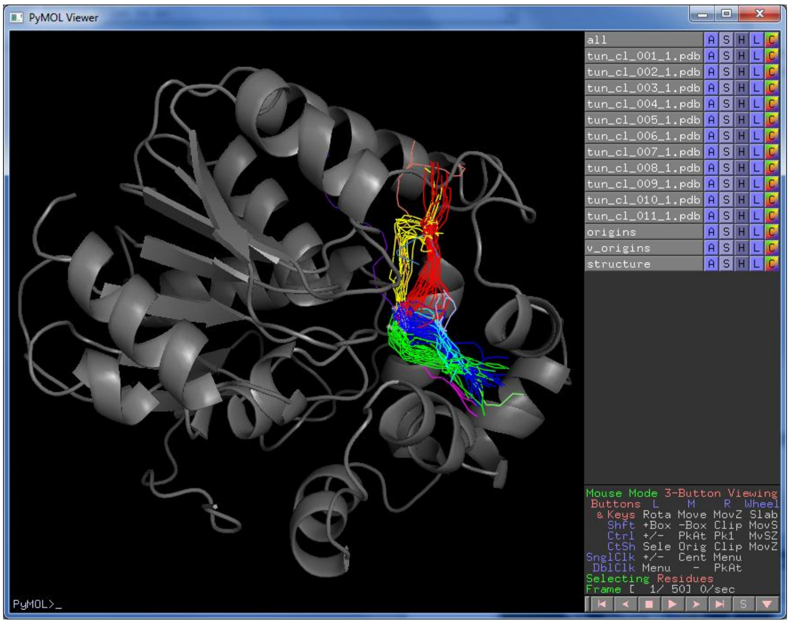

For VMD, go to the out/vmd directory and launch the vmd_timeless.bat script (make sure that the path to the VMD software is correctly specified in the script). Alternatively, you can open the results using the VMD Tk console (Extensions > Tk Console) by typing "cd *Path_to_vmd_dir*" and then "source scripts/view_timeless.tcl"

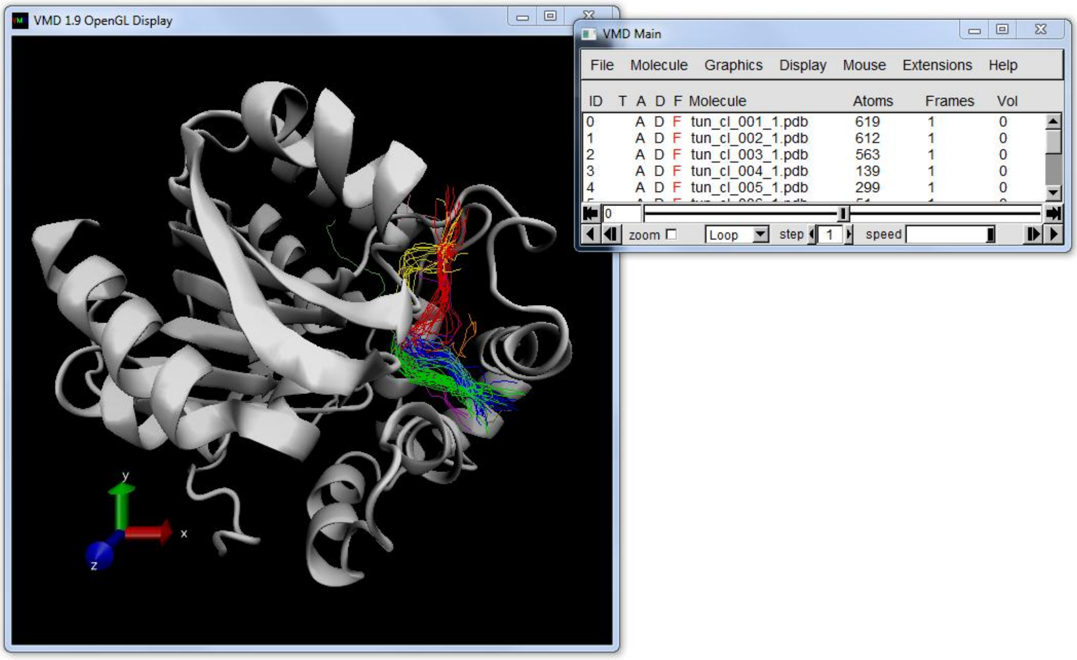

Tunnels identified throughout the MD simulation are all visualized in one frame as tunnel centerlines. Individual tunnel clusters are colored in different colors (the colors can be changed using the standard procedure of a given visualization software). Each tunnel cluster corresponds to one object (molecule); the name of each object includes the ID of a respective tunnel cluster. Important: For large datasets, one cluster may be divided into two or more objects.

Because a low *clustering_threshold* (= 2) was used for clustering, quite similar tunnels are divided into different clusters:

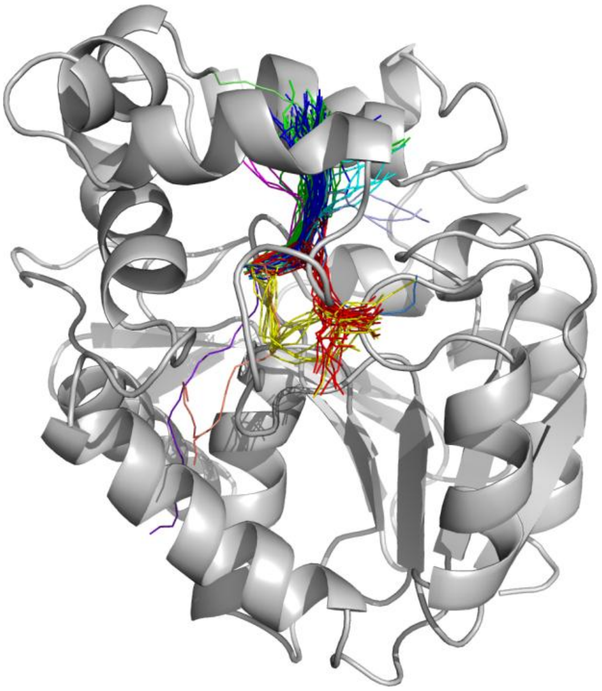

For example, the cluster 1 (dark blue), cluster 2 (green), cluster 4 (cyan), cluster 7 (violet in PyMOL, orange in VMD) and cluster 8 (bright green in PyMOL, olive in VMD) all represent very similar tunnels and should be clustered together. To improve the clustering results, you should increase the *clustering_threshold*.

Important: Using the default settings, the data for visualization of tunnel dynamics are not saved (i.e., the out/pymol/view.py and out/vmd/vmd.bat scripts will not work). If you want to analyze tunnel dynamics, open the config.txt file, set the *save_dynamics_visualization*, *load_tunnels* and *load_cluster_tree* parameters to *yes* and launch the caver.bat (or run the CAVER from the command line).

4. Adjusting clustering_threshold

Open the config.txt file and set the *clustering_threshold* to 4.0. It is not necessary to recalculate the tunnels or repeat the clustering procedure to adjust the clustering results. Therefore, set both the *load_tunnels* and *load_cluster_tree* parameters to *yes* and launch the caver.bat (or run the CAVER from the command line).

You can see in the command prompt that the tunnels are not being identified *de novo,* but loaded from the disk. Similarly, the hierarchy of tunnel clusters is loaded from the out/data/tree.txt file, which was obtained in previous analysis:

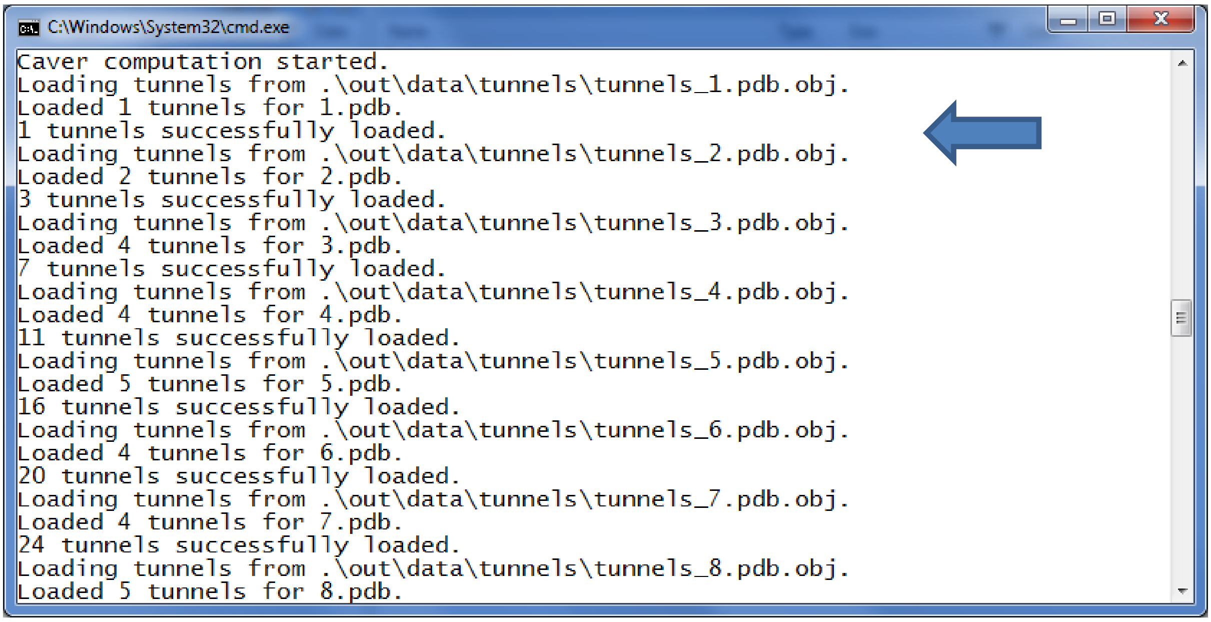

The loaded cluster hierarchy will be cut at the 4.0 threshold, and the new output files, reflecting the new composition of tunnel clusters, will be generated:

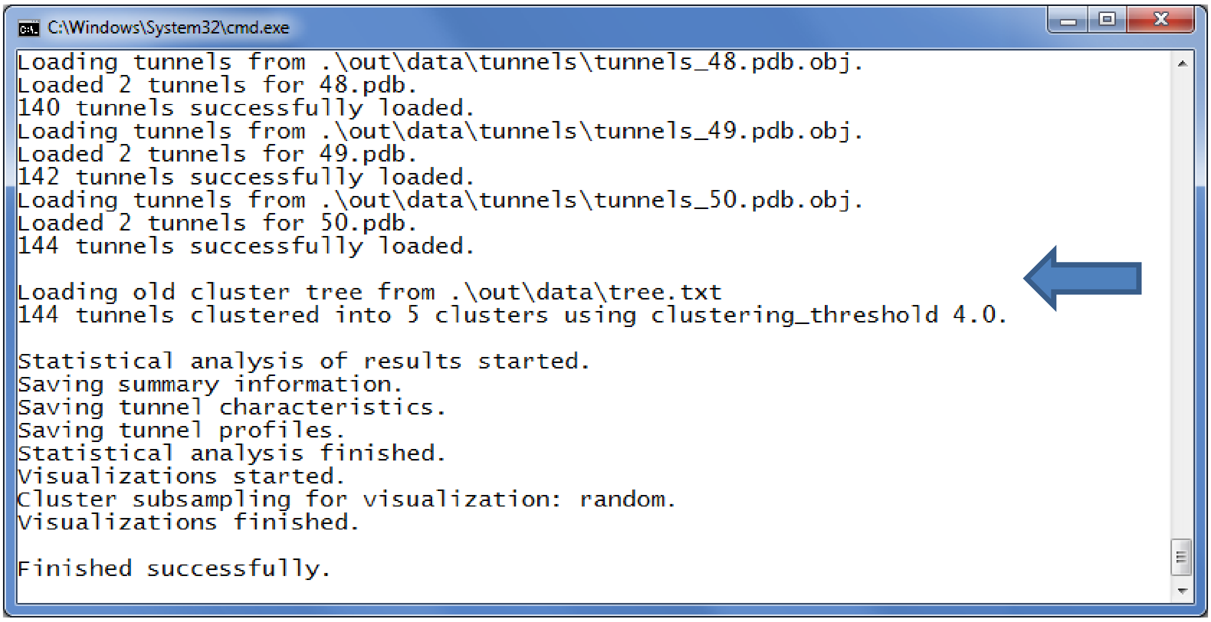

Launch the out/pymol/view_timeless.py or out/vmd/vmd_timeless.bat scripts to visualize the new results in PyMOL or VMD, respectively. You can see that several previously separated clusters are now joined together.

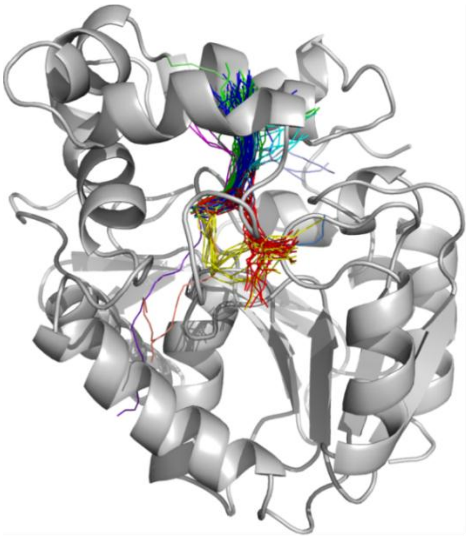

clustering_threshold 2.0

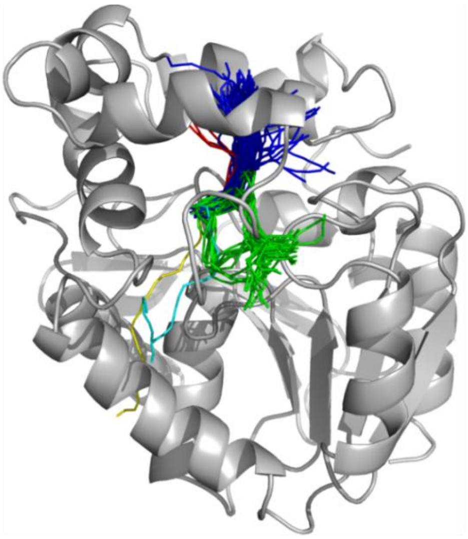

clustering_threshold 4.0

If you are still not satisfied with the clustering results, you can further continue with optimizing the *clustering_threshold* parameter. For example, the tunnels from the cluster 2 (green) have a common opening to the bulk solvent, and in fact represent alternative connections between this opening and the active site. In this sense, these two tunnels can be considered as two variants of one pathway. If we wanted to analyze the relative importance of these two pathways to each other, we would have to set the *clustering_threshold* to a lower value (e.g., 3.0), resulting in the subdivision of the green cluster into two clusters. In this demo, we will further analyze the results obtained using the *clustering_threshold* of 4.0.

Important: Using the default settings, the data for visualization of tunnel dynamics are not saved (i.e., the out/pymol/view.py and out/vmd/vmd.bat scripts will not work). If you want to analyze tunnel dynamics, open the config.txt file, set the *save_dynamics_visualization* to *yes* and launch the caver.bat (or run the CAVER from the command line).

5. Generation of a complete set of output files

As soon as you are satisfied with the clustering results, you can generate all output files, including the files with higher demands on memory and computation time that were not generated during the optimization procedure. Open the config.txt file, go to the “Generation of outputs” section and set the following parameters:

- *one_tunnel_in_snapshot* cheapest
- save_dynamics_visualization yes
- *generate_histograms* yes
- *generate_bottleneck_heat_map* yes
- *generate_profile_heat_map* yes
- *compute_tunnel_residues* yes
- *compute_bottleneck_residues* yes

Then move to the “Advanced settings” and change the following parameters:

- *compute_errors* yes
- *save_error_profiles* yes
- *generate_trajectory* yes

Make sure, that both the *load_tunnels* and *load_cluster_tree* parameters are set to *yes* and then launch the caver.bat file (or run the CAVER from the command line). You may notice on the screen that besides the summary information, tunnel characteristics and profiles, additional output files are generated. These “new” outputs include the information about tunnel-lining residues and atoms, tunnel bottlenecks, heat maps and histograms. The most demanding step is the calculation of maximum possible errors caused by the approximation of the input structure’s atoms by a set of balls. All output files are stored in the out directory.

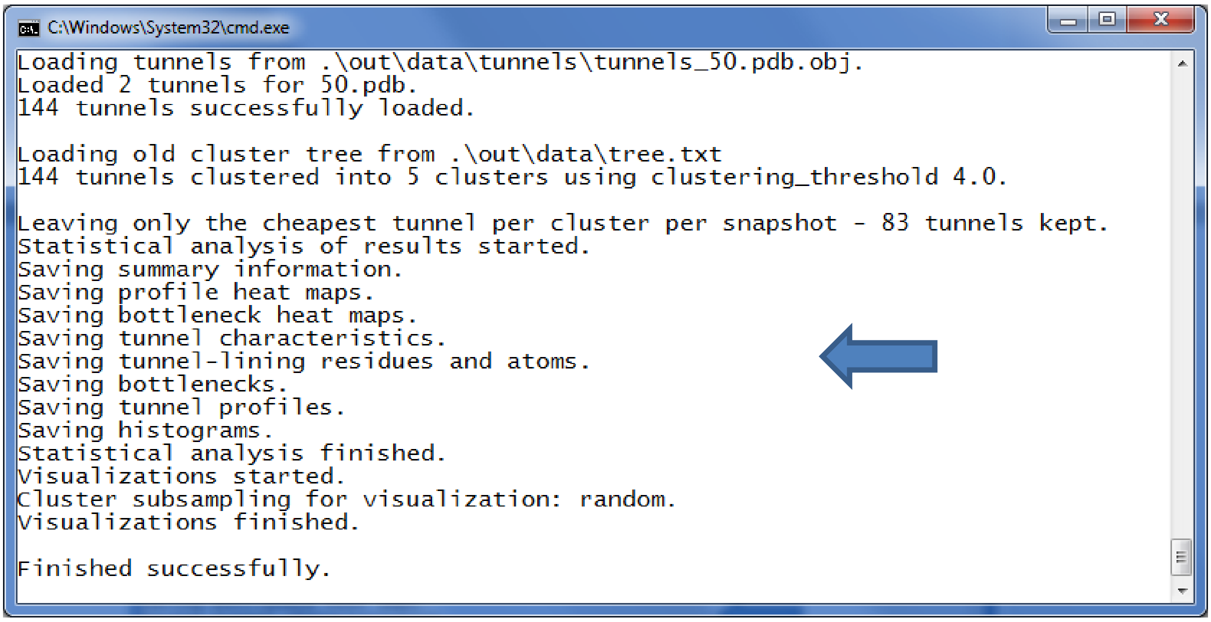

6. Analysis of identified tunnels

Overview of identified tunnels

Launch the out/pymol/view_timeless.py or out/vmd/vmd_timeless.bat scripts to visualize the final tunnel clusters, and explore the variability of identified tunnels in PyMOL or VMD, respectively. You may notice the difference between the clusters you have obtained now and the clusters obtained during the optimization steps (see 4. Adjusting clustering_threshold)—some of the previously visible tunnels are not visible now. The difference is caused by the fact that earlier you visualized all identified tunnels (this option is recommended for the evaluation of clustering results), while now only the best (i.e., cheapest) tunnel from each tunnel cluster is reported in individual snapshots. This option (*one_tunnel_in_snapshot* cheapest) is recommended for the interpretation of data. (If you consider also other “non-cheapest” pathways as relevant for the purpose of your study, you should separate them from the dominant tunnels by decreasing the *clustering_threshold* and analyze them as a separate tunnel cluster).

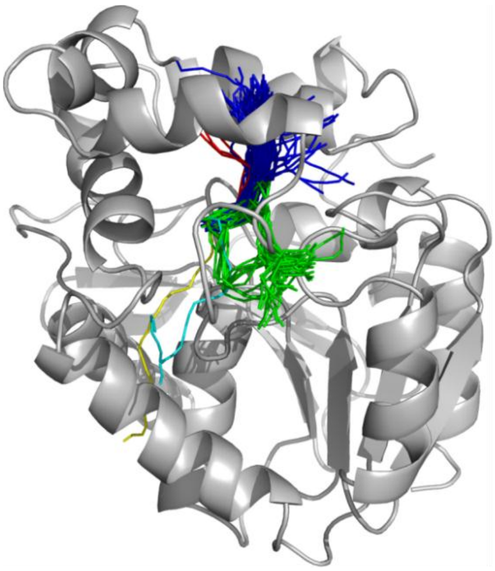

one_tunnel_in_snapshot no

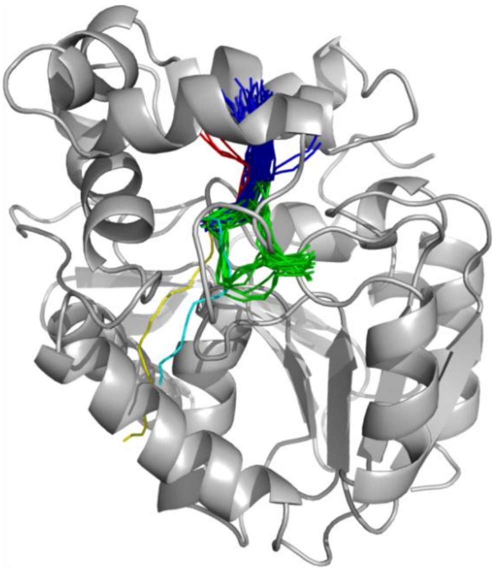

one_tunnel_in_snapshot cheapest

You can also visually analyze tunnel dynamics. For that, launch the out/vmd/vmd.bat script, which will open the MD trajectory together with identified tunnels in VMD. Short trajectories can also be visualized in PyMOL using either the out/pymol/view_trajectory.py script (opens both the trajectory and dynamic tunnels) or out/pymol/view.py script (opens dynamic tunnels but only one static structure). Warning: Depending on your system, opening of large data may cause PyMOL to crash).

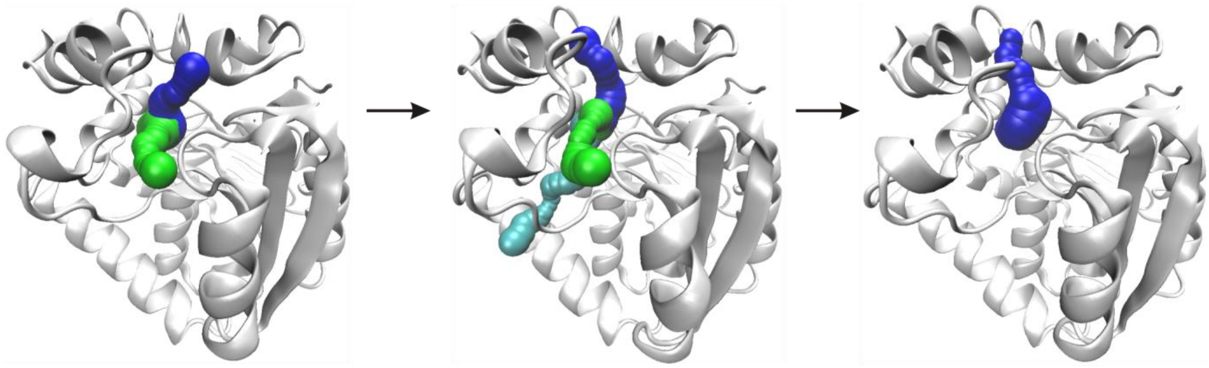

snapshot 44 snapshot 45 snapshot 46

Comparison of characteristics of individual tunnel clusters

Open the out/summary.txt file and explore the summary characteristics of all identified tunnel clusters:

ID	No	No_snaps	Avg_BR	SD	Max_BR	Avg_L	SD	Avg_C	SD
1	50	50	1.414	0.245	2.33	13.978	1.618	1.308	0.109
2	28	28	1.066	0.124	1.48	16.896	1.671	1.328	0.102
3	3	3	0.934	0.019	0.95	15.650	1.502	1.488	0.153
4	1	1	0.926	0.000	0.93	19.944	0.000	1.259	0.000
5	1	1	0.905	0.000	0.91	23.930	0.000	1.330	0.000

The tunnels from the first ranked tunnel cluster (cluster 1) were identified in all 50 snapshots, which means that they have the bottleneck radius equal or larger than the used *probe_radius* of 0.9 Å. The tunnels from the second best-ranked cluster were identified in approximately half of the analyzed snapshots, while the tunnels from remaining three clusters were rarely identified. The Avg_BR column shows that the first cluster has the highest average bottleneck radius. Note that the values of average bottleneck are overestimated for the clusters 2–5 since averages are calculated only over snapshots where some tunnel from a given cluster was identified. The average values should be therefore interpreted with caution. The Max_BR column shows the maximum opening of the tunnel bottleneck throughout the entire MD simulation. The values indicate that both the cluster 1 and cluster 2 were open enough to enable passage of water molecules (considering the usual probe radius of water molecule of 1.4 Å). The Avg_C column suggests that the tunnel cluster 4 is the straightest from the identified clusters, however this should be again interpreted with caution since this cluster is represented by only one tunnel (more snapshots would need to be analyzed to provide more reliable data). The summary.txt file further provides priorities and average throughputs of individual tunnel clusters, as well as the information about the maximal approximation errors (i.e., the maximum overestimation of tunnel radii caused by the approximation of input structure).

Analysis of tunnel-lining atoms and residues

To get an overview of residues and atoms making up individual tunnel clusters, open the out/analysis/residues.txt file. For each tunnel cluster, all residues and atoms that were at least in one snapshot found within the 3 Å distance from some ball of a given tunnel cluster are reported:

```
== Tunnel cluster 1 ==
# C    res  AA      N   sideN atoms
  X     38 ASN     50      50 50:H_570 50:CA_571 50:CB_573 50:HB3_575 50:CG_
  X    103 ASP     50      50 50:CA_1603 50:CB_1605 50:HB2_1606 50:CG_1608 5
  X    104 TRP     50      50 50:CD1_1621 50:HD1_1622 50:NE1_1623 50:HE1_162
  X    129 ILE     50      50 50:CD1_2025 41:HD13_2028 40:HD11_2026 40:HD12_
  X    138 TRP     50      50 50:HH2_2183 49:HZ2_2181 49:CH2_2182 48:CZ2_218
  X    141 PHE     50      50 50:O_2239 49:C_2238 45:CE2_2234 45:CD2_2236 44
  X    142 ALA     50      50 50:N_2240 50:CA_2242 50:HA_2243 50:CB_2244 50:
  X    146 PHE     50      50 50:CG_2310 50:CD1_2311 50:HD1_2312 50:CE1_2313
  X    165 PHE     50      50 50:CD1_2610 50:CE1_2612 50:HE1_2613 50:CZ_2614
  X    169 ALA     50      50 50:HB1_2668 50:O_2672 49:CB_2667 48:N_2663 48:
  X    172 LYS     50      50 50:CB_2710 50:HB3_2712 50:CD_2716 50:C_2726 49
  X    173 CYS     50      50 50:CA_2730 50:CB_2732 50:HB2_2733 50:HB3_2734
  X    202 PHE     50      50 50:HB3_3237 49:CB_3235 47:C_3249 43:O_3250 38:
  X    203 PRO     50      50 48:CD_3252 48:HD3_3254 48:HA_3262 47:N_3251 47
  X    206 LEU     50      50 49:CD1_3303 49:HD11_3304 49:CD2_3307 48:CG_330
  X    242 VAL     50      50 50:CG1_3857 47:HG13_3860 46:HG11_3858 46:HG12_
  X    243 LEU     50      50 44:CD1_3876 44:CD2_3880 41:CG_3874 40:HD11_387
  X    269 HID     50      50 50:CB_4249 50:HB2_4250 50:HB3_4251 50:CG_4252
  X    270 TYR     50      50 50:OH_4275 50:HH_4276 49:CE1_4272 49:HE1_4273
  X    145 THR     49      49 49:HB_2294 49:CG2_2295 48:CB_2293 48:HG23_2298
  [illegible] 149 PHE     49      49 49:HE2_2365 48:CE2_2364 46:CZ_2362 46:HZ_2363
  X    168 GLY     47      34 47:O_2662 46:C_2661 38:CA_2658 34:HA3_2660 21:
```

For example, Gly168 (chain X) was identified as the tunnel-lining residue of the tunnel cluster 1 in 47 snapshots, and the side-chain of this residue was lining the tunnel in 34 snapshots. For each residue, the tunnel-lining atoms are also listed, e.g., the CA atom (atom number 2658) of residue Gly168 was identified as tunnel-lining atom in 38 snapshots.

Time evolution of tunnels

You can further analyze the time evolution of characteristics of individual tunnel clusters. The distributions of bottleneck radii and throughputs of individual tunnel clusters are provided in the out/analysis/histograms directory (bottleneck_histograms.csv and throughput_histograms.csv files).

The out/analysis/bottleneck_heat_maps/bottleneck_heat_map.png file provides a graphical overview of bottleneck radii of individual tunnel clusters:

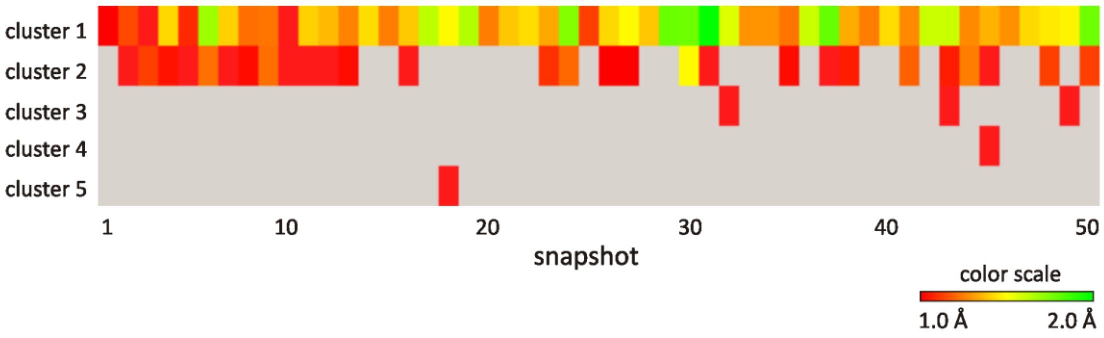

The bottleneck heat map clearly shows that the tunnel cluster 1 is a dominant pathway—it was identified in all snapshots, has the widest bottlenecks and was relatively frequently open to water molecules (i.e., bottleneck radius > 1.4, indicated by yellow and green colors). It also shows that only one of the 28 identified tunnels from the cluster 2 was open to water molecules:

Information about characteristics of each individual tunnel identified throughout the MD simulation is provided in the out/analysis/tunnel_characteristics.csv file. The data in this file are sorted by cluster IDs, so that you can directly plot the graphs showing the time evolution of selected characteristics of individual tunnel clusters. Important: Before you plot graphs, make sure that for each analyzed snapshot, the data are only reported for one tunnel from each tunnel cluster. This is controlled by the "*one_tunnel_in_snapshot* cheapest" option.

Use any spreadsheet editor to open the out/analysis/tunnel_characteristics.csv file and plot the time evolution of bottleneck radius of the cluster 1:

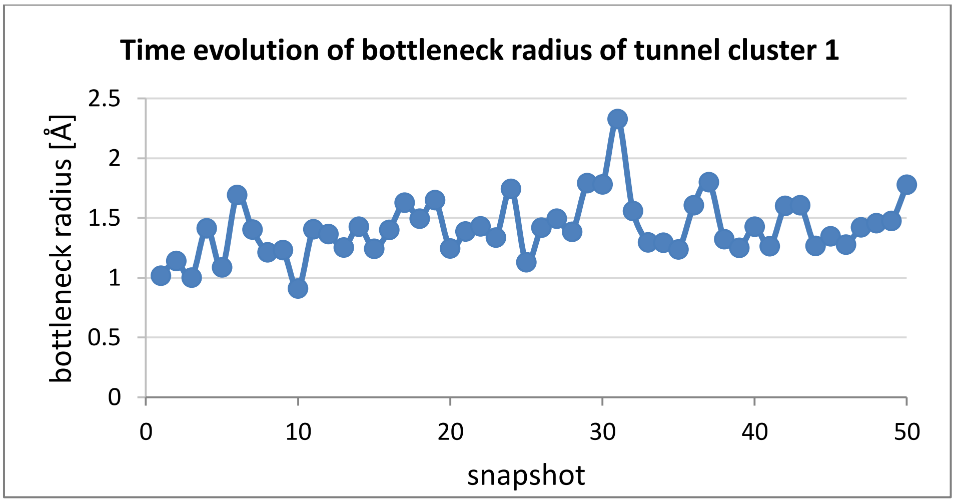

The bottleneck radius of this tunnel cluster ranges from 0.9 Å to 2.3 Å. The largest bottleneck radius from all tunnels from the cluster 1 had the tunnel identified in the snapshot 31, while the tunnel identified in the snapshot 10 had the smallest bottleneck. You can plot and compare the profiles (i.e., tunnel radius over the distance from the starting point measured along the tunnel axis) of these two tunnels using the data provided in the out/analysis/tunnel_profiles.csv file:

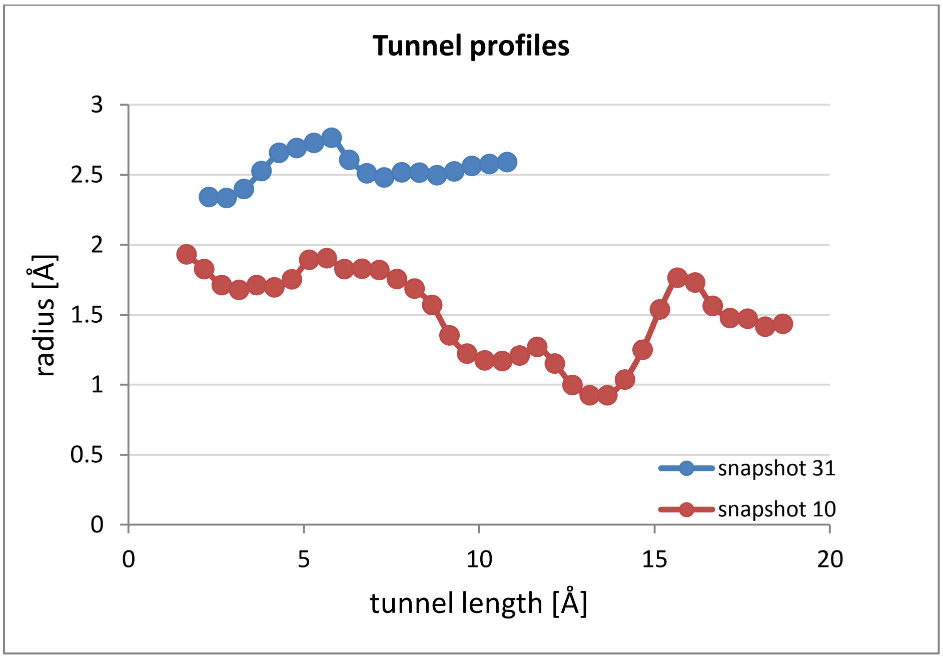

The comparison of both profiles shows significant differences between both tunnels in terms of their radii and length. The tunnel from the snapshot 31 is wide open along its entire length and has no clear bottleneck. The short length of this tunnel suggests that the tunnel will most likely be straight and/or wide open to the bulk solvent. The time evolution of the profiles of individual tunnel clusters can be also analyzed using the heat map visualizations provided in the out/analysis/profile_heat_maps/ directory. To see the profile heat map of the first tunnel cluster, open the cl_000001_profile_heat_map.png file:

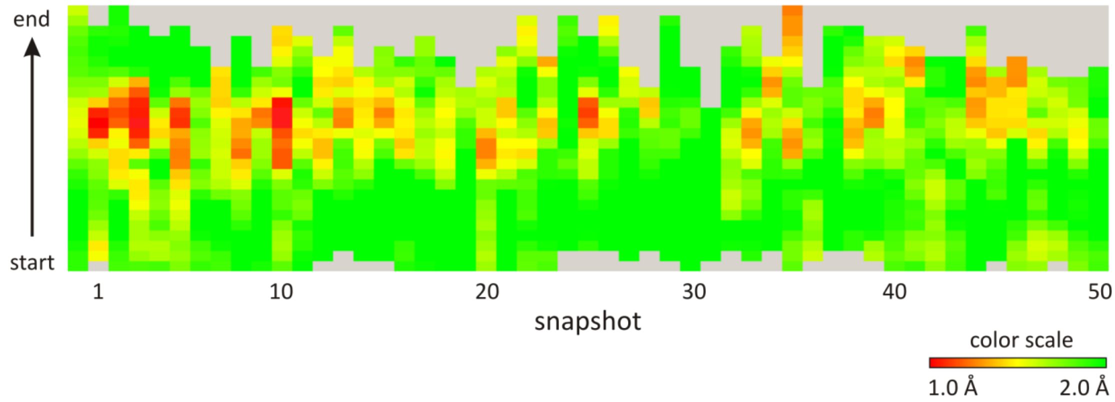

As you can see, the tunnel from the snapshot 31 is green (i.e. wide) along its entire length, while the tunnel from the snapshot 10 has a quite long narrow segment (red color). You may also notice that most of the tunnels have the bottleneck located approximately at 2/3 of the tunnel length.

Analysis of bottlenecks

We will further investigate the differences between the tunnels from snapshot 10 and 31 in the visualization software. Launch the vmd.bat script and go to given snapshots of MD (note that in VMD, the snapshots are numbered from 0; the tunnels from snapshot 10 and 31 thus correspond to the frames 9 and 30, respectively). You will see that both tunnels have very different geometry.

Furthermore, it is often useful to identify and highlight the residues making up the bottlenecks of the tunnels. As was mentioned above, there is no clear bottleneck in the tunnel from snapshot 31, while the tunnel from snapshot 10 has quite long narrow segment in approximately 2/3 of its length. Open the out/analysis/bottlenecks.csv file and find the bottleneck residues of the tunnel from the snapshot 10 (note that all residues located within 3 Å distance from the bottleneck are reported; in most cases, you can consider the three closest resides as the bottleneck residues). You will find that the three closest residues to the bottleneck are residues 142, 146 and 173 (all belonging to the chain X). Try to highlight the residue in VMD and compare their locations in snapshot 10 and 31. You may notice the differences in location of helices carrying the bottleneck residues as well as the change of orientation of Cys173 and Phe146:

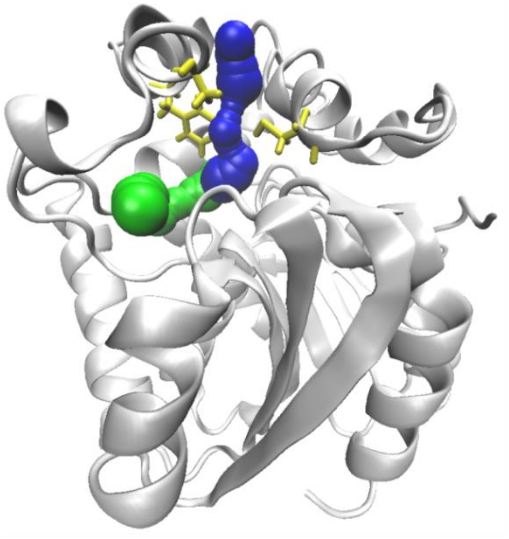

snapshot 10

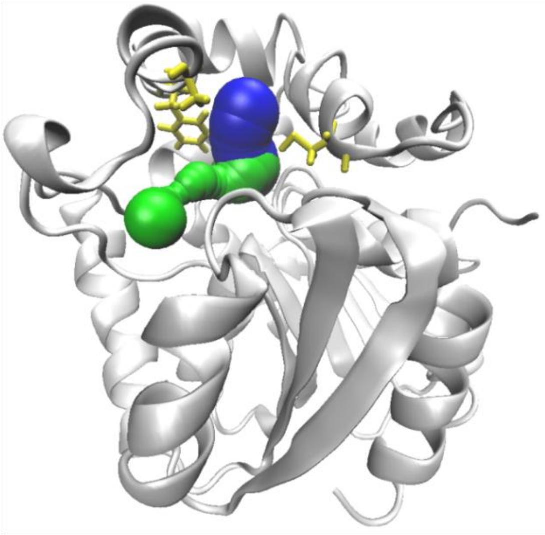

snapshot 31
