# Supplementary material for: CAVER 3.0: A Tool for the Analysis of Transport Pathways in Dynamic Protein Structures
Source: PLoS Comput Biol. 2012 Oct 18;8(10):e1002708. doi: 10.1371/journal.pcbi.1002708 (PMC3475669; doi:10.1371/journal.pcbi.1002708)
Supplement: Software S1 — CAVER 3.0 package containing CAVER 3.0 executable, source code, license, documentation and examples. The latest release of CAVER 3.0 can be downloaded from http://www.caver.cz. (ZIP) [file pcbi.1002708.s006.zip › caver_3.0/user_guide/caver_userguide.pdf]

Version 3.0

User Guide

The CAVER 3.0 software package, including source codes, executables, example data sets and this documentation, are distributed under the terms of the GNU General Public License v3.0. There is no warranty for the program, to the extent permitted by applicable law. Except when otherwise stated in writing the copyright holders and/or other parties provide the program "as is" without warranty of any kind, either expressed or implied, including, but not limited to, the implied warranties of merchantability and fitness for a particular purpose. The entire risk as to the quality and performance of the program is with you. Should the program prove defective, you assume the cost of all necessary servicing, repair or correction.

Recommended Citation:

Eva Chovancová, Antonín Pavelka, Petr Beneš, Ondřej Strnad, Jan Brezovský, Barbora Kozlíková, Artur Gora, Vilém Šustr, Martin Klvaňa, Petr Medek, Lada Biedermannová, Jiří Sochor & Jiří Damborský. **CAVER 3.0: A Tool for the Analysis of Transport Pathways in Dynamic Protein Structures.** *In preparation*

Developer Team:

Human Computer Interaction Laboratory
Faculty of Informatics, Masaryk University
Botanická 68a, 602 00 Brno, Czech Republic
Webpage: http://decibel.fi.muni.cz
Phone: +420 549 496 939, Fax: +420 549 491 820

Loschmidt Laboratories, Department of Experimental Biology and
Research Centre for Toxic Compounds in the Environment
Faculty of Science, Masaryk University
Kamenice 5, Bld. A13, 625 00 Brno, Czech Republic
Webpage: http://loschmidt.chemi.muni.cz
Phone: +420 549 493 467, Fax: +420 549 496 302

Webpage: **http://www.caver.cz**
E-mail: **caver@caver.cz**

© Copyright 2005-2012
Human Computer Interaction Laboratory, Faculty of Informatics
and Loschmidt Laboratories, Department of Experimental Biology and
Research Centre for Toxic Compounds in the Environment, Faculty of Science
Masaryk University
Brno, Czech Republic

Contents

A. INSTALLATION ____ **5**

Package content ____ 5

Executable jar file ____ 5

Source code ____ 5

B. RUNNING CAVER 3.0 ____ **6**

Running command ____ 6

Quick start ____ 7

C. INPUTS ____ **9**

Directory with PDB files ____ 9

Configuration file (config.txt) ____ 9

Bin directory ____ 9

D. CALCULATION SETTINGS ____ **11**

Calculation setup ____ 11

Input data ____ 12

Tunnel calculation ____ 14

Tunnel clustering ____ 16

Generation of outputs ____ 19

E. ADVANCED SETTINGS ____ **26**

Starting point optimization ____ 26

Advanced tunnel calculation ____ 27

Redundant tunnels removal ____ 29

Averaging of tunnel ends ____ 30

Approximate clustering ____ 31

Outputs ____ 33

Others ____ 35

F. OUTPUTS ____ **36**

summary.txt ____ 36

summary_precise_numbers.csv ____ 37

log.txt ____ 37

warnings.txt ____ 37

analysis *directory* ____ 37
pymol *directory* ____ 43
vmd *directory* ____ 46
data *directory* ____ 47
G. GUIDED EXAMPLE ____ 51
Calculation settings ____ 51
Running CAVER 3.0 ____ 51
Analysis of clustering results ____ 54
Adjusting clustering_threshold ____ 55
Generation of a complete set of output files ____ 57
Analysis of identified tunnels ____ 58

A. INSTALLATION

CAVER 3.0 is written in the Java programming language and runs on all operating systems with installed Java Runtime Environment 6.0 or higher. To check the version of Java installed on your system, follow the instructions at http://www.java.com/en/download/help/testvm.jsp.

CAVER 3.0 is licensed under the GNU General Public License v3.0. You can freely download the CAVER 3.0 package (**caver_3.0.zip**) including the CAVER 3.0 executable jar file, source code, documentation and examples from http://www.caver.cz/.

Package content:

- source_code
- caver:
  - caver.jar: CAVER executable jar file
  - bin: directory containing heat map palettes, template script files and table of atomic radii
  - lib: external libraries
  - license: CAVER license
- examples
  - QUICK_START
  - guided_example
  - static_structures
- user_guide
  - caver_userguide.pdf
  - config_default.txt: example configuration file with default settings of all parameters

Executable jar file

Download the **caver_3.0.zip** package and decompress it into any directory in your computer. If you have an appropriate version of Java installed on your system, you can directly start to use CAVER 3.0 (see B. Running CAVER 3.0).

Source code

If you want to build your own executable jar file, we recommend you to use NetBeans, which can be freely downloaded at http://netbeans.org/. Download the **caver_3.0.zip** package. Decompress the archive to any directory. Then start NeatBeans, go to "File" > "Open Project", then navigate to the directory where you decompressed the archive, enter the **source_code** directory and open the "**caver**" project. Finally, go to "Run" and click on "Clean and Build Project". Now, the file **caver.jar** and folder **lib** can be found in the directory where you unpacked the archive, under source_code/caver/dist. If you have an appropriate version of Java installed on your system, you can now start to use CAVER 3.0 (see B. Running CAVER 3.0). If you want to run CAVER on examples provided in the caver_3.0 package, you should copy the newly built caver.jar file and lib folder to the central caver directory (overwrite the original caver.jar and lib directory that were provided in the caver_3.0 package).

B. RUNNING CAVER 3.0

Running command

If you have CAVER 3.0 and Java installed on your system, you should be able to run CAVER 3.0 from the command line by typing:

java -Xmx*1200m* **-cp** *path_to_lib* **-jar** *path_to_caver.jar* **-home** *path_to_caver_home* **-pdb** *path_to_pdb_files* -**conf** *path_to_config_file* **-out** *path_to_output_directory*

-Xmx: specification of maximum Java heap size. Increase the maximum heap size if you encounter "out of memory" error. If you want to use heap size larger than 1200m, you will probably need to use 64bit java. Important: To ensure smooth calculation, please check that you have enough operating memory to allocate specified heap space and still maintain memory requirements of your system.

-cp: path to external libraries; (e.g., -cp ../caver /lib)

-jar: path to caver.jar (e.g., -jar ../caver/caver.jar)

-home: path to CAVER home directory (e.g., -home ../caver). This directory must contain **bin** directory.

-pdb: path to the directory with input PDB files (e.g., -pdb ./input_files)

-conf: path to the CAVER configuration file (e.g., -conf ./config.txt). This parameter is optional—if not specified, *Path_to_caver_home*/config.txt will be used

-out: path to the output directory (e.g., -out ./out). This parameter is optional—if not specified, *Path_to_caver_home*/out will be used

Example commands:

- java -Xmx1200m -cp ../caver/lib -jar ../caver /caver.jar -home ../caver -pdb ./input_files -conf ./config.txt out ./out
- java –Xmx4000m -cp ../caver /lib -jar ../caver /caver.jar -home ../caver -pdb ./input_files
- "C:\Program Files\Java\jre1.6.0_06\bin\java" -Xmx1000m -cp "C:\Program Files\caver\lib" -jar "C:\Program Files\caver\caver.jar" -home D:\caver_home -pdb ./input_files

Alternatively, you can also run CAVER 3.0 by launching the **caver.bat** file (make sure that all paths in caver.bat are specified correctly).

Warning: Paths must NOT end with backslash "\" or slash "/", otherwise the command might be interpreted incorrectly by the operating system. Tip: Both absolute and relative paths can be used.

Important: If you are getting the *"java is not recognized as an internal or external command, operable program or batch file"* error, try to specify the full path to java executable in the running command (or caver.bat file), e.g., "C:\Program Files\Java\jre1.6.0_06\bin\java" -Xmx1200m ... Alternatively, you may add java to the Path in your Environment Variables.

Important: If you are getting the "*Error occurred during initialization of VM. Could not reserve enough space for object heap. Could not create the Java virtual machine*." error, you should decrease Java heap size or use 64bit Java.

Quick start

The quick start briefly introduces the basics of CAVER analysis. For more detailed description of CAVER calculation, optimization of clustering parameters and interpretation of results, see **G. Guided example**.

1. Go to the **examples**/**QUICK_START/inputs** directory.

2. Edit the **caver.bat** file: specify the Java heap space (e.g., -Xmx1200m), path to the CAVER library (e.g., -cp ../../../caver/lib), path to the caver.jar file (e.g., -jar ../../../caver/caver.jar), path to the caver home directory (e.g., -home ../../../caver), path to the directory with PDB files (e.g., -pdb ../md_snapshots), and optionally also path to the configuration file (e.g., -conf ./config.txt) and output directory (e.g., -out ./out).

3. **Example**: java -Xmx1200m -cp ../../../caver/lib -jar ../../../caver/caver.jar -home ../../../caver -pdb ../md_snapshots -conf ./config.txt -out ./out
   Important: To ensure smooth calculation, please check that you have enough operating memory to allocate specified heap space and still maintain memory requirements of your system.

4. If you do not want to use example input structures, put your own PDB files into the **../md_snapshots** directory (or specify different directory in the caver.bat file).

5. Specify the calculation starting point and optionally also other parameters in the **config.txt** configuration file (especially *probe_radius*, *shell_radius*, *shell_depth* and *clustering_threshold*). If you plan to visualize the results in VMD, you should also set the *path_to_vmd* parameter.

6. Launch **caver.bat** (in unix/linux systems, make sure that the file is executable) or, alternatively, type the running command directly in the command line.
   Important:
   - If you are getting the *"java is not recognized as an internal or external command, operable program or batch file"* error, try to specify the full path to java executable in the running command (or caver.bat file), e.g., "C:\Program Files\Java\jre1.6.0_06\bin\java". Alternatively, you may add java to the Path in your Environment Variables.
   - If you are getting the "*Error occurred during initialization of VM. Could not reserve enough space for object heap. Could not create the Java virtual machine*." error, you should decrease Java heap size or use 64bit Java.
   - Try to increase the maximum heap size if you encounter "out of memory" error during the calculation of tunnels. If you want to use heap size larger than 1200m, you will probably need to use 64bit java. Alternatively, you may try to solve this problem by setting the *number_of_approximating_balls* parameter to a lower value.

Visualize clustering results in VMD using the out/vmd/**vmd_timeless.bat** (make sure that the path to VMD is correctly set in the script; you can also open the results using the VMD Tk console by typing "cd *Path_to_vmd_dir*" and then "source scripts/view_timeless.tcl"). Alternatively, you can visualize

the clustering results in PyMOL by running the out/pymol/**view_timeless.py** script (you have to associate the extension ".py" with PyMOL first or run the script via PyMOL using the menu File > Run > view_timeless.py or by typing "run view_timeless.py"in the PyMOL command line).

7. Visualize dynamic tunnels in VMD or PyMOL by running out/vmd/**vmd.bat** or out/pymol/**view.py** script, respectively.

8. Explore the tunnel characteristics provided in out/**summary.txt** and out/analysis/**tunnel_characteristics.csv** and out/analysis/**tunnel_profiles.csv.**

9. Use the data provided in out/analysis/**tunnel_profiles.csv** to plot profiles of selected tunnels.

10. Explore the tunnel-lining atoms and residues using the out/analysis/**atoms.txt** and out/analysis/**residues.txt** files.

11. Explore the bottleneck residues of individual tunnels using the out/analysis/**bottlenecks.csv** file.

12. Explore the profile and bottleneck heat maps provided in the out/analysis/**profile_heat_maps** and out/analysis/**bottleneck_heat_maps** directory.

Tip: If you want to adjust the clustering results, just change the *clustering_threshold* parameter. After that, set both the *load_tunnels* and *load_cluster_tree* parameters to *yes* and launch the caver.bat.

Important: If you want to exactly reproduce the results of previous analyses, you have to use the exactly same input files, with the same file names and content (the same number and order of atoms), as well as the same setting of the *seed* parameter.

C. INPUTS

Directory with PDB files

As an input, you have to provide a structure or a set of aligned structures in the PDB format. The directory with input structures has to be specified in the running command (or in the caver.bat file) by the -pdb parameter. Each input structure must be provided as one PDB file. Warning: The input structures have to be aligned beforehand by the user, otherwise irrelevant results will be obtained.

The atoms that you do not want to include in the CAVER 3.0 analysis (e.g, ligands, water molecules, etc.) can be removed manually beforehand or using the CAVER 3.0 atom filters (see the Input data parameters in section D.). If alternate conformations of residues are detected, they are either automatically removed by the program before the analysis (for each residue, the alternate conformation with the highest occupancy is retained), or in some specific cases, you will be asked to remove them manually. Important: The name of the PDB file is used for initialization of the pseudo-random number generator. If you want to reproduce the results of previous analyses precisely, you have to use the input files of exactly same names.

Configuration file (config.txt)

You can adjust all important calculation parameters by editing the calculation configuration file (see D. Calculation settings and E. Advanced settings). The configuration file can be specified in the running command (or in the caver.bat file) by the -conf parameter. If the configuration file is not specified in the configuration file, the caver_home/config.txt will be used. Important: You always have to specify the calculation starting point (there is no default value for that). Note that the optimal parameter settings vary for different systems and therefore we recommend you to adjust also other parameters (especially *probe_radius*, *shell_radius*, *shell_depth* and *clustering_threshold*) so that they suit your target system well. Tip: The order of individual parameters in the configuration file is not fixed and, therefore, you may arrange it as you wish. It is also not necessary to list all parameters in the configuration file—the default values are used for the parameters that are not specified by the user.

Bin directory

The bin directory contains the file with settings of atomic radii and template files for generation of heat maps and visualization scripts. If you want to change some of the predefined settings, edit the corresponding file in the bin directory. Warning: The bin directory must be located in the caver home directory, otherwise the CAVER 3.0 computation will not proceed.

Table of atomic radii

- **atom_radii.csv**: settings of radii of individual atoms. Edit this file if you want to specify radii of atoms that are not set or change the predefined settings.

Visualization templates for PyMOL

- **view_timeless.py**: template script for the visualization of clustering results in PyMOL. Edit this file if you want to change the predefined visualization.
- **view.py**: template script for the visualization of dynamic tunnels in PyMOL. Edit this file if you want to change the predefined visualization.
- **rgb.py**: template script for the coloring of tunnel clusters in PyMOL. Edit this file if you want to change the predefined coloring scheme.
- **zones.py**: template script for visualization of tunnel zones. Edit this file if you want to change the predefined visualization or coloring.

Visualization templates for VMD

- **view.tcl**: template script for the visualization of dynamic tunnels in VMD. Edit this file if you want to change the predefined coloring scheme of tunnels.
- **view_timeless.tcl**: template script for the visualization of clustering results in VMD. Edit this file if you want to change the predefined coloring scheme of tunnels.
- **vmd_load_structure.tcl**: template script for the loading of the sample input structure into VMD. Edit this file if you want to change the predefined visualization and color of the input structure.
- **vmd_load_structures.tcl**: template script for loading of trajectory into VMD. Edit this file if you want to change the predefined visualization and color of input structures.
- **radii.tcl**: template script for visualization of dynamic tunnels in VMD.
- **rgb.tcl**: template script for coloring of the tunnels in VMD.

Heat map palettes

- **palette.png**: color scale to be used for generating the profile heat map.
- **unknown.png**: color to be used to indicate missing tunnels or tunnel sections in the profile heat map.
- **bottleneck_palette.png**: color scale to be used for generating the bottleneck heat map.
- **bottleneck_unknown.png**: color scale to be used to indicate missing tunnels in the bottleneck heat map.

D. CALCULATION SETTINGS

Calculation setup

The CAVER 3.0 algorithm consists of three separable steps: (i) calculation of tunnels in each provided structure, i.e., each snapshot of molecular dynamics (MD) simulation; (ii) clustering of tunnels identified in all snapshots; and (iii) calculation and generation of output data. The results of each step can be saved and processed in the subsequent steps repeatedly with varying parameters. The setup of the calculation is controlled by the *load_tunnels*, *load_cluster_tree* and *stop_after* parameters.

load_tunnels yes | **no**

specifies whether the tunnels should be calculated or loaded from the disk.

- *load_tunnels* no (default): Calculation of tunnels will be performed, and identified tunnels will be stored in the out/data/tunnels/ directory. Warning: Previous results stored in the out/data/tunnels/ directory will be lost.
- *load_tunnels* yes: Tunnels will be loaded from the out/data/tunnels/ directory. This option enables to load the tunnels identified in previous analyses. Important: The tunnels are loaded as they are; therefore, changes in the settings of parameters related to the tunnel calculation step will not be reflected (this relates to parameters listed in the Input data, Tunnel calculation, Advanced tunnel calculation, Starting point optimization, Redundant tunnels removal, Averaging of tunnel ends and Others sections).

load_cluster_tree yes | **no**

specifies whether the hierarchy of tunnel clusters should be calculated or loaded from the disk.

- *load_cluster_tree* no (default): Pairwise distances of all tunnels from the out/data/tunnels/ directory will be calculated and used for hierarchical clustering of the tunnels. A tree hierarchy of tunnel clusters will be stored as a text file in the out/data/tree.txt. Warning: Previous results stored in the out/data/tree.txt file will be lost.
- *load_cluster_tree* yes: The tree hierarchy of tunnel clusters will be loaded from the out/data/tree.txt file. Important: The hierarchy of tunnel clusters is loaded as it is in the tree.txt file; therefore, changes in the settings of parameters related to the calculation of pairwise distances of tunnels will not be reflected (with the exception of *clustering_threshold*, this relates to all parameters listed in the Tunnel clustering and Averaging of tunnel ends sections). On the other hand, this option is highly recommended if you just want to optimize the *clustering_threshold* or output settings.

stop_after **never** | tunnels | cluster_tree

specifies the steps of CAVER 3.0 computation that should be performed.

- *stop_after* never (default): All three steps of CAVER 3.0 algorithm will be performed.
- *stop_after* tunnels: The computation will stop after the tunnel calculation step; this option is useful if you want to run the calculation of tunnels in different snapshots in parallel.
- *stop_after* cluster_tree: The computation will stop after the tunnel clustering step.

Input data

As the input, you have to provide a structure or a set of aligned structures in the PDB format. A sequence of digits is identified in the name of each PDB file and the name is divided onto prefix, number and suffix. PDB files are then ordered first by prefix, then in case of equality by number and finally in case of equality by suffix (e.g., model_9.pdb will be processed before model_10.pdb and both before structure_1.pdb). You can specify a subset of the structure dataset (MD simulation snapshots) that will be analyzed by the *time_sparsity*, *first_frame* and *last_frame* parameters.

Additionally, you can specify atoms (atoms representing protein structure, cofactors, water solvent, etc.) to be used for the analysis by the *include ** option or *include_atom_numbers*, *include_residue_ids, include_residue_names*, *exclude_atom_numbers*, *exclude_residue_ids* and *exclude_residue_names* parameters. Important: The CAVER algorithm first selects all atoms and residues specified by the "include" parameters, and then excludes all atoms or residues specified by the "exclude" parameters from this selection. The order of the "include" and "exclude" parameters in the config file does not influence the order of the whole process.

first_frame **1** | integer $n_{first} > 0$

specifies the first snapshot that will be analyzed.

- *first_frame* 1 (default): The analysis will start from the first snapshot.

last_frame **100000** | integer $n_{last} \geq n_{first}$

specifies the last snapshot that will be analyzed. Tip: The option can be set to a higher number than the actual number of snapshots in the dataset. In such a case, the *last_frame* parameter is neglected—all snapshots satisfying the *first_frame* and *time_sparsity* criteria will be used.

- *last_frame* 100000 (default): The last snapshot that will be analyzed is the $100{,}000^{th}$ snapshot.

time_sparsity **1** | integer $n > 0$

Every n-th snapshot from the range of snapshots specified by the *first_frame* and *last_frame* parameters will be analyzed.

- *time_sparsity* 1 (default): All snapshots will be analyzed.
- *time_sparsity* 10: Every tenth snapshot will be analyzed.

include *

All atoms present in the input structure will be selected, and those of them which are not specified in any of the *exclude_atom_numbers*, *exclude_residue_ids* or *exclude_residue_names* parameters will be used for the CAVER 3.0 calculation. Important: This option is automatically applied if none of the *include_atom_numbers*, *include_residue_ids*, *include_residue_names* parameters is specified in the config file.

include_residue_names list of residue names (no default value)

All atoms of all residues whose name is specified in the list will be selected, and those of them which are not specified in any of the *exclude_atom_numbers*, *exclude_residue_ids* or *exclude_residue_names* parameters will be used for the CAVER 3.0 calculation. Tip: Instead of listing all individual amino acids, you can use "20_AA". Warning: 20_AA stands only for the twenty most

commonly used notations of the twenty standard amino acids (ALA, ARG, ASN ASP, CYS, GLN, GLU, GLY, HIS, ILE, LEU, LYS, MET, PHE, PRO, SER, THR, TRP, TYR, and VAL). Other amino acid residues or residue notations must be specified separately (e.g. SEC, PYL, HIE, HIP, HID, etc.).

- *include_residue_names* HEM 20_AA: All atoms of the standard amino acids notated in the structure by their most commonly used abbreviations as well as all atoms of the HEM group will be selected, and those of them which are not specified in any of the "exclude" parameter will be used for the CAVER 3.0 calculation.

include_residue_ids list of residue identifiers (no default value)
All atoms of all residues whose identifier is specified in the list will be selected, and those of them which are not specified in any of the *exclude_atom_numbers*, *exclude_residue_ids* or *exclude_residue_names* parameters will be used for the CAVER 3.0 calculation. Last character of the identifier may be an insertion code (e.g., 20A). Instead of a number, a range can be specified (e.g. 5–15), without white space characters around '–'). Warning: If the same residue identifier relates to more chains, the chain should be specified before the identifier (e.g., B:5), otherwise the residue is considered to belong to the first chain.

- *include_residue_ids* 20 21 21A B:100 150–160: All atoms of residues 20, 21, 21A and 150–160 from the chain A and the residue 100 from the chain B will be selected, and those of them which are not specified in any of the "exclude" parameter will be used for the CAVER 3.0 calculation.

include_atom_numbers list of atom serial numbers (no default value)
All atoms whose serial number is in the list will be selected, and those of them which are not specified in any of the *exclude_atom_numbers*, *exclude_residue_ids* or *exclude_residue_names* parameters will be used for the CAVER 3.0 calculation. Instead of a number, a range can be specified (e.g. 5–15, without white space characters around '–').

- *include_atom_numbers* 5 150–160 321: Atoms with serial number 5, 150–160 and 321 will be selected, and those of them which are not specified in any of the "exclude" parameter will be used for the CAVER 3.0 calculation.

exclude_residue_names list of residue names (no default value)
All atoms of all residues whose name is specified in the list will be excluded from the CAVER 3.0 calculation.

- *exclude_residue_names* HOH CL: All water molecules (named in the input structure as HOH) and chloride ions will be excluded from the CAVER 3.0 calculation.

exclude_residue_ids list of residue identifiers (no default value)
All atoms of all residues whose identifier is specified in the list will be excluded from the CAVER 3.0 calculation. Last character of the identifier may be an insertion code (e.g., 20A). Instead of a number, a range can be specified (e.g. 5–15), without white space characters around '–'). Warning: If the same residue identifier relates to more chains, the chain should be specified before the identifier (e.g., B:5), otherwise the residue is considered to belong to the first chain.

- *exclude_residue_ids* 24B C:100: All atoms of residue 24B from the chain A and residue 100 from the chain C will be excluded from the CAVER 3.0 calculation.

exclude_atom_numbers list of atom serial numbers (no default value)
All atoms whose serial number is in the list will be excluded from the CAVER 3.0 calculation. Instead of a number, a range can be specified (e.g. 5–15, without white space characters around '– ').

- *exclude_atom_numbers* 6 3115–3140: Atoms with serial number 6 and 3115–3140 will be excluded from the CAVER 3.0 calculation.

Tunnel calculation

The starting point for the calculation of tunnels is initially placed into the center of gravity of the atoms, residues or point specified by *starting_point_atom*, *starting_point_residue* and *starting_point_coordinates*, respectively. You may use one, two or all three parameters. Each of the specified entities contributes by the same weight. Warning: There is no default value for the starting point parameters. Therefore, you must specify at least one of these parameters in the config file otherwise the calculation will not proceed. Additionally, you should adjust the *shell_radius* and *shell_depth* parameters, which are used for approximation of the protein molecular surface, and the *probe_radius* parameter specifying the minimum width of tunnels you are interested in.

starting_point_atom list of atom serial numbers (no default value)
specifies the serial numbers of atoms that will contribute to the calculation of the initial starting point.

- *starting_point_atom* 578 745: Atoms 578 and 745 will contribute to the calculation of the initial starting point.

starting_point_residue list of residue identifiers (no default value)
specifies the identifiers of residue that will contribute to the calculation of the initial starting point. Last character of the identifier may be an insertion code (e.g., 20A). Warning: If the same residue identifier relates to more chains, the chain should be specified before the identifier (e.g., B:5), otherwise the residue is considered to belong to the first chain.

- *starting_point_residue* 128 B:146: Residue 128 from the chain A and residue 146 from the chain B will contribute to the calculation of the initial starting point.

starting_point_coordinates x, y and z coordinates (no default value)
The point specified by the listed x, y and z coordinates will contribute to the calculation of the initial starting point.

- *starting_point_coordinates* 1.156 2.2 3.38: The point defined by the [1.156, 2.2, 3.38] coordinates will contribute to the calculation of the initial starting point.

***probe_radius* 0.9** | number $n > 0$
specifies the minimum radius the tunnel must have to be identified (all edges of the Voronoi diagram which cannot be traversed by the probe of the specified *probe_radius* are removed). Warning: Using small values slows down the computation significantly, and reports many irrelevant tunnels, as such tunnels exist nearly everywhere.

- *probe_radius* 0.9 (default): Only the tunnels with the bottleneck (minimum) radius ≥ 0.9 Å will be identified.

***shell_radius* 3** | number *n* > 0
specifies the radius of the shell probe which will be used to define which parts of the Voronoi diagram represent the bulk solvent. Warning: The value of *shell_radius* must be higher than the bottleneck radius of each tunnel in the input structure, otherwise no tunnel will be identified (all tunnels with the bottleneck radius larger than the shell_radius, and hence also the calculation starting point, will be assigned as the bulk solvent). To enable identification of tunnels, the *shell_radius* is in such cases set to the value of the largest bottleneck radius in a given snapshot multiplied by the value of the *automatic_shell_radius_bottleneck_multiplier* parameter (see the Advanced settings). Important: The value of *shell_radius* should be higher than maximum radii of individual tunnels. Therefore, this value may significantly differ between individual systems and you should adjust it to suit your target system (e.g., the optimal *shell_radius* for transmembrane channel proteins is usually much larger than for globular proteins).

- *shell_radius* 3 (default): The probe of 3 Å radius will be used to define the bulk solvent region (all vertices in the Voronoi diagram that can be accessed from protein exterior by this probe will be considered as the bulk solvent vertices). Warning: The default value is rarely suitable for transmemrane proteins or any other proteins with wide channels or tunnels.

***shell_depth* 4** | number *n* > 0
specifies the maximal depth of a surface region, i.e., a part of the input structure located below the bulk solvent region. The tunnel branching is not allowed in the surface region. The minimum distance of the surface region from the calculation starting point is controlled by the *starting_point_protection_radius* (see the Advanced settings). Important: The *shell_depth* parameter was implemented to minimize overshadowing of tunnels, and especially identification of irrelevant tunnel branches appearing as tunnels slithering on the protein surface. Identification of many irrelevant tunnels slithering on the protein surface indicates that the *shell_depth* value is probably not set correctly and should be increased. Warning: The tunnel branching is prohibited within the surface region. Therefore make sure that all important tunnel branches are identified under your settings. If some important tunnel or some important tunnel branch is not identified, the *shell_depth* parameter is probably set to a too high value and should be decreased.

- *shell_depth* 4 (default): The maximal depth of the surface region will be 4 Å

Tunnel clustering

The clustering of tunnels is performed by the average-link hierarchical algorithm based on the calculated matrix of pairwise tunnel distances (i.e., dissimilarities). Alternatively, only the matrix of distances can be generated, and used as the input for clustering in the user-provided external software. The switch between these two options is controlled by the *clustering* parameter.

For the purpose of calculation of pairwise tunnel distances, each tunnel is divided into three zones—the starting zone, middle zone, and the ending zone—of which only the tunnel segments belonging to the middle_zone are considered in the computation. The individual zones are defined by the *exclude_start_zone, exclude_end_zone* and *min_middle_zone* parameters. The visualization of zone boundaries can be saved via the *save_zones* parameter. The calculation of pairwise distances is also influenced by the *weighting_coefficient* defining the importance of individual tunnel segments for the calculation, and by the parameters from the Averaging of tunnel ends section (Advanced settings).

The clustering result is stored as a tree hierarchy of tunnel clusters. The size of the resulting clusters may be optimized rapidly by cutting this tree at a varying level of detail, which is specified by the *clustering_threshold* parameter. Warning: The average-link hierarchical clustering becomes relatively demanding in respect to computer time and disk capacity for datasets containing more than 50,000 tunnels. Therefore, for the clustering of large datasets (generally > 100,000 tunnels, but depends on the computational capacity available), a fast approximate algorithm based on supervised machine learning is recommended (see the Approximate clustering section in the Advanced settings). Important: Note that by changing the *clustering_threshold* or any other parameter related to the clustering, some tunnel clusters may join together or split into more clusters. Since only the cheapest (i.e., lowest cost) tunnel is retained for each cluster in each snapshot by default (see the *one_tunnel_in_snapshot* parameter in the Generation of outputs section), the splitting and joining of clusters may appear as emerging or disappearing of some tunnels, respectively (Figure 1A). If you want to see the clustering results for all tunnels, you have to switch the *one_tunnel_in_snapshot* parameter to *no* (Figure 1B).

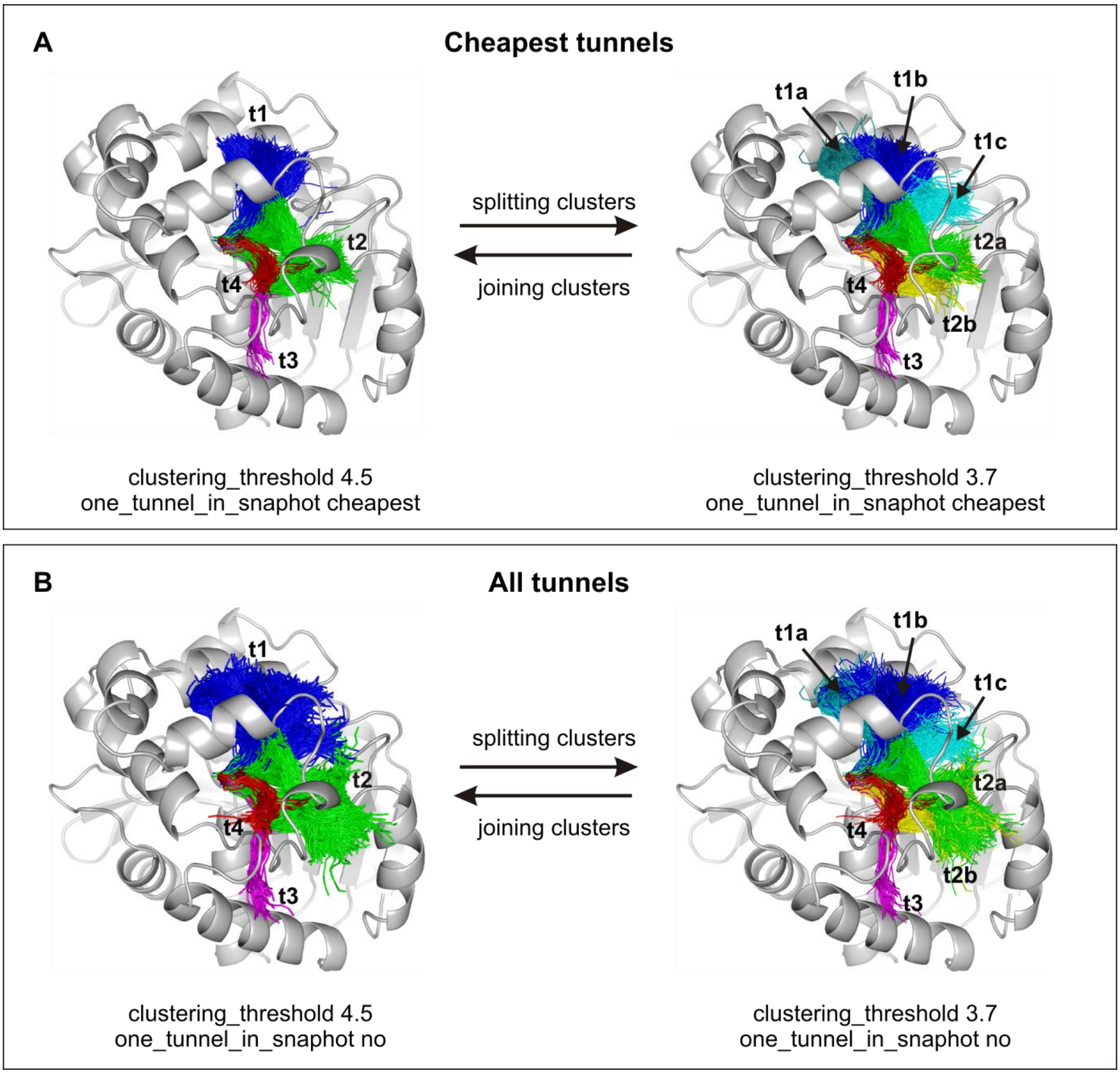

Figure 1. The influence of the *clustering_threshold* parameter on the resulting tunnel clusters. The tunnels identified throughout the entire MD simulation are shown as their centerlines, all in one snapshot. Individual tunnel clusters are colored by different colors. A decrease of clustering_threshold leads to splitting of the t1 cluster into t1a, t1b and t1c clusters, and splitting of the t2 cluster into t2a and t2b clusters, while an increase of the threshold leads to the joining of all the t1 and t2 clusters into one t1 and t2 cluster, respectively. A) For each snapshot, only the best (i.e., cheapest) tunnel of a given cluster is shown (one_tunnel_in_snapshot set to *cheapest*, default settings). Therefore, many tunnels which were visible using the clustering_threshold of 3.7 are not visible under setting with higher clustering_threshold, as they became part of some other cluster with better tunnels. B) The same clustering results as in the panel A, only this time all tunnels are visualized (one_tunnel_in_snapshot set to *no*).

clustering **average_link** | matrix
specifies whether the calculated matrix of pairwise tunnel distances should be directly processed by the intrinsic average-link hierarchical clustering algorithm or stored on the disk for future processing by the user-provided clustering software.

- *clustering* average_link (default): The average-link hierarchical clustering will be performed. The tree hierarchy of tunnel clusters will be stored in the out/data/tree.txt file.
- *clustering* matrix: The clustering step will end after generation of the distance matrix. The matrix will be stored in the out/data/matrix.edges file.

***weighting_coefficient* 1** | number *n* > 0

defines the importance of individual tunnel segments for the calculation of pairwise tunnel distances. The importance of the tunnel segments can be either constant along the entire tunnel (*weighting_coefficient* = 1) or linearly increase (*weighting_coefficient* > 1) or decrease (*weighting_coefficient* < 1) toward the tunnel ending (i.e., entrance to the tunnel from the bulk solvent). The segments used for the calculation of pairwise tunnel distances are specified by the *exclude_start_zone*, *exclude_end_zone* and *min_middle_zone* paramters. Important: If you change the *weighting_coefficient*, you have to repeat the clustering procedure (set *load_cluster_tree* to *no*), otherwise the change will not be reflected.

- *weighting_coefficient* 1 (default): All tunnel segments will have equal impact for the calculation of pairwise tunnel distances.
- *weighting_coefficient* 10: The distance between the ending sections of two tunnels will have ten times higher impact for the calculation of the pairwise distance of these tunnels than the distance between their beginning sections.

***clustering_threshold* 3.5** | number *n* ≥ 0

specifies the level of detail at which the tree hierarchy of tunnel clusters will be cut, and thus influences the size of resulting clusters. The higher the *clustering_threshold*, the larger the resulting clusters. Important: If you have conducted your analysis and just want to optimize *clustering_threshold* parameter, you do not need to repeat the time-consuming clustering step (set load_tunnels and load_cluster_tree to *yes*).

- *clustering_threshold* 3.5 (default): the tree hierarchy of tunnel clusters will be cut at the value of 3.5

***exclude_start_zone* 2** | number *n* ≥ 0

The tunnel segments located within the *exclude_start_zone* distance from the average starting point (i.e., the center of gravity of starting points from all input structures) will not be used for the calculation of pairwise tunnel distances. This parameter enables to diminish differences in positions of calculation starting points in individual input structures, which are caused by the structure dynamics. Important: If you change the *exclude_start_zone*, you have to repeat the clustering procedure (set *load_cluster_tree* to *no*), otherwise the change will not be reflected.

- *exclude_start_zone* 2 (default): The tunnel segments located within 2 Å distance from the average starting point will be excluded from the calculation of the pairwise tunnel distances.

***exclude_end_zone* 0** | number *n* ≥ 0

The tunnel segments located within the *exclude_end_zone* distance from the average tunnel end will not be used for the calculation of pairwise tunnel distances. This parameter enables to diminish differences in length of tunnels from individual snapshots caused by the dynamical changes of

surface protrusions. Important: If you change the *exclude_end_zone*, you have to repeat the clustering procedure (set *load_cluster_tree* to *no*), otherwise the change will not be reflected.

- *exclude_end_zone* 0 (default): the parameter will not be applied (i.e., no ending segments will be excluded from the calculation of pairwise tunnel distances).
- *exclude_end_zone* 1: The tunnel segments located within 1 Å distance from the average surface will be excluded from the calculation of pairwise tunnel distances.

min_middle_zone **5** | number *n* ≥ 0
specifies the minimum distance between the ending and starting zone of the tunnel. This parameter should guarantee that a sufficient portion of the tunnel segments will be used for the calculation of the pairwise tunnel distances. Important: If you change the *min_middle_zone*, you have to repeat the clustering procedure (set *load_cluster_tree* to *no*), otherwise the change will not be reflected.

- *min_middle_zone* 5 (default): The starting zone must be at least 5 Å far from the ending zone.

save_zones **yes** | no
Each tunnel is divided into three zones: the starting zone (see the *exclude_start_zone* parameter), middle zone, and the ending zone (see the *exclude_end_zone* parameter). For the calculation of pairwise tunnel distances, only the tunnel segments belonging to the middle zone are used. The *save_zones* parameter specifies whether the points delineating those three zones will be saved for visualization.

- *save_zones* yes (default): The points delineating individual tunnel zones will be saved into the out/data/start_zone.pdb and out/data/end_zone.pdb, and can be visualized in PyMOL using the out/pymol/zones.py script.
- *save_zones* no: The points delineating individual tunnel zones will not be saved.

Generation of outputs

Very often, two or even more tunnels belonging to the same cluster are identified in the same input structure. In such cases, only the best (i.e., cheapest) tunnel is reported, if not specified otherwise by the *one_tunnel_in_snapshot* parameter. The maximum number of tunnel clusters that will be reported can be limited by the *max_output_clusters* parameter.

The identified tunnels can be visualized all in a single frame, which is useful for the evaluation of clustering results and a general overview of tunnel variability, and additionally also as a MD trajectory to visually analyze the changes of individual tunnels over time. While the files for the visualization of all tunnels in a single frame are always generated, the latter option is controlled by the *save_dynamics_visualization* parameter. Important: If you have conducted your analysis and just want to change visualization settings, set both the *load_tunnels* and *load_cluster_tree* parameters to *yes*.

Additionally to the files for visualization, the calculation results include: (i) summary characteristics of individual tunnel clusters, (ii) characteristics of individual tunnels in individual snapshots, (iii) data for plotting of tunnel profiles, (iv) data for plotting of bottleneck radius and throughput histograms, (v) heat maps showing time evolution of the bottleneck radii and pathway profiles, (vi) list of tunnel-lining atoms and residues and (vii) list of the residues making up the tunnel

bottleneck. Generation of all these output files for large datasets may be quite time-consuming. The *generate_summary*, *generate_tunnel_characteristics*, *generate_tunnel_profiles*, *generate_histograms*, *generate_profile_heat_map*, *generate_bottleneck_heat_map*, *compute_tunnel_residues* and *compute_bottleneck_residues* parameters can be used to control which of the output files should be generated. The *bottleneck_histogram*, *throughput_histogram*, *profile_heat_map_resolution*, *profile_heat_map_range*, *profile_heat_map_element_size*, *bottleneck_heat_map_range* and *bottleneck_heat_map_element_size* parameters enables to define how individual histograms and heat-maps should look like, while the *residue_contact_distance* and *bottleneck_contact_distance* parameters specify the criteria to be used for the identification of tunnel-lining and bottleneck atoms and residues.

Tip: During the optimization of calculation settings, it is generally useful to generate the summary, tunnel characteristics and tunnel profiles files, while the reporting of other output files can be switched off. All output files can be generated after the calculation settings are optimized. Important: If you have conducted your analysis and just want to change output settings, set both the *load_tunnels* and *load_cluster_tree* parameters to *yes*. Further parameters related to the generation of outputs are available in the Outputs section (Advanced settings).

one_tunnel_in_snapshot **cheapest** | random | no

specifies which tunnels will be retained in the case that two or more tunnels from the same cluster were identified in the same input structure. Warning: This parameter relates not only to the visualization but also to all other output files.

- *one_tunnel_in_snapshot* cheapest (default): For each cluster, only one—the cheapest—tunnel will be retained in individual snapshots. Tip: This option is recommended for the final interpretation of data.
- *one_tunnel_in_snapshot* random: For each cluster, only one—randomly selected—tunnel will be retained in individual snapshots.
- *one_tunnel_in_snapshot* no: All tunnels will be retained. Warning: The summary characteristics of individual tunnel clusters are calculated using only the cheapest tunnels (i.e., the lowest cost tunnel from a given tunnel cluster in each snapshot). Otherwise, the characteristics of the tunnel clusters, which contain in some snapshots multiple tunnels, would be underestimated. Tip: This option is recommended for the optimization of parameters related to clustering, as it provides the overview of the clustering results for all tunnels. On the other hand, this option is not recommended for the final interpretation of data.

max_output_clusters **999** | number $n > 0$

specifies the maximum number of tunnel clusters that will be reported. Warning: if you use too small value of *max_output_clusters*, you may overlook some important tunnel clusters. You should always make sure that all important clusters are included in the reported results.

- *max_output_clusters* 999 (default): The results will be reported for at most 999 top-ranked tunnel clusters.

save_dynamics_visualization yes | **no**

specifies whether the dynamic visualization of tunnels will be generated.

- *save_dynamics_visualization* no (default): The dynamic visualization of tunnels will not be generated.
- *save_dynamics_visualization* yes: The dynamic visualization of tunnels will be generated; the tunnels separated into frames will be stored in the out/data/clusters/ directory, and can be easily opened in VMD by the out/vmd/vmd.bat script or in PyMOL using the out/pymol/view.py script. Important: Make sure that the *path_to_vmd* parameter is correctly set (see Outputs section in the Advanced settings). Important: Opening of large MD simulation may cause PyMOL to crash. Therefore, the out/pymol/view.py script loads only one static structure of protein into PyMOL. If you want to load all input structures into PyMOL, set the *generate_trajectory* to *yes* (see Outputs section in the Advanced settings), and run the out/pymol/view_trajectory.py script. Tip: Generation of the dynamic visualization for large datasets may be time-consuming. Therefore, we recommend you to use this option only for the final production of data (not during the optimization of calculation parameters).

generate_summary **yes** | no
specifies whether the summary characteristics of individual tunnel clusters will be reported.

- *generate_summary* yes (default): The summary characteristics of individual tunnels, including their frequency, priority, mean and maximum radius of the tunnel bottleneck and the mean length, curvature and throughput of the tunnel, will be saved to the out/summary.txt file.
- *generate_summary* no: The summary characteristics will not be reported.

generate_tunnel_characteristics **yes** | no
specifies whether the tunnel characteristics will be reported individually for each tunnel in each snapshot.

- *generate_tunnel_characteristics* yes (default): The tunnel characteristics, including the tunnel bottleneck radius, length, curvature, cost and throughput, will be reported individually for each tunnel in each snapshot and saved to the out/analysis/tunnel_characteristics.csv file. Tip: Use this option if you want to analyze the changes of tunnel characteristics over time.
- *generate_tunnel_characteristics* no: The detailed tunnel characteristics will not be reported.

generate_tunnel_profiles **yes** | no
specifies whether the data for the plotting of the tunnel profiles (i.e., the tunnel radius over distance from the start) will be reported.

- *generate_tunnel_profiles* yes (default): The tunnel profile of each tunnel in each snapshot will be saved to the out/analysis/tunnel_profiles.csv file. Important: The smoothness of the profile can be controlled by the *profile_tunnel_sampling_step* parameter (see the Output section in the Advanced settings).
- *generate_tunnel_profiles* no: The profiles of individual tunnels will not be reported.

generate_histograms yes | **no**
specifies whether the distributions of the bottleneck radii and throughputs will be reported for individual tunnel clusters.

- *generate_histograms* no (default): The distributions of the bottleneck radii and throughputs will not be reported
- *generate_histograms* yes: The distributions of the bottleneck radii and throughputs of individual tunnel clusters will be reported and stored in the out/analysis/histograms directory. Tip: The upper and lower limits and the number of intervals of the histogram are controlled by the *bottleneck_histogram* and *throughput_histogram* parameters.

***bottleneck_histogram* 0 2.0 20** | numbers n_1 n_2 integer n_3 (where $n_1 \geq 0$; $n_2 > n_1$; $n_3 > 0$)
specifies the histogram lower and upper limits (i.e., the minimum and maximum value of the bottleneck radius that will be included in the histogram) and the number of intervals of the histogram. Important: Applies only if the *generate_histograms* parameter is set to *yes*.

- *bottleneck_histogram* 0 2.0 20 (default): The distributions of the bottleneck radii of each tunnel cluster will be reported using 20 intervals, with the lower limit of 0 Å and the upper limit of 2.0 Å: <0, 0.1), <0.1, 0.2), ..., <1.9, 2.0). Tip: We recommend you to set the lower and upper limits so that they reflect the range of bottleneck radii observed in your system.

***throughput_histogram* 0 1.0 10** | numbers n_1 n_2 integer n_3 (where $n_1 \geq 0$; $n_2 > n_1$; $n_3 > 0$)
specifies the histogram lower and upper limits (i.e., the minimum and maximum value of the throughput that will be included in the histogram) and the number of intervals of the histogram. Important: Applies only if the *generate_histograms* parameter is set to *yes*.

- *throughput_histogram* 0 1.0 10 (default): The distributions of the throughputs of each tunnel cluster will be reported using 10 intervals, with the lower limit of 0 and the upper limit of 1.0: <0, 0.1), <0.1, 0.2),..., <0.9, 1.0). Tip: We recommend you to set the lower and upper limits so that they reflect the range of throughputs observed in your system.

generate_bottleneck_heat_map yes | **no**
specifies whether the heat map showing the time evolution of the bottleneck radii of individual tunnel clusters will be generated.

- *generate_bottleneck_heat_map* no (default): The heat map will not be generated.
- *generate_bottleneck_heat_map* yes: The heat map showing the time evolution of bottleneck radii of individual tunnel clusters will be saved as the bottleneck_heat_map.png file in the out/analysis/bottleneck_heat_maps directory. Important: The heat map color scale is defined by the bin/bottleneck_palette.png and bin/bottleneck_unknown.png files and can be modified by users. Additional settings of heat maps can be controlled by the *bottleneck_heat_map_range* and *bottleneck_heat_map_element_size* parametes. Warning: Generation of the heat maps for a large dataset may be memory demanding. Therefore, we recommend you to use this option only for the final production of data (not during the optimization of calculation parameters).

***bottleneck_heat_map_range* 1.0 2.0** |numbers n_1 n_2 (where $n_1 \geq 0$ and $n_2 > n_1$)
defines the range of values of the bottleneck radii that will be displayed in the heat map by different colors (corresponding to the color scale). Important: The values out of the specified range will be displayed by the corresponding boundary color, i.e., the color on the left of the color scale for values lower than the lower limit of the *bottleneck_heat_map_range*, and by the color on the right of the

color scale for values higher than the upper limit of the *bottleneck_heat_map_range*. Important: Applies only if the *generate_bottleneck_heat_map* parameter is set to *yes*.

- *bottleneck_heat_map_range* 1.0 2.0 (default): The heat map color scale will be adjusted to the range of tunnel bottleneck radii from 1.0 Å (color on the left of the color scale) to 2.0 Å (color on the right of the color scale). The tunnels with bottleneck radii falling within the specified *bottleneck_heat_map_range* will be colored based on the adjusted color scale. The tunnels with the bottleneck radius larger than 2.0 Å will be colored by the same color as the tunnels with the bottleneck radius equal to 2.0 Å. Similarly, the tunnels with the bottleneck radius lower than 1.0 Å will be colored by the same color as the tunnels with the bottleneck radius equal to 1.0 Å.

bottleneck_heat_map_element_size **10 10** | integers n_1 n_2 (where $n_1 > 0$ and $n_2 > 0$)
specifies the width and height of each heat map element (i.e., one tunnel) in pixels. Important: Applies only if the *generate_bottleneck_heat_map* parameter is set to *yes*.

- *bottleneck_heat_map_element_size* 10 10 (default): Each tunnel in the heat map will be represented by a square of 10 px width and 10 px height.

generate_profile_heat_map yes | **no**
specifies whether the heat maps showing the time evolution of the profiles of individual tunnel clusters will be generated.

- *generate_profile_heat_map* no (default):The profile heat maps will not be generated.
- *generate_profile_heat_map* yes: The profile heat maps showing time evolution of the profiles of individual tunnel clusters as well as the averaged tunnel profile of each tunnel cluster will be saved as the cl_*CLUSTER ID*_profile_heat_map.png files in the out/analysis/profile_heat_maps directory. Important: The heat map color scale is defined by the bin/palette.png and bin/unknown.png files and can be modified by users. Additional settings of heat maps can be controlled by the *profile_heat_map_resolution*, *profile_heat_map_range* and *profile_heat_map_element_size* parametes. Warning: Generation of the heat maps for a large dataset may be memory demanding. Therefore, we recommend you to use this option only for the final production of data (not during the optimization of calculation parameters).

profile_heat_map_resolution profile_tunnel_sampling_step | $n \geq$ profile_tunnel_sampling_step
defines the length of the tunnel segment corresponding to one element, i.e., one segment of the tunnel profile, in the heat map. Warning: The must not be lower than profile_tunnel_sampling_step, otherwise there can be gaps of unknown radius in the heat map. Important: Applies only if the *generate_profile_heat_map* parameter is set to *yes*.

- *profile_heat_map_resolution* value equal to the setting of the *profile_tunnel_sampling_step* parameter (default): Each segment of the tunnel profile in the heat map will correspond to the *profile_tunnel_sampling_step* Å tunnel segment.
- *profile_heat_map_resolution* 0.5: Each segment of the tunnel profile in the heat map will correspond to the 0.5 Å tunnel segment.

profile_heat_map_range **1.0 2.0** |numbers n_1 n_2 (where $n_1 \geq 0$ and $n_2 > n_1$)
defines the range of values of the tunnel radii that will be displayed in the heat map by different colors (corresponding to the color scale). Important: The values out of the specified range will be displayed by the corresponding boundary color, i.e., the color on the left of the color scale for values lower than the lower limit of the *profile_heat_map_range* and by the color on the right of the color scale for values higher than the upper limit of the *profile_heat_map_range*. Important: Applies only if the *generate_profile_heat_map* parameter is set to *yes*.

- *profile_heat_map_range* 1.0 2.0 (default): The heat map color scale will be adjusted to the range of tunnel radii from 1.0 Å (color on the left of the color scale) to 2.0 Å (color on the right of the color scale). The tunnel segments with radii falling within the specified *profile_heat_map_range* will be colored based on the adjusted color scale. The tunnel segments with radius larger than 2.0 Å will be colored by the same color as the segments with the radius equal to 2.0 Å. Similarly, the tunnel segments with radius lower than 1.0 Å will be colored by the same color as the segments with the radius equal to 1.0 Å.

profile_heat_map_element_size **20 10** | integers n_1 n_2 (where $n_1 > 0$ and $n_2 > 0$)
specifies the width and height of each heat map element (i.e., one segment of one tunnel profile) in pixels. Important: Applies only if the *generate_profile_heat_map* parameter is set to *yes*.

- *profile_heat_map_element_size* 20 10 (default): Each segment of the tunnel profile in the heat map will be represented by a rectangle of 20 px width and 10 px height.

compute_tunnel_residues yes | **no**
specifies whether the tunnel-lining atoms and residues will be reported.

- *compute_tunnel_residues* no (default): The tunnel-lining atoms and residues will not be reported.
- *compute_tunnel_residues* yes: The tunnel-lining atoms and residues will be identified and stored in the out/analysis/atoms.txt and out/analysis/residues.txt files. The tunnel-lining atoms can be visualized in PyMOL using the out/pymol/atoms.py script. Important: The atom and its corresponding residue are considered as tunnel-lining if the distance of the atom's surface to a surface of some tunnel ball is equal to or lower than the threshold defined by the *residue_contact_distance* parameter. Warning: Identification of the tunnel-lining atoms and residues in a large dataset is time-consuming. Therefore, we recommend you to use this option only for the final production of data (not during the optimization of calculation parameters).

residue_contact_distance **3.0** | number $n > 0$
The atom and its corresponding residue are considered as tunnel-lining if the distance of the atom's surface to a surface of some tunnel ball is equal to or lower than the value specified by the *residue_contact_distance* parameter. Important: Applies only if the *compute_tunnel_residues* parameter is set to *yes*.

- *residue_contact_distance* 3.0 (default): All atoms and residues located within the 3 Å distance from the tunnel surface (i.e. surface of the tunnel approximated by the sequence of balls) will be reported as tunnel-lining atoms and tunnel-lining residues, respectively.

compute_bottleneck_residues yes | **no**

specifies whether the bottleneck residues will be reported.

- *compute_bottleneck_residues* no (default): The bottleneck residues will not be reported.
- *compute_bottleneck_residues* yes: The bottleneck residues will be identified and stored in the out/analysis/bottlenecks.csv file. Important: The bottleneck residues are identified as residues with at least one atom within the specified distance (see the *bottleneck_contact_distance* parameter) from the tunnel bottleneck (i.e., the smallest ball of the tunnel). Warning: Identification of the bottleneck residues in large dataset is time-consuming. Therefore, we recommend you to use this option only for the final production of data (not during the optimization of calculation parameters).

bottleneck_contact_distance **3.0** | number *n* > 0

The residue is considered as the bottleneck residue if the distance of the residue's surface to a surface of the smallest ball of the tunnel is equal to or lower than the value specified by the *bottleneck_contact_distance* parameter. Important: Applies only if the *compute_bottleneck_residues* parameter is set to *yes*.

- *bottleneck_contact_distance* 3.0 (default): All residues located within the 3 Å distance from the tunnel bottleneck (i.e., from the surface of the smallest ball of the tunnel) will be reported as the bottleneck residues of a given tunnel.

E. ADVANCED SETTINGS

Starting point optimization

The calculation starting point is initially placed into the center of gravity of the user-specified residues, atoms or a point defined by x, y and z coordinates (see the *starting_point_atom*, *starting_point_residue* and *starting_point_coordinates* parameters in the Tunnel calculation section). The calculation starting point (i.e., starting Voronoi vertex) is then identified in the vicinity of the initial starting point based on the criteria specified by the *max_distance* and *desired_radius* parameters.

***max_distance* 3** | *n* ≥ 0

specifies the maximal distance of the calculation starting point (i.e. the starting Voronoi vertex) from the initial starting point. If no vertex is found within the user-specified distance, the default value of *max_distance* is used. If still no vertex is found, the vertex closest to the initial starting point is selected as the calculation starting point. Warning: Simultaneous setting of the *max_distance* and *desired_radius* parameters to high values may displace the calculation starting point outside of the input structure.

- *max_distance* 3 (default): The calculation starting point should be localized within the 3 Å distance from the initial starting point. If no vertex is found, the vertex closest to the initial starting point will be selected as the calculation starting point.
- *max_distance* 1.5: The calculation starting point should be localized within the 1.5 Å distance from the initial starting point. If no vertex is found within the 1.5 Å distance, the calculation starting point will be searched within the 3 Å distance. If no vertex is found within the 3 Å distance, the vertex closest to the initial starting point will be selected as the calculation starting point.

***desired_radius* 5.0** | *n* ≥ 0

The closest Voronoi vertex to the initial starting point, which is located within the specified distance (see the *max_distance* parameter) from the initial starting point and at least *desired_radius* far from the balls representing the input structure, will be used as the starting point for the calculation of tunnels. If no such vertex exists, then the vertex with maximal distance to the balls representing the input structure will be selected from all vertices which are located within the specified distance from the initial starting point. Tip: Setting of *desired_radius* to a high value will place the starting point to the center of the largest free space within the *max_distance* area. Warning: Simultaneous setting of the *max_distance* and *desired_radius* parameters to high values may displace the calculation starting point outside of the input structure.

- *desired_radius* 5 (default): The minimum distance between the starting Voronoi vertex and the balls representing the input structure should be 5 Å. If no such Voronoi vertex can be found, the vertex located within the specified distance from the initial starting and the maximal distance to the input structure will be used as the starting Voronoi vertex.

Advanced tunnel calculation

Prior to the calculation of the Voronoi diagram, all atoms of the input structure, which are larger than the smallest atom within the structure, are approximated by a user-specified number of balls with the van der Waals (VDW) radius equal to the VDW radius of the smallest atom. The *number_of_approximating_balls* parameter can be used to specify by how many balls will individual atoms be approximated.

A number of additional settings are available to control the tunnel searching step. Users can (i) limit the number of identified tunnels in individual input structures by the *max_number_of_tunnels* parameter, (ii) adjust the cost function (i.e., criteria for tunnel searching) via the *cost_function_exponent* and *max_limiting_radius* parameters, and (iii) request an automatic setting of the shell radius through the *automatic_shell_radius* and *automatic_shell_radius_bottleneck_multiplier* parameters. Finally, the *starting_point_protection_radius* parameter guaranteeing that the user-specified surface region will not collide with the calculation starting point can be adjusted.

number_of_approximating_balls 20 | **12** | 8 | 6 | 4

specifies the number of balls which will be used to approximate individual atoms in the input structure. Important: Approximation of the input structure by a set of balls with equal radii is necessary for correct construction of Voronoi diagram. The usage of approximation, however, leads to a certain overestimation of tunnel radii. The maximum calculation errors caused by the approximation are provided in individual output files (see the *compute_errors* and *save_error_profiles* parameters). Warning: Using high number of balls for the approximation increase the accuracy of results, but also the computational time and demands on memory. Try to decrease the number of balls if you encounter problems during construction of the Voronoi diagram (may happen for large molecular systems with high number of atoms).

- *number_of_approximating_balls* 12 (default): Selected atoms will be approximated by 12 balls with the radius equal to the van der Waals radius of the smallest atom in the input structure.

max_number_of_tunnels **10000** |integer $n > 0$

specifies the maximum number of tunnels that will be reported for one input structure (e.g., one snapshot). Important: The number of identified tunnels is influenced by the *probe_radius* parameter. Warning: If you use too small value of *max_number_of_tunnels*, you may overlook some important tunnels.

- *max_number_of_tunnels* 10000 (default): For each input structure, at most 10,000 top-scoring tunnels will be reported. Tip: Using the default value of 10,000 tunnels usually means that all identified tunnels will be reported.

max_limiting_radius 100 | numbers $0 \leq n \geq 100$

The tunnel segments with the radius larger than *max_limiting_radius* are optimized for length only. This parameter in fact specifies the radius up to which the differences in the tunnel width are biologically significant.

- *max_limiting_radius* 100 (default): The tunnel segments with the radius > 100 Å will be optimized for length only—the contribution of the tunnel width to the cost function will be same for all

such segments as for the segments with the radius of 100 Å. Tip: The default value in fact means that the *max_limiting_radius* parameter will not be used.

cost_function_exponent **2 |** number 0 ≤ *n* ≥ 100

The search for optimal tunnels is performed based on the criteria defined by the cost function. The *cost_function_exponent* parameter specifies the exponent of the cost function and by this way controls the balance between width and length of the tunnel. Tip: We recommend you to use the values between 0 and 3.

- *cost_function_exponent* 2 (default): The cost function exponent of 2 will be used, preferring wide paths with a reasonable length.
- *cost_function_exponent* 0: Only the length of the tunnel will be optimized.
- *cost_function_exponent* 50: The path only slightly narrower than the widest path will be more expensive, unless it is many times longer.

automatic_shell_radius yes | **no**

specifies whether the *shell_radius* parameter (see the Tunnel calculation section) will be set automatically using the *automatic_shell_radius_bottleneck_multiplier* parameter.

- *automatic_shell_radius* no (default): The user-defined value of *shell_radius* will be used.
- *automatic_shell_radius* yes: The value of *shell_radius* will be set automatically using the *automatic_shell_radius_bottleneck_multiplier* parameter.

automatic_shell_radius_bottleneck_multiplier **2** | number *n* > 1

The *shell_radius* parameter will be set to the value of the largest bottleneck radius in a given snapshot multiplied by the value of *automatic_shell_radius_bottleneck_multiplier*. Warning: Applies only if the *automatic_shell_radius* parameter is set to *yes* or in the case that the user-defined *shell_radius* is smaller than the bottleneck radius of some tunnel in a given input structure (for details, see the *shell_radius* parameter)

- *automatic_shell_radius_bottleneck_multiplier* 2 (default): The *shell_radius* parameter will be set to the value of the largest bottleneck radius in a given snapshot multiplied by 2. For example, if the largest bottleneck radius is 3 Å, *shell_radius* will be set to 6 Å (3 × 2).

starting_point_protection_radius **4** | number *n* ≥ 0

The parts of the Voronoi diagram within the specified *starting_point_protection_radius* distance from the calculation starting point will never be considered as the surface or bulk solvent region, even if *shell_radius* is set to very small and the shell_depth to very high value.

- *starting_point_protection_radius* 4 (default): The parts of the Voronoi diagram within the 4 Å distance from the calculation starting point will not be assigned as the surface or bulk solvent region.

Redundant tunnels removal

Several pathways with nearly identical axes may be identified within one input structure. The removal of such redundant pathways can be controlled by the *frame_clustering*, *frame_weighting_coefficient*, *frame_clustering_threshold*, *frame_exclude_start_zone*, *frame_exclude_end_zone* and *frame_min_middle_zone* parameters.

frame_clustering **yes** | no

specifies whether the redundant tunnels (i.e., very similar tunnels identified in the same input structure) will be removed. A simple clustering procedure based on the pairwise tunnel distances will be performed for this purpose—the cheapest tunnel will be selected as a representative and all tunnels, which have the pairwise distance with the representative tunnel smaller than the specified value (see *frame_clustering_threshold*) will be discarded. The procedure will be then repeated with the next cheapest tunnel from the remaining ones, until all tunnels are either selected or discarded. The calculation of pairwise tunnel distances can be controlled by the *frame_weighting_coefficient*, *frame_exclude_start_zone, frame_exclude_end_zone* and *frame_min_middle_zone* parameters.

- *frame_clustering* yes (default): All tunnels identified within one input structure will be clustered and only the cheapest tunnel will be retained for each cluster.
- *frame_clustering* no: All identified tunnels will be retained. Warning: Retaining of all redundant tunnels unnecessarily increase the computational and memory demands of the subsequent calculation steps. Therefore we recommend you to use this option only if you need it for some specific purpose.

frame_weighting_coefficient 1.0 | number $n > 0$

defines the importance of individual tunnel segments for the calculation of pairwise tunnel distances. The importance of the tunnel segments can be either constant along the entire tunnel (*frame_weighting_coefficient* = 1) or linearly increase (*frame_weighting_coefficient* > 1) or decrease (*frame_weighting_coefficient* < 1) toward the tunnel ending (i.e., entrance to the tunnel from the bulk solvent).

- *frame_weighting_coefficient* 1.0 (default): all tunnel sections will have the same impact for the calculation of pairwise tunnel distances.
- *frame_weighting_coefficient* 0.2: The distance between the starting sections of two tunnels will have five times higher impact for the calculation of the pairwise distance of these tunnels than the distance between their ending sections.

frame_clustering_threshold **1.0** | number $n \geq 0$

Two tunnels identified within one input structure with the pairwise tunnel distance smaller than the *frame_clustering_threshold* value will be considered as highly similar to each other. If one of these tunnels is assigned as a representative tunnel, the second one will be considered as redundant and discarded. Warning: Using too high values of *frame_clustering_threshold* may lead to discarding of important tunnels or tunnel branches. You should always make sure that only truly redundant tunnels are removed under your selected settings. Warning: Applies only if the *frame_clustering* parameter is set to *yes.*

- *frame_clustering_threshold* 1.0 (default): If the pairwise distance between a tunnel T and some representative tunnel from the same input structure is smaller than or equal to 1.0 Å, the tunnel T will be discarded as redundant.

frame_exclude_start_zone **0** | number *n* ≥ 0
The tunnel segments located within the *frame_exclude_start_zone* distance from the calculation starting point will not be used for the calculation of pairwise tunnel distances (relates to the calculation of pairwise distances of tunnels identified within the same input structure).

- *frame_exclude_start_zone* 0 (default): The *frame_exclude_start_zone* parameter will not be used.
- *frame_exclude_start_zone* 2: The tunnel segments located within 2 Å distance from the calculation starting point will be excluded from the calculation of the pairwise tunnel distances.

frame_exclude_end_zone **0** | number *n* ≥ 0
The tunnel segments located within the *frame_exclude_end_zone* distance from the average tunnel end will not be used for the calculation of pairwise tunnel distances (relates to the calculation of pairwise distances of tunnels identified within the same input structure).

- *frame_exclude_end_zone* 0 (default): The *frame_exclude_end_zone* parameter will not be used.
- *frame_exclude_end_zone* 2: The tunnel segments located within 2 Å distance from the average tunnel end will be excluded from the calculation of pairwise tunnel distances.

frame_min_middle_zone **5** | number *n* ≥ 0
specifies the minimum distance between the ending and starting zone of the tunnel. This parameter should guarantee that a sufficient portion of the tunnel segments will be used for the calculation of pairwise tunnel distances (relates to the calculation of pairwise distances of tunnels identified within the same input structure).

- *frame_min_middle_zone* 5 (default): The starting zone must be at least 5 Å far from the ending zone.

Averaging of tunnel ends

The pathway ending may prolong or shorten over time even though the other pathway segments do not change. It is usually undesirable to assign tunnels into different clusters just because of different length of their endings. While it is possible to align tunnel ends together for measuring tunnel distances, it is usually better to use an averaged location of their ends as a basis for tunnel alignment. To achieve this, an average surface (i.e., average tunnel ends) can be computed.

average_surface_frame **yes** | no
specifies whether the average surface will be used for calculation of tunnel distances (for the removal of redundant tunnels).

- *average_surface_frame* yes (default): Average surface will be used for removal of redundant pathways. Important: This option is rather suitable for calculation of tunnels than channels
- *average_surface_frame* no: Average surface will not be used for removal of redundant pathways.

average_surface_global yes | no
specifies whether the average surface will be used for calculation of tunnel distances (for the clustering of identified tunnels).

- *average_surface_global* yes (default): Average surface will be used for clustering of pathways. Important: This option is rather suitable for calculation of tunnels than channels
- *average_surface_global* no: Average surface will not be used for clustering of redundant pathways.

***average_surface_tunnel_sampling_step* 0.5** | number *n* >0
Each tunnel is represented by a sequence of balls, which are placed in regular intervals on the tunnel axis. For the purpose of the calculation of average surfaces, the distance between the centers of two neighboring balls is defined by the *average_surface_tunnel_sampling_step* parameter. This parameter in fact determines the accuracy of tunnel end point estimation Warning: Using low values slows down computation of average surfaces.

- *average_surface_tunnel_sampling_step* 0.5 (default): For the calculation of average surfaces, the tunnel will be represented by a sequence of balls placed on the tunnel axis in 0.5 Å distance intervals (i.e., distance between centers of two neighboring balls measured along the tunnel axis). Each tunnel point will be estimated with the tolerance of ± 0.25 Å.

***average_surface_smoothness_angle* 10** | number 0 <*n* < *360*
defines the size of space over which the tunnel ends are averaged to get a point of average surface. The space can be likened to a beam of light emanating from the averaged calculation starting point *S* (infinite cone with vertex *S*).

- *average_surface_smoothness_angle* 10 (default): The average surface point of a given tunnel is obtained by averaging tunnel ends which lie in the infinite cone of opening angle 20° (2 × *average_surface_smoothness_angle*)

***average_surface_point_min_angle* 5.73** | number n>0
Each tunnel end lies in an axis of a cone with the opening angle of 2 × *average_surface_point_min_angle*. If some higher cost tunnel lies within the cone of some cheaper tunnel, the position of tunnel end of the cheaper tunnel is used to represent both of them, thus saving the computation time.

- *average_surface_point_min_angle* 5.73 (default): the cone with an opening angle of 11. 46° (2 × 5.73) will be used to select representative tunnel ends. Tip: the values around 5 represent a reasonable compromise between accuracy and performance.

Approximate clustering

The average-link hierarchical clustering becomes relatively demanding in respect to computer time and disk capacity for large datasets. The *do_approximate_clustering* parameter can be used to switch on the approximate clustering procedure. In the first step, a set of tunnels of manageable size (specified by the *cluster_by_hierarchical_clustering* parameter) is randomly sampled from the tunnel dataset and clustered using the average-link hierarchical clustering. Then the specified number of the top-scoring clusters (set by the *max_training_clusters* parameter) is treated as separate classes, and

all remaining clusters as a single class. This dataset is then used for training a *k*-nearest neighbor classifier. Finally, the classifier is used to assign remaining tunnels from the original dataset either to one of the top-ranking clusters or to the "unclassified" cluster. Users may choose whether they want to report the "unclassified" cluster in the outputs by the *generate_unclassified_cluster* paramter.

do_approximate_clustering yes | **no**
specifies whether the hierarchical average-link clustering will be combined with the approximate algorithm based on supervised machine learning.

- *do_approximate_clustering* no (default): All tunnels will be clustered by the hierarchical average-link clustering. Warning: The average-link clustering becomes demanding in respect to computer time and disk capacity for large datasets.
- *do_approximate_clustering* yes: A portion of tunnels specified by the *cluster_by_hierarchical_clustering* parameter will be clustered by average-link clustering. The remaining tunnels will be classified using the supervised machine learning. Warning: The approximate clustering is less accurate than the average-link clustering. This option is recommended for large datasets containing hundreds of thousands of tunnels as well as in the case that the average-link hierarchical clustering takes too much time or fails due to insufficient memory or disk space.

cluster_by_hierarchical_clustering **20000** | integer *n* > 0
specifies the number of tunnels that will be clustered by average-link clustering and used as training data. The remaining tunnels will be clustered using the classifier (see the *classifier* parameter) trained on the results of the average-link clustering. Important: the number of the tunnels used for the training should not be too small. Warning: Applies only if the *do_approximate_clustering* parameter is set to *yes.*

- *cluster_by_hierarchical_clustering* 20000 (default): 20,000 randomly selected tunnels will be clustered by average-link hierarchical clustering; all remaining tunnels will be classified using the supervised machine learning.

max_training_clusters **15** | integer *n* > 0
specifies the maximum number of top-ranking tunnel clusters that will be distinguished by the classifier (see the *classifier* parameter). All remaining tunnel clusters will be regarded as one cluster. Important: Make sure that the parameter is set so that all biologically relevant tunnel clusters are among the top-ranking clusters which are distinguished by the classifier (i.e., do not belong to the last cluster containing a mixture of different tunnels). Warning: Applies only if the *do_approximate_clustering* parameter is set to *yes.*

- *max_training_clusters* 15 (default): The 15 top-ranking tunnel clusters will be distinguished by the classifier, all remaining clusters will be regarded as one cluster.

generate_unclassified_cluster yes | **no**
The classifier distinguishes the defined number of top-ranking tunnel clusters (see the *max_training_clusters* parameter), while all remaining tunnel clusters are regarded as one

“unclassified” cluster. The *generate_unclassified_cluster* parameter specifies whether the “unclassified” cluster containing a mixture of different tunnels will be reported.

- *generate_unclassified_cluster* no (default): Only the top-ranking tunnel clusters which are distinguished by the classifier will be reported.
- *generate_unclassified_cluster* yes: All tunnel clusters including the “unclassified” cluster will be reported. Important: We recommend you to use this option to check that all important tunnels were classified into one of the top-ranking clusters (i.e., were not included in the “unclassified” cluster containing a mixture of different tunnels). Warning: The “unclassified” cluster may get a good score, which is entirely artificial. Warning: Generation “unclassified” tunnel cluster for a large dataset may be time-consuming.

Outputs

The advanced output parameters include (i) *compute_errors* and *save_error_profiles*, controlling the reporting of calculation errors, (ii) *tunnel_sampling_step* and *visualization_sampling_step*, specifying the density of tunnel balls along the tunnel axis, (iii) *visualize_tunnels_per_cluster* and *visualization_subsampling*, defining the sample of tunnels that will be visualized, and finally (iv) *path_to_vmd* and *generate_trajectory*, which are related to the visualization of results in VMD and PyMOL, respectively.

compute_errors yes | **no**

specifies whether the maximal calculation errors caused by the approximation of the input structure (see the *number_of_approximating_balls* parameter) will be reported.

- *compute_errors* no (default): The calculation errors will not be reported.
- *compute_errors* yes: The maximal calculation errors will be reported in the out/summary.txt and out/analysis/tunnel_characteristics.csv files. Warning: The computation of the maximal calculation errors for large datasets is time-consuming. Therefore, we recommend you to use this option only for the final production of data (not during the optimization of calculation parameters).

save_error_profiles yes | **no**

specifies whether the profiles of the maximal calculation errors caused by the approximation of the input structure (see the *number_of_approximating_balls* parameter) will be reported.

- *save_error_profiles* no (default): The profiles of the calculation errors will not be reported.
- *save_error_profiles* yes: The profiles of the maximal calculation errors will be reported in the out/analysis/tunnel_profiles.csv. Important: Applies only if the *compute_errors* parameter is set to *yes*. Warning: The computation of the maximal calculation errors for large datasets is time-consuming. Therefore, we recommend you to use this option only for the final production of data (not during the optimization of calculation parameters).

profile_tunnel_sampling_step **0.5** | number $n > 0$

Each tunnel is represented by a sequence of balls, which are placed in regular intervals on the tunnel axis. For the purpose of the calculation of profiles and tunnel-lining atoms, the distance between the

centers of two neighboring balls is defined by the *profile_tunnel_sampling_step* parameter. Warning: Using low values slows down computation of the profiles and tunnel-lining atoms.

- *profile_tunnel_sampling_step* 0.5 (default): For the calculation of the tunnel profile and tunnel-lining atoms, the tunnel will be represented by a sequence of balls placed on the tunnel axis in 0.5 Å distance intervals (i.e., distance between centers of two balls measured along the tunnel axis).

visualization_tunnel_sampling_step **1.0** | number *n* > 0

Each tunnel is represented by a sequence of balls, which are placed in regular intervals on the tunnel axis. For visualization purposes, the distance between the centers of two neighboring balls is defined by the *visualization_tunnel_sampling_step* parameter. Warning: Using low values slows down visualization of tunnels in PyMOL.

- *visualization_tunnel_sampling_step* 1.0 (default): For the visualization purposes, the tunnel will be represented by a sequence of balls placed on the tunnel axis in 1.0 Å distance intervals (i.e., distance between centers of two balls measured along the tunnel axis).

visualize_tunnels_per_cluster **10000** | integer *n* > 0

specifies for each cluster the maximum number of tunnels that will be visualized. The criterion for the selection of tunnels can be controlled by the *visualization_subsampling* parameter. Important: The parameter relates only to the visualization not to the other output files. Warning: If you want to see a representative set of tunnels, you should avoid using of too small values. On the other hand, using of too high values may cause PyMOL to crash.

- *visualize_tunnels_per_cluster* 5000 (default): For each tunnel cluster, maximally 5,000 selected tunnels will be visualized.

visualization_subsampling **random** | cheapest

If the number of tunnels in some cluster exceeds the limit specified by the *visualize_tunnels_per_cluster* parameter, a subsample of the tunnels is selected for visualization purposes. The *visualization_subsampling* parameter specifies the criterion which will be used for selection of the subsample. Important: Applies only if some cluster contains a higher number of tunnels than specified by the *visualize_tunnels_per_cluster* parameter. Tip: For everyday usage, we recommend you to use the default setting (*random*) as it provides better overview of existing tunnels than the *cheapest* option.

- *visualization_subsampling* random (default): The subsample of tunnels for visualization will be selected randomly.
- *visualization_subsampling* cheapest: The tunnels will be ordered by their cost and the specified number of the cheapest tunnels will be selected for visualization. Warning: This option does not provide a representative set of tunnels, therefore, we recommend you to use it only if you need it for some specific purpose.

path_to_vmd **"c:/Program Files/University of Illinois/VMD/vmd.exe"** | path to the VMD executable specifies the path to the VMD executable on your system. Tip: The setting of this parameter enables you to use the provided scripts (out/vmd/vmd_timeless.bat and out/vmd/vmd.bat) for an automatic opening of the results in VMD software.

generate_trajectory yes | **no**
specifies whether a multi-model PDB file with all input structures will be created and stored as out/data/trajectory.pdb. This file is necessary for the visualization of structure dynamics in PyMOL.

- *generate_trajectory* no (default): The multi-model PDB file will not be created.
- *generate_trajectory* yes: The multi-model PDB file with all input structures will be created. The trajectory together with dynamical tunnels can be loaded into PyMOL using the out/pymol/view_trajectory.py script. Warning: Opening of large MD simulation may cause PyMOL to crash. If this happens, we recommend you to either open the results in VMD (using the out/vmd/vmd.bat script) or use the out/pymol/view.py script, which loads the dynamical tunnels and only one static input structure into PyMOL. Important: For visualization of dynamical tunnels, you have to set the *save_dynamics_visualization* parameter is set to *yes*.

Others

seed **1** | -2147483648 ≤ *n* ≤ 2147483647
represents the seed for pseudo-random number generator. If this parameter is omitted, the seed is initialized at the start of computation using current time in milliseconds. If a specific seed is provided, pseudorandom number generator is initialized before computation of tunnels in each snapshot using this seed and the number derived from the name of the analyzed file. Seed is also later used for random subsampling of pathways. The advantage of specific seed is that results stay exactly same for one input. Omitting the seed has the advantage of unbiased computation.

swap **yes** | maybe | no
specifies whether part of information related to identified tunnels will be stored on hard disk to save the memory.

- *swap* yes (default): Part of the tunnel information will be stored on hard disk. Tip: We recommend you to use this option for analysis of large datasets. Warning: This option slows down the calculation.
- *swap* maybe: All tunnel information is stored in both memory and on hard disk. Tip: This option allows later to switch the *swap* parameter to *yes* without re-computing all tunnels. We recommend you to use it if you do not want to lose time by using the swap, but you are, at the same time, not sure whether you have enough memory to conduct the analysis without swap.
- *swap* no: All tunnel information will be stored in memory. Warning: If you analyze a large dataset and do not have enough memory, you may encounter out of memory errors during the lager steps of CAVER calculation. In such a case, you will have to re-compute all tunnels with the swap *parameter* set to *yes*.

F. OUTPUTS

The results are stored in the "**out**" directory containing the **analysis**, **data**, **pymol** and **vmd** folders, and **summary.txt**, **log.txt** and **warnings.txt** files.

summary.txt

Location: out/summary.txt

File with the summary information about all identified tunnel clusters. Important: All averaged values are calculated over snapshots, where a given cluster has at least one tunnel. The only exception where the average is calculated over all snapshots is Priority. Warning: The summary characteristics of individual tunnel clusters are calculated using only the cheapest tunnels (i.e., the lowest cost tunnel from a given tunnel cluster in each snapshot). Otherwise, the characteristics of the tunnel clusters, which contain in some snapshots multiple tunnels, would be underestimated.

- **ID**: The ID of a given tunnel cluster (= ranking of a given cluster based on the Priority).
- **No**: The total number of tunnels belonging to a given cluster (there can exist more tunnels in one snapshot).
- **No_snaps**: The number of snapshots with at least one tunnel with a radius larger or equal to the value specified by the *probe_radius* paramter.
- **Avg_BR**: The average bottleneck radius in a given tunnel cluster.
- **Max_BR**: The maximum bottleneck radius in a given tunnel cluster.
- **Avg_L**: The average tunnel length in a given tunnel cluster.
- **Avg_C**: The average tunnel curvature (*curvature = length/distance*, where *length* is the length of the tunnel and *distance* is the shortest possible distance between the calculation starting point and the tunnel ending point.
- **Priority:** The priority of a given tunnel cluster calculated by averaging sum of tunnel throughputs over all snapshots—if the tunnel is not found in a given snapshot, zero value is used.
- **Avg_throughput**: The average tunnel throughput in a given tunnel cluster.
- **Avg_up_E_BR**: The average upper error bound of bottleneck radius estimation.
- **Avg_up_E_TR**: The average upper error bound of tunnel profile radii estimation.
- **Max_up_E_BR**: The maximal upper errors bound of bottleneck radii estimation.
- **Max_up_E_TR**: The maximal upper error bound of tunnel radii estimation.
- **SD**: The standard deviation of a value listed in the column preceding the SD column.

Related parameters:

- *generate_summary*—specifies whether the summary characteristics of individual tunnel clusters will be reported.
- *compute_errors* yes | no—specifies whether the upper bounds of calculation errors will be reported.
- *one_tunnel_in_snapshot* cheapest | random | no—specifies which tunnels will be reported in the case that two or more tunnels belonging to the same cluster were identified in the same input structure.

summary_precise_numbers.csv

Location: out/summary_precise_numbers.csv

File containing precise numbers of Priority and Average throughput of individual tunnel clusters (these numbers are rounded in the summary.txt file).

log.txt

Location: out/log.txt

File containing the log of the calculation.

warnings.txt

Location: out/warnings.txt

File containing warning messages from the calculation.

analysis *directory*

tunnel_characteristics.csv

Location: out/analysis/tunnel_characteristics.csv

The CSV file containing information about characteristics of individual tunnels in individual snapshots:

- **Snapshot:** The name of the input structure, i.e. MD snapshot, in which a given tunnel was identified.
- **Tunnel cluster:** The ID of a tunnel cluster to which a given tunnel belongs (corresponds to the Tunnel cluster ID in the summary.txt).
- **Tunnel:** ID of a given tunnel in a given snapshot.
- **Throughput:** The throughput of a given tunnel (throughput = e^{-cost}).
- **Cost:** The cost of a given tunnel.
- **Bottleneck radius:** The radius of the bottleneck, i.e., the narrowest part, of a given tunnel.
- **Bottleneck R error bound:** The upper error bound for estimation of the bottleneck radius of a given tunnel, i.e., the maximal possible overestimation of the bottleneck radius due to approximation of input structure.
- **Length:** The length of a given tunnel.
- **Curvature:** The curvature of a given tunnel; curvature = *length*/*distance*, where *length* is the length of the tunnel (distance from the calculation starting point to the tunnel ending point calculated along the tunnel axis) and *distance* is the shortest possible distance between the calculation starting point and the tunnel ending point.

Related parameters:

- *generate_tunnel_characteristics* yes | no—specifies whether the tunnel_characteristics.csv file will be created.

- *max_output_clusters n* > 0—specifies the maximum number of tunnel clusters that will be reported.
- *compute_errors* yes | no—specifies whether the maximal calculation errors will be reported.
- *one_tunnel_in_snapshot* cheapest | random | no—specifies which tunnels will be reported in the case that two or more tunnels belonging to the same cluster were identified in the same input structure.

tunnel_profiles.csv

Location: out/analysis/tunnel_profiles.csv

The CSV file with data for plotting the profiles of individual tunnels in individual snapshots:

- **Snapshot:** The name of the input structure, i.e., MD snapshot, in which a given tunnel was identified.
- **Tunnel cluster:** The ID of a tunnel cluster to which a given tunnel belongs (corresponds to the Tunnel cluster ID in the summary.txt).
- **Tunnel:** ID of a given tunnel in a given snapshot.
- **Throughput:** The throughput of a given tunnel (throughput = e^{-cost}).
- **Cost:** The cost of a given tunnel.
- **Bottleneck radius:** The radius of the bottleneck, i.e., the narrowest part, of a given tunnel.
- **Average R error bound:** Average upper error bound for estimation of radii in a given tunnel profile.
- **Max. R error bound:** The maximal upper error bound for estimation of radii in a given tunnel profile.
- **Bottleneck R error bound:** The upper error bound for estimation of the bottleneck radius of a given tunnel, i.e., the maximal possible overestimation of the bottleneck radius.
- **Curvature:** The curvature of a given tunnel; curvature = *length*/*distance*, where *length* is the length of the tunnel (distance from the calculations starting point to the tunnel ending point calculated along the tunnel axis) and *distance* is the shortest possible distance between the calculation starting point and the tunnel ending point.
- **Length:** The length of a given tunnel.
- **Axis:** Characteristics reported for each point of the tunnel profile.
- **Values...**
  - Axis X: x coordinate of a given point of the tunnel axis.
  - Axis Y: y coordinate of a given point of the tunnel axis.
  - Axis Z: z coordinate of a given point of the tunnel axis.
  - Axis distance: shortest possible distance between a given point of the tunnel axis from the calculation starting point.
  - Axis length: distance between a given point of the tunnel axis from the calculation starting calculated along the tunnel axis.
  - Axis R: tunnel radius in a given point of the tunnel axis, i.e., the radius of the largest possible ball that can be centered at a given point of the tunnel axis without colliding with the input structure.

- Axis Upper limit of R overestimation: the upper error bound for estimation of the tunnel radius in a given point of the tunnel axis, i.e., the maximal possible overestimation of the tunnel radius in a given point.

Important: The tunnel profile is usually plotted as the tunnel radius over the tunnel length (i.e., the distance between the point and the calculation starting point measured along the tunnel axis). Alternatively, you can plot the tunnel radius over the shortest possible distance of the profile point from the calculation starting point. By this way, you usually obtain profiles that are better aligned to each other than profiles calculated along the tunnel axis.

Related parameters:

- *generate_tunnel_profiles* yes | no—specifies whether the tunnel_profiles.csv file will be created.
- *profile_tunnel_sampling_step* *n* > 0—specifies the distance (measured along the tunnel axis) between two neighboring profile points.
- *max_output_clusters* *n* > 0—specifies the maximum number of tunnel clusters that will be reported.
- *compute_errors* yes | no—specifies whether the maximal calculation errors will be reported.
- *save_error_profiles* yes| no—specifies whether the maximal calculation errors will be reported for each point of the tunnel profile; applies only if the compute_errors paramter is set to *yes.*
- *one_tunnel_in_snapshot* cheapest | random | no—specifies which tunnels will be reported in the case that two or more tunnels belonging to the same cluster were identified in the same input structure.

atoms.txt

Location: out/analysis/atoms.txt

The list of tunnel-lining atoms (atom serial numbers). For each tunnel cluster, all atoms located in at least one snapshot within the specified distance from some ball of a given tunnel are reported.

Related parameters:

- *compute_tunnel_residues* yes |no—specifies whether the atoms.txt file will be created.
- *residue_contact_distance* *n* > 0—all atoms (residues) located within the specified distance from some tunnel ball will be considered as tunnel-lining atoms (residues).
- *one_tunnel_in_snapshot* cheapest | random | no—specifies which tunnels will be reported in the case that two or more tunnels belonging to the same cluster were identified in the same input structure.

residues.txt

Location: out/analysis/residues.txt

The list of tunnel-lining residues. For each tunnel cluster, all residues located in at least one snapshot within the specified distance from some ball of a given tunnel are reported.

- **C**: ID of a chain to which a given residue belongs.
- **res**: Index of a given residue in the PDB file.
- **AA**: Residue name.

- **N**: Number of snapshots in which a given residue lined a given tunnel.
- **sideN**: Number of snapshots in which at least one side chain atom of a given residue lined a given tunnel. For this purpose, all atoms except those named H, N, C, O, CA or HA are considered as side chain atoms.
- **atoms**: Tunnel-lining atoms. Format is <number of snapshots>:<atom name>_<atom serial number> (e.g., 2:O_582—the atom 582(O) lined a given tunnel cluster in 2 snapshots); each atom can be for a given tunnel cluster counted only once in one snapshot (e.g., in the snapshot 3, the atom 2146 lines two tunnels from the tunnel cluster 4, however it will be counted only once).

Related parameters:

- *compute_tunnel_residues* yes |no—specifies whether the residues.txt file will be created.
- *residue_contact_distance n* > 0—all residues located within the specified distance from some tunnel ball will be considered as tunnel-lining residues.
- *one_tunnel_in_snapshot* cheapest | random | no—specifies which tunnels will be reported in the case that two or more tunnels belonging to the same cluster were identified in the same input structure.

bottlenecks.csv

Location: out/analysis/bottlenecks.csv

The list of bottleneck residues of individual tunnels. For each snapshot, all residues located within the specified distance from the bottleneck of a given tunnel are reported as its bottleneck residues.

- **Snapshot**: The name of the input structure, i.e. MD snapshot, in which a given tunnel was identified.
- **Tunnel cluster**: The ID of a tunnel cluster to which a given tunnel belongs (corresponds to the Tunnel cluster ID in the summary.txt).
- **Tunnel**: ID of a given tunnel in a given snapshot.
- **Throughput**: The throughput of a given tunnel (throughput = e^{-cost}).
- **Bottleneck X**: x-coordinate of the bottleneck of a given tunnel.
- **Bottleneck Y**: y-coordinate of the bottleneck of a given tunnel.
- **Bottleneck Z**: z-coordinate of the bottleneck of a given tunnel.
- **Bottleneck R**: The radius of the bottleneck of a given tunnel.
- **Bottleneck residues**: residues located within the specified distance from the bottleneck of a given tunnel; bottleneck residues are ordered from the closest to the most distant ones. Format is chain:residue index (e.g., B:152—residue 152 from the chain B).

Related parameters:

- *compute_bottleneck_residues* yes |no—specifies whether the bottleneck.csv file will be created.
- *bottleneck_contact_distance n* > 0—all residues located within the specified distance from the tunnel bottleneck will be reported as bottleneck residues of a given tunnel.
- *one_tunnel_in_snapshot* cheapest | random | no—specifies which tunnels will be reported in the case that two or more tunnels belonging to the same cluster were identified in the same input structure.

- *profile_tunnel_sampling_step n* > 0—specifies the distance (measured along the tunnel axis) between two neighboring profile points; the tunnel radius is calculated in each profile point, and therefore, the setting of this parameter influences the preciseness of localization of the tunnel bottleneck.

bottleneck_heat_maps *directory*
Location: out/analysis/bottleneck_heat_maps
The directory congaing heat maps, i.e., figures visualizing the time evolution of the bottleneck radii.

- **bottleneck_heat_map.png**: Heat map showing the time evolution of the bottleneck radii of individual tunnel clusters. The color of an element with coordinates x and y expresses the bottleneck radius of a tunnel from y^{th} tunnel (top of the figure corresponds to the first cluster) in x^{th} snapshot (Figure 2).
- **bottleneck_heat_map.csv**: File with the source data for plotting of the bottleneck heat map.

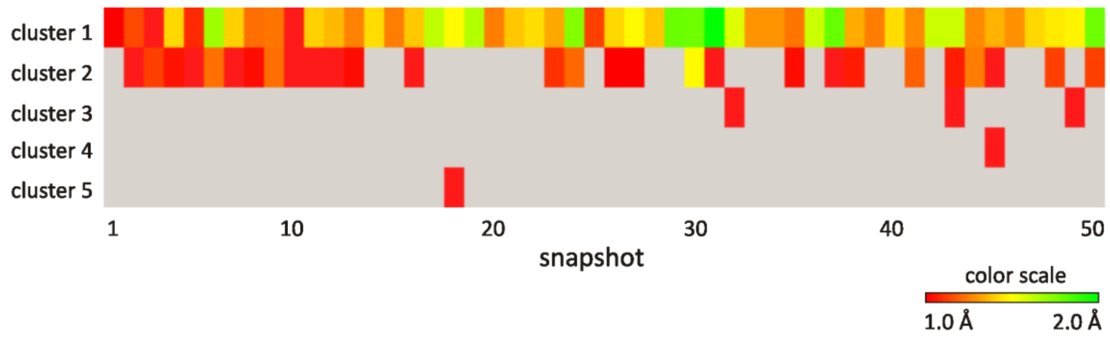

Figure 2. Heat map visualizing the time evolution of bottleneck radii of individual tunnel clusters. Each row corresponds to one cluster. The color of each element expresses the bottleneck radius of a given tunnel cluster in x^{th} snapshot. The gray color indicates that no tunnel from a given cluster was identified in a given snapshot.

Related parameters:

- *generate_bottleneck_heat_map* yes | no—specifies whether the bottleneck heat map will be generated.
- *bottleneck_heat_map_range n1 n2* (where *n1* ≥ 0 and *n2* > n1)—defines the range of values of the tunnel bottleneck radii that will be displayed in the heat map by different colors (corresponding to the color scale).
- *bottleneck_heat_map_element_size n1 n2* (where *n1* > 0 and *n2* > 0)—specifies the width and height (in pixels) of each element in the bottleneck heat map.
- bin/bottleneck_palette.png—color scale to be used for generating the bottleneck heat map.
- bin/bottleneck_unknown.png—color scale to be used to indicate missing tunnels in the bottleneck heat map.

profile_heat_maps *directory*
Location: out/analysis/ profile_heat_maps
The directory congaing heat maps, i.e., figures visualizing the time evolution of the profiles of individual tunnel clusters.

- **cl_*CLUSTER ID*_profile_heat_map.png** (e.g., cl_000001_profile_heat_map.png): Heat map showing the time evolution of profiles of a given tunnel cluster. The first column represents the average profile; each following column corresponds to one snapshot. The color of an element with coordinates x and y expresses the radius of a given tunnel in x-1th snapshot and yth distance interval (Figure 3).
- **average_images**: Directory containing the average profile heat map of each tunnel cluster. Important: The average image should be cut at the distance corresponding to the average distance of given tunnel cluster (the last elements in the y-axis are usually not relevant since they represent an average of only few—the longest—tunnels).
- **csv**: Directory containing source data for plotting of the profile heat maps.

Related parameters:

- *generate_profile_heat_map* yes | no—specifies whether the profile heat maps will be generated.
- *profile_heat_map_resolution n ≥ profile_tunnel_sampling_step—defines the length of the tunnel segment corresponding to one element, i.e., one segment of the tunnel profile, in the heat map.*
- *profile_heat_map_range n1 n2* (where *n1* ≥ 0 and *n2* > n1)—defines the range of values of the tunnel radii that will be displayed in the heat map by different colors (corresponding to the color scale).
- *profile_heat_map_element_size n1 n2* (where *n1* > 0 and *n2* > 0)—specifies the width and height (in pixels) of each element (i.e., one segment of one tunnel profile) in the profile heat map.
- bin/palette.png—color scale to be used for generating the profile heat map.
- bin/unknown.png—color to be used to indicate missing tunnels or tunnel segments in the profile heat map.

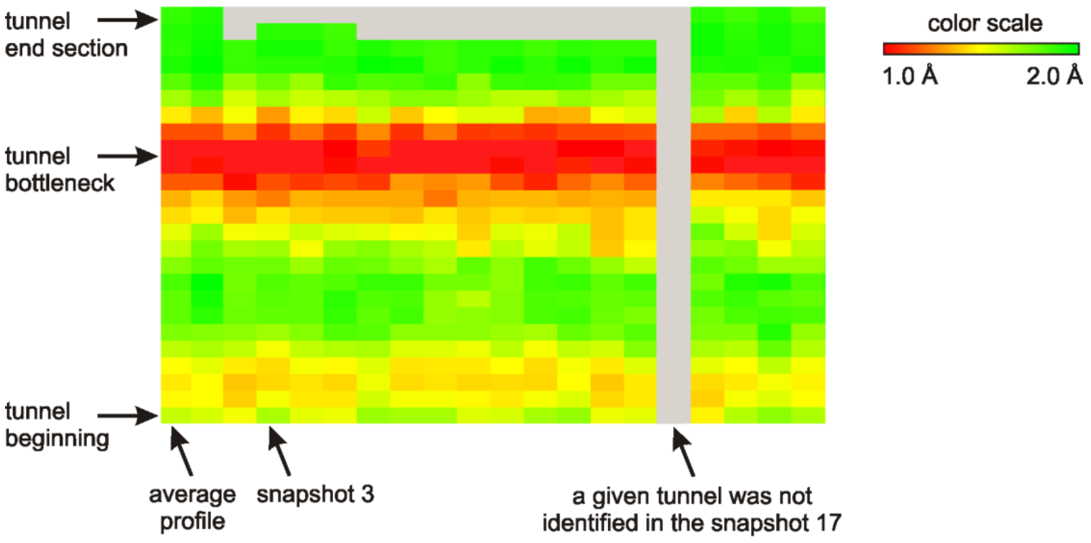

Figure 3. Heat map visualizing the time evolution of the tunnel profile. The first column represents the average profile; each following column corresponds to one snapshot. The color of an element with coordinates x and y expresses the radius of a given tunnel in x-1th snapshot and yth distance interval.

Histograms *directory*

Location: out/analysis/histograms

The directory containing data for plotting of the bottleneck radius and throughput histograms.

bottleneck_histograms.csv:

- **Tunnel cluster**: The ID of a given tunnel cluster.
- **Average bottleneck radius**: The average bottleneck radius of a given tunnel cluster.
- **Standard deviation**: The standard deviation of bottleneck radii in a given tunnel cluster.
- **Out of histogram range**: The number of tunnels with the value of the bottleneck radius out of the histogram range (e.g., histogram ranges from 1 Å to 2 Å, the "Out of the histogram range" column specifies the number of tunnels with bottleneck radii smaller than 1 Å or larger than 2 Å).
- **Histogram**: The number of tunnels belonging to individual histogram intervals, e.g., for the <0.9_0.96) interval, it is the number of tunnels with the bottleneck radii ≥ 0.9 and < 0.96.

throughput_histograms.csv

- **Tunnel cluster**: The ID of a given tunnel cluster.
- **Average throughput**: The average throughput of a given tunnel cluster.
- **Standard deviation**: The standard deviation of tunnel throughputs in a given tunnel cluster.
- **Out of histogram range**: The number of tunnels with the value of throughput out of the histogram range (e.g., histogram ranges from 0.5 to 1, the "Out of the histogram range" column specifies the number of tunnels with throughput smaller than 0.5 Å or larger than 1).
- **Histogram**: The number of tunnels belonging to individual histogram intervals, e.g., for the <0.5_0.85) interval, it is the number of tunnels with the throughput ≥ 0.5 and < 0.85.

Related parameters:

- *generate_histograms* yes | no—specifies whether the bottleneck_histograms.csv and throughput_histograms.csv files will be generated.
- *bottleneck_histogram* n1 n2 n3 (where n1 ≥ 0; n2 > n1; n3 > 0)—specifies the lower and upper limits (i.e., the minimum and maximum value of the bottleneck radius that will be included in the histogram) and the number of intervals of the histogram.
- *throughput_histogram* n1 n2 n3 (where n1 ≥ 0; n2 > n1; n3 > 0)— the lower and upper limits (i.e., the minimum and maximum value of the throughput that will be included in the histogram) and the number of intervals of the histogram.

pymol *directory*

view_timeless.py

Location: out/pymol/view_timeless.py

The script for the visualization of clustering results in PyMOL. The script opens the files from the out/data/clusters_timeless/ directory together with the out/data/origins.pdb and out/data/v_origins.pdb files. You can run the script either by clicking the view_timeless.py file (you have to associate the extension ".py" with PyMOL first) or run the script via the PyMOL menu (File > Run > view_timeless.py) or type "run view_timeless.py" in the PyMOL command line. Tip: If you want

to change the predefined visualization, edit the bin/view_timeless.py file. Warning: Opening of large data may cause PyMOL to crash. To prevent this, try to adjust the *visualization_tunnel_sampling_step* and *visualize_tunnels_per_cluster* parameters. Alternatively, you can use the out/vmd/vmd_timeless.bat script to open the clustering results in VMD.

Related parameters:

- bin/view_timeless.py—template script for the visualization of clustering results in PyMOL. Edit this file if you want to change the predefined visualization.
- *visualization_tunnel_sampling_step n* > 0—defines the distance between the centers of two neighboring balls used for visualization of tunnels.
- *visualize_tunnels_per_cluster n* > 0—specifies the maximum number of tunnels that will be visualized for each cluster.
- *visualization_subsampling* random | cheapest— specifies the criterion to be used for selection of the subsample of tunnels for visualization.
- *one_tunnel_in_snapshot* cheapest | random | no—specifies which tunnels will be reported in the case that two or more tunnels belonging to the same cluster were identified in the same input structure.

view_trajectory.py

Location: out/pymol/view_trajectory.py

The script for the visualization of dynamic tunnels in PyMOL. The script opens the files from the out/data/clusters/ directory together with the out/data/trajectory.pdb, out/data/origins.pdb and out/data/v_origins.pdb files. You can run the script either by clicking the view_trajectory.py file (you have to associate the extension ".py" with PyMOL first) or run the script via the PyMOL menu (File > Run > view_trajectory.py) or type "run view_trajectory.py" in the PyMOL command line. Tip: If you want to change the predefined visualization, edit the bin/view.py file. Warning: Opening of large MD simulation may cause PyMOL to crash. In such a case, try to use the out/pymol/view.py script, which loads only one static input structure into PyMOL. Alternatively, you can use the out/vmd/vmd.bat script and visualize the tunnel dynamics in VMD.

Related parameters:

- *generate_trajectory* yes | no — specifies whether the trajectory.pdb file will be created.
- *save_dynamics_visualization* yes | no—specifies whether the dynamic visualization of tunnels will be generated.
- *visualization_tunnel_sampling_step n* > 0—defines the distance between the centers of two neighboring balls used for visualization of tunnels.
- bin/view.py—template script for the visualization of dynamic tunnels in PyMOL. Edit this file if you want to change the predefined visualization.

view.py

Location: out/pymol/view.py

The script for the visualization of dynamic tunnels in PyMOL. The script opens the sample input structure and files from the out/data/clusters/ directory together with the out/data/origins.pdb and

out/data/v_origins.pdb files. You can run the script either by clicking the view.py file (you have to associate the extension ".py" with PyMOL first) or run the script via the PyMOL menu (File > Run > view.py) or type "run view.py" in the PyMOL command line. Tip: If you want to change the predefined visualization, edit the bin/view.py file. Warning: Opening of large MD simulation may cause PyMOL to crash. Therefore, the out/pymol/view.py script loads only one static input structure into PyMOL. If you want to load all input structures into PyMOL, set the *generate_trajectory* parameter to *yes* and run the out/pymol/view_trajectory.py script. Alternatively, you can use the out/vmd/vmd.bat script and visualize the tunnel dynamics in VMD.

Related parameters:

- *save_dynamics_visualization* yes | no—specifies whether the dynamic visualization of tunnels will be generated.
- *visualization_tunnel_sampling_step n* > 0—defines the distance between the centers of two neighboring balls used for visualization of tunnels.
- bin/view.py—template script for the visualization of dynamic tunnels in PyMOL. Edit this file if you want to change the predefined visualization.

atoms.py

Location: out/pymol/atoms.py

The script for the selection of tunnel-lining atoms in PyMOL. You can run the script via the PyMOL menu (File > Run > atoms.py) or type "run atoms.py" in the PyMOL command line.

Related parameters:

- *compute_tunnel_residues* yes |no—specifies whether the tunnel-lining atoms and residues will be created.
- *residue_contact_distance n* > 0—all atoms (and the corresponding residues) located within the specified distance from some tunnel ball will be considered as tunnel-lining atoms (residues).

zones.py

Location: out/pymol/zones.py

The script for the visualization of boundaries of three tunnel zones (starting zone, middle zone, ending zone). The script opens the out/data/zones.pdb file. You can run the script via the PyMOL menu (File > Run > zones.py) or type "run zones.py" in the PyMOL command line.

Related parameters:

- *save_zones*—specifies whether the zones.pdb file will be created.

rgb.py

Location: out/pymol/rgb.py

The script that is used by the out/pymol/view.py, out/pymol/view_trajectory.py and out/pymol/view_timeless scripts for the coloring of individual tunnel clusters. Tip: If you want to change the predefined tunnel colors, edit the bin/rgb.py file.

Related parameters:

- bin/rgb.py—template script for the coloring of tunnel clusters in PyMOL. Edit this file if you want to change the predefined coloring scheme.

vmd *directory*

vmd_timeless.bat

Location: out/vmd/vmd_timeless.bat

The script for the visualization of clustering results in VMD. The script launches VMD and the out/vmd/scripts/view_timeless.tcl script. All input structures together with tunnel clusters from the out/data/clusters_timeless/ directory are loaded. Tip: If you want to change the predefined visualization and colors, edit the bin/vmd/view_timeless.tcl or bin/vmd_load_structure.tcl files. Important: Make sure that the *path_to_vmd* parameter is correctly set. If the vmd_timeless.bat script does not work, try to open the results using the VMD Tk console (Extensions > Tk Console) by typing "cd *Path_to_vmd_dir*" and then "source scripts/view_timeless.tcl"

Related parameters:

- *path_to_vmd* path to the VMD executable—specifies the path to the VMD executable on your system for an automatic opening of the results in VMD software.
- *visualization_tunnel_sampling_step n* > 0—defines the distance between the centers of two neighboring balls used for visualization of tunnels.
- bin/view_timeless.tcl—template script for the visualization of clustering results in VMD. Edit this file if you want to change the predefined coloring scheme of tunnels.
- bin/vmd_load_structure.tcl—template script for the loading of sample input structure into VMD. Edit this file if you want to change the predefined visualization and color of the input structure.
- *visualize_tunnels_per_cluster n* > 0—specifies the maximum number of tunnels that will be visualized for each cluster.
- *visualization_subsampling* random | cheapest— specifies the criterion to be used for selection of the subsample of tunnels for visualization.

vmd.bat

Location: out/vmd/vmd.bat

The script for the visualization of dynamic tunnels in VMD. The script launches VMD and the out/vmd/scripts/view.tcl script. All input structures together with the dynamic tunnels from the out/data/clusters/ directory are loaded. Tip: If you want to change the predefined visualization and colors, edit the bin/vmd/view.tcl or bin/vmd_load_structures.tcl files. Important: Make sure that the *path_to_vmd* parameter is correctly set. If the vmd.bat script does not work, try to open the results using the VMD Tk console (Extensions > Tk Console) by typing "cd *Path_to_vmd_dir*" and then "source scripts/view.tcl"

Related parameters:

- *save_dynamics_visualization* yes | no—specifies whether the dynamic visualization of tunnels will be generated.

- *path_to_vmd* path to the VMD executable—specifies the path to the VMD executable on your system for an automatic opening of the results in VMD software.
- *visualization_tunnel_sampling_step n* > 0—defines the distance between the centers of two neighboring balls used for visualization of tunnels.
- *one_tunnel_in_snapshot* cheapest | random | no—specifies which tunnels will be reported in the case that two or more tunnels belonging to the same cluster were identified in the same input structure.
- bin/view.tcl—template script for the visualization of dynamic tunnels in VMD. Edit this file if you want to change the predefined coloring scheme of tunnels.
- bin/vmd_load_structures.tcl view.tcl—template script for the loading of trajectory into VMD. Edit this file if you want to change the predefined visualization and color of the input structure.

scripts *directory*

Location: out/vmd/scripts

The directory with scripts used by the out/vmd/vmd.bat and out/vmd_timeless.bat for visualization of the results in VMD. The scripts can also be opened using the VMD Tk console (Extensions > Tk Console) by typing "cd *Path_to_vmd_dir*" and then "source scripts/*name_of_the_script*.tcl"

data *directory*

clusters_timeless *directory*

Location: out/data/clusters_timeless

The directory with PDB files for the visualization and evaluation of clustering results. Each PDB file represents one tunnel cluster. Large clusters are divided into more PDB files. Tunnels identified in all input structures (e.g, throughout the entire MD simulation) are stored in one frame. All PDB files may be opened at once in PyMOL (Figure 4) using the out/pymol/view_timeless.py script or in VMD using out/vmd/vmd_timeless.bat script.

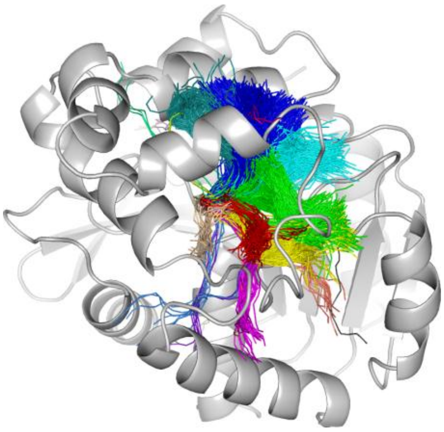

Figure 4. Visualization of clustering results in PyMOL. All tunnels identified throughout the entire MD simulation are visualized in one frame, which is useful for the evaluation of the clustering results and for a general overview of the tunnel variability. Individual tunnel clusters are colored in different colors, tunnels are shown as tunnel center-lines.

clusters *directory*
Location: out/data/clusters
The directory with PDB files for visualization of tunnel dynamics. Each PDB file represents one cluster of tunnels. Large clusters are divided into more PDB files. Tunnels identified in different snapshots are stored in separate frames. All PDB files may be opened at once in VMD (Figure 5) using out/vmd/view.bat script or in PyMOL using out/pymol/view.py or out/pymol/view_trajectory.py scripts.

Related parameters:

- *save_dynamics_visualization* yes | no—specifies whether the dynamic visualization of tunnels will be generated.

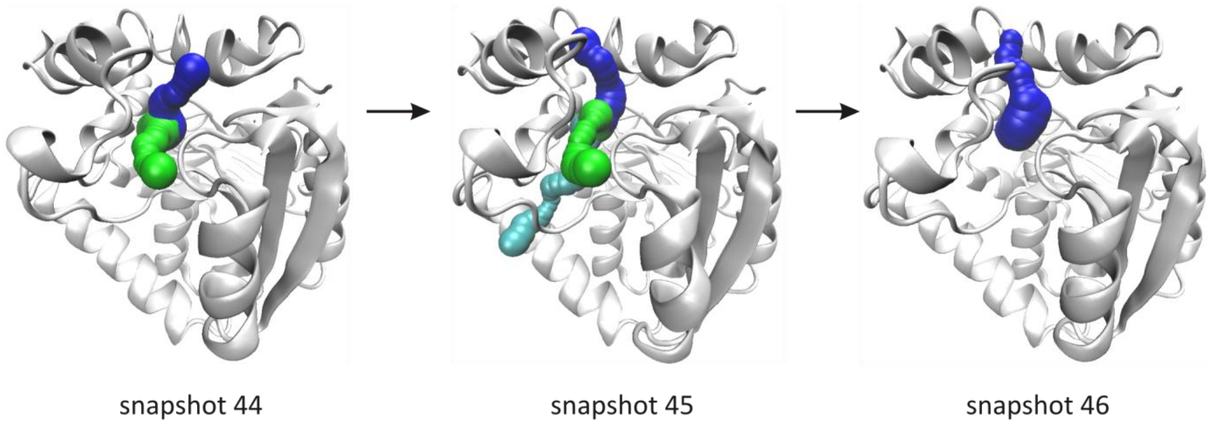

Figure 5. Visualization of tunnel dynamics in VMD. Three snapshot are shown, individual tunnels (represented by a sequence of balls) are colored in different colors.

tunnels *directory*
Location: out/data/tunnels
The storage of identified tunnels. Tunnels identified within one snapshot are stored in one file.

Related parameters:

- *load_tunnels* yes | no—specifies whether the tunnels will be calculated or loaded from the disk.

tunnel_edges *directory*
Location: out/data/tunnel_edges
The storage of tunnel edges. Edges of tunnels identified within one snapshot are stored in one file.

Related parameters:

- *swap* yes | no—specifies whether the tunnel edges will be stored separately from tunnels.

cluster_radii *directory*
Location: out/data/cluster_radii
The directory with tunnel radii of individual tunnels. These data are used for visualization of tunnel dynamics in VMD.

Related parameters:

- *save_dynamics_visualization* yes | no—specifies whether the dynamic visualization of tunnels will be generated.

tree.txt
Location: out/data/tree.txt
The file containing a tree hierarchy of tunnels (i.e., clustering results).

Related parameters:

- *load_tree* yes | no—specifies whether the tree hierarchy of tunnel clusters will be calculated or loaded from the disk.

training.arff
Location: out/data/training.arff
The file with the training data for approximate clustering.

Related parameters:

- *do_approximate_clustering* yes | no—specifies whether the approximate clustering will be applied.

times.txt
Location: out/data/times.txt
The file containing the calculation times of individual computation steps.

start_zone.pdb
Location: out/data/start_zone.pdb
The PDB file with points representing boundary between the starting zone and middle zone of the tunnels. It can be visualized in PyMOL using the out/pymol/zones.py script.

Related parameters:

- *save_zones* yes | no—specifies whether the start_zone.pdb and end_zone.pdb files will be created.

end_zone.pdb
Location: out/data/end_zone.pdb
The PDB file with points representing boundary between the ending and middle zone of the tunnels. It can be visualized in PyMOL using the out/pymol/zones.py script.

Related parameters:

- *save_zones* yes | no—specifies whether the start_zone.pdb and end_zone.pdb files will be created.

origins.pdb

Location: out/data/origins.pdb

The PDB file with initial starting points (specified by the user) for individual input structures. The initial starting points are, together with the tunnels, visualized in PyMOL using the out/pymol/view_timeless, out/pymol/view.py or out/pymol/view_trajectory.py scripts.

v_origins.pdb

Location: out/data/v_origins.pdb

The PDB file with calculation starting points for all analyzed input structures. The calculation starting points are, together with the tunnels, visualized in PyMOL using the out/pymol/view_timeless, out/pymol/view.py or out/pymol/view_trajectory.py scripts.

trajectory.pdb

Location: out/data/trajectory.pdb

A multi-model PDB file with all input structures, which is used for the visualization of structure dynamics in PyMOL. The trajectory together with the dynamic tunnels can be loaded into PyMOL using the out/pymol/view_trajectory.py script.

Related parameters:

- *generate_trajectory* yes | no —specifies whether the trajectory.pdb file will be created.

G. GUIDED EXAMPLE

Analysis of tunnels in snapshots from MD simulation

This example will show you how to identify and analyze tunnels in snapshots from the MD simulation. For the demo purposes, you will analyze 50 snapshots from the 10 ns MD simulation of haloalkane dehalogenase DhaA. The input structures can be found in the examples/guided_example/md_snapshots directory. If you want to go through the whole analysis, go to the **examples/guided_example/inputs** directory. If you just want to explore the outputs of individual steps, go to the **examples/guided_example/results** directory and analyze the pre-calculated results. Important: If you want to exactly reproduce the results of previous analyses, you have to use the exactly same input files, with the same file names and content (the same number and order of atoms), as well as the same setting of the *seed* parameter.

1. Calculation settings

Go to the examples/guided_example/inputs directory. Open the config.txt file and check the settings of parameters. If you plan to visualize the results in VMD, you should set properly the *path_to_vmd* parameter (Advanced settings).

2. Running CAVER 3.0

Important: To ensure smooth calculation, please check that you have enough operating memory to allocate specified heap space and still maintain memory requirements of your system.

Launch the caver.bat file or type the following command in the command prompt (make sure that you are running the command from the examples/guided_example/inputs directory):
java -Xmx1200m -cp ../../../caver/lib -jar ../../../caver/caver.jar -home ../../../caver -pdb ../md_snapshots -conf ./config.txt -out ./out

Important: If you are getting the "*java is not recognized as an internal or external command, operable program or batch file*" error, try to specify the full path to java executable in the running command (or caver.bat file), e.g., "C:\Program Files\Java\jre1.6.0_06\bin\java" -Xmx1200m ... Alternatively, you may add java to the Path in your Environment Variables.
Important: If you are getting the "*Error occurred during initialization of VM. Could not reserve enough space for object heap. Could not create the Java virtual machine.*" error, you should decrease Java heap size or use 64bit Java.

In the command prompt, you will see the settings used for the identification of tunnels and the calculation progress:

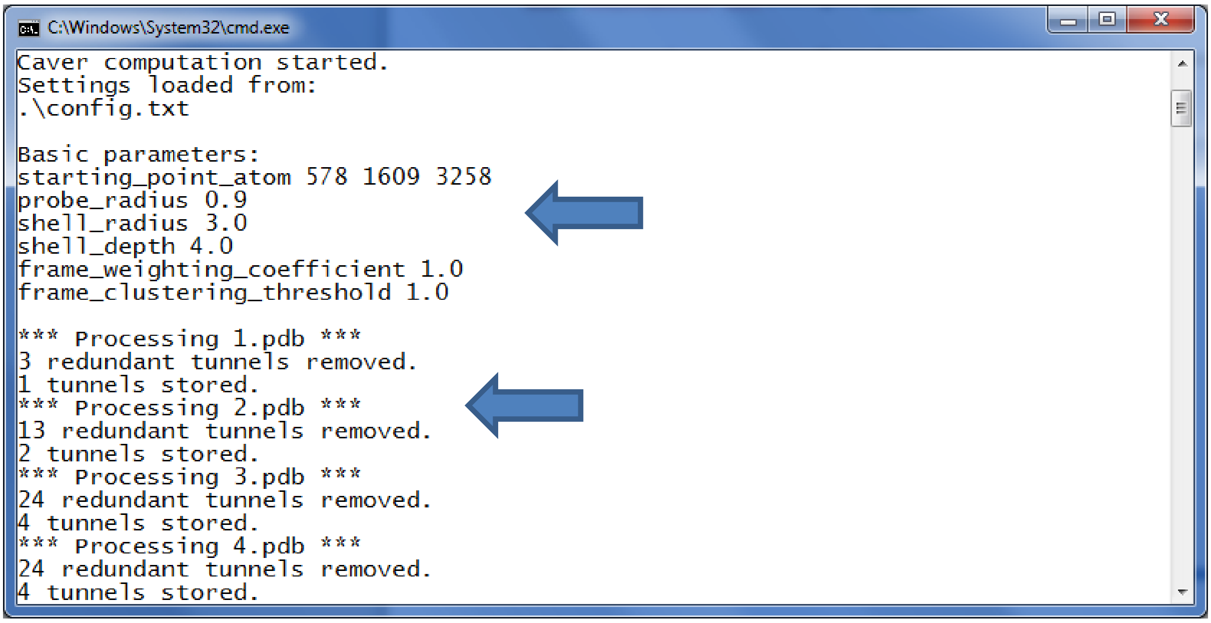

```
C:\Windows\System32\cmd.exe
Caver computation started.
Settings loaded from:
.\config.txt

Basic parameters:
starting_point_atom 578 1609 3258
probe_radius 0.9
shell_radius 3.0
shell_depth 4.0
frame_weighting_coefficient 1.0
frame_clustering_threshold 1.0

*** Processing 1.pdb ***
3 redundant tunnels removed.
1 tunnels stored.
*** Processing 2.pdb ***
13 redundant tunnels removed.
2 tunnels stored.
*** Processing 3.pdb ***
24 redundant tunnels removed.
4 tunnels stored.
*** Processing 4.pdb ***
24 redundant tunnels removed.
4 tunnels stored.
```

After the tunnels are identified in all analyzed snapshots, they are loaded for clustering:

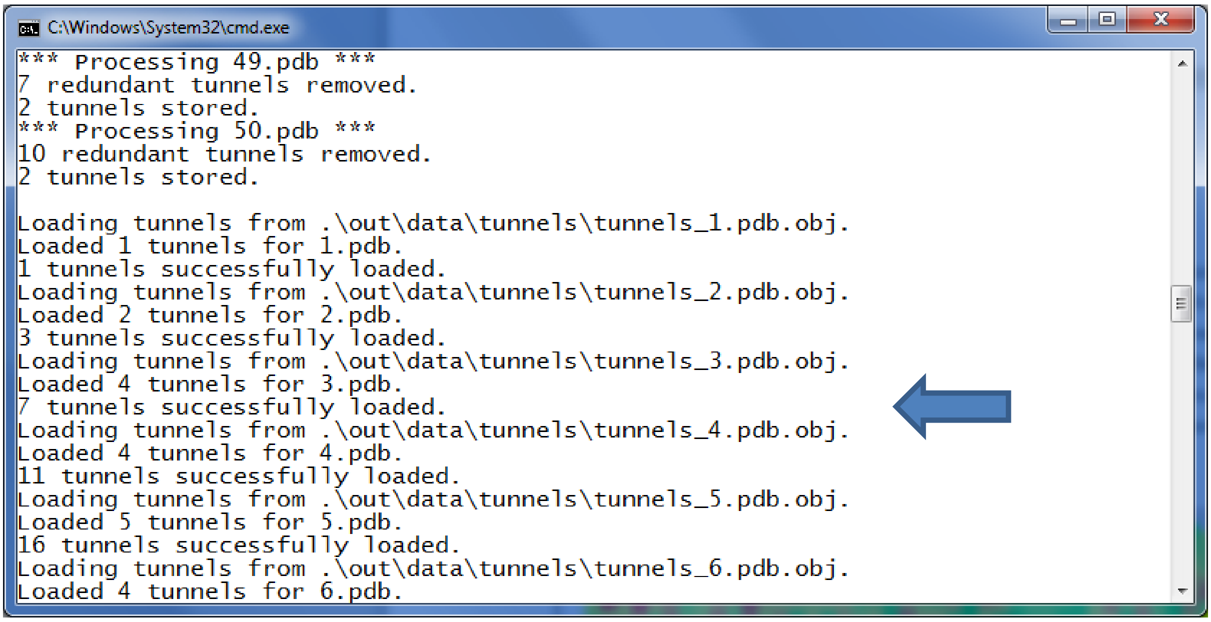

```
C:\Windows\System32\cmd.exe
*** Processing 49.pdb ***
7 redundant tunnels removed.
2 tunnels stored.
*** Processing 50.pdb ***
10 redundant tunnels removed.
2 tunnels stored.

Loading tunnels from .\out\data\tunnels\tunnels_1.pdb.obj.
Loaded 1 tunnels for 1.pdb.
1 tunnels successfully loaded.
Loading tunnels from .\out\data\tunnels\tunnels_2.pdb.obj.
Loaded 2 tunnels for 2.pdb.
3 tunnels successfully loaded.
Loading tunnels from .\out\data\tunnels\tunnels_3.pdb.obj.
Loaded 4 tunnels for 3.pdb.
7 tunnels successfully loaded.
Loading tunnels from .\out\data\tunnels\tunnels_4.pdb.obj.
Loaded 4 tunnels for 4.pdb.
11 tunnels successfully loaded.
Loading tunnels from .\out\data\tunnels\tunnels_5.pdb.obj.
Loaded 5 tunnels for 5.pdb.
16 tunnels successfully loaded.
Loading tunnels from .\out\data\tunnels\tunnels_6.pdb.obj.
Loaded 4 tunnels for 6.pdb.
```

The clustering step involves the calculation of average surface of tunnels, computation of pairwise tunnel distances and average-link hierarchical clustering:

C:\Windows\System32\cmd.exe

```
Loaded 6 tunnels for 45.pdb.
136 tunnels successfully loaded.
Loading tunnels from .\out\data\tunnels\tunnels_46.pdb.obj.
Loaded 1 tunnels for 46.pdb.
137 tunnels successfully loaded.
Loading tunnels from .\out\data\tunnels\tunnels_47.pdb.obj.
Loaded 1 tunnels for 47.pdb.
138 tunnels successfully loaded.
Loading tunnels from .\out\data\tunnels\tunnels_48.pdb.obj.
Loaded 2 tunnels for 48.pdb.
140 tunnels successfully loaded.
Loading tunnels from .\out\data\tunnels\tunnels_49.pdb.obj.
Loaded 2 tunnels for 49.pdb.
142 tunnels successfully loaded.
Loading tunnels from .\out\data\tunnels\tunnels_50.pdb.obj.
Loaded 2 tunnels for 50.pdb.
144 tunnels successfully loaded.

Computing average surface for 144 tunnels.
Average surface represented by 27 points.
Computing distance matrix using weighting_coefficient 1.0.
Performing average link hierarchical clustering to create .\out\data\tree.txt.
144 tunnels clustered into 11 clusters using clustering_threshold 2.0.
```

Once clustering is finished, the output files specified in the config.txt (the summary information, tunnel characteristics and tunnel profiles) are generated. The results are stored in the out directory:

C:\Windows\System32\cmd.exe

```
140 tunnels successfully loaded.
Loading tunnels from .\out\data\tunnels\tunnels_49.pdb.obj.
Loaded 2 tunnels for 49.pdb.
142 tunnels successfully loaded.
Loading tunnels from .\out\data\tunnels\tunnels_50.pdb.obj.
Loaded 2 tunnels for 50.pdb.
144 tunnels successfully loaded.

Computing average surface for 144 tunnels.
Average surface represented by 27 points.
Computing distance matrix using weighting_coefficient 1.0.
Performing average link hierarchical clustering to create .\out\data\tree.txt.
144 tunnels clustered into 11 clusters using clustering_threshold 2.0.

Statistical analysis of results started.
Saving summary information.
Saving tunnel characteristics.
Saving tunnel profiles.
Statistical analysis finished.
Visualizations started.
Cluster subsampling for visualization: random.
Visualizations finished.

Finished successfully.
```

Tip: Try to increase the maximum heap size if you encounter “out of memory” error during the calculation of tunnels. If you want to use heap size larger than 1200m, you will probably need to use 64bit java. Alternatively, you may try to solve the “out of memory” error by setting *number_of_approximating_balls* parameter to a lower value.

3. Analysis of clustering results

First, you should evaluate clustering results. Provided scripts enable you to open the results all at once in PyMOL or VMD. For PyMOL, go to the out/pymol directory. If you have “py” extension associated with PyMOL, you can launch the results by clicking the view_timeless.py file. Alternatively, you can open the results via the PyMOL menu (File > Run > *Path_to_pymol_dir*/view_timeless.py) or PyMOL command line (“run *Path_to_pymol_dir*/view_timeless.py”):

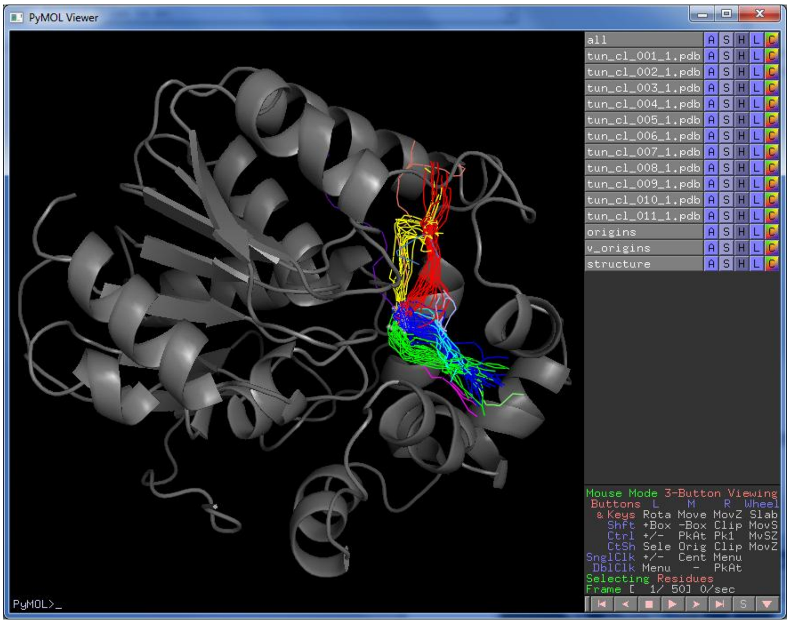

For VMD, go to the out/vmd directory and launch the vmd_timeless.bat script (make sure that the path to the VMD software is correctly specified in the script). Alternatively, you can open the results using the VMD Tk console (Extensions > Tk Console) by typing “cd *Path_to_vmd_dir*” and then “source scripts/view_timeless.tcl”

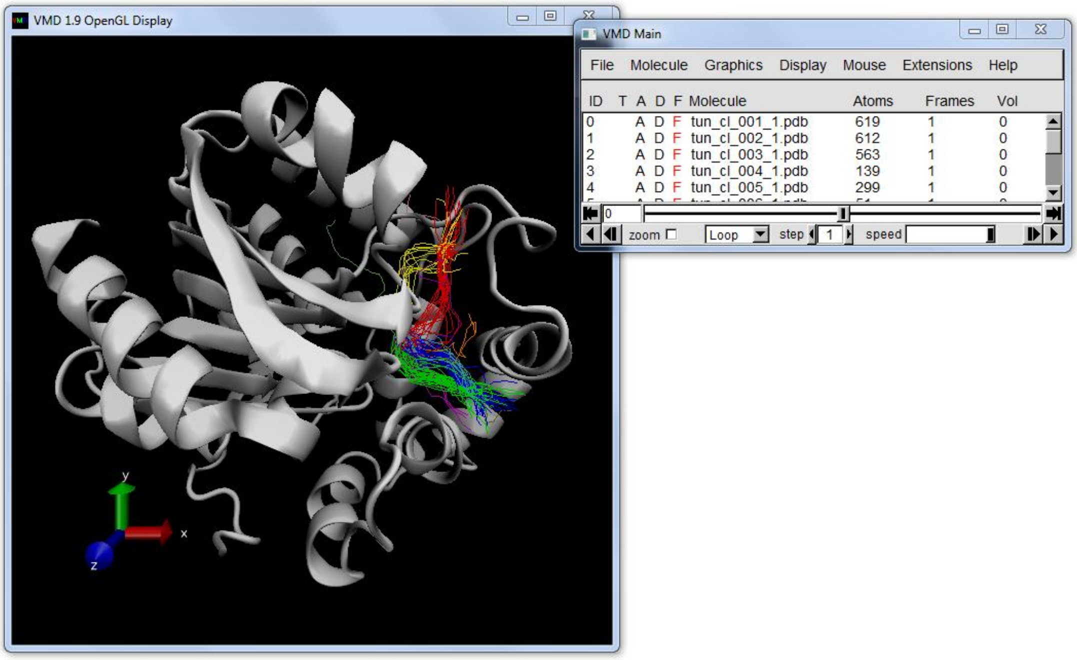

Tunnels identified throughout the MD simulation are all visualized in one frame as tunnel centerlines. Individual tunnel clusters are colored in different colors (the colors can be changed using the standard procedure of a given visualization software). Each tunnel cluster corresponds to one object (molecule); the name of each object includes the ID of a respective tunnel cluster. Important: For large datasets, one cluster may be divided into two or more objects.
Because a low *clustering_threshold* (= 2) was used for clustering, quite similar tunnels are divided into different clusters:

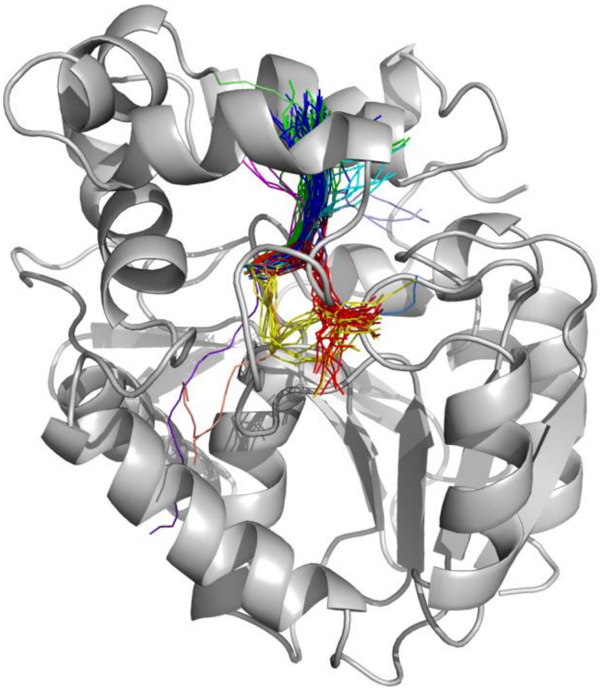

For example, the cluster 1 (dark blue), cluster 2 (green), cluster 4 (cyan), cluster 7 (violet in PyMOL, orange in VMD) and cluster 8 (bright green in PyMOL, olive in VMD) all represent very similar tunnels and should be clustered together. To improve the clustering results, you should increase the *clustering_threshold*.

Important: Using the default settings, the data for visualization of tunnel dynamics are not saved (i.e., the out/pymol/view.py and out/vmd/vmd.bat scripts will not work). If you want to analyze tunnel dynamics, open the config.txt file, set the *save_dynamics_visualization*, *load_tunnels* and *load_cluster_tree* parameters to *yes* and launch the caver.bat (or run the CAVER from the command line).

4. Adjusting clustering_threshold

Open the config.txt file and set the *clustering_threshold* to 4.0. It is not necessary to recalculate the tunnels or repeat the clustering procedure to adjust the clustering results. Therefore, set both the *load_tunnels* and *load_cluster_tree* parameters to *yes* and launch the caver.bat (or run the CAVER from the command line).

You can see in the command prompt that the tunnels are not being identified *de novo,* but loaded from the disk. Similarly, the hierarchy of tunnel clusters is loaded from the out/data/tree.txt file, which was obtained in previous analysis:

```
C:\Windows\System32\cmd.exe
Caver computation started.
Loading tunnels from .\out\data\tunnels\tunnels_1.pdb.obj.
Loaded 1 tunnels for 1.pdb.
1 tunnels successfully loaded.
Loading tunnels from .\out\data\tunnels\tunnels_2.pdb.obj.
Loaded 2 tunnels for 2.pdb.
3 tunnels successfully loaded.
Loading tunnels from .\out\data\tunnels\tunnels_3.pdb.obj.
Loaded 4 tunnels for 3.pdb.
7 tunnels successfully loaded.
Loading tunnels from .\out\data\tunnels\tunnels_4.pdb.obj.
Loaded 4 tunnels for 4.pdb.
11 tunnels successfully loaded.
Loading tunnels from .\out\data\tunnels\tunnels_5.pdb.obj.
Loaded 5 tunnels for 5.pdb.
16 tunnels successfully loaded.
Loading tunnels from .\out\data\tunnels\tunnels_6.pdb.obj.
Loaded 4 tunnels for 6.pdb.
20 tunnels successfully loaded.
Loading tunnels from .\out\data\tunnels\tunnels_7.pdb.obj.
Loaded 4 tunnels for 7.pdb.
24 tunnels successfully loaded.
Loading tunnels from .\out\data\tunnels\tunnels_8.pdb.obj.
Loaded 5 tunnels for 8.pdb.
```

The loaded cluster hierarchy will be cut at the 4.0 threshold, and the new output files, reflecting the new composition of tunnel clusters, will be generated:

```
C:\Windows\System32\cmd.exe
Loading tunnels from .\out\data\tunnels\tunnels_48.pdb.obj.
Loaded 2 tunnels for 48.pdb.
140 tunnels successfully loaded.
Loading tunnels from .\out\data\tunnels\tunnels_49.pdb.obj.
Loaded 2 tunnels for 49.pdb.
142 tunnels successfully loaded.
Loading tunnels from .\out\data\tunnels\tunnels_50.pdb.obj.
Loaded 2 tunnels for 50.pdb.
144 tunnels successfully loaded.

Loading old cluster tree from .\out\data\tree.txt
144 tunnels clustered into 5 clusters using clustering_threshold 4.0.

Statistical analysis of results started.
Saving summary information.
Saving tunnel characteristics.
Saving tunnel profiles.
Statistical analysis finished.
Visualizations started.
Cluster subsampling for visualization: random.
Visualizations finished.

Finished successfully.
```

Launch the out/pymol/view_timeless.py or out/vmd/vmd_timeless.bat scripts to visualize the new results in PyMOL or VMD, respectively. You can see that several previously separated clusters are now joined together.

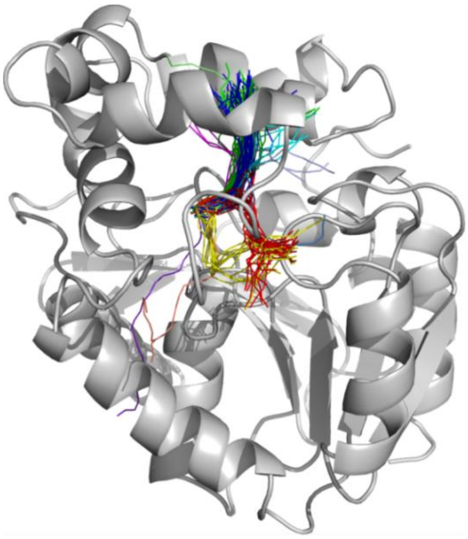

clustering_threshold 2.0

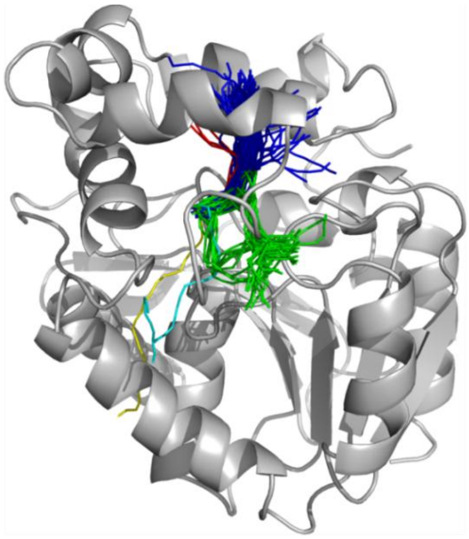

clustering_threshold 4.0

If you are still not satisfied with the clustering results, you can further continue with optimizing the *clustering_threshold* parameter. For example, the tunnels from the cluster 2 (green) have a common opening to the bulk solvent, and in fact represent alternative connections between this opening and the active site. In this sense, these two tunnels can be considered as two variants of one pathway. If we wanted to analyze the relative importance of these two pathways to each other, we would have to set the *clustering_threshold* to a lower value (e.g., 3.0), resulting in the subdivision of the green cluster into two clusters. In this demo, we will further analyze the results obtained using the *clustering_threshold* of 4.0.

Important: Using the default settings, the data for visualization of tunnel dynamics are not saved (i.e., the out/pymol/view.py and out/vmd/vmd.bat scripts will not work). If you want to analyze tunnel dynamics, open the config.txt file, set the *save_dynamics_visualization* to *yes* and launch the caver.bat (or run the CAVER from the command line).

5. Generation of a complete set of output files

As soon as you are satisfied with the clustering results, you can generate all output files, including the files with higher demands on memory and computation time that were not generated during the optimization procedure. Open the config.txt file, go to the “Generation of outputs” section and set the following parameters:

- *one_tunnel_in_snapshot* cheapest
- save_dynamics_visualization yes
- *generate_histograms* yes
- *generate_bottleneck_heat_map* yes
- *generate_profile_heat_map* yes
- *compute_tunnel_residues* yes
- *compute_bottleneck_residues* yes

Then move to the "Advanced settings" and change the following parameters:

- *compute_errors* yes
- *save_error_profiles* yes
- *generate_trajectory* yes

Make sure, that both the *load_tunnels* and *load_cluster_tree* parameters are set to *yes* and then launch the caver.bat file (or run the CAVER from the command line). You may notice on the screen that besides the summary information, tunnel characteristics and profiles, additional output files are generated. These "new" outputs include the information about tunnel-lining residues and atoms, tunnel bottlenecks, heat maps and histograms. The most demanding step is the calculation of maximum possible errors caused by the approximation of the input structure's atoms by a set of balls. All output files are stored in the out directory.

```
C:\Windows\System32\cmd.exe
Loading tunnels from .\out\data\tunnels\tunnels_50.pdb.obj.
Loaded 2 tunnels for 50.pdb.
144 tunnels successfully loaded.

Loading old cluster tree from .\out\data\tree.txt
144 tunnels clustered into 5 clusters using clustering_threshold 4.0.

Leaving only the cheapest tunnel per cluster per snapshot - 83 tunnels kept.
Statistical analysis of results started.
Saving summary information.
Saving profile heat maps.
Saving bottleneck heat maps.
Saving tunnel characteristics.
Saving tunnel-lining residues and atoms.
Saving bottlenecks.
Saving tunnel profiles.
Saving histograms.
Statistical analysis finished.
Visualizations started.
Cluster subsampling for visualization: random.
Visualizations finished.

Finished successfully.
```

6. Analysis of identified tunnels

Overview of identified tunnels

Launch the out/pymol/view_timeless.py or out/vmd/vmd_timeless.bat scripts to visualize the final tunnel clusters, and explore the variability of identified tunnels in PyMOL or VMD, respectively. You may notice the difference between the clusters you have obtained now and the clusters obtained during the optimization steps (see 4. Adjusting clustering_threshold)—some of the previously visible tunnels are not visible now. The difference is caused by the fact that earlier you visualized all identified tunnels (this option is recommended for the evaluation of clustering results), while now only the best (i.e., cheapest) tunnel from each tunnel cluster is reported in individual snapshots. This option (*one_tunnel_in_snapshot* cheapest) is recommended for the interpretation of data. (If you consider also other "non-cheapest" pathways as relevant for the purpose of your study, you should separate them from the dominant tunnels by decreasing the *clustering_threshold* and analyze them as a separate tunnel cluster).

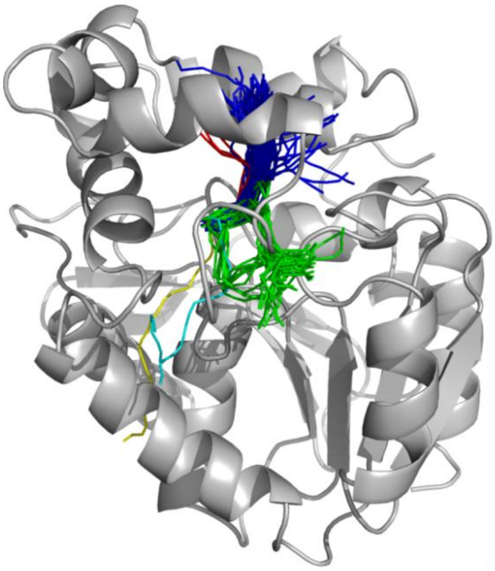

one_tunnel_in_snapshot no

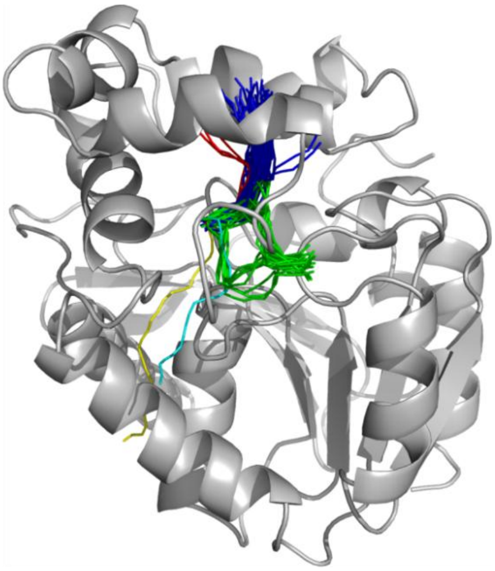

one_tunnel_in_snapshot cheapest

You can also visually analyze tunnel dynamics. For that, launch the out/vmd/vmd.bat script, which will open the MD trajectory together with identified tunnels in VMD. Short trajectories can also be visualized in PyMOL using either the out/pymol/view_trajectory.py script (opens both the trajectory and dynamic tunnels) or out/pymol/view.py script (opens dynamic tunnels but only one static structure). Warning: Depending on your system, opening of large data may cause PyMOL to crash).

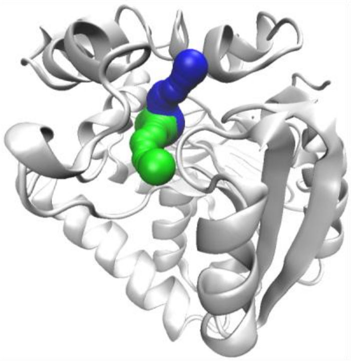

snapshot 44

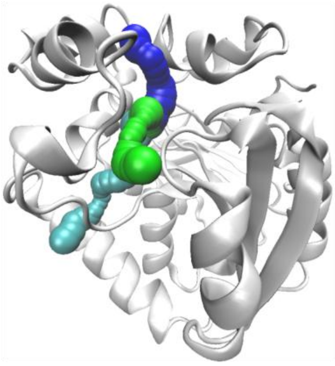

snapshot 45

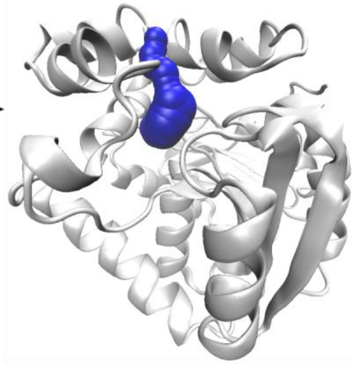

snapshot 46

Comparison of characteristics of individual tunnel clusters

Open the out/summary.txt file and explore the summary characteristics of all identified tunnel clusters:

ID	No	No_snaps	Avg_BR	SD	Max_BR	Avg_L	SD	Avg_C	SD
1	50	50	1.414	0.245	2.33	13.978	1.618	1.308	0.109
2	28	28	1.066	0.124	1.48	16.896	1.671	1.328	0.102
3	3	3	0.934	0.019	0.95	15.650	1.502	1.488	0.153
4	1	1	0.926	0.000	0.93	19.944	0.000	1.259	0.000
5	1	1	0.905	0.000	0.91	23.930	0.000	1.330	0.000

The tunnels from the first ranked tunnel cluster (cluster 1) were identified in all 50 snapshots, which means that they have the bottleneck radius equal or larger than the used *probe_radius* of 0.9 Å. The tunnels from the second best-ranked cluster were identified in approximately half of the analyzed snapshots, while the tunnels from remaining three clusters were rarely identified. The Avg_BR column shows that the first cluster has the highest average bottleneck radius. Note that the values of average bottleneck are overestimated for the clusters 2–5 since averages are calculated only over snapshots where some tunnel from a given cluster was identified. The average values should be therefore interpreted with caution. The Max_BR column shows the maximum opening of the tunnel bottleneck throughout the entire MD simulation. The values indicate that both the cluster 1 and cluster 2 were open enough to enable passage of water molecules (considering the usual probe radius of water molecule of 1.4 Å). The Avg_C column suggests that the tunnel cluster 4 is the straightest from the identified clusters, however this should be again interpreted with caution since this cluster is represented by only one tunnel (more snapshots would need to be analyzed to provide more reliable data). The summary.txt file further provides priorities and average throughputs of individual tunnel clusters, as well as the information about the maximal approximation errors (i.e., the maximum overestimation of tunnel radii caused by the approximation of input structure).

Analysis of tunnel-lining atoms and residues

To get an overview of residues and atoms making up individual tunnel clusters, open the out/analysis/residues.txt file. For each tunnel cluster, all residues and atoms that were at least in one snapshot found within the 3 Å distance from some ball of a given tunnel cluster are reported:

```
== Tunnel cluster 1 ==
# C     res  AA       N   sideN atoms
  X      38 ASN      50      50 50:H_570 50:CA_571 50:CB_573 50:HB3_575 50:CG_
  X     103 ASP      50      50 50:CA_1603 50:CB_1605 50:HB2_1606 50:CG_1608 5
  X     104 TRP      50      50 50:CD1_1621 50:HD1_1622 50:NE1_1623 50:HE1_162
  X     129 ILE      50      50 50:CD1_2025 41:HD13_2028 40:HD11_2026 40:HD12_
  X     138 TRP      50      50 50:HH2_2183 49:HZ2_2181 49:CH2_2182 48:CZ2_218
  X     141 PHE      50      50 50:O_2239 49:C_2238 45:CE2_2234 45:CD2_2236 44
  X     142 ALA      50      50 50:N_2240 50:CA_2242 50:HA_2243 50:CB_2244 50:
  X     146 PHE      50      50 50:CG_2310 50:CD1_2311 50:HD1_2312 50:CE1_2313
  X     165 PHE      50      50 50:CD1_2610 50:CE1_2612 50:HE1_2613 50:CZ_2614
  X     169 ALA      50      50 50:HB1_2668 50:O_2672 49:CB_2667 48:N_2663 48:
  X     172 LYS      50      50 50:CB_2710 50:HB3_2712 50:CD_2716 50:C_2726 49
  X     173 CYS      50      50 50:CA_2730 50:CB_2732 50:HB2_2733 50:HB3_2734
  X     202 PHE      50      50 50:HB3_3237 49:CB_3235 47:C_3249 43:O_3250 38:
  X     203 PRO      50      50 48:CD_3252 48:HD3_3254 48:HA_3262 47:N_3251 47
  X     206 LEU      50      50 49:CD1_3303 49:HD11_3304 49:CD2_3307 48:CG_330
  X     242 VAL      50      50 50:CG1_3857 47:HG13_3860 46:HG11_3858 46:HG12_
  X     243 LEU      50      50 44:CD1_3876 44:CD2_3880 41:CG_3874 40:HD11_387
  X     269 HID      50      50 50:CB_4249 50:HB2_4250 50:HB3_4251 50:CG_4252
  X     270 TYR      50      50 50:OH_4275 50:HH_4276 49:CE1_4272 49:HE1_4273
  X     145 THR      49      49 49:HB_2294 49:CG2_2295 48:CB_2293 48:HG23_2298
[illegible]     149 PHE      49      49 49:HE2_2365 48:CE2_2364 46:CZ_2362 46:HZ_2363
  X     168 GLY      47      34 47:O_2662 46:C_2661 38:CA_2658 34:HA3_2660 21:
```

For example, Gly168 (chain X) was identified as the tunnel-lining residue of the tunnel cluster 1 in 47 snapshots, and the side-chain of this residue was lining the tunnel in 34 snapshots. For each residue, the tunnel-lining atoms are also listed, e.g., the CA atom (atom number 2658) of residue Gly168 was identified as tunnel-lining atom in 38 snapshots.

Time evolution of tunnels

You can further analyze the time evolution of characteristics of individual tunnel clusters. The distributions of bottleneck radii and throughputs of individual tunnel clusters are provided in the out/analysis/histograms directory (bottleneck_histograms.csv and throughput_histograms.csv files).

The out/analysis/bottleneck_heat_maps/bottleneck_heat_map.png file provides a graphical overview of bottleneck radii of individual tunnel clusters:

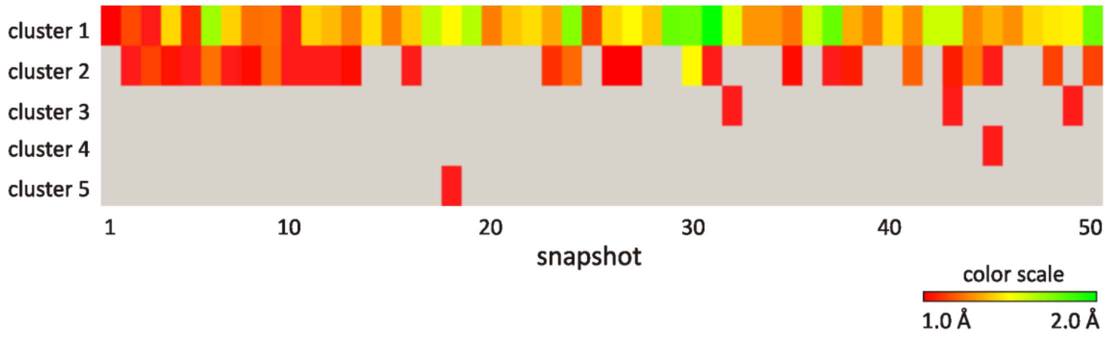

The bottleneck heat map clearly shows that the tunnel cluster 1 is a dominant pathway—it was identified in all snapshots, has the widest bottlenecks and was relatively frequently open to water molecules (i.e., bottleneck radius > 1.4, indicated by yellow and green colors). It also shows that only one of the 28 identified tunnels from the cluster 2 was open to water molecules:

Information about characteristics of each individual tunnel identified throughout the MD simulation is provided in the out/analysis/tunnel_characteristics.csv file. The data in this file are sorted by cluster IDs, so that you can directly plot the graphs showing the time evolution of selected characteristics of individual tunnel clusters. Important: Before you plot graphs, make sure that for each analyzed snapshot, the data are only reported for one tunnel from each tunnel cluster. This is controlled by the "*one_tunnel_in_snapshot* cheapest" option.

Use any spreadsheet editor to open the out/analysis/tunnel_characteristics.csv file and plot the time evolution of bottleneck radius of the cluster 1:

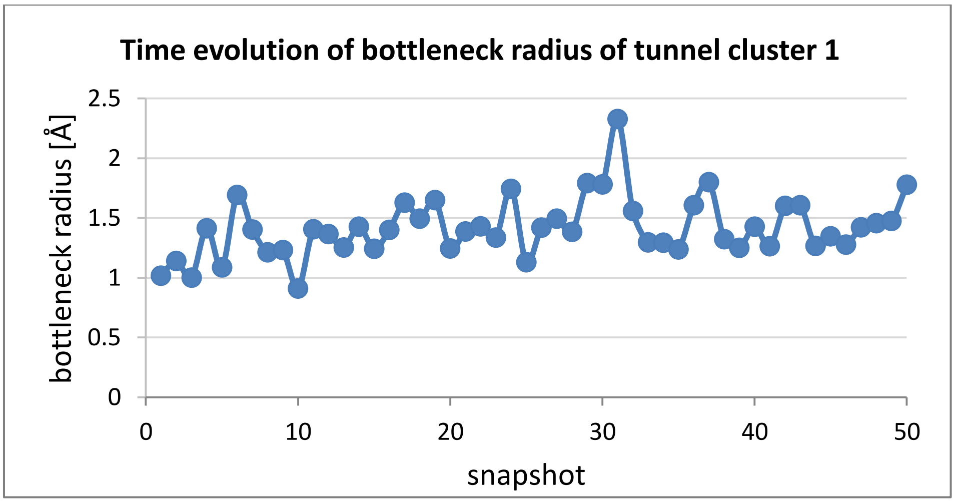

The bottleneck radius of this tunnel cluster ranges from 0.9 Å to 2.3 Å. The largest bottleneck radius from all tunnels from the cluster 1 had the tunnel identified in the snapshot 31, while the tunnel identified in the snapshot 10 had the smallest bottleneck. You can plot and compare the profiles (i.e., tunnel radius over the distance from the starting point measured along the tunnel axis) of these two tunnels using the data provided in the out/analysis/tunnel_profiles.csv file:

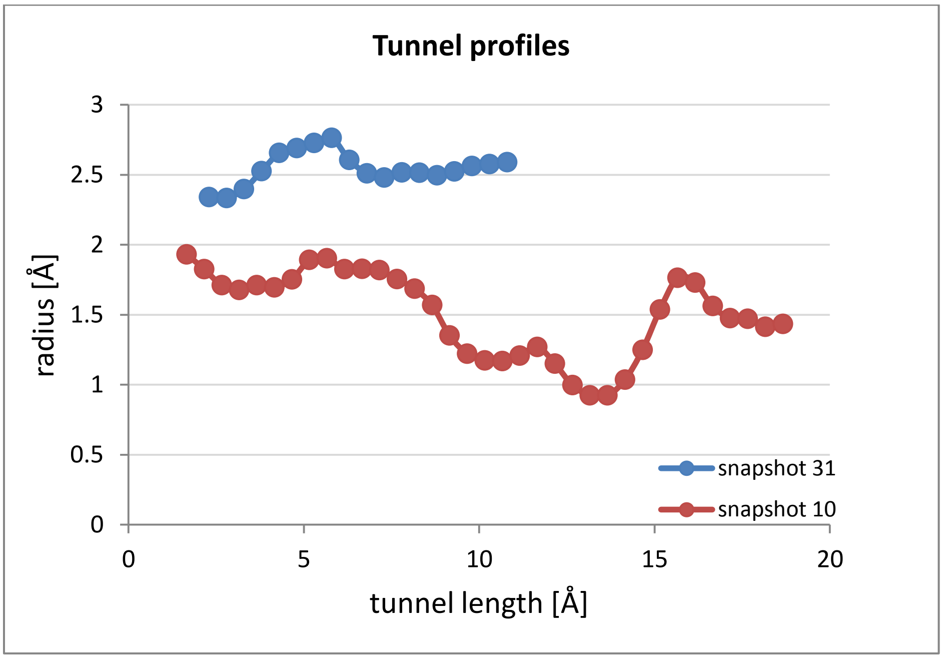

The comparison of both profiles shows significant differences between both tunnels in terms of their radii and length. The tunnel from the snapshot 31 is wide open along its entire length and has no clear bottleneck. The short length of this tunnel suggests that the tunnel will most likely be straight and/or wide open to the bulk solvent. The time evolution of the profiles of individual tunnel clusters can be also analyzed using the heat map visualizations provided in the out/analysis/profile_heat_maps/ directory. To see the profile heat map of the first tunnel cluster, open the cl_000001_profile_heat_map.png file:

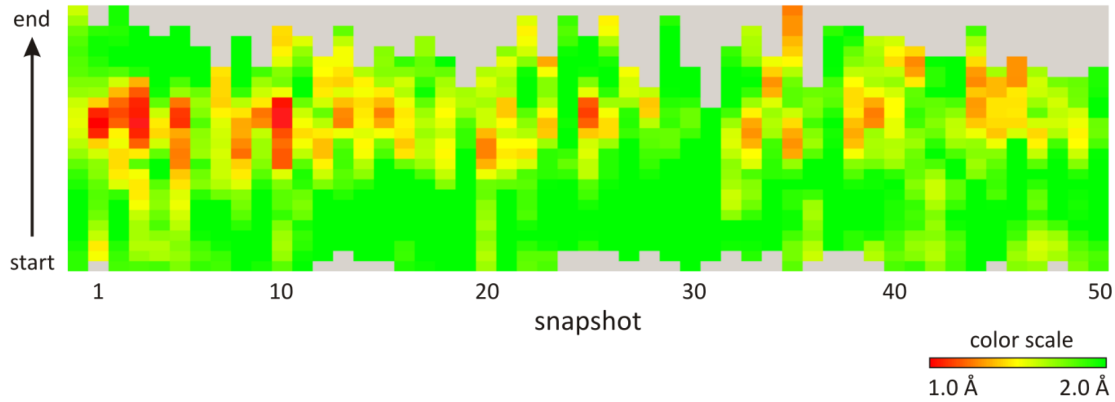

As you can see, the tunnel from the snapshot 31 is green (i.e. wide) along its entire length, while the tunnel from the snapshot 10 has a quite long narrow segment (red color). You may also notice that most of the tunnels have the bottleneck located approximately at 2/3 of the tunnel length.

Analysis of bottlenecks

We will further investigate the differences between the tunnels from snapshot 10 and 31 in the visualization software. Launch the vmd.bat script and go to given snapshots of MD (note that in VMD, the snapshots are numbered from 0; the tunnels from snapshot 10 and 31 thus correspond to the frames 9 and 30, respectively). You will see that both tunnels have very different geometry.

Furthermore, it is often useful to identify and highlight the residues making up the bottlenecks of the tunnels. As was mentioned above, there is no clear bottleneck in the tunnel from snapshot 31, while the tunnel from snapshot 10 has quite long narrow segment in approximately 2/3 of its length. Open the out/analysis/bottlenecks.csv file and find the bottleneck residues of the tunnel from the snapshot 10 (note that all residues located within 3 Å distance from the bottleneck are reported; in most cases, you can consider the three closest resides as the bottleneck residues). You will find that the three closest residues to the bottleneck are residues 142, 146 and 173 (all belonging to the chain X). Try to highlight the residue in VMD and compare their locations in snapshot 10 and 31. You may notice the differences in location of helices carrying the bottleneck residues as well as the change of orientation of Cys173 and Phe146:

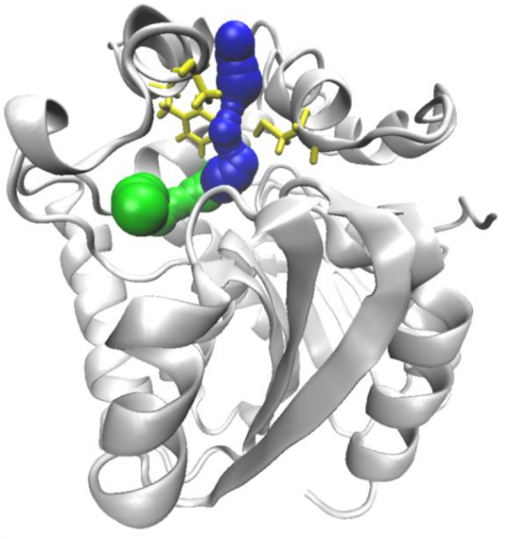

snapshot 10

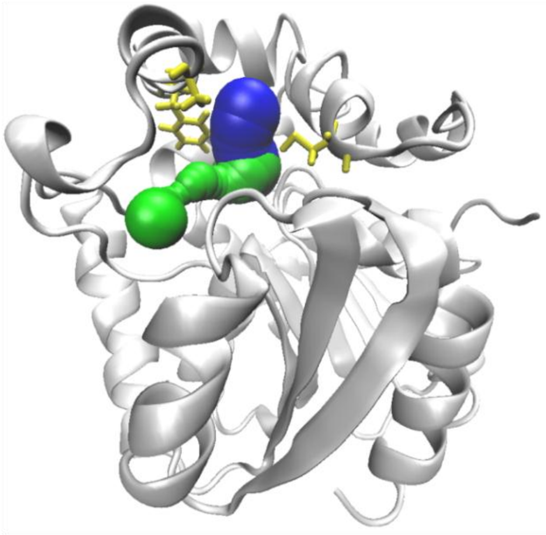

snapshot 31
