# Supplementary material for: CAVER 3.0: A Tool for the Analysis of Transport Pathways in Dynamic Protein Structures
Source: PLoS Comput Biol. 2012 Oct 18;8(10):e1002708. doi: 10.1371/journal.pcbi.1002708 (PMC3475669; doi:10.1371/journal.pcbi.1002708)
Supplement: Software S1 — CAVER 3.0 package containing CAVER 3.0 executable, source code, license, documentation and examples. The latest release of CAVER 3.0 can be downloaded from http://www.caver.cz. (ZIP) [file pcbi.1002708.s006.zip › caver_3.0/examples/QUICK_START/caver_quick_start.pdf]

CAVER 3.0 - Quick start

The quick start briefly introduces the basics of CAVER analysis. For more detailed description of CAVER calculation, optimization of clustering parameters and interpretation of results, go to the **examples/guided_example** directory.

1. Go to the **examples**/**QUICK_START/inputs** directory.

2. Edit the **caver.bat** file: specify the Java heap space (e.g., -Xmx1200m), path to the CAVER library (e.g., -cp ../../../caver/lib), path to the caver.jar file (e.g., -jar ../../../caver/caver.jar), path to the caver home directory (e.g., -home ../../../caver), path to the directory with PDB files (e.g., -pdb ../md_snapshots), and optionally also path to the configuration file (e.g., -conf ./config.txt) and output directory (e.g., -out ./out).

3. **Example**: java -Xmx1200m -cp ../../../caver/lib -jar ../../../caver/caver.jar -home ../../../caver -pdb ../md_snapshots -conf ./config.txt -out ./out
   Important: To ensure smooth calculation, please check that you have enough operating memory to allocate specified heap space and still maintain memory requirements of your system.

4. If you do not want to use example input structures, put your own PDB files into the **../md_snapshots** directory (or specify different directory in the caver.bat file).

5. Specify the calculation starting point and optionally also other parameters in the **config.txt** configuration file (especially *probe_radius*, *shell_radius*, *shell_depth* and *clustering_threshold*). If you plan to visualize the results in VMD, you should also set the *path_to_vmd* parameter.

6. Launch **caver.bat** (in unix/linux systems, make sure that the file is executable) or, alternatively, type the running command directly in the command line.
   Important:
   - If you are getting the *"java is not recognized as an internal or external command, operable program or batch file"* error, try to specify the full path to java executable in the running command (or caver.bat file), e.g., "C:\Program Files\Java\jre1.6.0_06\bin\java". Alternatively, you may add java to the Path in your Environment Variables.
   - If you are getting the "*Error occurred during initialization of VM. Could not reserve enough space for object heap. Could not create the Java virtual machine*." error, you should decrease Java heap size or use 64bit Java.
   - Try to increase the maximum heap size if you encounter "out of memory" error during the calculation of tunnels. If you want to use heap size larger than 1200m, you will probably need to use 64bit java. Alternatively, you may try to solve this problem by setting the *number_of_approximating_balls* parameter to a lower value.

Visualize clustering results in VMD using the out/vmd/**vmd_timeless.bat** (make sure that the path to VMD is correctly set in the script; you can also open the results using the VMD Tk console by typing "cd *Path_to_vmd_dir*" and then "source scripts/view_timeless.tcl"). Alternatively, you can visualize

the clustering results in PyMOL by running the out/pymol/**view_timeless.py** script (you have to associate the extension ".py" with PyMOL first or run the script via PyMOL using the menu File > Run > view_timeless.py or by typing "run view_timeless.py"in the PyMOL command line).

7. Visualize dynamic tunnels in VMD or PyMOL by running out/vmd/**vmd.bat** or out/pymol/**view.py** script, respectively.

8. Explore the tunnel characteristics provided in out/**summary.txt** and out/analysis/**tunnel_characteristics.csv** and out/analysis/**tunnel_profiles.csv.**

9. Use the data provided in out/analysis/**tunnel_profiles.csv** to plot profiles of selected tunnels.

10. Explore the tunnel-lining atoms and residues using the out/analysis/**atoms.txt** and out/analysis/**residues.txt** files.

11. Explore the bottleneck residues of individual tunnels using the out/analysis/**bottlenecks.csv** file.

12. Explore the profile and bottleneck heat maps provided in the out/analysis/**profile_heat_maps** and out/analysis/**bottleneck_heat_maps** directory.

Tip: If you want to adjust the clustering results, just change the *clustering_threshold* parameter. After that, set both the *load_tunnels* and *load_cluster_tree* parameters to *yes* and launch the caver.bat.

Important: If you want to exactly reproduce the results of previous analyses, you have to use the exactly same input files, with the same file names and content (the same number and order of atoms), as well as the same setting of the *seed* parameter.
